# Global, Regional, and Country Incidence of Peritoneal Dialysis-Associated Peritonitis in the Contemporary Peritoneal Dialysis Practice: A Systematic Review and Meta-Analysis

**DOI:** 10.3390/medsci14050592

**Published:** 2026-09-20

**Authors:** Surapon Nochaiwong, Chidchanok Ruengorn, Kajohnsak Noppakun, Kiatkriangkrai Koyratkoson, Mati Chuamanochan, Panjit Chieosilapatham, Pajaree Mongkhon, Nuttaya Wachiraphansakul, Thanawat Vongchaiudomchoke, Tanun Ngamvichchukorn, Kednapa Thavorn, Manish M. Sood, Greg A. Knoll, Apichat Tantraworasin

**Affiliations:** 1 Department of Bioinformatic and Clinical Epidemiology, Faculty of Medicine, Chiang Mai University, Chiang Mai 50200, Thailand; surapon.nochaiwong@cmu.ac.th; 2 Department of Pharmaceutical Care, Faculty of Pharmacy, Chiang Mai University, Chiang Mai 50200, Thailand; chidchanok.r@cmu.ac.th (C.R.); kiatkriangkrai.k@cmu.ac.th (K.K.); 3 Pharmacoepidemiology and Statistics Research Center (PESRC), Chiang Mai University, Chiang Mai 50200, Thailand; kajohnsak.noppakun@cmu.ac.th (K.N.); mati.c@cmu.ac.th (M.C.); panjit.ch@cmu.ac.th (P.C.); pajaree.mo@up.ac.th (P.M.); iewnuttaya@gmail.com (N.W.); thanawat.lp@cpird.in.th (T.V.); tanun@nmu.ac.th (T.N.); kthavorn@ohri.ca (K.T.); 4 Division of Nephrology, Department of Internal Medicine, Faculty of Medicine, Chiang Mai University, Chiang Mai 50200, Thailand; 5 Division of Dermatology, Department of Internal Medicine, Faculty of Medicine, Chiang Mai University, Chiang Mai 50200, Thailand; 6 Department of Microbiology, Faculty of Medicine, Chiang Mai University, Chiang Mai 50200, Thailand; 7 Division of Social and Administrative Pharmacy (SAP), Department of Pharmaceutical Care, School of Pharmaceutical Sciences, University of Phayao, Phayao 56000, Thailand; 8 Department of Internal Medicine, Lampang Hospital, Lampang 52100, Thailand; 9 Division of Nephrology, Department of Medicine, Faculty of Medicine Vajira Hospital, Navamindradhiraj University, Bangkok 10300, Thailand; 10 Ottawa Hospital Research Institute, Ottawa Hospital, Ottawa, ON K1H 8L6, Canada; msood@toh.ca (M.M.S.); gknoll@toh.ca (G.A.K.); 11 School of Epidemiology and Public Health, Faculty of Medicine, University of Ottawa, Ottawa, ON K1G 5Z3, Canada; 12 ICES uOttawa, Institute of Clinical and Evaluative Sciences, Ottawa, ON K1Y 4E9, Canada; 13 Division of Nephrology, Department of Medicine, University of Ottawa, Ottawa, ON K1H 8L6, Canada; 14 Department of Surgery, Faculty of Medicine, Chiang Mai University, Chiang Mai 50200, Thailand

**Keywords:** incidence rate, inequality, infection, peritoneal dialysis, peritonitis, quality indicator, worldwide

## Abstract

**Background/Objectives**: Since peritoneal dialysis (PD) has been used in nephrology practice for several decades, contemporary evidence on global, regional, and country incidence rates of PD-associated peritonitis has not been comprehensively synthesized. We performed a systematic review and meta-analysis to estimate the incidence rates of PD-associated peritonitis worldwide in contemporary PD practice. **Methods**: We systematically searched 6 electronic databases to identify all relevant English-language articles that reported rates of PD-associated peritonitis according to International Society for Peritoneal Dialysis (ISPD) criteria, regardless of age, sex, or PD modality, from 2011 to 2026. We used a random-effects meta-analysis to estimate the pooled incidence rate (episodes per patient-year), with corresponding 95% confidence intervals. We reported summary incidence rates of PD-associated peritonitis for the global estimation (overall rate), World Health Organization (WHO) region, and each country. We also estimated meta-syntheses by causative microorganism of PD-associated peritonitis. **Results**: We identified 12,364 records, of which 153 unique study populations of PD patients fulfilled the study selection criteria. These included over 132,000 PD patients from 37 countries worldwide. Despite substantial heterogeneity, the global incidence rate estimate of PD-associated peritonitis was 0.31 (0.28–0.34) episodes per patient-year (total peritonitis episodes, 88,769; total patient-years at risk, 264,296.30). According to WHO region (*p* for difference between regions <0.001), the regional incidence rates of PD-associated peritonitis were 0.99 (0.68–1.45) for the African Region; 0.38 (0.31–0.45) for the Region of the Americas; 0.41 (0.35–0.48) for the South-East Asia Region; 0.37 (0.30–0.46) for the European Region; 0.39 (0.31–0.49) for the Eastern Mediterranean Region; and 0.21 (0.19–0.24) for the Western Pacific Region. Across 37 different countries, the top three highest incidence rates of PD-associated peritonitis were observed in South Africa, followed by Portugal and Sri Lanka. Meanwhile, the top three lowest incidence rates of PD-associated peritonitis were observed in South Korea, followed by mainland China and Japan. According to the ISPD benchmark, 10 of the 37 included countries had an overall PD-associated peritonitis rate > 0.40 episodes per patient-year. Regarding the causative microorganisms of PD-associated peritonitis, Gram-positive bacteria had the highest pooled incidence rate of PD-associated peritonitis (0.127 [0.113–0.142]), followed by culture-negative (0.073 [0.065–0.083]), Gram-negative bacteria (0.065 [0.058–0.073]), polymicrobial (0.017 [0.014–0.021]), fungus (0.009 [0.008–0.011]), and Mycobacterium (0.004 [0.003–0.006]). **Conclusions**: These global estimates of PD-associated peritonitis incidence rates demonstrated variation across regions and countries. Given differences in healthcare systems across countries, sharing innovations and knowledge for continuous quality improvement and standardizing and harmonizing PD practice settings are warranted to improve quality indicators and patient well-being and reduce inequalities in PD management worldwide.

## 1. Introduction

Since peritoneal dialysis (PD) has been used in nephrology practice for several decades, this dialysis modality is a well-established home kidney replacement therapy option for patients with end-stage kidney disease (ESKD) [1]. To date, PD is available as a treatment for ESKD in 130 countries worldwide [2]. Globally, the median prevalence of PD utilization is 21.0 per million population, ranging from 0.2 per million population in the African region to 126.0 per million population in North and East Asia [2].

Given the flexibility that allows patients with ESKD to undergo dialysis at home with fewer restrictions on daily activities, PD also offers advantages in terms of health-related quality of life, including physical functioning and mental health, and serves as a preferred dialysis modality during the transition to kidney transplantation [1,3,4]. Furthermore, PD treatment for patients with ESKD has also been suggested to be cost-effective compared with in-center hemodialysis, offering good monetary value for healthcare systems, particularly in resource-limited countries [5,6].

Although PD uptake and quality of care have substantially improved, PD-associated peritonitis remains a major complication among the PD population worldwide [3,7,8]. Indeed, PD-associated peritonitis is the leading cause of long-term adverse outcomes, including peritoneal membrane deterioration and malfunction, PD technique failure, all-cause mortality, and cardiovascular death [9,10,11]. Unfortunately, previous systematic reviews have several key limitations, including a focus on specific causative microorganisms (i.e., nontuberculous mycobacterium episodes) [12]; summary results based only on studies conducted in the African region [13]; and syntheses based solely on aggregated annual data reported from country or regional population-based registries and databases, without formal study quality assessment or characterization of PD-related characteristics [14]. Moreover, most reviews summarizing incidence rates of PD-associated peritonitis were published before 2020 and were limited by the number and diversity of PD patients included [12,13,14].

To the best of our knowledge, few up-to-date, comprehensive evidence-based syntheses have addressed global estimates (overall rate), regional rates, and country rates of PD-associated peritonitis in contemporary PD practice. Accordingly, we performed a systematic review and meta-analysis to estimate the worldwide incidence rates of PD-associated peritonitis and provide robust evidence to inform PD communities and to improve quality of PD care.

## 2. Materials and Methods

We followed and reported this systematic review in accordance with the Preferred Reporting Items for Systematic Reviews and Meta-Analyses (PRISMA) 2020 statement (Supplementary File S1) [15]. As part of a systematic review project on the evidence synthesis of PD-related infections, we registered the prespecified protocol in PROSPERO, the International Prospective Register of Systematic Reviews (CRD42020209053). Given the nature of this systematic review and meta-analysis, we did not seek ethics approval because the synthesis was based on existing published data with de-identified and anonymized patient information. To reflect contemporary PD practice, we amended the prespecified protocol to include only full-text studies published from 2011 to 2026. We implemented the amendment on 15 May 2026, before title and abstract screening began and before data extraction or statistical analysis.

### 2.1. Data Source and Search Strategy

We modified search strategies for PD populations and PD-related infections developed by our team [4,16,17,18] and constructed search frameworks and search iterations in consultation with an experienced medical librarian. Using 6 electronic databases (i.e., MEDLINE, Embase, PubMed, Cochrane Library, Scopus, and CINAHL), we identified all relevant abstracts from 2011 to 30 May 2026. We considered only published full-text articles in English. The main keywords and Medical Subject Headings used in the search strategy included “Peritoneal Dialysis” or “Continuous Ambulatory Peritoneal Dialysis” or “Automated Peritoneal Dialysis”, AND “Peritonitis” or “Catheter Infection”. Supplementary Table S1 describes the full systematic search strategy for each database.

### 2.2. Study Selection Criteria and Outcome of Interest

Eligible titles and abstracts of records identified through the systematic literature search were screened independently by pairs of investigators from our team (S.N., K.K., M.C., P.C., P.M., and T.N.). Next, we reviewed potentially relevant full-text articles against the selection criteria. The team resolved any disagreements during article screening through discussion. We documented and reported the final study selection process in line with the PRISMA flow diagram [15].

Supplementary Table S2 describes the details of study selection criteria. Briefly, we included published full-text English-language articles from 2011 to 2026 that (i) included patients with ESKD undergoing chronic PD treatment regardless of age, sex, PD modality, and comorbid conditions; (ii) reported sufficient information to recalculate the overall incidence rate of PD-associated peritonitis; and (iii) reported episodes of PD-associated peritonitis according to criteria consistent with the established ISPD diagnostic criteria; the 2022 ISPD recommendations were used as the contemporary reference framework for eligibility assessment [7]. The diagnostic criteria were based on at least 2 of the following: clinical features consistent with peritonitis (i.e., abdominal pain and/or cloudy dialysis effluent), dialysis effluent white blood cell count > 100 cells/µL [with >50% polymorphonuclear leukocytes], and positive dialysate effluent culture.

We excluded studies that (i) used other criteria for the diagnosis of PD-associated peritonitis (e.g., based on the International Classification of Diseases or read codes) or provided unclear information; (ii) reported only specific causative microorganisms without information on the overall rates of PD-associated peritonitis; (iii) included fewer than 100 PD patients or had a follow-up time or study period < 1 year, to reduce the influence of inflated rates, very small study populations, and PD facilities with low-volume PD uptake and practice; (iv) focused on specific comorbid conditions or etiology of ESKD (e.g., diabetes or glomerular diseases); and (v) were non-human studies, n-of-one, case series/case reports, qualitative studies, reports not involving original data, or records with no full text available.

We reconciled companion studies or post hoc analyses with overlapping patients or study periods with the corresponding primary eligible study populations, retaining the report that provided the most up-to-date and comprehensive information.

### 2.3. Data Extraction and Study Quality Assessment

Pairs of investigators from our team (S.N., K.K., M.C., P.C., P.M., and T.N.) independently extracted the following prespecified information using a standardized approach to collect data on:

(i) Patient and PD treatment characteristics: mean age of the study population, study population (i.e., adult [elderly and non-elderly], pediatric, mixed, or not specified), sex (proportion of males), comorbid conditions (e.g., diabetes), level of education, PD modality (i.e., continuous ambulatory PD [CAPD], automated PD [APD], mixed, or not specified), and number of PD centers included in the study.
(ii) Study characteristics: first author’s name, sample size, study setting and country, year of publication, period of data collection, and data collection approach (i.e., retrospective, prospective, ambispective, or registry data).
(iii) Predefined outcomes: the overall rate of PD-associated peritonitis (i.e., total episodes of peritonitis, total patient-years at risk, overall episodes per patient-year, or patient-months per episode). If available, we also extracted relevant information by causative microorganism (i.e., Gram-positive bacteria, Gram-negative bacteria, fungi, Mycobacterium, polymicrobial, and culture-negative).

For multinational studies, we extracted data based on the relevant information reported for each country. Where information in potentially included studies was unclear or incomplete, we contacted the first or corresponding author via email for clarification. However, if the authors did not respond after 2 attempts, we used the most relevant information reported to perform the required calculations or excluded the study from the formal analyses, as appropriate. Two pairs of investigators from our team (C.R., K.N., N.W., and T.V.) independently cross-checked the final dataset. The team resolved any discrepancies through discussion.

Two investigators (S.N. and C.R.) independently critically appraised the methodological quality of the included studies using the modified Hoy et al. (2012) tool [19]. This appraisal tool consisted of 2 domains: external validity (4 items) and internal validity (6 items). The total summary scores ranged from 0 to 10 points, with 1 point assigned to each item. We classified the summary study quality score as low quality (0 to 4 points), moderate quality (5 to 7 points), and high quality (8 to 10 points). A score of 10 represents the maximum quality score and was categorized as “very high quality” for the sensitivity analysis. We resolved any discrepancies during this process through discussion with third-party investigators (K.N. and A.T.).

### 2.4. Statistical Analyses

We considered differences with a two-tailed *p*-value < 0.05 to be statistically significant. We used Stata software version 18.5 (StataCorp, College Station, TX, USA) for all analyses and the summary pooled incidence rates. For the sample PD cohort studies, we estimated PD-associated peritonitis incidence rates as episodes per patient-year, based on the total number of peritonitis episodes and the corresponding total patient-years at risk reported for each unique PD study population.

For the main meta-analysis, incidence rate estimates with corresponding 95% confidence intervals (CIs) were reported globally (overall rate), for each World Health Organization (WHO) region (i.e., African Region, Region of the Americas, South-East Asia Region, European Region, Eastern Mediterranean Region, and Western Pacific Region), and for each country. For additional meta-analyses, we also estimated incidence rates of PD-associated peritonitis by causative organism group (i.e., Gram-positive bacteria, Gram-negative bacteria, fungi, Mycobacterium, polymicrobial, and culture-negative).

Given differences in methodological approaches and substantial between-study heterogeneity, we used random-effects meta-analysis of natural logarithm-transformed incidence rates to estimate pooled incidence rates, expressed as episodes per patient-year [20]. We used the corresponding standard errors of the log-transformed incidence rates for inverse-variance weighting and estimated between-study variance using restricted maximum likelihood. We calculated pooled estimates and corresponding 95% CIs on the log scale and then back-transformed them to episodes per patient-year. We also calculated 95% prediction intervals on the log scale and back-transformed them to episodes per patient-year to characterize the range of incidence rates expected in comparable study populations, incorporating between-study heterogeneity [21]. For the meta-analysis, we explored statistical heterogeneity between studies using the *Q* statistic with *p*-value, *I*^2^ index (95% CI), and tau-squared (*τ*^2^) [22,23]. We categorized statistical heterogeneity as low (*I*^2^ of 25.0%, *τ*^2^ of 0.04), moderate (*I*^2^ of 50.0%, *τ*^2^ of 0.16), or high (*I*^2^ of 75.0%, *τ*^2^ of 0.36).

We did not include zero-event incidence-rate estimates in the corresponding meta-analyses. Consequently, no continuity correction was applied. For the main analysis, all included study populations reported at least one episode of PD-associated peritonitis. For organism-specific analyses, we included estimates only when at least one episode attributable to the respective organism group was reported. Thus, we performed the meta-analyses using non-zero incidence-rate estimates without applying an artificial continuity correction.

To explore potential effect modifiers and sources of heterogeneity across the included study populations of PD patients, a preplanned set of subgroup analyses was performed according to patient and study characteristics: age (i.e., adult [elderly and non-elderly], pediatric, mixed, or not specified), sex (male vs. female), diabetes status (yes vs. no), level of education (i.e., illiterate, primary education, secondary education, or higher education), PD modality (CAPD, APD, mixed, or not specified), number of PD centers included in the study (single, 2–25, or >25 centers), sample size (100–500, 501–1000, or >1000 patients), year of publication (2011–2015, 2016–2020, or 2021–2026), period of data collection (before the coronavirus disease-19 [COVID-19] pandemic, before 2020 vs. peri/during the COVID-19 pandemic and post-emergency/post-COVID-19 pandemic, i.e., 2020 onward), and data collection approach (i.e., retrospective, prospective, ambispective, or registry data).

To address the robustness of the main meta-analysis of global rates of PD-associated peritonitis, we performed post hoc sensitivity analyses by: (i) restricting the analysis to studies that directly reported PD-associated peritonitis episodes along with causative microorganisms; (ii) restricting the analysis to studies that represented a national PD population; and (iii) restricting analysis to studies that were judged to be very high study quality (summary score of 10 points).

When sufficient data were available (at least 5 estimates per meta-analysis), we visualized asymmetry funnel plots and Doi-Galbraith plots. The preplanned testing for potential small-study effects was based on (i) the global rate estimate (overall pooled rate) of PD-associated peritonitis; (ii) pooled rates according to WHO regions (i.e., African Region, Region of the Americas, South-East Asia Region, European Region, Eastern Mediterranean Region, and Western Pacific region); (iii) pooled rates according to causative microorganisms (i.e., Gram-positive bacteria, Gram-negative bacteria, fungi, Mycobacterium, polymicrobial, and culture negative); and (iv) PD study populations (i.e., adult, pediatric, mixed, or not specified). We exploratorily assessed potential small-study effects for each estimate using Begg’s and Egger’s regression tests (*p*-value < 0.100 suggested publication bias) [24,25], along with the Luis Furuya-Kanamori index (values exceeding 1 or −1 suggested publication bias) [26]. We also used a nonparametric trim-and-fill method to adjust effect estimates when publication bias was detected [27].

## 3. Results

### 3.1. Search Findings and Study Selection

**Figure 1:**
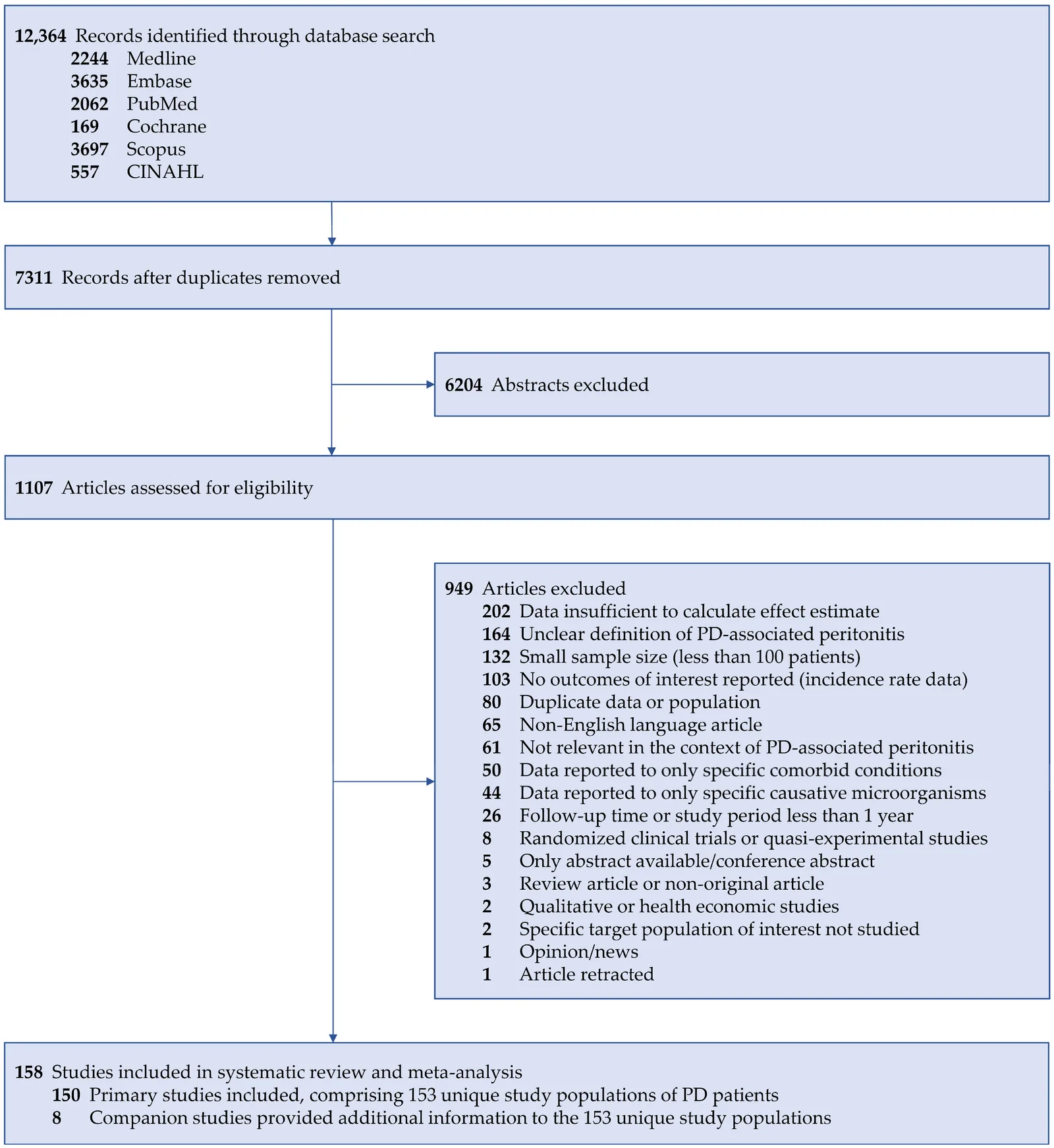
The PRISMA Flowchart on the Selection of Eligible Studies. Abbreviation: PD, peritoneal dialysis.

Initially, we identified 12,364 records from 6 electronic databases (Figure 1). After removing 5053 duplicates, 7311 records remained for the next stage of reviewing the literature evidence. Following title and abstract screening, 1107 were identified as potentially relevant full-text articles. Based on the study selection criteria, after screening and reassembling studies with overlapping study populations, 158 published studies were eligible for this systematic review and meta-analysis (details and reasons for excluding the 949 ineligible studies are described in Supplementary Table S3). Of the 158 included studies, 150 comprised 153 primary populations with the most recent updated information [28,29,30,31,32,33,34,35,36,37,38,39,40,41,42,43,44,45,46,47,48,49,50,51,52,53,54,55,56,57,58,59,60,61,62,63,64,65,66,67,68,69,70,71,72,73,74,75,76,77,78,79,80,81,82,83,84,85,86,87,88,89,90,91,92,93,94,95,96,97,98,99,100,101,102,103,104,105,106,107,108,109,110,111,112,113,114,115,116,117,118,119,120,121,122,123,124,125,126,127,128,129,130,131,132,133,134,135,136,137,138,139,140,141,142,143,144,145,146,147,148,149,150,151,152,153,154,155,156,157,158,159,160,161,162,163,164,165,166,167,168,169,170,171,172,173,174,175,176,177], and 8 companion studies [178,179,180,181,182,183,184,185] provided additional information to the primary study populations.

### 3.2. Key Features of the Included Study Populations

Based on studies published between 2011 and 2026, Table 1 summarizes the 153 unique study populations of PD patients worldwide [28,29,30,31,32,33,34,35,36,37,38,39,40,41,42,43,44,45,46,47,48,49,50,51,52,53,54,55,56,57,58,59,60,61,62,63,64,65,66,67,68,69,70,71,72,73,74,75,76,77,78,79,80,81,82,83,84,85,86,87,88,89,90,91,92,93,94,95,96,97,98,99,100,101,102,103,104,105,106,107,108,109,110,111,112,113,114,115,116,117,118,119,120,121,122,123,124,125,126,127,128,129,130,131,132,133,134,135,136,137,138,139,140,141,142,143,144,145,146,147,148,149,150,151,152,153,154,155,156,157,158,159,160,161,162,163,164,165,166,167,168,169,170,171,172,173,174,175,176,177], with data collected between 1981 and 2024. In total, we included 132,213 PD patients (missing information for 3 studies) from 37 countries worldwide. The mean (SD) age was 54.5 (11.1) years, with adult populations being the most common (86 studies [56.2%]), followed by unspecified PD populations (44 studies [28.7%]), mixed populations (16 studies [10.5%]), and pediatric populations (7 studies [4.6%]). The mean (SD) proportion of male participants was 55.9% (8.4%), ranging from 37.3% to 80.0%. Most studies included PD patients who were treated with either APD or CAPD (mixed PD modalities; 82 studies [53.6%]), followed by CAPD (35 studies [22.9%]), unspecified treatment modality (34 studies [22.2%]), and APD (2 studies [1.3%]). Most included studies used retrospective data collection (103 studies [67.3%]), were conducted before the COVID-19 pandemic (122 studies [79.7%]), and were primarily conducted at a single PD center (100 studies [65.4%]). Supplementary Table S4 describes the characteristics of each study population included in this systematic review and meta-analysis.

According to WHO region, the included studies were conducted in the African Region (2 studies, *n* = 292) [62,93], the Region of the Americas (21 studies, *n* = 30,425) [35,41,43,44,46,47,57,65,71,75,85,95,99,105,117,129,139,144,150,163], the South-East Asia Region (16 studies, *n* = 13,837) [32,33,34,39,45,63,68,100,108,116,133,134,136,146,151,157], European region (41 studies, *n* = 29,987 [missing information for 2 studies) [28,29,30,36,37,38,40,42,48,56,58,59,67,70,73,76,84,86,87,101,103,104,111,113,117,118,119,120,123,137,140,145,147,156,158,160,165,167,168,169,172], the Eastern Mediterranean Region (12 studies, *n* = 4244) [64,78,90,96,102,107,109,122,152,155,164,175], and the Western Pacific Region (60 studies, *n* = 45,032 [missing information for 1 study) [31,49,50,51,52,53,54,55,60,61,66,69,72,74,79,80,81,82,83,88,89,91,92,94,97,98,106,110,112,114,115,117,121,124,125,126,127,128,130,131,132,135,138,141,142,143,148,149,153,154,159,161,162,166,170,171,173,174,176,177]. Of the 37 countries included in this meta-analysis, most studies were conducted in mainland China (23 studies, *n* = 14,041).

With respect to the study quality of the 153 unique study populations included in this meta-analysis (Table 1), all included studies were rated as high quality, with summary scores ranging from 8 to 10 points (3 studies had 8 points [2.0%]; 112 studies had 9 points [73.2%]; and 38 studies had 10 points [24.8%]). The major limitation of the included studies was limited external validity due to national representation or close representation of the target PD populations (Supplementary Table S5).

### 3.3. Global, Regional, and Country Incidence Rate Estimates of PD-Associated Peritonitis

**Figure 2:**
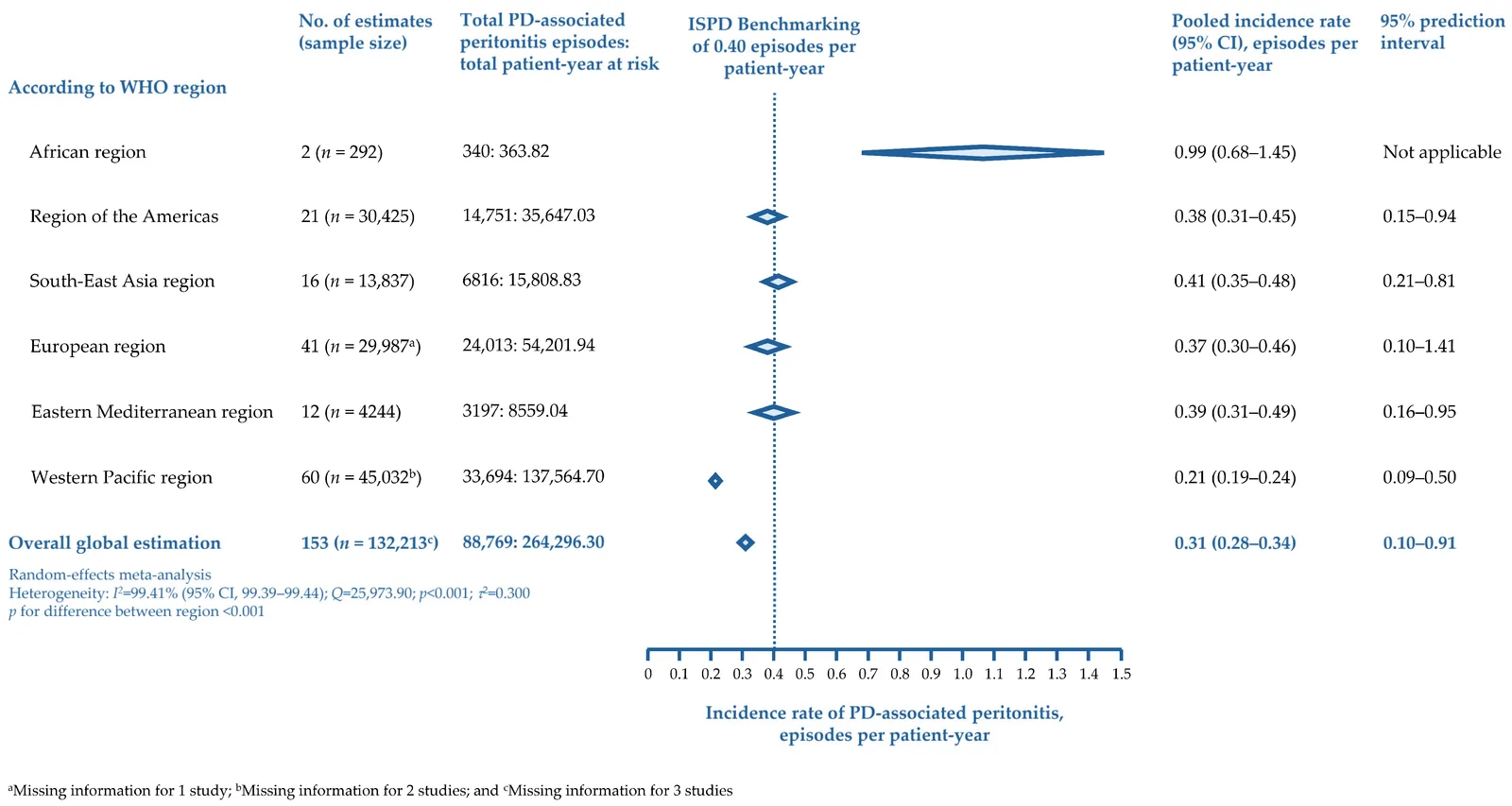
Global and Regional Rates Estimation of PD-associated peritonitis. Abbreviations: CI, confidence interval; ISPD, International Society for Peritoneal Dialysis; PD, peritoneal dialysis; WHO, World Health Organization.

Table 2 summarizes the incidence rate estimates of PD-associated peritonitis in episodes per patient-year. Overall, we found substantial heterogeneity across all pooled incidence rate estimates of PD-associated peritonitis (*I*^2^ ranged from 74.37% to 99.80% with all *p*-values for heterogeneity <0.001 and *τ*^2^ ranging from 0.068 to 0.631, indicating high heterogeneity). Based on the included 153 different PD populations worldwide (*n* = 132,213, with missing information for 3 studies; total peritonitis episodes = 88,769; total patient-years at risk = 264,296.30; Table 2; Figure 2; and Supplementary Figure S1), the pooled global incidence rate of PD-associated peritonitis was 0.31 episodes per patient-year (95% CI, 0.28–0.34).

By WHO region (*p* for difference between regions < 0.001) (Table 2; Figure 2; and Supplementary Figure S2), the regional incidence rates of PD-associated peritonitis were (i) 0.99 episodes per patient-year (95% CI, 0.68–1.45) for the African Region (*n* = 292); (ii) 0.38 episodes per patient-year (95% CI, 0.31–0.45) for the Region of the Americans (*n* = 30,425); (iii) 0.41 episodes per patient-year (95% CI, 0.35–0.48) for the South-East Asia Region (*n* = 13,837); (iv) 0.37 episodes per patient-year (95% CI, 0.30–0.46) for the European Region (*n* = 29,987 with missing information for 2 studies); (v) 0.39 episodes per patient-year (95% CI, 0.31–0.49) for the Eastern Mediterranean Region (*n* = 4244); and (vi) 0.21 episodes per patient-year (95% CI, 0.19–0.24) for the Western Pacific Region (*n* = 45,032 with missing information for 1 study).

**Figure 3:**
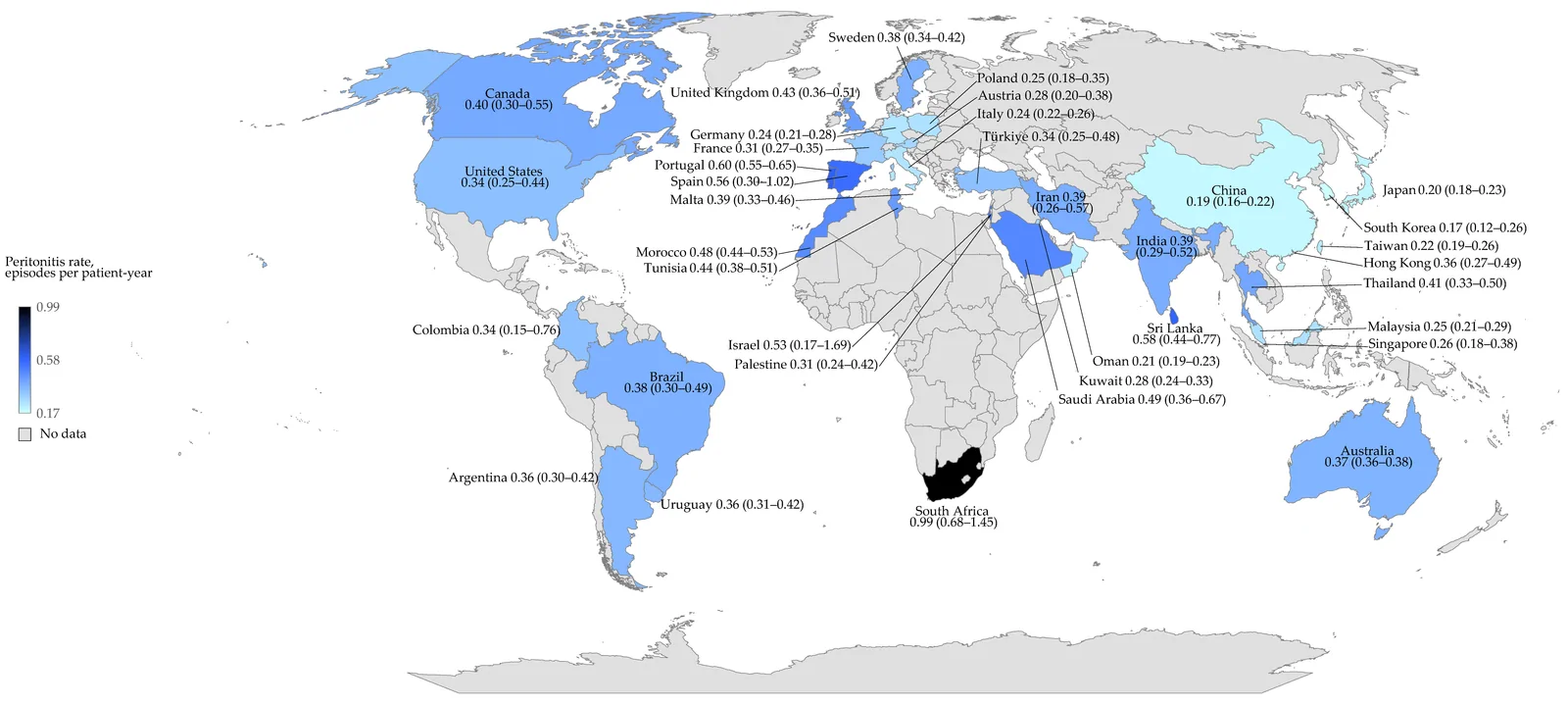
Rates Estimation of PD-Associated Peritonitis with Corresponding 95% CIs, by World Countries. Abbreviations: CI, confidence interval; PD, peritoneal dialysis.

Across the 37 countries included (Figure 3 and Supplementary Table S6), the top three highest incidence rates of PD-associated peritonitis were South Africa (0.99 episodes per patient-year; 95% CI, 0.68–1.45; 2 studies; *n* = 292), followed by Portugal (0.60 episodes per patient-year; 95% CI, 0.55–0.65; 1 study; *n* = 438), and Sri Lanka (0.58 episodes per patient-year; 95% CI, 0.44–0.77; 1 study; *n* = 116). The three lowest incidence rates of PD-associated peritonitis were observed in South Korea (0.17 episodes per patient-year; 95% CI, 0.12–0.26; 7 studies; *n* = 3658), followed by mainland China (0.19 episodes per patient-year; 95% CI, 0.16–0.22; 23 studies; *n* = 14,041), and Japan (0.20 episodes per patient-year; 95% CI, 0.18–0.23; 10 studies; *n* = 6912). According to the 2022 ISPD benchmark, 10 of the 37 included countries had an overall PD-associated peritonitis rate >0.40 episodes per patient-year: South Africa, Portugal, Sri Lanka, Spain, Israel, Saudi Arabia, Morocco, Tunisia, the United Kingdom, and Thailand (Supplementary Table S6).

### 3.4. Causative Microorganism of PD-Associated Peritonitis

**Figure 4:**
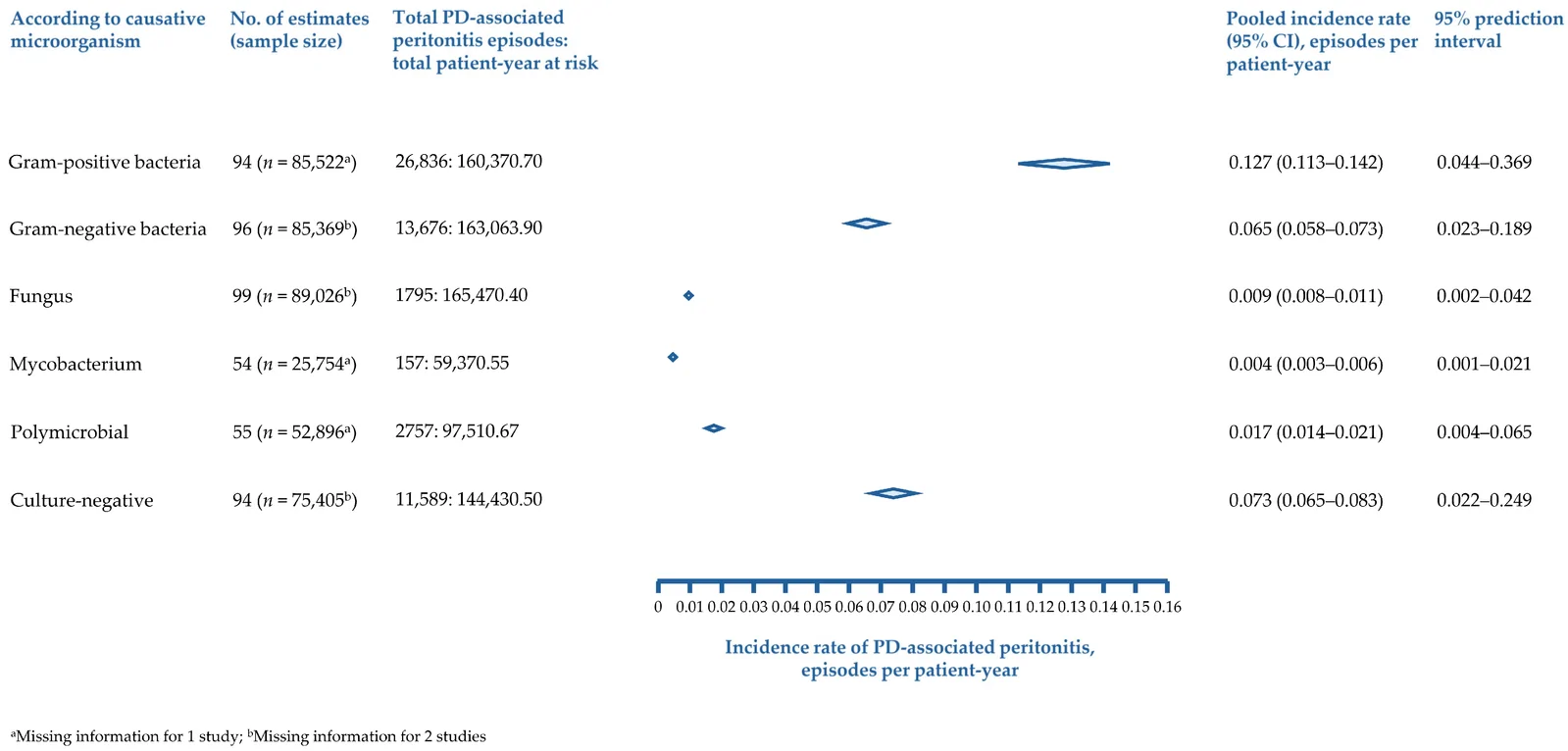
Rates Estimation of PD-Associated Peritonitis by Causative Microorganisms. Abbreviations: CI, confidence interval; PD, peritoneal dialysis.

With substantial heterogeneity across causative microorganism groups (Table 2; Figure 4; and Supplementary Figure S3), the pooled incidence rates of PD-associated peritonitis were (i) 0.127 episodes per patient-year (95% CI, 0.113–0.142) for Gram-positive bacteria (94 studies; *n* = 85,522, with missing information for 1 study); (ii) 0.065 episodes per patient-year (95% CI, 0.058–0.073) for Gram-negative bacteria (96 studies; *n* = 85,369, with missing information for 2 studies); (iii) 0.009 episodes per patient-year (95% CI, 0.008–0.011) for fungus (99 studies; *n* = 89,026 with missing information for 2 studies); (iv) 0.004 episodes per patient-year (95% CI, 0.003–0.006) for Mycobacterium (29 studies; *n* = 16,684, with missing information for 1 study); (v) 0.017 episodes per patient-year (95% CI, 0.014–0.021) for polymicrobial (55 studies; *n* = 52,896 with missing information for 1 study); and (vi) 0.073 episodes per patient-year (95% CI, 0.065–0.083) for culture-negative (94 studies; *n* = 75,405, with missing information for 2 studies), with *p* for difference between groups of causative microorganisms < 0.001.

### 3.5. Subgroup Analysis, Sensitivity Analysis, and Assessment of Potential Small-Study Effects

In the subgroup analyses, which showed high between-study heterogeneity (Table 2), differences by effect modifiers were observed for study population and publication year (*p* for the difference between subgroups = 0.017 and 0.001, respectively). Of these, the incidence rate of PD-associated peritonitis was highest in pediatric populations (7 studies; *n* = 5083; pooled incidence rate of 0.46 episodes per patient-year [95% CI, 0.36–0.58]). Based on the publication years of the 153 included studies, the estimated incidence rate of PD-associated peritonitis gradually decreased from the 2011–2015 period (48 studies; *n* = 38,813, with missing information for 1 study; pooled incidence rate of 0.37 episodes per patient-year [95% CI, 0.34–0.42]) to the 2021–2026 period (57 studies; *n* = 52,359, with missing information for 2 studies; pooled incidence rate of 0.28 episodes per patient-year [95% CI, 0.24–0.33]).

All post hoc sensitivity analyses of incidence rate estimates for PD-associated peritonitis were similar and did not substantially differ from the main findings (all *p*-values for testing compared to the main analysis >0.05; Supplementary Table S7). Across multiple statistical approaches, we identified potential small-study effects for the overall global incidence rate estimate or when focusing on WHO regions, causative microorganism, and study population (Supplementary Table S8). These findings were also suggested by asymmetry in the funnel plot visualizations (Supplementary Figure S4). However, given the substantial between-study heterogeneity, funnel-plot asymmetry may reflect potential small-study effects or other sources of heterogeneity rather than publication bias alone. However, in an additional exploratory trim-and-fill analysis, the pooled incidence rate estimate of PD-associated peritonitis did not substantially alter the findings (Supplementary Table S8).

## 4. Discussion

### 4.1. Overview of the Findings

To the best of our knowledge, this systematic review and meta-analysis is the first to comprehensively summarize up-to-date evidence on the global incidence rate of PD-associated peritonitis in contemporary PD practice. Based on data from over 132,000 PD patients across 37 countries, including 88,769 peritonitis episodes and 264,296.30 patient-years at risk, the pooled global incidence rate was 0.31 (95% CI, 0.28–0.34) episodes per patient-year, with Gram-positive bacteria identified as the most common causative microorganisms of PD-associated peritonitis. Given the substantial heterogeneity, the meta-analysis findings appear to vary across regional and country contexts. Moreover, the incidence rates of PD-associated peritonitis were higher in pediatric populations, and lower rates were observed in more recent publication-year groups. As a quality indicator in PD management and to promote long-term PD program sustainability, our findings underscore wide variation and inequalities in PD-associated peritonitis rates across WHO regions and countries worldwide.

### 4.2. Contextualizing Findings with Relevant Evidence

Collectively, the three existing systematic reviews summarized information regarding the occurrence of PD-related peritonitis based on the literature before 2020 [12,13,14]. Without providing a formal rate estimate, the first review by Song et al. (2012) [12] focused only on nontuberculous mycobacterial episodes, which were identified in 57 PD patients (57.9% in the United States, followed by Asian countries [26.3%] and European countries [10.5]). The second systematic review, by Okpechi et al. (2020) [13], focused on the African Region and reported wide variation in PD-associated peritonitis rates, ranging from 0.33 to 2.72 episodes per patient-year. The review also found a higher rate in the pediatric population than in the adult population (1.78 vs. 0.63 episodes per patient-year). Moreover, Gram-positive bacteria were the most common pathogens causing peritonitis (37.0%). Without formal study quality assessment or relevant information on PD characteristics, another systematic review by Marshall (2022) [14] synthesized evidence based on PD populations in 33 countries using aggregated annual data reported from national or regional population-based registries, illustrating wide variation in PD-associated peritonitis rates that decreased from 0.60 episodes per patient-year in 1992 to 0.30 episodes per patient-year in 2019.

Considering the substantial methodological and statistical heterogeneity, our systematic review and meta-analysis updated and estimated global, regional, and country-level rates of PD-associated peritonitis in contemporary PD practice using data from 153 unique study populations of PD patients in 37 countries worldwide. PD-associated peritonitis rates ranged from 0.17 (95% CI, 0.12–0.26) episodes per patient-year in South Korea to 0.99 (95% CI, 0.68–1.45) episodes per patient-year in South Africa. Our global estimates of PD-associated peritonitis align with previous reviews and appear to have decreased over time, with lower rates observed in the most recent publication-year group (2021–2026 compared with 2011–2015). Meanwhile, these rates appeared to be higher in pediatric PD populations than in adult PD populations.

Given differences in systematic review methodologies and the availability of updated evidence, we expanded existing reviews by providing a comprehensive overview of global, regional, and country-level incidence estimates of PD-associated peritonitis, as well as estimates by causative microorganism, across more diverse PD populations worldwide. For cross-country comparisons, we also found that over one-fourth (27.0%) of the countries included had PD-associated peritonitis rates above an international benchmark of >0.40 episodes per patient-year, according to the ISPD statement [7]. We considered that genuine variations in country-level estimates of PD-associated peritonitis may reflect differences in healthcare systems. These variations in PD-associated peritonitis rates may also reflect differences and potential inequalities in PD management and practice, including healthcare resources, national policies for PD implementation, and dialysis preparedness and infrastructure [2,11]. At the PD population level, differences in PD-associated peritonitis rates may also reflect variation in social risk, social determinants of health (SDOH), and patient vulnerability. For instance, children may have higher rates of PD-associated peritonitis than adults because of caregiver-dependent procedures or more frequent opportunities for touch contamination, which may make it more difficult to maintain hygiene and aseptic technique and could increase bacterial exposure.

### 4.3. Strengths and Limitations

In this meta-analysis, we used a rigorous, comprehensive approach to provide an up-to-date overview of evidence on global and regional estimates of PD-associated peritonitis across 37 countries. Methodologically, we used stringent study selection criteria, including only published peer-reviewed articles with an adequate sample size (≥100 PD patients) that met the ISPD criteria for diagnosing PD-associated peritonitis. As a result, we judged all included studies to be of high quality. Our findings expanded on and addressed the limitations of previous systematic reviews, such as summary findings based solely on specific causative microorganisms, limited to particular regions, or relying on aggregated annual registry data reported without the context of patient and PD characteristics [12,13,14]. We also expanded the meta-analysis by including more studies with diverse PD populations worldwide and considered global and regional levels, including comparisons between countries for international quality indicator benchmarking. To account for substantial heterogeneity across countries, we estimated PD-associated peritonitis incidence rates using a random-effects model to estimate pooled rates representing an average across the included PD populations. Finally, sensitivity analyses were consistent with the main meta-analysis, supporting the robustness of the effect estimates.

Despite the up-to-date evidence and rigorous systematic review and meta-analytic approach, several limitations must be acknowledged when interpreting the findings. First, despite an extensive search framework and iteration, information for some WHO regions, particularly the African region, was limited. Meanwhile, most PD patients included in this meta-analysis were from North and East Asia, particularly mainland China. These results appear to reflect global PD uptake and utilization, particularly in countries with established PD programs and policy-supported implementation, with both incidence and prevalence of chronic PD highest in North and East Asia and lowest in Africa [2]. However, cross-country comparisons should be interpreted cautiously and should not be considered definitive evidence of differences in national performance or international disparities.

Second, we could not comprehensively perform preplanned subgroup analyses based on key patient and PD characteristics (i.e., specific age groups, sex, comorbid conditions, and education level) because of limited available data. Relatively few data were available from the post-COVID-19 era. Moreover, data were poorly reported, with mixed or unspecified values for some variables, such as study population and PD modality, limiting estimation and comparison. Reporting of specific causative microorganisms, as well as microbiological trends and rates of antimicrobial resistance in PD-associated peritonitis across different countries, was also limited. Therefore, global rates of PD-associated peritonitis for these issues cannot be established.

Third, although we included only articles that reported criteria consistent with the ISPD recommendations for PD-associated peritonitis, it should be noted that routinely reported rates of PD-associated peritonitis may not be uniform, for example, in the methods and components used to report outcomes, across PD settings and countries [186]. Although the overall rate of PD-associated peritonitis was lower in more recent publication periods, this finding may reflect changes in study populations, data-collection periods, clinical practice, and other methodological or contextual factors. Because the exact year-by-year distribution of data collection was unavailable for most studies, we used publication year only as a broad descriptive grouping variable. As a result, our findings should not be interpreted as a direct measure of secular trends in peritonitis incidence. Based on the ISPD recommendation, we found that 80.9% of the included studies reported a proportion of culture-negative episodes >15% of total PD-associated peritonitis episodes. Thus, culture-negative episodes remain a recognized concern for laboratory practices and reporting quality.

Fourth, despite a comprehensive search approach, excluding data from non-English articles may have limited the generalizability of our findings to real-world PD practice settings. In addition, most effect estimates showed asymmetric funnel plots, and several statistical assessments suggested potential small-study effects and funnel-plot asymmetry. Given the substantial between-study heterogeneity, however, these findings should be interpreted cautiously, as funnel-plot asymmetry may arise from factors other than publication bias, including genuine heterogeneity and differences in study populations, study designs, and methodological characteristics. Nevertheless, the exploratory trim-and-fill analyses did not substantially alter the pooled effect estimates, suggesting the overall findings were relatively robust to potential small-study effects. However, these analyses cannot establish the presence or absence of publication bias. To broaden the evidence base and reduce the risk of missing relevant published studies, we searched 6 biomedical databases using a comprehensive search strategy.

Finally, we identified substantial statistical heterogeneity among the included studies in effect estimates. Moreover, wide prediction intervals accompanied all effect estimates, indicating substantial clinical diversity and inconsistency across settings. Although we performed several sensitivity analyses and subgroup analyses concerning effect modifiers of patient and study characteristics, substantial heterogeneity persisted. As noted above, in addition to heterogeneity in healthcare systems across countries and PD settings, we observed differences in reporting methods in this systematic review and meta-analysis. This heterogeneity in estimated incidence rates likely reflect substantial methodological and clinical differences across the included studies in study design, patient populations, PD practice, data collection and reporting methods, healthcare settings, and data collection periods, rather than solely statistical variability. We also note that our analyses cannot fully explain the genuine variation in PD-associated peritonitis rate estimates across countries. Given these limitations, our findings should be interpreted cautiously.

### 4.4. PD Practice Considerations, Implications for Public Health, and Future Perspectives

To date, PD serves as an alternative and affordable dialysis modality in various countries worldwide. This meta-analysis of PD-associated peritonitis incidence rates provides additional information to improve performance on PD quality indicators by comparing countries worldwide. Beyond variation within individual PD populations and across PD centers, differences in PD-associated peritonitis rates across countries may reflect social risk factors, SDOH gradients, and environmental differences that shape cross-country health inequalities and healthcare disparities.

Despite the limitations of our findings, this meta-analysis provides the best available evidence to inform the temporal epidemiology of a key global quality indicator of PD treatment performance and to implement targeted policy initiatives and public interventions that eliminate health gaps and health inequalities across countries, which may help improve rates of PD-associated peritonitis. Sharing innovations and knowledge for continuous quality improvement between PD centers and countries, particularly from higher-performing settings, including gap identification, best practice adoption, data-driven goals with evidence-based performance standards, cultural changes in PD training and monitoring, and resource allocation and optimization, could facilitate and mitigate widening in rates of PD-associated peritonitis and promote well-being and PD program sustainability worldwide.

Moreover, standardizing and harmonizing PD practice settings and the reporting of quality indicators are warranted, particularly for culture-negative PD-associated peritonitis episodes. We advocate for integrated policy initiatives and continuous quality improvement programs through collaboration among public health sectors, governments, and non-governmental organizations at national and international levels to promote high-quality PD care and management worldwide. To address these global challenges, future research on regional and country-level effects on health gaps and inequalities in PD management, including the prevention and treatment of PD-associated peritonitis globally, is needed to better understand and target interventions to regional- and country-specific needs.

## 5. Conclusions

In conclusion, this systematic review and meta-analysis provides a comprehensive overview of the global incidence of PD-associated peritonitis, a key quality indicator in patients with ESKD receiving PD. PD-associated peritonitis remains an important clinical challenge in contemporary PD practice, with widening rates across regions and countries. Given heterogeneity in healthcare systems across countries, sharing innovations and knowledge to support continuous quality improvement, together with greater standardization and harmonization of PD practice, may help improve patient well-being and reduce inequalities in PD management worldwide.

## Figures and Tables

**Table 1:** Summary of the 153 Unique Study Populations of PD Patients Worldwide ^a^.

Characteristics of Included Studies	Number of Studies (%)
Mean age in year, grand mean (SD); median (min–max); missing information for 15 studies (9.8%)	54.5 (11.1); 56.1 (7.7–78.6)
Study population	
Adult	86 (56.2)
Pediatric	7 (4.6)
Mixed	16 (10.5)
Not specified	44 (28.7)
% Male, mean (SD); median (min–max); missing information for 12 studies (7.8%)	55.9 (8.4); 55.8 (37.3–80.0)
PD modality	
CAPD	35 (22.9)
APD	2 (1.3)
Mixed	82 (53.6)
Not specified	34 (22.2)
PD center, median (min–max)	1 (1–158)
Single center	100 (65.4)
Multicenter: 2–25	38 (24.8)
Multicenter: >25	15 (9.8)
Sample size, median (min–max); missing information for 3 studies (2.0%)	336.5 (100–9699)
100–500	98 (65.3)
501–1000	22 (14.7)
>1000	30 (20.0)
Year of publication	
2011–2015	48 (31.4)
2016–2020	48 (31.4)
2021–2026	57 (37.2)
Period of data collection	
Before 2020 (before COVID-19 pandemic)	122 (79.7)
2020 onward (peri/during COVID-19 pandemic and post-emergency/post-COVID-19 pandemic)	5 (3.3)
Comprised both before and after 2020	26 (17.0)
Data collection approach	
Retrospective	103 (67.3)
Prospective	27 (17.6)
Ambispective	3 (2.0)
Registry data	20 (13.1)
Study quality, mean (SD); median (min–max) ^b^	9.2 (0.5); 9 (8–10)
8 points	3 (2.0)
9 points	112 (73.2)
10 points	38 (24.8)
WHO region: country (no. of studies) ^c^	
African Region: South Africa (2)	2 (1.3)
Region of the Americas: Argentina (1), Brazil (5), Canada (5), Colombia (4), United States (4), Uruguay (1), and mixed Canada and United States (1)	21 (13.8)
South-East Asia Region: India (5), Sri Lanka (1), and Thailand (10)	16 (10.5)
European Region: Austria (2), France (3), Germany (1), Israel (2), Italy (4), Malta (1), Poland (2), Portugal (1), Spain (8), Sweden (1), Türkiye (10), and United Kingdom (6)	41 (27.0)
Eastern Mediterranean Region: Iran (3), Kuwait (1), Morocco (1), Oman (1), Palestine (1), Saudi Arabia (4), and Tunisia (1)	12 (7.9)
Western Pacific Region: Australia (2), China (23), Hong Kong (5), Japan (10), Malaysia (2), Singapore (2), South Korea (7), Taiwan (9), and mixed Australia/New Zealand (1)	60 (39.5)

^a^ Based on 150 included studies, including 1 multinational study that provided 4 unique study populations across 4 countries. ^b^ Based on the modified Hoy et al. quality appraisal tool, with the possible summary score ranging from 0–10 points (see details in Supplement Table S5). ^c^ One multinational registry mixed dataset with PD populations across multiple continents was not counted (the French Peritoneal Dialysis Registry [RDPLF], which includes France [core contributor], Belgium, Switzerland, Algeria, Morocco, Tunisia, and Luxembourg). Abbreviations: APD, automated peritoneal dialysis; CAPD, continuous ambulatory peritoneal dialysis; COVID-19, coronavirus disease-2019; PD, peritoneal dialysis; SD, standard deviation; WHO, World Health Organization.

**Table 2:** Summary Findings of Incidence Rates Estimate of PD-Associated Peritonitis Worldwide.

Meta-Analysis of Evidence	No. of Estimates (Total Sample Size)	Total PD-Associated Peritonitis Episodes: Total Patient-Year at Risk	Incidence Rate Estimation (95% CI), Episodes per Patient-Year	95% Prediction Interval	Heterogeneity				*p* for Difference Between Subgroup
					*Q* Statistic	*p*-Value	*I*^2^ (95% CI), %	*τ* ^2^	
**Main analysis**									
Overall global estimation	153 (*n* = 132,213 ^a^)	88,769:264,296.30	0.31 (0.28–0.34)	0.10–0.91	25,973.90	<0.001	99.41 (99.39–99.44)	0.300	NA
According to WHO region									
African Region	2 (*n* = 292)	340:363.82	0.99 (0.68–1.45)	NA	11.99	0.001	91.70 (NA)	0.068	<0.001
Region of the Americas	21 (*n* = 30,425)	14,751:35,647.03	0.38 (0.31–0.45)	0.15–0.94	2416.01	<0.001	99.17 (99.04–99.29)	0.182	
South-East Asia Region	16 (*n* = 13,837)	6816:15,808.83	0.41 (0.35–0.48)	0.21–0.81	539.18	<0.001	97.22 (96.41–97.84)	0.095	
European Region	41 (*n* = 29,987 ^b^)	24,013:54,201.94	0.37 (0.30–0.46)	0.10–1.41	9414.25	<0.001	99.58 (99.54–99.61	0.425	
Eastern Mediterranean Region	12 (*n* = 4244)	3197:8559.04	0.39 (0.31–0.49)	0.16–0.95	394.06	<0.001	97.21 (96.24–97.93)	0.146	
Western Pacific Region	60 (*n* = 45,032 ^c^)	33,694:137,564.70	0.21 (0.19–0.24)	0.09–0.50	5443.41	<0.001	98.92 (98.81–99.01)	0.177	
**Additional analysis**									
According to causative microorganism									
Gram-positive bacteria	94 (*n* = 85,522 ^c^)	26,836:160,370.70	0.127 (0.113–0.142)	0.044–0.369	7293.71	<0.001	98.72 (98.62–98.82)	0.286	<0.001
Gram-negative bacteria	96 (*n* = 85,369 ^b^)	13,676:163,063.90	0.065 (0.058–0.073)	0.023–0.189	3731.79	<0.001	97.45 (97.19–97.69)	0.284	
Fungus	99 (*n* = 89,026 ^b^)	1795:165,470.40	0.009 (0.008–0.011)	0.002–0.042	1025.79	<0.001	91.23 (89.81–92.44)	0.555	
Mycobacterium	29 (*n* = 16,684 ^c^)	157:42,603.07	0.004 (0.003–0.006)	0.001–0.021	109.23	<0.001	74.37 (63.17–82.16)	0.631	
Polymicrobial	55 (*n* = 52,896 ^c^)	2757:97,510.67	0.017 (0.014–0.021)	0.004–0.065	1094.48	<0.001	95.34 (94.51–96.04)	0.436	
Culture-negative	94 (*n* = 75,405 ^b^)	11,589:144,430.50	0.073 (0.065–0.083)	0.022–0.249	4236.81	<0.001	97.80 (97.58–98.00)	0.373	
**Subgroup analysis**									
Study population									
Adult	86 (*n* = 79,932 ^a^)	50,783:166,327.10	0.29 (0.26–0.32)	0.12–0.69	9306.77	<0.001	99.09 (99.02–99.15)	0.194	0.017
Pediatric	7 (*n* = 5083)	4036:7919.74	0.46 (0.36–0.58)	0.19–1.12	264.05	<0.001	97.73 (96.69–98.44)	0.105	
Mixed	16 (*n* = 9471 ^a^)	6892:17,278.32	0.37 (0.28–0.48)	0.11–1.24	1732.89	<0.001	99.13 (98.97–99.27)	0.299	
Not specified	44 (*n* = 37,727)	27,058:72,771.20	0.31 (0.25–0.38)	0.07–1.30	12,464.43	<0.001	99.66 (99.63–99.68)	0.494	
PD modality									
CAPD	35 (*n* = 17,448)	13,648:46,261.24	0.31 (0.25–0.38)	0.08–1.21	5568.33	<0.001	99.39 (99.32–99.45)	0.437	0.097
APD	2 (*n* = 363)	248:465.00	0.42 (0.16–1.12)	NA	33.79	<0.001	97.00 (NA)	0.489	
Mixed	82 (*n* = 90,369 ^a^)	56,583:150,187.50	0.33 (0.29–0.37)	0.12–0.92	13,667.12	<0.001	99.41 (99.37–99.44)	0.255	
Not specified	34 (*n* = 24,033)	18,290:67,382.61	0.25 (0.20–0.30)	0.07–0.82	5627.30	<0.001	99.41 (99.35–99.47)	0.338	
PD center									
Single center	100 (*n* = 36,040 ^b^)	32,307:117,513.30	0.28 (0.25–0.32)	0.09–0.86	9772.45	<0.001	98.99 (98.91–99.06)	0.309	0.054
Multicenter: 2–25	38 (*n* = 33,990 ^c^)	26,246:66,637.89	0.36 (0.29–0.46)	0.09–1.52	11,873.84	<0.001	99.69 (99.66–99.71)	0.483	
Multicenter: >25	15 (*n* = 62,183)	30,216:80,145.10	0.34 (0.30–0.39)	0.18–0.63	1907.24	<0.001	99.26 (99.13–99.38)	0.075	
Sample size									
100–500	98 (*n* = 23,171)	17,610:60,966.28	0.30 (0.27–0.34)	0.10–0.88	5040.02	<0.001	98.08 (97.90–98.24)	0.285	0.125
501–1000	22 (*n* = 15,057)	9809:35,268.23	0.26 (0.21–0.33)	0.09–0.81	2555.32	<0.001	99.18 (99.05–99.29)	0.276	
>1000	30 (*n* = 93,985)	58,181:159,458.30	0.36 (0.29–0.44)	0.11–1.14	16,683.15	<0.001	99.83 (99.81–99.84)	0.305	
Year of publication									
2011–2015	48 (*n* = 38,813 ^c^)	30,151:66,547.53	0.37 (0.34–0.42)	0.18–0.80	3993.53	<0.001	98.82 (98.69–98.94)	0.138	0.001
2016–2020	48 (*n* = 41,041)	21,123:70,156.58	0.28 (0.25–0.32)	0.11–0.72	4002.27	<0.001	98.82 (98.69–98.95)	0.210	
2021–2026	57 (*n* = 52359 ^b^)	37,495:127,592.20	0.28 (0.24–0.33)	0.07–1.09	15,355.66	<0.001	99.64 (99.61–99.66)	0.450	
Period of data collection									
Before COVID-19 pandemic	125 ^d^ (*n* = 107,222)	76,615:213,322.00	0.32 (0.29–0.35)	0.11–0.95	22,129.83	<0.001	99.44 (99.41–99.47)	0.298	0.473
Peri/during COVID-19 pandemic and post-emergency/post-COVID-19 pandemic	5 (*n* = 1474)	366: 1416.30	0.28 (0.19–0.40)	0.07–1.15	48.23	<0.001	91.71 (83.63–95.80)	0.162	
Data collection approach									
Retrospective	103 (*n* = 60,679 ^b^)	44,200:155,479.60	0.29 (0.26–0.32)	0.10–0.84	12,035.28	<0.001	99.15 (99.09–99.21)	0.277	0.164
Prospective	27 (*n* = 12,573)	4803:15,598.74	0.30 (0.25–0.37)	0.10–0.90	1225.56	<0.001	97.88 (97.46–98.22)	0.268	
Ambispective	3 (*n* = 4648)	2649:7418.08	0.29 (0.20–0.41)	0.00–21.02	17.04	<0.001	88.26 (67.36–95.78)	0.082	
Registry data	20 (*n* = 54,313 ^c^)	37,117:85,799.90	0.41 (0.32–0.52)	0.13–1.32	9690.84	<0.001	99.80 (99.79–99.82)	0.296	

^a^ Missing information for 3 studies. ^b^ Missing information for 2 studies. ^c^ Missing information for 1 study. ^d^ Based on 122 unique study populations of PD patients and 3 companion studies. Abbreviations: APD, automated peritoneal dialysis; CAPD, continuous ambulatory peritoneal dialysis; CI, confidence interval; COVID-19, coronavirus disease-2019; NA, not applicable; PD, peritoneal dialysis; WHO, World Health Organization.

## Data Availability

No new data were created or analyzed in this study. Data sharing is not applicable to this article.

## Supplementary Materials

The following supporting information can be downloaded at: https://www.mdpi.com/article/10.3390/medsci14050592/s1, Table S1: Systematic Review Search Strategy; Table S2: The PICOTS Framework: Study Inclusion/Exclusion Criteria; Table S3: Excluded Studies; Table S4: Description of the 153 Unique Study Populations of PD Patients Included in Meta-Analysis; Table S5: Quality Assessment of the Included Studies for Incidence Rate Estimation of PD-Associated Peritonitis; Table S6: Meta-Analysis of Incidence Rate Estimation of PD-Associated Peritonitis, by Country; Table S7: Sensitivity Analysis of Incidence Rate Estimation of PD-Associated Peritonitis; Table S8: Meta-Analysis of Incidence Rate Estimation of PD-Associated Peritonitis with Calibration for Publication Bias; Figure S1: Meta-Analysis of Global Incidence Rate Estimation of PD-Associated Peritonitis; Figure S2: Meta-Analysis of Incidence Rate Estimation of PD-Associated Peritonitis, by WHO Regions; Figure S3: Meta-Analysis of Incidence Rate Estimation of PD-Associated Peritonitis, by Causative Organisms; Figure S4: Funnel Plot and Doi Plot of Incidence Rate Estimation of PD-Associated Peritonitis; File S1: PRISMA 2020 Statement Checklist; File S2: Study Quality Appraisal Tool; File S3: eReferences.
