# Supplementary material for: Global, Regional, and Country Incidence of Peritoneal Dialysis-Associated Peritonitis in the Contemporary Peritoneal Dialysis Practice: A Systematic Review and Meta-Analysis
Source: Med Sci (Basel). 2026 Sep 20;14(5):592. doi: 10.3390/medsci14050592 (PMC13609861; doi:10.3390/medsci14050592)
Supplement: Supplementary file 1 [file medsci-14-00592-s001.zip › medsci-4550593-supplementary.pdf]

Supplementary Materials

Global, Regional, and Country Incidence of Peritoneal Dialysis-Associated Peritonitis in the Contemporary Peritoneal Dialysis Practice: A Systematic Review and Meta-Analysis

Surapon Nochaiwong, Chidchanok Ruengorn, Kajohnsak Noppakun, Kiatkriangkrai Koyratkoson, Mati Chuamanochan, Panjit Chieosilapatham, Pajaree Mongkhon, Nuttaya Wachiraphansakul, Thanawat Vongchaiudomchoke, Tanun Ngamvichchukorn, Kednapa Thavorn, Manish M. Sood, Greg A. Knoll, Apichat Tantraworasin

Supplementary Online Content

**Table S1**	Systematic Review Search Strategy	S3
**Table S2**	The PICOTS Framework: Study Inclusion/Exclusion Criteria	S9
**Table S3**	Excluded Studies	S10
**Table S4**	Description of the 153 Unique Study Populations of PD Patients Included in Meta-Analysis	S59
**Table S5**	Quality Assessment of the Included Studies for Incidence Rate Estimation of PD-Associated Peritonitis	S68
**Table S6**	Meta-Analysis of Incidence Rate Estimation of PD-Associated Peritonitis, by Country	S74
**Table S7**	Sensitivity Analysis of Incidence Rate Estimation of PD-Associated Peritonitis	S76
**Table S8**	Meta-Analysis of Incidence Rate Estimation of PD-Associated Peritonitis with Calibration for Publication Bias	S77
**Figure S1**	Meta-Analysis of Global Incidence Rate Estimation of PD-Associated Peritonitis	S79
**Figure S2**	Meta-Analysis of Incidence Rate Estimation of PD-Associated Peritonitis, by WHO Regions	S80
**Figure S3**	Meta-Analysis of Incidence Rate Estimation of PD-Associated Peritonitis, by Causative Organisms	S86
**Figure S4**	Funnel Plot and Doi Plot of Incidence Rate Estimation of PD-Associated Peritonitis	S92
**File S1**	PRISMA 2020 Statement Checklist	S111
**File S2**	Study Quality Appraisal Tool	S113
**File S3**	eReferences	S114

Table S1 Systematic Review Search Strategy

**Ovid MEDLINE(R) ALL (1946 to May 30, 2026)**		
**Search**	**Query**	**Items Found**
#1	exp Peritoneal Dialysis/	29690
#2	*dialysis, peritoneal/	16613
#3	(Peritoneal Dialysis or Continuous Ambulatory Peritoneal Dialysis).ti,ab,kf.	31686
#4	exp Continuous Ambulatory Peritoneal Dialysis/	10232
#5	Automated Peritoneal Dialysis.mp	801
#6	(Continuous Ambulatory Peritoneal Dialysis or Automated Peritoneal Dialysis).ti,ab,kf.	7192
#7	or/1-6	38847
#8	Catheter infection$.mp	1433
#9	exp Peritonitis/	29915
#10	Exit Site Infection$.mp	1037
#11	Tunnel infection$.mp	511
#12	(Peritoneum and infection$).ti,ab,kf.	1567
#13	(infectious complication$ or (catheter adj1 infection$) or (catheter adj1 peritonitis) or catheter complication$ or perivisceritis).ti,ab,kf.	20172
#14	(percutaneous access and infection$).ti,ab,kf.	109
#15	((intraperiton* or periton*) and infection$).ti,ab,kf.	32497
#16	or/8-15	77499
#17	7 and 16	8698
#18	(news or newspaper article or letter or interview comment or editorial or review or systematic review or case report or case series).pt.	6167824
#19	17 not 18	6815
#20	limit 19 to humans	6081
#21	limit #20 to articles published in year 2011 to 2026	2244

Table S1 Systematic Review Search Strategy (Continued)

Embase (From Inception to May 30, 2026)		
**Search**	**Query**	**Items Found**
#1	‘peritoneal dialysis’/exp AND [embase]/lim	48902
#2	‘continuous ambulatory peritoneal dialysis’/exp AND [embase]/lim	11205
#3	‘automated peritoneal dialysis’/exp AND [embase]/lim	267
#4	#1 OR #2 OR #3	48902
#5	‘catheter infection*’/exp AND [embase]/lim	22625
#6	(catheter:ti,ab AND infection$:ti,ab) AND [embase]/lim	40111
#7	‘peritonitis’/exp AND [embase]/lim	64920
#8	‘exit site infection*’/exp AND [embase]/lim	1565
#9	‘tunnel infection*’/exp AND [embase]/lim	701
#10	‘peritoneum infection*’/exp AND [embase]/lim	5
#11	‘infectious complication*’/exp AND [embase]/lim	32032
#12	(catheter:ti,ab AND (infection*:ti,ab OR peritonitis*:ti,ab OR complication*:ti,ab OR perivisceritis:ti,ab)) AND [embase]/lim	98132
#13	(‘percutaneous access’:ti,ab AND infection*:ti,ab) AND [embase]/lim	214
#14	((intraperitoneal*:ti,ab OR peritoneal*:ti,ab) AND infection*:ti,ab) AND [embase]/lim	30642
#15	#5 OR #6 OR #7 OR #8 OR #9 OR #10 OR #11 OR #12 OR #13 OR #14	221323
#16	#4 AND #15	13356
#17	(news:it OR ‘newspaper article’:it OR comment:it OR editorial:it OR interview:it OR letter:it OR review:it OR ‘systematic review’:it OR ‘case report’:it OR ‘case series’:it) AND [embase]/lim	4563359
#18	#16 NOT #17	11380
#19	#18 AND ('case control study'/de OR 'clinical article'/de OR 'clinical study'/de OR 'clinical trial'/de OR 'cohort analysis'/de OR 'comparative effectiveness'/de OR 'comparative study'/de OR 'controlled clinical trial'/de OR 'controlled study'/de OR 'diagnostic test accuracy study'/de OR 'double blind procedure'/de OR 'feasibility study'/de OR 'human'/de OR 'longitudinal study'/de OR 'major clinical study'/de OR 'medical record review'/de OR 'multicenter study'/de OR 'observational study'/de OR 'open study'/de OR 'outcomes research'/de OR 'pilot study'/de OR 'proportional hazards model'/de OR 'prospective study'/de OR 'randomized controlled trial'/de OR 'randomized controlled trial topic'/de OR 'retrospective study'/de OR 'total quality management'/de OR 'trend study'/de) AND ('article'/it OR 'article in press'/it OR 'short survey'/it)	6806
#20	limit #19 to articles published in year 2011 to 2026	3635

Table S1 Systematic Review Search Strategy (Continued)

**PubMed (From Inception to May 30, 2026)**		
**Search**	**Query**	**Items Found**
#1	(Peritoneal Dialysis) OR (Peritoneal Dialysis[MeSH Terms])	39682
#2	(((((Continuous Ambulatory Peritoneal Dialysis[Title/Abstract]) OR (Automated Peritoneal Dialysis[Title/Abstract])) OR (Continuous Cycling Peritoneal Dialysis[Title/Abstract])) OR (Intermittent Peritoneal Dialysis[Title/Abstract])) OR (Nocturnal Intermittent Peritoneal Dialysis[Title/Abstract])) OR (Tidal Peritoneal Dialysis[Title/Abstract])	7606
#3	#1 OR #2	39682
#4	((Peritonitis[Title/Abstract]) OR (Exit Site Infection*[Title/Abstract])) OR (Tunnel infection*[Title/Abstract])	39389
#5	(((Peritoneum[Title/Abstract] AND infection*[Title/Abstract]) OR (catheter adj1 peritonitis[Title/Abstract])) OR (perivisceritis[Title/Abstract])) OR (percutaneous access[Title/Abstract] AND infection*[Title/Abstract])	1715
#6	(intraperiton*[Title/Abstract] OR periton*[Title/Abstract]) AND (infection*[Title/Abstract])	32558
#7	#4 OR #5 OR #6	62605
#8	#3 AND #7	9414
#9	(((((Case Reports[Publication Type]) OR Guideline[Publication Type]) OR News[Publication Type]) OR Newspaper Article[Publication Type]) OR Review[Publication Type])	6636905
#10	#8 NOT #9	6278
#11	Filters applied: Humans	5417
#12	limit #11 to articles published in year 2011 to 2026	2062

Table S1 Systematic Review Search Strategy (Continued)

**Cochrane Library (From Inception to May 30, 2026)**		
**Search**	**Query**	**Items Found**
#1	MeSH descriptor: [Peritoneal Dialysis] explode all trees	1176
#2	MeSH descriptor: [Peritoneal Dialysis, Continuous Ambulatory] explode all trees	520
#3	(“Continuous Ambulatory Peritoneal Dialysis” OR “Automated Peritoneal Dialysis” OR “Continuous Cycling Peritoneal Dialysis” OR “Intermittent Peritoneal Dialysis” OR “Nocturnal Intermittent Peritoneal Dialysis” OR “Tidal Peritoneal Dialysis”):ti,ab,kw	726
#4	#1 OR #2 OR #3	1454
#5	(Peritonitis OR “Exit Site Infection” OR “Exit-Site Infection” OR “Tunnel infection”):ti,ab,kw	2808
#6	(Peritoneum OR Perivisceritis OR Catheter OR “Percutaneous Access”):ti,ab,kw AND (Peritonitis OR Infection):ti,ab,kw	5197
#7	(Intraperiton OR Periton*):ti,ab,kw AND (Infection):ti,ab,kw	1932
#8	#5 OR #6 OR #7	7947
#9	#4 AND #8	472
#10	Limit to Trials	460
#11	limit #10 to articles published in year 2011 to 2026	169

Table S1 Systematic Review Search Strategy (Continued)

**Scopus (From Inception to May 30, 2026)**		
**Search**	**Query**	**Items Found**
#1	(TITLE-ABS-KEY (peritoneal AND dialysis) OR TITLE-ABS-KEY (continuous AND ambulatory AND peritoneal AND dialysis) OR TITLE-ABS-KEY (automated AND peritoneal AND dialysis) OR TITLE-ABS-KEY (continuous AND cycling AND peritoneal AND dialysis) OR TITLE-ABS-KEY (intermittent AND peritoneal AND dialysis) OR TITLE-ABS-KEY (nocturnal AND intermittent AND peritoneal AND dialysis) OR TITLE-ABS-KEY (tidal AND peritoneal AND dialysis))	56436
#2	(TITLE-ABS-KEY (catheter AND infection) OR TITLE-ABS-KEY (peritonitis) OR TITLE-ABS-KEY (exit AND site AND infection) OR TITLE-ABS-KEY (tunnel AND infection))	142261
#3	(TITLE-ABS-KEY (peritoneum AND infection) OR TITLE-ABS-KEY (infectious AND complication) OR TITLE-ABS-KEY (catheter AND infection) OR TITLE-ABS-KEY (catheter AND peritonitis) OR TITLE-ABS-KEY (catheter AND complication) OR TITLE-ABS-KEY (perivisceritis))	257406
#4	(TITLE-ABS-KEY (percutaneous AND access) AND TITLE-ABS-KEY (infection))	2080
#5	(TITLE-ABS-KEY (intraperitoneal AND infection) OR TITLE-ABS-KEY (peritoneal AND infection))	42099
#6	#2 OR #3 OR #4 OR #5	356732
#7	#1 AND #6	17649
#8	Filter: #7 AND (LIMIT-TO (DOCTYPE , "ar")) AND (LIMIT-TO (SRCTYPE , "j")) AND (LIMIT-TO (LANGUAGE , "English")) AND (EXCLUDE (EXACTKEYWORD , "Case Report") OR EXCLUDE (EXACTKEYWORD , "Nonhuman") OR EXCLUDE (EXACTKEYWORD , "Human Tissue") OR EXCLUDE (EXACTKEYWORD , "Human Cell") OR EXCLUDE (EXACTKEYWORD , "Animal Experiment") OR EXCLUDE (EXACTKEYWORD , "Animals")	7476
#9	limit #8 to articles published in year 2011 to 2026	3697

Table S1 Systematic Review Search Strategy (Continued)

**CINAHL (From Inception to May 30, 2026)**		
**Search**	**Query**	**Items Found**
#1	MW peritoneal dialysis	4692
#2	AB “peritoneal dialysis” OR AB “continuous ambulatory peritoneal dialysis” OR AB “automated peritoneal dialysis” OR AB “continuous cycling peritoneal dialysis” OR AB “intermittent peritoneal dialysis” OR AB “nocturnal intermittent peritoneal dialysis” OR AB “tidal peritoneal dialysis”	3326
#3	S1 OR S2	5731
#4	AB “catheter infection” OR peritonitis OR “exit site infection” OR “tunnel infection”	4714
#5	AB “peritoneum infection” OR “infectious complication” OR “catheter infection” OR “catheter peritonitis” OR “catheter complication” OR perivisceritis	507
#6	AB “percutaneous access” AND infection	24
#7	AB “intraperitoneal infection” OR “peritoneal infection”	52
#8	S4 OR S5 OR S6 OR S7	5121
#9	S3 AND S8	895
#10	Expanders - Apply equivalent subjects<br>Source Types - Academic Journals	824
#11	limit #10 to articles published in year 2011 to 2026	557

Table 2 The PICOTS Framework: Study Inclusion/Exclusion Criteria

Category	Criteria for inclusion	Criteria for exclusion
Populations	• ESKD patients undergoing chronic PD treatment regardless of age, sex, modality of PD, and comorbid conditions<br>• Subgroup analyses or companion studies that provided sufficient data to calculate the effect estimates of the outcome of interest also considered	• Use of PD for the treatment of acute kidney injury or fluid overload<br>• In vitro, in vivo, or animal studies<br>• Studies that included target population less than 100 patients<br>• Studies that focused on a particular comorbid condition or etiology of ESKD of PD patients other than age, race/ethnicity, and PD modalities
Interventions	• Not applicable	• Not applicable
Comparators	• Not applicable	• Not applicable
Outcomes	• Total incidence rate of PD-associated peritonitis according to the ISPD guideline recommendations<br>• Incidence rate of causative organism of PD-associated peritonitis according to the ISPD guideline recommendations	• Reported only specific causative organism of PD-related peritonitis<br>• Unclear definitions of PD-related infections based on the ISPD definition<br>• Non-infectious peritonitis (i.e., chemical or sclerosing encapsulating peritonitis)<br>• Follow-up time or study period less than 1 year<br>• Studies with unclear information or insufficient data for recalculation the effect estimates of the outcome of interest
Timing	• From 2011 bibliographic databases onward<br>• Studies was limited to English language publication	• Studies that were published in the timing frame before 2011<br>• Non-English language studies
Study design	• Full published articles based on retrospective, prospective, or ambispective data collection and registry data that provided event rates corresponding to their patient-time at risks, in any setting and context	• Results based on intervention studies for the prevention or treatment of PD-related infections (RCTs [including secondary analysis of RCTs], or quasi-experimental studies) pharmacokinetics and pharmacodynamic studies, qualitative studies, cross-sectional/case-control studies, N-of-one trials, case series/case reports, and unpublished abstracts (conference abstracts)<br>• Letters/brief reports without formal methods<br>• Reports not involving primary data, including narrative review, systematic review, meta-analysis, news items, consensus statement, guidelines, and opinion/editorials/news

Abbreviations: ESKD, end-stage kidney disease; ISPD, International Society for Peritoneal Dialysis; NA, not applicable; PD, peritoneal dialysis; RCTs, randomized controlled trials.

Table S3 Excluded Studies

List	Source	Year	Title	Journal	Reason for Exclusion
1	Alwakeel JS, et al	2011	Outcome and complications in peritoneal dialysis patients: a five-year single center experience.	Saudi Journal of Kidney Diseases & Transplantation.2011; 22 (2): 245-51.	Small sample size (less than 100 patients)
2	Barone RJ, et al	2011	The importance of the Patient's training in chronic peritoneal dialysis and peritonitis.	Advances in Peritoneal Dialysis.2011; 27 97-100.	Small sample size (less than 100 patients)
3	Burke M, et al	2011	Relapsing and recurrent peritoneal dialysis-associated peritonitis: a multicenter registry study.	American Journal of Kidney Diseases.2011; 58 (3): 429-436.	Duplicate population (ANZDATA) with Cho Y, et al (2012)
4	Chang TI, et al	2011	Early catheter removal improves patient survival in peritoneal dialysis patients with fungal peritonitis: results of ninety-four episodes of fungal peritonitis at a single center.	Peritoneal Dialysis International.2011; 31 (1): 60-6.	Specific organism (bacterial and fungal)
5	Chavada R, et al	2011	Seeking clarity within cloudy effluents: differentiating fungal from bacterial peritonitis in peritoneal dialysis patients.	PLoS ONE.2011; 6 (12): e28247.	Duplicate population with Cho Y, et al (2012) and Cho Y (2013)
6	Chuengsaman P, et al	2011	Does previous abdominal operation affect peritoneal dialysis complications and outcomes?	Journal of the Medical Association of Thailand.2011; 94 Suppl 4 S64-70.	Data insufficient to calculate effect estimate
7	Cnossen TT, et al	2011	Comparison of outcomes on continuous ambulatory peritoneal dialysis versus automated peritoneal dialysis: results from a USA database.	Peritoneal Dialysis International.2011; 31 (6): 679-84.	Unclear definition of PD-associated peritonitis and data insufficient to calculate effect estimate
8	Dong J, et al	2011	Daily protein intake and survival in patients on peritoneal dialysis.	Nephrology Dialysis Transplantation.2011; 26 (11): 3715-21.	Duplicate population with Xu R, et al (2012)
9	Elhassan E, et al	2011	Prolonged duration of peritoneal dialysis catheter embedment does not lower the catheter success rate.	Peritoneal Dialysis International.2011; 31 (5): 558-64.	No outcome reported (incidence data [no. of event and time-at-risk])
10	Ghali JR, et al	2011	Microbiology and outcomes of peritonitis in Australian peritoneal dialysis patients.	Peritoneal Dialysis International.2011; 31 (6): 651-62.	Duplicate population (ANZDATA) with Cho Y, et al (2012)
11	Gürsu M, et al	2011	The effect of culture technique on culture positivity in peritoneal dialysis patients with peritonitis.	Klimik Dergisi.2011; 24 (2): 94-97.	Non-English language (article in Turkish)
12	Huang ST, et al	2011	Outcome of peritoneal dialysis in cirrhotic patients with end-stage renal disease - a 24-years' experience in Taiwan.	Clinical Nephrology.2011; 76 (4): 306-313.	Duplicate population with Huang ST, et al (2011)
13	Jiang Z, et al	2011	Advancing the use and quality of peritoneal dialysis by developing a peritoneal dialysis satellite center program.	Peritoneal Dialysis International.2011; 31 (2): 121-126.	Unclear definition of PD-associated peritonitis and data insufficient to calculate effect estimate
14	Jurina H, et al	2011	Thirty years experience of treatment with peritoneal dialysis in university hospital "Sveti Duh".	Acta Medica Croatica.2011; 65 (SUPP 3): 41-44.	Non-English language (article in Croatian)
15	Kanokkantapong C, et al	2011	The effects of peritoneal dialysis catheter insertion using paramedian versus midline approach on CAPD patients.	Journal of the Medical Association of Thailand.2011; 94 Suppl 4 S52-7.	Unclear definition of PD-associated peritonitis and data insufficient to calculate effect estimate
16	Karkar A, et al	2011	Outcome of patients treated with automated peritoneal dialysis: effects of selection of patients.	Saudi Journal of Kidney Diseases & Transplantation.2011; 22 (1): 40-8.	Small sample size (less than 100 patients)
17	Khan FY, et al	2011	Fungal peritonitis in patients undergoing continuous ambulatory peritoneal dialysis in Qatar.	Journal of Infection in Developing Countries.2011; 5 (9): 646-51.	Data insufficient to calculate effect estimate
18	Khositrangsikun K, et al	2011	Comparison of the Seldinger technique and surgical technique in Tenckhoff catheter insertion in CAPD patients: a single center experience.	Journal of the Medical Association of Thailand.2011; 94 Suppl 4 S77-80.	Unclear definition of PD-associated peritonitis and data insufficient to calculate effect estimate
19	Koc Y, et al	2011	Clinical outcome of diabetic peritoneal dialysis patients and evaluation of factors affecting mortality: A single centre's experience from turkey.	Journal of Renal Care.2011; 37 (2): 94-100.	Small sample size (less than 100 patients)
20	Ladd AP, et al	2011	Impact of primary omentectomy on longevity of peritoneal dialysis catheters in children.	American Journal of Surgery.2011; 201 (3): 401-405.	Unclear definition of PD-associated peritonitis and data insufficient to calculate effect estimate

Table S3 Excluded Studies (Continued)

List	Source	Year	Title	Journal	Reason for Exclusion
21	Li L, et al	2011	Peritoneal Dialysis as the First-line Renal Replacement Therapy in Patients With Autosomal Dominant Polycystic Kidney Disease.	American Journal of Kidney Diseases.2011; 57 (6): 903-907.	Duplicate population with Szeto CC, et al (2022)
22	Lim WH, et al	2011	Remote indigenous peritoneal dialysis patients have higher risk of peritonitis, technique failure, all-cause and peritonitis-related mortality.	Nephrology Dialysis Transplantation.2011; 26 (10): 3366-72.	Data insufficient to calculate effect estimate
23	Lim WH, et al	2011	Compared with younger peritoneal dialysis patients, elderly patients have similar peritonitis-free survival and lower risk of technique failure, but higher risk of peritonitis-related mortality.	Peritoneal Dialysis International.2011; 31 (6): 663-71.	Data insufficient to calculate effect estimate
24	Lobbedez T, et al	2011	Peritoneal dialysis in polycystic kidney disease patients. Report from the French peritoneal dialysis registry (RDPLF).	Nephrology Dialysis Transplantation.2011; 26 (7): 2332-9.	Data insufficient to calculate effect estimate
25	Lobo J, et al	2011	Peritoneal dialysis in Argentina. A nationwide study.	Peritoneal Dialysis International.2011; 31 (1): 19-26.	Data insufficient to calculate effect estimate
26	Mehrotra R, et al	2011	Serum albumin as a predictor of mortality in peritoneal dialysis: comparisons with hemodialysis.	American Journal of Kidney Diseases.2011; 58 (3): 418-28.	Not relevant in the context of PD-associated peritonitis
27	Mizuno M, et al	2011	Peritonitis is still an important factor for withdrawal from peritoneal dialysis therapy in the Tokai area of Japan.	Clinical & Experimental Nephrology.2011; 15 (5): 727-737.	Duplicate population with Mizuno M, et al (2016)
28	Muraoka K, et al	2011	Early partial re-implantation of Tenckhoff catheters to treat intractable exit-site or tunnel infection.	Peritoneal Dialysis International.2011; 31 (3): 350-3.	Small sample size (less than 100 patients)
29	Najafi I, et al	2011	Epidemiology of culture-negative peritonitis in Iranian patients on continuous ambulatory peritoneal dialysis.	Iranian Journal of Kidney Diseases.2011; 5 (5): 332-337.	Duplicate population with Najafi (2014)
30	Pattanachaiwit N, et al	2011	The changing profile of PD-related peritonitis in Thailand: a single centers experience.	Journal of the Medical Association of Thailand.2011; 94 Suppl 4 S44-51.	Small sample size (less than 100 patients)
31	Portolés J, et al	2011	Peritoneal dialysis and kidney transplant. A two-way ticket in an integrated renal replacement therapy model.	Nefrologia.2011; 31 (4): 441-8.	Unclear definition of PD-associated peritonitis and data insufficient to calculate effect estimate
32	Rufino Hernández JM, et al	2011	Current peritoneal dialysis compared with haemodialysis: Medium-term survival analysis of incident dialysis patients in the canary Islands in recent years.	Nefrologia.2011; 31 (2): 174-184.	Not relevant in the context of PD-associated peritonitis
33	Sari F, et al	2011	The Evaluation of the Causes of Discontinuation in Patients Undergoing Continuous Ambulatory Peritoneal Dialysis.	Turkiye Klinikleri Journal of Medical Sciences.2011; 31 (4): 805-808.	Data insufficient to calculate effect estimate
34	Shigidi MM, et al	2011	Peritoneal dialysis, an expanding mode of renal replacement therapy in Qatar.	Saudi Journal of Kidney Diseases & Transplantation.2011; 22 (3): 587-93.	Unclear definition of PD-associated peritonitis and data insufficient to calculate effect estimate
35	Shu KH, et al	2011	Association of interleukin-1beta gene polymorphism and peritonitis in uremic patients undergoing peritoneal dialysis.	Blood Purification.2011; 32 (3): 156-60.	Small sample size (less than 100 patients)
36	Sirivongs D, et al	2011	Experiences on bedside Tenckhoff catheter implantation.	Journal of the Medical Association of Thailand.2011; 94 Suppl 4 S58-63.	Follow-up time/study period 1 year
37	Sun CY, et al	2011	In younger dialysis patients, automated peritoneal dialysis is associated with better long-term patient and technique survival than is continuous ambulatory peritoneal dialysis.	Peritoneal Dialysis International.2011; 31 (3): 301-307.	Data insufficient to calculate effect estimate
38	Szeto CC, et al	2011	Repeat peritonitis in peritoneal dialysis: retrospective review of 181 consecutive cases.	Clinical Journal of the American Society of Nephrology.2011; 6 (4): 827-833.	Specific population (repeat/relapsing episodes)
39	Szeto CC, et al	2011	Persistent symptomatic intra-abdominal collection after catheter removal for PD-related peritonitis.	Peritoneal Dialysis International.2011; 31 (1): 34-8.	Duplicate population with Li L (2011) and Kwan BC (2013)

Table S3 Excluded Studies (Continued)

List	Source	Year	Title	Journal	Reason for Exclusion
40	Toyohara T, et al	2011	Prognosis of patients on continuous ambulatory peritoneal dialysis (CAPD) for over 10 years.	Internal Medicine.2011; 50 (21): 2519-23.	Small sample size (less than 100 patients)
41	Ur-Rehman K, et al	2011	Peritoneal dialysis for chronic kidney disease patients: a single-center experience in Saudi Arabia.	Saudi Journal of Kidney Diseases & Transplantation.2011; 22 (3): 581-6.	Small sample size (less than 100 patients)
42	Yang X, et al	2011	Successfully managing a rapidly growing peritoneal dialysis program in Southern China.	Chin Med J (Engl).2011; 124 (17): 2696-700.	Duplicate population with Wu H, et al (2022)
43	Yang YF, et al	2011	Early initiation of continuous ambulatory peritoneal dialysis in patients undergoing surgical implantation of Tenckhoff catheters.	Peritoneal Dialysis International.2011; 31 (5): 551-7.	Follow-up time/study period 1 year
44	Yao KH, et al	2011	Particularities of tuberculosis in black Africans chronically dialysed.	Nephrologie et Therapeutique.2011; 7 (4): 242-244.	Non-English language (article in French)
45	Yavascan O, et al	2011	The comparison of exit-site care with normal saline and povidone-iodine in preventing exit-site infection and peritonitis in children on chronic peritoneal dialysis treatment.	Saudi Journal of Kidney Diseases & Transplantation.2011; 22 (5): 931-4.	Small sample size (less than 100 patients)
46	Yip T, et al	2011	Clinical course and outcomes of single-organism Enterococcus peritonitis in peritoneal dialysis patients.	Peritoneal Dialysis International.2011; 31 (5): 522-8.	Duplicate population with Yap DY (2012)
47	Zhou H, et al	2011	Survival advantage of normal weight in peritoneal dialysis patients.	Renal Failure.2011; 33 (10): 964-968.	Not relevant in the context of PD-associated peritonitis
48	Abdolmalehi EY, et al	2012	Although maintenance dialysis prevents death from uremia, patient survival and quality of life remains an important issue.	Journal of Mazandaran University of Medical Sciences.2012; 21 (86): 212-220.	Non-English language (article in Persian)
49	Antonia Gil G, et al	2012	Clinical effectiveness in peritoneal catheter exit orifice care.	Enfermería Nefrológica.2012; 15 (3): 194-201.	Non-English language (article in Spanish)
50	Barretti P, et al	2012	Peritoneal dialysis-related peritonitis due to Staphylococcus aureus: a single-center experience over 15 years.	PLoS ONE [Electronic Resource].2012; 7 (2): e31780.	Data insufficient to calculate effect estimate
51	Boudville N, et al	2012	Recent peritonitis associates with mortality among patients treated with peritoneal dialysis.	Journal of the American Society of Nephrology (JASN).2012; 23 (8): 1398-1405.	Data insufficient to calculate effect estimate
52	Chang EP, et al	2012	High serum DcR3 levels are associated with the occurrence of peritonitis in patients receiving chronic peritoneal dialysis.	Journal of the Chinese Medical Association: JCMA.2012; 75 (12): 644-8.	Small sample size (less than 100 patients)
53	Chen HC, et al	2012	Hemoglobin variability does not predict mortality in peritoneal dialysis patients.	Chang Gung Medical Journal.2012; 35 (1): 79-87.	Unclear definition of PD-associated peritonitis and data insufficient to calculate effect estimate
54	Cho KH, et al	2012	Catheter revision for the treatment of intractable exit site infection/tunnel infection in peritoneal dialysis patients: a single centre experience.	Nephrology.2012; 17 (8): 760-6.	Unclear definition of PD-associated peritonitis and data insufficient to calculate effect estimate
55	Cho Y, et al	2012	The effects of living distantly from peritoneal dialysis units on peritonitis risk, microbiology, treatment and outcomes: a multi-centre registry study.	BMC Nephrology.2012; 13 (1): 41-41.	Duplicate population with Cho Y, et al (2012)
56	Cyr-Mockler B, et al	2012	PARADISE almost found: realizing peritonitis analysis of rates advancing dialysis information and staff education.	CANNT Journal.2012; 22 (2): 19-19.	Opinion/news
57	Davenport A	2012	Do topical antibiotics reduce exit site infection rates and peritonitis episodes in peritoneal dialysis patients? The Pan Thames Renal Audit.	Journal of Nephrology.2012; 25 (5): 819-24.	Data insufficient to calculate effect estimate
58	Fernandez-Reyes MJ, et al	2012	The influence of initial peritoneal transport characteristics, inflammation, and high glucose exposure on prognosis for peritoneal membrane function.	Peritoneal Dialysis International.2012; 32 (6): 636-44.	Unclear definition of PD-associated peritonitis and data insufficient to calculate effect estimate

Table S3 Excluded Studies (Continued)

List	Source	Year	Title	Journal	Reason for Exclusion
59	Segura FC, et al	2012	Our experience with the self-locating catheter compared to other variants of the Tenckhoff catheter.	Enfermería Nefrológica.2012; 15 (4): 283-289.	Non-English language (article in Spanish)
60	Ghaffari A, et al	2012	Urgent-start peritoneal dialysis: a quality improvement report.	American Journal of Kidney Diseases.2012; 59 (3): 400-408.	Small sample size (less than 100 patients)
61	Han SH, et al	2012	Effects of icodextrin on patient survival and technique success in patients undergoing peritoneal dialysis.	Nephrol Dial Transplant.2012; 27 (5): 2044-50.	Unclear definition of PD-associated peritonitis and data insufficient to calculate effect estimate
62	Herget-Rosenthal S, et al	2012	Definition and risk factors of rapidly declining residual renal function in peritoneal dialysis: An observational study.	Kidney and Blood Pressure Research.2012; 35 (4): 233-241.	Small sample size (less than 100 patients)
63	Hiramatsu M, et al	2012	Application of peritoneal dialysis in elderly patients by classifying the age into young-old, old, and oldest-old.	Contrib Nephrol.2012; 177 48-56.	Unclear definition of PD-associated peritonitis and data insufficient to calculate effect estimate
64	Johnson DW, et al	2012	Weekend compared with weekday presentations of peritoneal dialysis-associated peritonitis.	Peritoneal Dialysis International.2012; 32 (5): 516-24.	Duplicate population with Cho Y, et al (2012)
65	Khan A, et al	2012	High rates of mortality and technique failure in peritoneal dialysis patients after critical illness.	Peritoneal Dialysis International.2012; 32 (1): 29-36.	Not relevant in the context of PD-associated peritonitis
66	Koc Y, et al	2012	Effects of AA amyloidosis on survival in peritoneal dialysis.	Kidney & Blood Pressure Research.2012; 36 (1): 182-90.	Unclear definition of PD-associated peritonitis and data insufficient to calculate effect estimate
67	Koc Y, et al	2012	Is there impact of mortality prior hemodialysis therapy in peritoneal dialysis patients?	Nefrologia.2012; 32 (3): 335-342.	Duplicate population with Koc Y, et al (2012)
68	Koch M, et al	2012	Peritoneal dialysis relieves clinical symptoms and is well tolerated in patients with refractory heart failure and chronic kidney disease.	European Journal of Heart Failure.2012; 14 (5): 530-539.	Follow-up time/study period 1 year
69	Kocyigit I, et al	2012	Improvement in culture-negative peritoneal dialysis-related peritonitis: a single center's experience.	Peritoneal Dialysis International.2012; 32 (4): 476-8.	Specific population (only peritonitis cases)
70	Lavergne V, et al	2012	Risk factors and consequences of hyperaluminemia in a peritoneal dialysis cohort.	Peritoneal Dialysis International.2012; 32 (6): 645-51.	Unclear definition of PD-associated peritonitis and data insufficient to calculate effect estimate
71	Lobbedez T, et al	2012	Is assisted peritoneal dialysis associated with technique survival when competing events are considered?	Clinical Journal of the American Society of Nephrology.2012; 7 (4): 612-618.	No outcome reported (incidence data [no. of event and time-at-risk])
72	Marinangeli G, et al	2012	Old and new perspectives on peritoneal dialysis in Italy emerging from the Peritoneal Dialysis Study Group Census.	Peritoneal Dialysis International.2012; 32 (5): 558-65.	Data insufficient to calculate effect estimate
73	Martinez-Mier G, et al	2012	Factors associated with early peritoneal dialysis catheter replacement in Veracruz, Mexico.	Nefrologia.2012; 32 (3): 353-8.	No outcome reported (incidence data [no. of event and time-at-risk])
74	Méndez Durán A, et al	2012	Seasonal behavior of peritoneal infections in patients under dialysis.	Dialisis y Trasplante.2012; 33 (1): 9-12.	Non-English language (article in Spanish)
75	Mou S, et al	2012	Change in cardiovascular disease status in peritoneal dialysis patients: A 5-year single-center experience.	Renal Failure.2012; 34 (1): 28-34.	Unclear definition of PD-associated peritonitis and data insufficient to calculate effect estimate
76	Nakamoto H	2012	How automated peritoneal dialysis is applied and maintained in Japan.	Contrib Nephrol.2012; 177 13-23.	Review article
77	Nessim SJ, et al	2012	Microbiology of peritonitis in peritoneal dialysis patients with multiple episodes.	Peritoneal Dialysis International.2012; 32 (3): 316-21.	Data insufficient to calculate effect estimate
78	Nobe K, et al	2012	Continuous ambulatory Peritoneal dialysis beyond a decade: cases from a single center.	Advances in Peritoneal Dialysis.2012; 28 74-8.	Not relevant in the context of PD-associated peritonitis
79	Okayama M, et al	2012	Aging is an important risk factor for peritoneal dialysis-associated peritonitis.	Advances in Peritoneal Dialysis.2012; 28 50-4.	Small sample size (less than 100 patients)

Table S3 Excluded Studies (Continued)

List	Source	Year	Title	Journal	Reason for Exclusion
80	Perl J, et al	2012	Changes in patient and technique survival over time among incident peritoneal dialysis patients in Canada.	Clin J Am Soc Nephrol.2012; 7 (7): 1145-54.	No outcome reported (incidence data [no. of event and time-at-risk])
81	Roszkowska-Blaim M, et al	2012	The effect of peritoneal dialysis method on residual renal function in children.	Advances in Peritoneal Dialysis.2012; 28 112-9.	Unclear definition of PD-associated peritonitis and data insufficient to calculate effect estimate
82	Sari F, et al	2012	Clinical outcomes of peritoneal dialysis patients at a secondary state hospital nephrology clinic.	Turkish Nephrology, Dialysis and Transplantation Journal.2012; 21 (1): 34-38.	Unclear definition of PD-associated peritonitis and data insufficient to calculate effect estimate
83	Schoenmaker NJ, et al	2012	Disparities in dialysis treatment and outcomes for Dutch and Belgian children with immigrant parents.	Pediatric Nephrology.2012; 27 (8): 1369-1379.	Unclear definition of PD-associated peritonitis and data insufficient to calculate effect estimate
84	Shu CC, et al	2012	Mycobacterial peritonitis: Difference between non-tuberculous mycobacteria and Mycobacterium tuberculosis.	Clinical Microbiology and Infection.2012; 18 (3): 246-252.	Not population of interest
85	Smyth A, et al	2012	Peritoneal dialysis in an ageing population: a 10-year experience.	International Urology & Nephrology.2012; 44 (1): 283-93.	Unclear definition of PD-associated peritonitis and data insufficient to calculate effect estimate
86	Taveras AE, et al	2012	Peritoneal dialysis in patients 75 years of age and older--a 22-year experience.	Advances in Peritoneal Dialysis.2012; 28 84-8.	Data insufficient to calculate effect estimate
87	Thirugnanasambathan T, et al	2012	Repeated peritoneal dialysis-associated peritonitis: a multicenter registry study.	American Journal of Kidney Diseases.2012; 59 (1): 84-91.	Duplicate population with Cho Y, et al (2012)
88	Tian S, et al	2012	Presence of peripheral arterial disease predicts loss of residual renal function in incident capd patients.	Peritoneal Dialysis International.2012; 32 (1): 67-72.	Small sample size (less than 100 patients)
89	Vidal E, et al	2012	Peritoneal dialysis in infants: the experience of the Italian Registry of Paediatric Chronic Dialysis.	Nephrology Dialysis Transplantation.2012; 27 (1): 388-95.	Small sample size (less than 100 patients)
90	Waness A, et al	2012	Tuberculous peritonitis associated with peritoneal dialysis.	Saudi Journal of Kidney Diseases & Transplantation.2012; 23 (1): 44-7.	Small sample size (less than 100 patients)
91	Wang IK, et al	2012	Bacteremia in hemodialysis and peritoneal dialysis patients.	Internal Medicine.2012; 51 (9): 1015-21.	Not relevant in the context of PD-associated peritonitis
92	Xu R, et al	2012	Impact of Individual and Environmental Socioeconomic Status on Peritoneal Dialysis Outcomes: A Retrospective Multicenter Cohort Study.	PLoS ONE.2012; 7 (11).	Specific organism (only first episodes) and data insufficient to calculate effect estimate
93	Yang CY, et al	2012	Essential trace element status and clinical outcomes in long-term dialysis patients: A two-year prospective observational cohort study.	Clinical Nutrition.2012; 31 (5): 630-636.	Small sample size (less than 100 patients)
94	Yang Z, et al	2012	Advanced nursing experience is beneficial for lowering the peritonitis rate in patients on peritoneal dialysis.	Peritoneal Dialysis International.2012; 32 (1): 60-6.	Duplicate population with Xu R, et al (2012)
95	Yoo DE, et al	2012	Good glycemic control is associated with better survival in diabetic patients on peritoneal dialysis: a prospective observational study.	PLoS ONE [Electronic Resource].2012; 7 (1): e30072.	No outcome reported (incidence data [no. of event and time-at-risk])
96	Agarwal G, et al	2013	Increase in peritonitis rates with the reuse of drain tubing of the Fresenius Liberty cycler.	Perit Dial Int.2013; 33 (5): 583-5.	Data insufficient to calculate effect estimate
97	Al-Hwiesh AK, et al	2013	Prevention of peritoneal dialysis catheter infections in saudi peritoneal dialysis patients: The emergence of high-level mupirocin resistance.	International Journal of Artificial Organs.2013; 36 (7): 473-483.	Invalid and data insufficient to calculate effect estimate
98	Briggs V, et al	2013	UK Renal Registry 15th annual report: Chapter 8 UK multisite peritoneal dialysis access catheter audit for first PD catheters 2011.	Nephron.2013; 123 Suppl 1 165-81.	Not relevant in the context of PD-associated peritonitis
99	Cheng CH, et al	2013	Clinical outcome of elderly peritoneal dialysis patients with assisted care in a single medical centre: a 25 year experience.	Nephrology.2013; 18 (6): 468-473.	Unclear definition of PD-associated peritonitis and data insufficient to calculate effect estimate

Table S3 Excluded Studies (Continued)

List	Source	Year	Title	Journal	Reason for Exclusion
100	Cho Y, et al	2013	Association of biocompatible peritoneal dialysis solutions with peritonitis risk, treatment, and outcomes.	Clinical Journal of the American Society of Nephrology.2013; 8 (9): 1556-1563.	Duplicate population with Cheikh Hassan HI, et al (2022)
101	Cho Y, et al	2013	Effects of climatic region on peritonitis risk, microbiology, treatment, and outcomes: a multicenter registry study.	Peritoneal Dialysis International.2013; 33 (1): 75-85.	Duplicate population with Cho Y, et al (2012)
102	Dong J, et al	2013	Peritoneal protein leakage, systemic inflammation, and peritonitis risk in patients on peritoneal dialysis.	Peritoneal Dialysis International.2013; 33 (3): 273-9.	Duplicate population with Xu R, et al (2012)
103	Dong J, et al	2013	Clinical characteristics and outcomes of "silent" and "non-silent" peritonitis in patients on peritoneal dialysis.	Peritoneal Dialysis International.2013; 33 (1): 28-37.	Data insufficient to calculate effect estimate
104	Fan SL, et al	2013	Clinical characteristics and outcomes of silent and non-silent peritonitis in patients on peritoneal dialysis.	Perit Dial Int.2013; 33 (4): 466-7.	Data insufficient to calculate effect estimate
105	Figueiredo AE, et al	2013	Peritonitis in patients on peritoneal dialysis: analysis of a single Brazilian center based on the International Society for Peritoneal Dialysis.	Jornal Brasileiro de Nefrologia.2013; 35 (3): 214-9.	Data insufficient to calculate effect estimate
106	Gray NA, et al	2013	Peritoneal dialysis in rural Australia.	BMC Nephrology.2013; 14 (1): 278-278.	Duplicate population with Cho Y, et al (2013)
107	Gultekin FA, et al	2013	Our long-term results of Tenckhoff peritoneal dialysis catheters placement via laparoscopic preperitoneal tunneling technique.	Semin Dial.2013; 26 (3): 349-54.	Small sample size (less than 100 patients)
108	Huang JW, et al	2013	Lean Body Mass Predicts Long-Term Survival in Chinese Patients on Peritoneal Dialysis.	PLoS ONE.2013; 8 (1).	Unclear definition of PD-associated peritonitis and data insufficient to calculate effect estimate
109	Hussain MI, et al	2013	Outcome of open Tenckhoff catheter insertions: 5 years experience in a major tertiary centre in central Saudi Arabia.	J Ayub Med Coll Abbottabad.2013; 25 (3-4): 12-5.	Cross-sectional studies
110	Jeloka T, et al	2013	'Ico-Alone' single nocturnal exchange to initiate peritoneal dialysis in patients with residual renal function--Five year, single centre experience.	Indian Journal of Nephrology.2013; 23 (4): 276-279.	Small sample size (less than 100 patients)
111	Jiang SH, et al	2013	Non-tuberculous mycobacterial PD peritonitis in Australia.	International Urology & Nephrology.2013; 45 (5): 1423-8.	Specific organism (NTM)
112	Katavetin P, et al	2013	Treatment failure in automated peritoneal dialysis and double-bag continuous ambulatory peritoneal dialysis.	Nephrology.2013; 18 (8): 545-548.	No outcome reported (incidence data [no. of event and time-at-risk])
113	Klaric D, et al	2013	Long-term effects of peritonitis on peritoneal dialysis outcomes.	International Urology & Nephrology.2013; 45 (2): 519-25.	Small sample size (less than 100 patients)
114	Kwan BC, et al	2013	Circulating bacterial-derived DNA fragments as a marker of systemic inflammation in peritoneal dialysis.	Nephrology Dialysis Transplantation.2013; 28 (8): 2139-45.	Duplicate population with Than WH, et al (2023)
115	Kwan BC, et al	2013	Automated peritoneal dialysis in Hong Kong: there are two distinct groups of patients.	Nephrology.2013; 18 (5): 356-64.	Duplicate population with Kwan BC, et al (2013)
116	Lee KO, et al	2013	Outcomes of peritonitis in children on peritoneal dialysis: a 25-year experience at Severance Hospital.	Yonsei Medical Journal.2013; 54 (4): 983-9.	Small sample size (less than 100 patients)
117	Lloyd A, et al	2013	The risk of peritonitis after an exit site infection: a time-matched, case-control study.	Nephrology Dialysis Transplantation.2013; 28 (7): 1915-21.	Duplicate population with Nessim SJ, et al (2013)
118	Lobbedez T, et al	2013	Outcome of the sub-optimal dialysis starter on peritoneal dialysis. Report from the French Language Peritoneal Dialysis Registry (RDPLF).	Nephrology Dialysis Transplantation.2013; 28 (5): 1276-1283.	Data insufficient to calculate effect estimate
119	Lopes K, et al	2013	Long-term peritoneal dialysis experience: quality control supports the use of fluconazole to prevent fungal peritonitis.	International Journal of Artificial Organs.2013; 36 (7): 484-8.	Duplicate population with Rocha A, et al (2012)

Table S3 Excluded Studies (Continued)

List	Source	Year	Title	Journal	Reason for Exclusion
120	Mitsnefes MM, et al	2013	Mortality risk among children initially treated with dialysis for end-stage kidney disease, 1990-2010.	JAMA.2013; 309 (18): 1921-1929.	No outcome reported (incidence data [no. of event and time-at-risk])
121	Nikibakhsh AA, et al	2013	Outcome of Immediate Use of the Permanent Peritoneal Dialysis Catheter in Children with Acute and Chronic Renal Failure.	Iranian Journal of Pediatrics.2013; 23 (2): 171-176.	Small sample size (less than 100 patients)
122	Phan J, et al	2013	Risk factors for morbidity and mortality in pediatric patients with peritoneal dialysis catheters.	Journal of Pediatric Surgery.2013; 48 (1): 197-202.	No outcome reported (incidence data [no. of event and time-at-risk])
123	Rodriguez-Garcia VH, et al	2013	Association between peritoneal protein excretion, peritonitis and D/P phosphate, in patients on peritoneal dialysis.	Nefrologia.2013; 33 (2): 204-13.	Small sample size (less than 100 patients)
124	Ros S, et al	2013	Increased risk of fatal infections in women starting peritoneal dialysis.	Peritoneal Dialysis International.2013; 33 (5): 487-94.	No outcome reported (incidence data [no. of event and time-at-risk])
125	Sayed SA, et al	2013	Effect of the patient's knowledge on peritonitis rates in peritoneal dialysis.	Peritoneal Dialysis International.2013; 33 (4): 362-6.	Small sample size (less than 100 patients)
126	Segura FC, et al	2013	Our experience with the self-locating catheter compared to other variants of the tenckhoff catheter. Multicentre study.	Enfermeria Nefrologica.2013; 15 (4): 283-289.	Duplicate with Seura FC, et al (2012)
127	Seziş Demirci M, et al	2013	The extracellular water corrected for height predicts technique survival in peritoneal dialysis patient.	Turkish Nephrology, Dialysis and Transplantation Journal.2013; 22 (2): 188-195.	Small sample size (less than 100 patients)
128	Sinangil A, et al	2013	Effects of infectious complications on patients' survival in peritoneal dialysis.	European Review for Medical & Pharmacological Sciences.2013; 17 (8): 1064-72.	Data insufficient to calculate effect estimate
129	Steenkamp R, et al	2013	UK renal registry 15th annual report: Chapter 5 survival and causes of death of UK adult patients on renal replacement therapy in 2011: National and centre-specific analyses.	Nephron - Clinical Practice.2013; 123 (SUPPL. 1): 93-123.	No outcome reported (incidence data [no. of event and time-at-risk])
130	Szeto CC, et al	2013	Bacteria-derived DNA fragment in peritoneal dialysis effluent as a predictor of relapsing peritonitis.	Clinical Journal of the American Society of Nephrology.2013; 8 (11): 1935-1941.	Specific population (only peritonitis cases)
131	Tan J, et al	2013	End stage renal disease in Brunei Darussalam-report from the first Brunei Dialysis Transplant Registry (BDTR).	Renal Failure.2013; 35 (8): 1101-1104.	Unclear definition of PD-associated peritonitis and data insufficient to calculate effect estimate
132	Teixeira L, et al	2013	Modelling competing risks in nephrology research: an example in peritoneal dialysis.	BMC Nephrology.2013; 14 (1): 110-110.	Data insufficient to calculate effect estimate
133	Tsai CC, et al	2013	Effects of age and diabetes mellitus on clinical outcomes in patients with peritoneal dialysis-related peritonitis.	Surgical Infections.2013; 14 (6): 540-546.	Duplicate with Tsai CC, et al (2013)
134	Unsal A, et al	2013	Clinical outcomes and mortality in peritoneal dialysis patients: a 10-year retrospective analysis in a single center.	Clinical Nephrology.2013; 80 (4): 270-9.	Unclear definition of PD-associated peritonitis and data insufficient to calculate effect estimate
135	Wu K, et al	2013	Comparison of gentamicin ointment to mupirocin ointment for prevention of peritoneal dialysis catheter-related infections.	Current Drug Therapy.2013; 8 (1): 38-44.	Small sample size (less than 100 patients)
136	Yeh CN, et al	2013	Dual-incision laparoscopic surgery for peritoneal dialysis catheter implantation and fixation: a novel, simple, and safe procedure.	Journal of Laparoendoscopic & Advanced Surgical Techniques. Part A.2013; 23 (8): 673-8.	Data insufficient to calculate effect estimate
137	Unal A, et al	2014	Does body mass index affect survival and technique failure in patients undergoing peritoneal dialysis?	Pak J Med Sci. 2014 Jan;30(1):41-4.	Unclear definition of PD-associated peritonitis and data insufficient to calculate effect estimate
138	Abebe M, et al	2014	Pet-related peritonitis and its prevention in peritoneal dialysis: a case study.	Peritoneal Dialysis International.2014; 34 (4): 466-8.	Small sample size (less than 100 patients)

Table S3 Excluded Studies (Continued)

List	Source	Year	Title	Journal	Reason for Exclusion
139	Achim C, et al	2014	Cardiovascular risk in esrd patients on the transplant waiting list for renal transplantation.	Journal of Translational Medicine and Research.2014; 19 (1-2): 30-36.	Small sample size (less than 100 patients)
140	Altunoglu A, et al	2014	Latex sensitization: A special risk for peritonitis in continuous peritoneal dialysis patients?	Journal of Clinical and Analytical Medicine.2014; 5 (2): 144-146.	Small sample size (less than 100 patients)
141	Ananthakrishnan S, et al	2014	Peritoneal dialysis outcomes in a modern cohort of overweight patients.	International Urology & Nephrology.2014; 46 (1): 183-9.	Small sample size (less than 100 patients)
142	Bechade C, et al	2014	Early failure in patients starting peritoneal dialysis: a competing risks approach.	Nephrology Dialysis Transplantation.2014; 29 (11): 2127-35.	Not relevant in the context of PD-associated peritonitis
143	Bekaoui S, et al	2014	Infectious peritonitis profile in peritoneal dialysis at Ibn Sina University hospital: A 6-year data report.	International Journal of Nephrology and Renovascular Disease.2014; 7 323-327.	Small sample size (less than 100 patients)
144	Briggs V, et al	2014	UK renal registry 16th annual report: Chapter 14 2012 multisite dialysis access audit in England, Northern Ireland and Wales and 2011 PD one year follow-up: National and centre-specific analyses.	Nephron - Clinical Practice.2014; 125 (1-4): 275-294.	Not relevant in the context of PD-associated peritonitis
145	Camargo CH, et al	2014	Peritoneal Dialysis-Related Peritonitis due to Coagulase-Negative Staphylococcus: A Review of 115 Cases in a Brazilian Center.	Clinical Journal of the American Society of Nephrology.2014; 9 (6): 1074-1081.	Specific organism (CoNS) and data insufficient to calculate effect estimate
146	Chang TI, et al	2014	Hyponatremia as a predictor of mortality in peritoneal dialysis patients.	PLoS ONE [Electronic Resource].2014; 9 (10): e111373.	No outcome reported (incidence data [no. of event and time-at-risk])
147	Cho Y, et al	2014	Peritoneal dialysis outcomes after temporary haemodialysis transfer for peritonitis.	Nephrology Dialysis Transplantation.2014; 29 (10): 1940-7.	Specific population (only peritonitis cases)
148	Cho Y, et al	2014	Outcomes of nephrologist-inserted peritoneal catheters in indigenous patients from Far North Queensland.	Peritoneal Dialysis International.2014; 34 (6): 663-7.	Small sample size (less than 100 patients)
149	Choi SJ, et al	2014	Does body fat mass define survival in patients starting peritoneal dialysis?	Peritoneal Dialysis International.2014; 34 (4): 376-82.	Unclear definition of PD-associated peritonitis and data insufficient to calculate effect estimate
150	Chula DC, et al	2014	Percutaneous and surgical insertion of peritoneal catheter in patients starting in chronic dialysis therapy: a comparative study.	Seminars in Dialysis.2014; 27 (3): E32-7.	Unclear definition of PD-associated peritonitis and data insufficient to calculate effect estimate
151	Cotovio P, et al	2014	Better outcomes of peritoneal dialysis in diabetic patients in spite of risk of loss of autonomy for home dialysis.	Peritoneal Dialysis International.2014; 34 (7): 775-780.	Unclear definition of PD-associated peritonitis and data insufficient to calculate effect estimate
152	Courivaud C, et al	2014	Peritoneal dialysis reduces the number of hospitalization days in heart failure patients refractory to diuretics.	Peritoneal Dialysis International.2014; 34 (1): 100-108.	No outcome reported (incidence data [no. of event and time-at-risk])
153	Crawford S, et al	2014	Can 2% chlorhexidine aqueous solution provide better infection control in PD exit site care when compared with 10% povidone-iodine: a single unit experience.	Renal Society of Australasia Journal.2014; 10 (3): 116-119.	No outcome reported (incidence data [no. of event and time-at-risk])
154	de Moraes TP, et al	2014	Characterization of the BRAZPD II cohort and description of trends in peritoneal dialysis outcome across time periods.	Peritoneal Dialysis International.2014; 34 (7): 714-23.	No outcome reported (incidence data [no. of event and time-at-risk])
155	de Moraes TP, et al	2014	Novel predictors of peritonitis-related outcomes in the BRAZPD cohort.	Peritoneal Dialysis International.2014; 34 (2): 179-87.	Data insufficient to calculate effect estimate
156	Fan X, et al	2014	Risk factors for the first episode of peritonitis in Southern Chinese continuous ambulatory peritoneal dialysis patients.	PLoS ONE [Electronic Resource].2014; 9 (9): e107485.	Duplicate population with Wu H, et al (2022)
157	Feng X, et al	2014	Escherichia coli Peritonitis in peritoneal dialysis: the prevalence, antibiotic resistance and clinical outcomes in a South China dialysis center.	Peritoneal Dialysis International.2014; 34 (3): 308-16.	Duplicate with population with Fan X, et al (2014)

Table S3 Excluded Studies (Continued)

List	Source	Year	Title	Journal	Reason for Exclusion
158	Hsieh YP, et al	2014	Predictors for and impact of high peritonitis rate in Taiwanese continuous ambulatory peritoneal dialysis patients.	International Urology and Nephrology.2014.	Duplicate with Hsieh YP, et al (2015)
159	Hsieh YP, et al	2014	Predictors of peritonitis and the impact of peritonitis on clinical outcomes of continuous ambulatory peritoneal dialysis patients in Taiwan--10 years' experience in a single center.	Peritoneal Dialysis International.2014; 34 (1): 85-94.	Duplicate with population with Hsieh YP, et al (2014)
160	Huang B, et al	2014	Diminished appetite predicts mortality of Chinese peritoneal dialysis patients.	Biol Res Nurs.2014; 16 (3): 241-9.	Small sample size (less than 100 patients)
161	Huang WH, et al	2014	Impact of environmental particulate matter and peritoneal dialysis-related infection in patients undergoing peritoneal dialysis.	Medicine.2014; 93 (25): e149.	Unclear definition of PD-associated peritonitis and data insufficient to calculate effect estimate
162	Jung HY, et al	2014	Free thyroxine level as an independent predictor of infection-related mortality in patients on peritoneal dialysis: a prospective multicenter cohort study.	PLoS ONE [Electronic Resource].2014; 9 (12): e112760.	No outcome reported (incidence data [no. of event and time-at-risk])
163	Kim Y, et al	2014	Use of a new connector decreases peritoneal dialysis-related peritonitis.	Peritoneal Dialysis International.2014; 34 (1): 128-30.	Small sample size (less than 100 patients)
164	Kumar VA, et al	2014	Predictors of peritonitis, hospital days, and technique survival for peritoneal dialysis patients in a managed care setting.	Peritoneal Dialysis International.2014; 34 (2): 171-8.	Unclear definition of PD-associated peritonitis and data insufficient to calculate effect estimate
165	Kwon JE, et al	2014	Effect of gastric acid suppressants and prokinetics on peritoneal dialysis-related peritonitis.	World Journal of Gastroenterology.2014; 20 (25): 8187-94.	Data insufficient to calculate effect estimate
166	Lan PG, et al	2014	The Association between Peritoneal Dialysis Modality and Peritonitis.	Clinical Journal of the American Society of Nephrology.2014; 9 (6): 1091-1097.	Data insufficient to calculate effect estimate
167	Lee SM, et al	2014	Comparison of exit site infection and peritonitis incidences between povidone-iodine and normal saline use for chronic exit site care in peritoneal dialysis patients.	Kidney Research and Clinical Practice.2014; 33 (3): 144-149.	Duplicate population with Lee SM, et al (2017)
168	Lin JH, et al	2014	Non-tuberculous and tuberculous mycobacterial peritonitis in peritoneal dialysis patients.	Renal Failure.2014; 36 (7): 1158-61.	Specific organism (NTM and tuberculous mycobacterial)
169	Liu Y, et al	2014	Impact of break-in period on the short-term outcomes of patients started on peritoneal dialysis.	Peritoneal Dialysis International.2014; 34 (1): 49-56.	Follow-up time/study period 1 year
170	Marley JV, et al	2014	Peritoneal dialysis outcomes of Indigenous Australian patients of remote Kimberley origin.	Australian Journal of Rural Health.2014; 22 (3): 101-108.	Data insufficient to calculate effect estimate
171	Martino F, et al	2014	Home visit program improves technique survival in peritoneal dialysis.	Blood Purification.2014; 37 (4): 286-290.	Data insufficient to calculate effect estimate
172	Mihalache O, et al	2014	Diagnosis characteristics and therapeutical options of infectious complications associated with peritoneal dialysis.	Journal of Medicine & Life. 7 Spec No.2014; 3 103-6.	Small sample size (less than 100 patients)
173	Niang A, et al	2014	Pilot experience in senegal with peritoneal dialysis for end-stage renal disease.	Peritoneal Dialysis International.2014; 34 (5): 539-43.	Small sample size (less than 100 patients)
174	Okamoto T, et al	2014	Free air on CT and the risk of peritonitis in peritoneal dialysis patients: a retrospective study.	Renal Failure.2014; 36 (10): 1492-6.	Data insufficient to calculate effect estimate
175	Ozener C, et al	2014	The impact of diabetes mellitus on peritoneal dialysis: the Turkey Multicenter Clinic Study.	Renal Failure.2014; 36 (2): 149-53.	Unclear definition of PD-associated peritonitis and data insufficient to calculate effect estimate
176	Pajek J, et al	2014	Outcomes of peritoneal dialysis patients and switching to hemodialysis: a competing risks analysis.	Perit Dial Int.2014; 34 (3): 289-98.	Not relevant in the context of PD-associated peritonitis

Table S3 Excluded Studies (Continued)

List	Source	Year	Title	Journal	Reason for Exclusion
177	Panorchan K, et al	2014	Diagnostic and prognostic role of peritoneal CA 125 in peritoneal dialysis patients presenting with acute peritonitis.	BMC Nephrology.2014; 15 (1): 149-149.	Not relevant in the context of PD-associated peritonitis
178	Pérez Fontán M, et al	2014	Compared decline of residual kidney function in patients treated with automated peritoneal dialysis and continuous ambulatory peritoneal dialysis: A multicenter study.	Nephron - Clinical Practice.2014; 128 352-360.	No outcome reported (incidence data [no. of event and time-at-risk])
179	Prasad N, et al	2014	Effect of body mass index on outcomes of peritoneal dialysis patients in India.	Peritoneal Dialysis International.2014; 34 (4): 399-408.	Duplicate population with Prasad KN, et al (2014)
180	Pulliam J, et al	2014	First-year outcomes of incident peritoneal dialysis patients in the United States.	American Journal of Kidney Diseases.2014; 64 (5): 761-769.	Specific organism (only first episodes)
181	Ram R, et al	2014	Risk factors that determine removal of the catheter in bacterial peritonitis in peritoneal dialysis.	Peritoneal Dialysis International.2014; 34 (2): 239-43.	Specific population (only peritonitis cases)
182	Remón-Rodríguez C, et al	2014	Results of the cooperative study of Spanish peritoneal dialysis registries: Analysis of 12 years of follow-up.	Nefrologia.2014; 34 (1): 18-33.	Data insufficient to calculate effect estimate
183	Restrepo CA, et al	2014	Implantation of peritoneal catheters by laparotomy: Nephrologists obtained similar results to general surgeons.	International Journal of Nephrology and Renovascular Disease.2014; 7 383-390.	Not relevant in the context of PD-associated peritonitis
184	Rodnguez-Carmona A, et al	2014	Correlation between glycemic control and the incidence of peritoneal and catheter tunnel and exit-site infections in diabetic patients undergoing peritoneal dialysis.	Peritoneal Dialysis International.2014; 34 (6): 618-626.	Specific population (diabetes patients)
185	Tamayo Isla RA, et al	2014	Continuous ambulatory peritoneal dialysis in Limpopo province, South Africa: Predictors of patient and technique survival.	Peritoneal Dialysis International.2014; 34 (5): 518-525.	Duplicate with Isla RA, et al (2014)
186	Tseng M-H, et al	2014	Hyponatremia is a surrogate marker of poor outcome in peritoneal dialysis-related peritonitis.	BMC Nephrology.2014; 15 (1): 113-113.	Specific population (only peritonitis cases)
187	Turkmen K, et al	2014	Health-related quality of life, depression and mortality in peritoneal dialysis patients in Turkey: seven-year experience of a center.	Renal Failure.2014; 36 (6): 859-64.	No outcome reported (incidence data [no. of event and time-at-risk])
188	van Esch S, et al	2014	32 years' experience of peritoneal dialysis-related peritonitis in a university hospital.	Peritoneal Dialysis International.2014; 34 (2): 162-70.	Data insufficient to calculate effect estimate
189	Vikrant S, et al	2014	Long-term clinical outcomes of peritoneal dialysis patients: 9-year experience of a single center from North India.	Peritoneal Dialysis International.2014; 34 (4): 426-433.	Small sample size (less than 100 patients)
190	Whittle A, et al	2014	Improving outcomes in peritoneal dialysis exit site care.	Renal Society of Australasia Journal.2014; 10 (3): 126-132.	Unclear definition of PD-associated peritonitis and data insufficient to calculate effect estimate
191	Yang ZK, et al	2014	The associations between the family education and mortality of patients on peritoneal dialysis.	PLoS ONE.2014; 9 (5).	Data insufficient to calculate effect estimate
192	Yu Y, et al	2014	Impact of continuous quality improvement initiatives on clinical outcomes in peritoneal dialysis.	Peritoneal Dialysis International.2014; 34 Suppl 2 S43-8.	Unclear definition of PD-associated peritonitis and data insufficient to calculate effect estimate
193	Zhang Q, et al	2014	Causes of death in peritoneal dialysis patients with different kidney diseases and comorbidities: a retrospective clinical analysis in a Chinese center.	International Urology & Nephrology.2014; 46 (6): 1201-7.	No outcome reported (incidence data [no. of event and time-at-risk])
194	Zhang X, et al	2014	The role of an integrated care model for kidney disease in the development of peritoneal dialysis: a single-center experience in China.	Perit Dial Int.2014; 34 Suppl 2 (Suppl 2): S55-8.	Data insufficient to calculate effect estimate
195	Abrahams AC, et al	2015	Improved outcome of enteric peritonitis in peritoneal dialysis (PD) patients with temporary discontinuation of PD and intravenous meropenem.	Nephrology dialysis transplantation.2015; 30 iii272.	Unpublished abstract (only abstract available)

Table S3 Excluded Studies (Continued)

List	Source	Year	Title	Journal	Reason for Exclusion
196	Al Agha R, et al	2015	Peritoneal dialysis: Trends, outcomes and complications.	Bahrain Medical Bulletin.2015; 37 (2): 121-123.	Unclear definition of PD-associated peritonitis and data insufficient to calculate effect estimate
197	Beduschi Gde C, et al	2015	Automated Peritoneal Dialysis Is Associated with Better Survival Rates Compared to Continuous Ambulatory Peritoneal Dialysis: A Propensity Score Matching Analysis.	PLoS One.2015; 10 (7): e0134047.	Specific organism (only first episodes)
198	Burkhalter F, et al	2015	Pseudomonas exit-site infection: Treatment outcomes with topical gentamicin in addition to systemic antibiotics.	Clinical Kidney Journal.2015; 8 (6): 781-784.	No outcome reported (incidence data [no. of event and time-at-risk])
199	Chao CT, et al	2015	Viridans streptococci in peritoneal dialysis peritonitis: clinical courses and long-term outcomes.	Peritoneal Dialysis International.2015; 35 (3): 333-41.	Specific organism (Gram-positive cocci)
200	Choy ASM, et al	2015	Weight change during the first year of peritoneal dialysis: Risk factors and prognostic implications.	Hong Kong Journal of Nephrology.2015; 17 (2): 28-35.	Unclear definition of PD-associated peritonitis and data insufficient to calculate effect estimate
201	Emre S, et al	2015	Chronic peritoneal dialysis in children: single center experience over 18 years.	Nephrology dialysis transplantation.2015; 30 iii540.	Unpublished abstract (only abstract available)
202	Erken E, et al	2015	The effect of serum mannose-binding lectin levels on dialysis-related peritonitis and catheter-related bacteremia.	Turkish Nephrology, Dialysis and Transplantation Journal.2015; 24 (2): 189-194.	Small sample size (less than 100 patients)
203	Guan JC, et al	2015	Influence of peritoneal transport characteristics on nutritional status and clinical outcome in Chinese diabetic nephropathy patients on peritoneal dialysis.	Chinese Medical Journal.2015; 128 (7): 859-864.	No outcome reported (incidence data [no. of event and time-at-risk])
204	Guo Q, et al	2015	The effect of fluid overload on clinical outcome in southern chinese patients undergoing continuous ambulatory peritoneal dialysis.	Peritoneal Dialysis International.2015; 35 (7): 691-702.	Duplicate with population with Fan X, et al (2014)
205	Hsieh YP, et al	2015	Predictors for and impact of high peritonitis rate in Taiwanese continuous ambulatory peritoneal dialysis patients.	International Urology & Nephrology.2015; 47 (1): 183-9.	Duplicate with population with Hsieh YP, et al (2014)
206	Janeiro D, et al	2015	Peritoneal Dialysis Can Be an Option for Dominant Polycystic Kidney Disease: an Observational Study.	Peritoneal Dialysis International.2015; 35 (5): 530-6.	Unclear definition of PD-associated peritonitis and data insufficient to calculate effect estimate
207	Jiang N, et al	2015	High Peritoneal Glucose Exposure Is Associated with Increased Incidence of Relapsing and Recurrent Bacterial Peritonitis in Patients Undergoing Peritoneal Dialysis.	Blood Purification.2015; 40 (1): 72-8.	Data insufficient to calculate effect estimate
208	Jovanovic DB, et al	2015	Inflammatory markers as mortality predictors in continuous ambulatory peritoneal dialysis patients.	Ren Fail.2015; 37 (2): 230-6.	Small sample size (less than 100 patients)
209	Ju-Yeh Y, et al	2015	Outcome Comparisons Between Patients on Peritoneal Dialysis With and Without Polycystic Kidney Disease: A Nationwide Matched Cohort Study.	Medicine.2015; 94 (48): 1-10.	Duplicate with Yang JY, et al (2015)
210	Kim H, et al	2015	Elderly Peritoneal Dialysis Compared with Elderly Hemodialysis Patients and Younger Peritoneal Dialysis Patients: Competing Risk Analysis of a Korean Prospective Cohort Study.	PLoS One.2015; 10 (6): e0131393.	Data insufficient to calculate effect estimate
211	Kim JE, et al	2015	Noninfectious complications of peritoneal dialysis in Korean children: A 26-year single-center study.	Yonsei Medical Journal.2015; 56 (5): 1359-1364.	Small sample size (less than 100 patients)
212	Kim JK, et al	2015	Excessive weight gain during the first year of peritoneal dialysis is associated with inflammation, diabetes mellitus, and a rapid decrease in residual renal function.	PLoS ONE.2015; 10 (9).	Data insufficient to calculate effect estimate
213	Kitterer D, et al	2015	Microbiological Surveillance of Peritoneal Dialysis Associated Peritonitis: Antimicrobial Susceptibility Profiles of a Referral Center in GERMANY over 32 Years.	PLoS ONE [Electronic Resource].2015; 10 (9): e0135969.	Specific population (only peritonitis cases)

Table S3 Excluded Studies (Continued)

List	Source	Year	Title	Journal	Reason for Exclusion
214	Koo HM, et al	2015	Changes in echocardiographic parameters according to the rate of residual renal function decline in incident peritoneal dialysis patients.	Medicine.2015; 94 (7): e427-e427.	Small sample size (less than 100 patients)
215	Laurin LP, et al	2015	Outcomes of Infection-Related Hospitalization according to Dialysis Modality.	Clinical Journal of The American Society of Nephrology: CJASN.2015; 10 (5): 817-24.	Not relevant in the context of PD-associated peritonitis
216	Lin J-H, et al	2015	Environmental NO2 level is associated with 2-year mortality in patients undergoing peritoneal dialysis.	Medicine.2015; 94 (1): e368-e368.	No outcome reported (incidence data [no. of event and time-at-risk])
217	Lin WH, et al	2015	Clinical and microbiological characteristics of peritoneal dialysis-related peritonitis caused by Klebsiella pneumoniae in southern Taiwan.	Journal of Microbiology, Immunology & Infection.2015; 48 (3): 276-83.	Specific population (only peritonitis cases)
218	Lu YH, et al	2015	Comparison of the impact of "fast decline" in residual renal function and "initial anuria" on long-term outcomes in CAPD patients.	Peritoneal Dialysis International.2015; 35 (2): 172-179.	No outcome reported (incidence data [no. of event and time-at-risk])
219	Maruyama Y, et al	2015	The Different Association between Serum Ferritin and Mortality in Hemodialysis and Peritoneal Dialysis Patients Using Japanese Nationwide Dialysis Registry.	PLoS ONE [Electronic Resource].2015; 10 (11): e0143430.	Unclear definition of PD-associated peritonitis and data insufficient to calculate effect estimate
220	McCartan D, et al	2015	Tenckhoff Peritoneal Dialysis Catheter Insertion in a Northern Ireland District General Hospital.	Ulster Medical Journal.2015; 84 (3): 166-70.	Small sample size (less than 100 patients)
221	McGuire AL, et al	2015	Effects of a Statewide Protocol for the Management of Peritoneal Dialysis-Related Peritonitis on Microbial Profiles and Antimicrobial Susceptibilities: A Retrospective Five-Year Review.	Peritoneal Dialysis International.2015; 35 (7): 722-8.	Data insufficient to calculate effect estimate
222	Medani S, et al	2015	Comparison of Percutaneous and Open Surgical Techniques for First-Time Peritoneal Dialysis Catheter Placement in the Unbreached Peritoneum.	Peritoneal Dialysis International.2015; 35 (5): 576-85.	Data insufficient to calculate effect estimate
223	Méndez-Durán A, et al	2015	Peritoneal infection. Comparative study of three providers.	Gaceta Medica de Bilbao.2015; 112 (3): 154-159.	Non-English language (article in Spanish)
224	Núñez Moral M, et al	2015	Utilidad de las tiras reactivas multistix 10 SG® en el diagnóstico de peritonitis en diálisis peritoneal.	Enfermería Nefrológica.2015; 18 (2): 124-129.	Non-English language (article in Spanish)
225	Proenca de Moraes T, et al	2015	Systemic lupus erythematous and clinical outcomes in peritoneal dialysis.	Lupus.2015; 24 (3): 290-8.	Unclear definition of PD-associated peritonitis and data insufficient to calculate effect estimate
226	Querido S, et al	2015	Results in Assisted Peritoneal Dialysis: A Ten-Year Experience.	International Journal of Nephrology.2015; 1-5.	Unclear definition of PD-associated peritonitis and data insufficient to calculate effect estimate
227	Ribeiro SC, et al	2015	Low Serum Potassium Levels Increase the Infectious-Caused Mortality in Peritoneal Dialysis Patients: A Propensity-Matched Score Study.	PLoS ONE [Electronic Resource].2015; 10 (6): e0127453.	No outcome reported (incidence data [no. of event and time-at-risk])
228	Sakaci T, et al	2015	Clinical outcomes and mortality in elderly peritoneal dialysis patients.	Clinics (Sao Paulo, Brazil).2015; 70 (5): 363-8.	Unclear definition of PD-associated peritonitis and data insufficient to calculate effect estimate
229	Sakaci T, et al	2015	Effect of initial PET status on clinical course in peritoneal dialysis patients.	BANTAO Journal.2015; 13 (1): 14-19.	Unclear definition of PD-associated peritonitis and data insufficient to calculate effect estimate
230	Singh K, et al	2015	Toll-like receptors TLR4 (Asp299Gly and Thr399Ile) and TLR2 (Arg677Trp and Arg753Gln) gene polymorphisms in end-stage renal disease patients on peritoneal dialysis.	International Urology & Nephrology.2015; 47 (12): 2031-7.	Not relevant in the context of PD-associated peritonitis
231	Sivanandam S, et al	2015	Bedside percutaneous tenckhoff peritoneal dialysis catheter placement by nephrologists: a single centre experience.	Nephrology dialysis transplantation.2015; 30 iii552.	Unpublished abstract (only abstract available)
232	Szeto CC, et al	2015	Predictors of residual renal function decline in patients undergoing continuous ambulatory peritoneal dialysis.	Perit Dial Int.2015; 35 (2): 180-8.	Duplicate population with Than WH, et al (2023)

Table S3 Excluded Studies (Continued)

List	Source	Year	Title	Journal	Reason for Exclusion
233	Tang W, et al	2015	Socio-Economic Status and Peritonitis in Australian Non-Indigenous Peritoneal Dialysis Patients.	Peritoneal Dialysis International.2015; 35 (4): 450-9.	Duplicate population with Cheikh Hassan HI, et al (2022)
234	Theodoridis M, et al	2015	The influence of early peritonitis appearance on technique failure for peritoneal dialysis patients.	Nephrology dialysis transplantation.2015; 30 iii266-iii267.	Unpublished abstract (only abstract available)
235	Thokhonelidze I, et al	2015	Single center experience in successful prevention of exit-site infection in patients on peritoneal dialysis.	Georgian Medical News.2015; (241): 54-8.	Intervention trials or experimental studies
236	van Diepen AT, et al	2015	The first peritonitis episode alters the natural course of peritoneal membrane characteristics in peritoneal dialysis patients.	Peritoneal Dialysis International.2015; 35 (3): 324-32.	Data insufficient to calculate effect estimate
237	Vélez-Segovia E, et al	2015	Housing conditions and development of first peritonitis in patients who initiated peritoneal dialysis during the 2002-2011 period at a hospital in Lima.	Dialisis y Trasplante.2015; 36 (2): 64-71.	Non-English language (article in Spanish)
238	Vincenzo LM, et al	2015	A simple modelto predict encapsulating peritoneal sclerosis in patients undergoing peritoneal dialysis: a 20 years prospective controlled longitudinal cohort study of peritoneal membrane function.	Nephrology dialysis transplantation.2015; 30 iii258.	Unpublished abstract (only abstract available)
239	Virzi GM, et al	2015	The Role of Cell-Free Plasma DNA in Peritoneal Dialysis Patients with Peritonitis.	Peritoneal Dialysis International.2015; 35 (7): 755-8.	Not relevant in the context of PD-associated peritonitis
240	Wang Z, et al	2015	Early peritonitis is an independent risk factor for mortality in elderly peritoneal dialysis patients.	Kidney & Blood Pressure Research.2015; 40 (3): 298-305.	Duplicate population with Zeng Y, et al (2020)
241	Williams L, et al	2015	A clinical audit to compare peritoniis rates between peritoneal dialysis delivery systems.	Renal Society of Australasia Journal.2015; 11 (2): 68-72.	Small sample size (less than 100 patients)
242	Workeneh B, et al	2015	Causes for Withdrawal in an Urban Peritoneal Dialysis Program.	International Journal of Nephrology.2015; 2015 1-4.	Unclear definition of PD-associated peritonitis and data insufficient to calculate effect estimate
243	Yang JY, et al	2015	Outcome Comparisons Between Patients on Peritoneal Dialysis With and Without Polycystic Kidney Disease: A Nationwide Matched Cohort Study.	Medicine.2015; 94 (48): e2166.	Unclear definition of PD-associated peritonitis (based on ICD codes) and included only first episode of peritonitis
244	Ye H, et al	2015	Higher alkaline phosphatase was associated with the short-term adverse outcomes of peritoneal dialysis-related peritonitis.	Clin Chem Lab Med.2015; 53 (4): e113-6.	Data insufficient to calculate effect estimate
245	Zabaneh R, et al	2015	Peginesatide to Manage Anemia in Chronic Kidney Disease Patients on Peritoneal Dialysis.	Perit Dial Int.2015; 35 (4): 481-9.	Intervention trials or experimental studies
246	Zhang L, et al	2015	Correlation of malnutrition, inflammation, and atherosclerosis and prognosis of patients undergoing peritoneal dialysis.	Journal of Shanghai Jiaotong University (Medical Science).2015; 35 (3): 358-363and379.	Non-English language (article in Chinese)
247	Zhao M, et al	2015	Pathogenic bacteria and drug resistance in 132 cases of peritoneal dialysis related peritonitis.	Chinese Journal of Infection and Chemotherapy.2015; 15 (3): 236-243.	Non-English language (article in Chinese)
248	Alyousef AM, et al	2016	Peritoneal dialysis and peritonitis rate: Kuwait, four years' experience.	Saudi Journal of Kidney Diseases & Transplantation.2016; 27 (4): 762-8.	Small sample size (less than 100 patients) and data insufficient to calculate effect estimate
249	Benabed A, et al	2016	Effect of assistance on peritonitis risk in diabetic patients treated by peritoneal dialysis: report from the French Language Peritoneal Dialysis Registry.	Nephrology Dialysis Transplantation.2016; 31 (4): 656-62.	Specific population (diabetes patients)
250	Campbell DJ, et al	2016	Assessment of current practice and barriers to antimicrobial prophylaxis in peritoneal dialysis patients.	Nephrology Dialysis Transplantation.2016; 31 (4): 619-27.	Data insufficient to calculate effect estimate
251	Carranza-Torres JM, et al	2016	Effectiveness of the scheme reimpregnation maintenance schedule vs. ceftazidime/cephalothin in dialysis patients with peritonitis.	Nefrologia.2016; 36 (4): 448-50.	Follow-up time/study period 1 year

Table S3 Excluded Studies (Continued)

List	Source	Year	Title	Journal	Reason for Exclusion
252	Chen QJ, et al	2016	Correlation between changes in cancer antibody 125 level in dialysate and prognosis in peritoneal dialysis patients.	Journal of Shanghai Jiaotong University (Medical Science).2016; 36 (3): 393-398.	Non-English language (article in Chinese)
253	Chen SS, et al	2016	Long-Term Exit-Site Gentamicin Prophylaxis and Gentamicin Resistance in a Peritoneal Dialysis Program.	Peritoneal Dialysis International.2016; 36 (4): 387-9.	Data insufficient to calculate effect estimate
254	Cho Y, et al	2016	Higher dialysate matrix metalloproteinase-2 levels are associated with peritoneal membrane dysfunction.	Peritoneal Dialysis International.2016; 36 (1): 16-25.	Intervention trial (secondary analysis of randomized trial)
255	Dalgaard LS, et al	2016	Risk and prognosis of bacteremia and fungemia among peritoneal dialysis patients: A population-based cohort study.	Peritoneal Dialysis International.2016; 36 (6): 647-654.	Not relevant in the context of PD-associated peritonitis
256	Eriguchi M, et al	2016	Extended Swan-Neck Catheter With Upper Abdominal Exit-Site Reduces Peritoneal Dialysis-Related Infections.	Ther Apher Dial. 2016 Apr;20(2):158-64.	Unclear definition of PD-associated peritonitis and data insufficient to calculate effect estimate
257	Feng S, et al	2016	Impact of early-onset peritonitis on mortality and technique survival in peritoneal dialysis patients.	SpringerPlus.2016; 5 (1).	Duplicate population with Zeng Y, et al (2020)
258	George N, et al	2016	Comparison of early mechanical and infective complications in first time blind, bedside, midline percutaneous tenckhoff catheter insertion with ultra-short break-in period in diabetics and non-diabetics: Setting new standards.	Peritoneal Dialysis International.2016; 36 (6): 655-661.	Data insufficient to calculate effect estimate
259	Giacobino J, et al	2016	Fungal peritonitis in patients undergoing peritoneal dialysis (PD) in Brazil: molecular identification, biofilm production and antifungal susceptibility of the agents.	Medical Mycology.2016; 54 (7): 725-732.	Specific organism (fungal)
260	Guan JC, et al	2016	Clinical Characteristics and Risk Factors of Enterogenous Peritoneal Dialysis-related Peritonitis.	Chinese General Practice.2016; 19 (15): 1781-1785.	Non-English language (article in Chinese)
261	Hwang SD, et al	2016	Serum Alkaline Phosphatase Levels Predict Infection-Related Mortality and Hospitalization in Peritoneal Dialysis Patients.	PLoS ONE [Electronic Resource].2016; 11 (6): e0157361.	Not relevant in the context of PD-associated peritonitis
262	Jellouli M, et al	2016	Peritoneal dialysis: Experience of the department of pediatrics of the hospital Charles Nicolle of Tunis.	Tunisie Medicale.2016; 94 (5): 368-374.	Systematic review
263	Jin H, et al	2016	Urgent-start peritoneal dialysis and hemodialysis in esrd patients: Complications and outcomes.	PLoS ONE.2016; 11 (11).	Small sample size (less than 100 patients)
264	Koc Y, et al	2016	Is peritoneal dialysis a therapeutic option for polycystic kidney disease? 15 years' experience in a single center.	Nephrologie et Therapeutique.2016; 12 (4): 215-20.	Small sample size (less than 100 patients)
265	Lanot a, et al	2016	Efficacy of Prophylactic Antibiotics at Peritoneal Catheter Insertion on Early Peritonitis: Data from the Catheter Section of the French Language Peritoneal Dialysis Registry.	American Journal of Nephrology.2016; 44 (6): 419-425.	Data insufficient to calculate effect estimate
266	Lee JH, et al	2016	Impact of dialysis modality on technique survival in end-stage renal disease patients.	Korean Journal of Internal Medicine.2016; 31 (1): 106-115.	Not relevant in the context of PD-associated peritonitis
267	Ma TKW, et al	2016	Peritonitis before peritoneal dialysis training: Analysis of causative organisms, clinical outcomes, risk factors, and long-term consequences.	Clinical Journal of the American Society of Nephrology.2016; 11 (7): 1219-1226.	Specific organism (pretraining peritonitis episode)
268	Masakane I, et al	2016	Peritoneal Dialysis Registry With 2013 Survey Report.	Ther Apher Dial. 2016 Dec;20(6):557-568.	Data insufficient to calculate effect estimate
269	Muthucumarana K, et al	2016	The Relationship Between Presentation and the Time of Initial Administration of Antibiotics With Outcomes of Peritonitis in Peritoneal Dialysis Patients: The PROMPT Study.	Kidney International Reports.2016; 1 (2): 65-72.	Specific population (only peritonitis cases)

Table S3 Excluded Studies (Continued)

List	Source	Year	Title	Journal	Reason for Exclusion
270	Nadeau-Fredette AC, et al	2016	Center-Specific Factors Associated with Peritonitis Risk-A Multi-Center Registry Analysis.	Peritoneal Dialysis International.2016; 36 (5): 509-18.	Duplicate population with Cheikh Hassan HI, et al (2022)
271	Neu AM, et al	2016	Implementation of standardized follow-up care significantly reduces peritonitis in children on chronic peritoneal dialysis.	Kidney International.2016; 89 (6): 1346-1354.	Duplicate population with Neu AM, et al (2021)
272	Perez Fontan M, et al	2016	Baseline Residual Kidney Function and Its Ensuing Rate of Decline Interact to Predict Mortality of Peritoneal Dialysis Patients.	PLoS ONE [Electronic Resource].2016; 11 (7): e0158696.	Unclear definition of PD-associated peritonitis and data insufficient to calculate effect estimate
273	Qian K, et al	2016	Application of continuous quality improvement in improving positive rate of bacteria culture in patients with peritoneal dialysis related peritonitis.	Chinese Nursing Research.2016; 30 (5B): 1779-1781.	Non-English language (article in Chinese)
274	Ren W, et al	2016	Analysis of peritoneal dialysis-related peritonitis pathogenic bacteria and its drug-resistance.	International Journal of Clinical and Experimental Medicine.2016; 9 (5): 8648-8655.	Specific population (only peritonitis cases)
275	Ros-Ruiz S, et al	2016	The impact of interventional nephrologists on the growth of a peritoneal dialysis program: Long-term, single-center experience.	Nefrologia.2016; 36 (1): 19-23.	Unclear definition of PD-associated peritonitis and data insufficient to calculate effect estimate
276	Sağmak-Tartar A, et al	2016	Continuous ambulatory peritoneal dialysis-related peritonitis: Clinical characteristics, etiological agents and their antibiotic susceptibilities.	Klimik Dergisi.2016; 29 (3): 107-111.	Non-English language (article in Turkish)
277	Sakaci T, et al	2016	Does body mass index affect survival and technique failure in patients undergoing peritoneal dialysis?	Minerva Urologica e Nefrologica.2016; 68 (3): 302-310.	Unclear definition of PD-associated peritonitis and data insufficient to calculate effect estimate
278	Santos C, et al	2016	Identification of Targets for Prevention of Peritoneal Catheter Tunnel and Exit-Site Infections in Low Incidence Settings.	Peritoneal Dialysis International.2016; 36 (1): 43-51.	No outcome reported (incidence data [no. of event and time-at-risk])
279	Şeker A, et al	2016	Evaluation of continuous ambulatory peritoneal dialysis-related peritonitis episodes.	Turkish Nephrology, Dialysis and Transplantation Journal.2016; 25 (2): 142-146.	Non-English language (article in Turkish)
280	Sethna CB, et al	2016	Risk Factors for and Outcomes of Catheter-Associated Peritonitis in Children: The SCOPE Collaborative.	Clinical Journal of The American Society of Nephrology: CJASN.2016; 11 (9): 1590-1596.	Duplicate population with Neu AM, et al (2021)
281	Shea YF, et al	2016	Prevalence of CMMSE defined cognitive impairment among peritoneal dialysis patients and its impact on peritonitis.	Clinical & Experimental Nephrology.2016; 20 (1): 126-33.	Follow-up time/study period 1 year
282	Spigolon DN, et al	2016	Impact of Pre-Dialysis Care on Clinical Outcomes in Peritoneal Dialysis Patients.	American Journal of Nephrology.2016; 43 (2): 104-111.	Unclear definition of PD-associated peritonitis and data insufficient to calculate effect estimate
283	Sun TY, et al	2016	Comparison of peritoneal dialysis catheter insertion techniques: Peritoneoscopic, radiological and laparoscopic : A single-centre study.	Nephrology.2016; 21 (5): 416-22.	Unclear definition of PD-associated peritonitis and data insufficient to calculate effect estimate
284	Szeto CC, et al	2016	Dialysate bacterial endotoxin as a prognostic indicator of peritoneal dialysis related peritonitis.	Nephrology.2016; 21 (12): 1069-1072.	Specific population (only peritonitis cases)
285	Tamayo-Isla RA, et al	2016	Mycobacterial Peritonitis in CAPD Patients in Limpopo: A 6-Year Cumulative Report from a Single Center in South Africa.	Peritoneal Dialysis International.2016; 36 (2): 218-22.	Specific organism (Mycobacterium)
286	Tantiyavarong P, et al	2016	Dialysate White Blood Cell Change after Initial Antibiotic Treatment Represented the Patterns of Response in Peritoneal Dialysis-Related Peritonitis.	International Journal of Nephrology.2016; 1-8.	Specific population (only peritonitis cases)
287	Tu Q, et al	2016	Clinical comparison of modified laparoscopic and conventional placement of peritoneal dialysis catheters.	National Medical Journal of China.2016; 96 (44): 3586-3589.	Non-English language (article in Chinese)
288	van Diepen AT, et al	2016	The Association Between Glucose Exposure and the Risk of Peritonitis in Peritoneal Dialysis Patients.	Peritoneal Dialysis International.2016; 36 (5): 533-9.	Data insufficient to calculate effect estimate

Table S3 Excluded Studies (Continued)

List	Source	Year	Title	Journal	Reason for Exclusion
289	van Esch S, et al	2016	The Natural Time Course of Membrane Alterations During Peritoneal Dialysis Is Partly Altered by Peritonitis.	Peritoneal Dialysis International.2016; 36 (4): 448-56.	Data insufficient to calculate effect estimate
290	van Esch S, et al	2016	The Mutual Relationship Between Peritonitis and Peritoneal Transport.	Peritoneal Dialysis International.2016; 36 (1): 33-42.	Small sample size (less than 100 patients)
291	Velioglu A, et al	2016	Prevention of peritonitis in newly-placed peritoneal dialysis catheters: efficacy of oral prophylaxis with cefuroxime axetil - a preliminary study.	Minerva Urologica e Nefrologica.2016; 68 (1): 27-31.	Small sample size (less than 100 patients)
292	Wang Q, et al	2016	The Association of Individual and Regional Socioeconomic Status on Initial Peritonitis and Outcomes in Peritoneal Dialysis Patients: A Propensity Score-Matched Cohort Study.	Peritoneal Dialysis International.2016; 36 (4): 395-401.	Data insufficient to calculate effect estimate
293	Wu H, et al	2016	Risk Factors for Early-Onset Peritonitis in Southern Chinese Peritoneal Dialysis Patients.	Peritoneal Dialysis International.2016; 36 (6): 640-646.	Duplicate population with Wu H, et al (2022)
294	Xie XS, et al	2016	Peritoneal dialysis for autosomal dominant polycystic kidney disease: a retrospective study.	Journal of Zhejiang University: Science B.2016; 17 (5): 375-381.	Small sample size (less than 100 patients)
295	Yi C, et al	2016	Clinical Outcomes of Remote Peritoneal Dialysis Patients: A Retrospective Cohort Study from a Single Center in China.	Blood Purification.2016; 41 (1-3): 100-7.	Duplicate population with Wu H, et al (2022)
296	Abraham G, et al	2017	Microbiology, clinical spectrum and outcome of peritonitis in patients undergoing peritoneal dialysis in India: Results from a multicentric, observational study.	Indian Journal of Medical Microbiology.2017; 35 (4): 491-498.	Data insufficient to calculate effect estimate
297	Abrahams AC, et al	2017	Improved Outcome of Enteric Peritonitis in Peritoneal Dialysis Patients Aged 50 Years and Older with Temporary Discontinuation of Peritoneal Dialysis and Intravenous Meropenem.	Peritoneal Dialysis International.2017; 37 (3): 298-306.	Data insufficient to calculate effect estimate
298	Bechade C, et al	2017	Centre characteristics associated with the risk of peritonitis in peritoneal dialysis: a hierarchical modelling approach based on the data of the French Language Peritoneal Dialysis Registry.	Nephrology Dialysis Transplantation.2017; 32 (6): 1018-1023.	Data insufficient to calculate effect estimate
299	Bevilacqua MU, et al	2017	Evaluation of a 12-month pilot of long-term and temporary assisted peritoneal dialysis.	Peritoneal Dialysis International.2017; 37 (3): 307-313.	Unclear definition of PD-associated peritonitis and data insufficient to calculate effect estimate
300	Bitencourt Dias D, et al	2017	Urgent-Start Peritoneal Dialysis: The First Year of Brazilian Experience.	Blood Purification.2017; 44 (4): 283-287.	Small sample size (less than 100 patients) and follow-up time/study period 1 year
301	Borzych-Duzalka D, et al	2017	Peritoneal Dialysis Access Revision in Children: Causes, Interventions, and Outcomes.	Clinical Journal of The American Society of Nephrology: CJASN.2017; 12 (1): 105-112.	Not relevant in the context of PD-associated peritonitis
302	Castro ACM, et al	2017	Waist circumference as a predictor of mortality in peritoneal dialysis patients: A follow-up study of 48 months.	British Journal of Nutrition.2017; 117 (9): 1299-1303.	Unclear definition of PD-associated peritonitis and data insufficient to calculate effect estimate
303	Chan S, et al	2017	Association of socio-economic position with technique failure and mortality in australian non-indigenous peritoneal dialysis patients.	Peritoneal Dialysis International.2017; 37 (4): 397-406.	Follow-up time/study period 1 year
304	Fernández P, et al	2017	Peritonitis in peritoneal dialysis. Epidemiology, risk factors, inclusion of bactecTM in traditional culture systems, and long-term mortality.	Revista de Nefrologia, Dialisis y Trasplante.2017; 37 (2): 81-88.	Non-English language (article in Spanish)
305	Figueiredo AE, et al	2017	Comparison between types of dressing following catheter insertion and early exit-site infection in peritoneal dialysis.	Journal of Clinical Nursing.2017; 26 (21-22): 3658-3663.	No outcome reported (incidence data [no. of event and time-at-risk])
306	Gulcan E, et al	2017	Factors affecting patient survival and technical survival in patients undergoing peritoneal dialysis.	International Journal of Clinical and Experimental Medicine.2017; 10 (1): 1004-1014.	Unclear definition of PD-associated peritonitis and data insufficient to calculate effect estimate

Table S3 Excluded Studies (Continued)

List	Source	Year	Title	Journal	Reason for Exclusion
307	Hoffman W, et al	2017	Outcomes of PD Patients at 6 Months.	Peritoneal Dialysis International.2017; 37 (1): 116-118.	Unclear definition of PD-associated peritonitis and data insufficient to calculate effect estimate
308	Ye H, et al	2017	The impact of peritoneal dialysis-related peritonitis on mortality in peritoneal dialysis patients.	BMC Nephrology.2017; 18 1-9.	Data insufficient to calculate effect estimate
309	Hsieh YP, et al	2017	Relationship between uric acid and technique failure in patients on continuous ambulatory peritoneal dialysis: a long-term observational cohort study.	BMJ Open.2017; 7 (4): e010816.	Unclear definition of PD-associated peritonitis and data insufficient to calculate effect estimate
310	Htay H, et al	2017	Outcomes of Corynebacterium Peritonitis: A Multicenter Registry Analysis.	Peritoneal Dialysis International.2017; 37 (6): 619-626.	Data insufficient to calculate effect estimate
311	Htay H, et al	2017	Multicenter Registry Analysis of Center Characteristics Associated with Technique Failure in Patients on Incident Peritoneal Dialysis.	Clinical Journal of The American Society of Nephrology: CJASN.2017; 12 (7): 1090-1099.	No outcome reported (incidence data [no. of event and time-at-risk])
312	Jahangiri F, et al	2017	Surgical Outcome of Peritoneal Dialysis Catheter Insertion in Pediatric Patients: An Experience in Iran.	Iranian Journal of Pediatrics.2017; 27 (5): 1-7.	Small sample size (less than 100 patients)
313	John MM, et al	2017	Impact of residual renal function on clinical outcome and quality of life in patients on peritoneal dialysis.	Saudi Journal of Kidney Diseases & Transplantation.2017; 28 (1): 30-35.	Small sample size (less than 100 patients)
314	Kheir SM, et al	2017	Results of application of the ISPD guidelines to the management of peritoneal dialysis in a single center in Sudan.	Journal of Infection and Public Health.2017; 10 (3): 348-352.	Data insufficient to calculate effect estimate
315	Khoshhali M, et al	2017	Predicting tree-year clinical outcomes using the baseline and trajectories of serum albumin in patients on peritoneal dialysis.	Iranian Red Crescent Medical Journal.2017; 19 (10).	Not relevant in the context of PD-associated peritonitis
316	Kim HJ, et al	2017	Lower Education Level Is a Risk Factor for Peritonitis and Technique Failure but Not a Risk for Overall Mortality in Peritoneal Dialysis under Comprehensive Training System.	PLoS ONE [Electronic Resource].2017; 12 (1): e0169063.	Duplicate population with Kang M, et al (2021)
317	Kitterer D, et al	2017	Gender-Specific Differences in Peritoneal Dialysis.	Kidney & Blood Pressure Research.2017; 42 (2): 276-283.	Unclear definition of PD-associated peritonitis and data insufficient to calculate effect estimate
318	Lee JE, et al	2017	Low serum phosphate as an independent predictor of increased infection-related mortality in dialysis patients: A prospective multicenter cohort study.	PLoS ONE [Electronic Resource].2017; 12 (10): e0185853.	Not relevant in the context of PD-associated peritonitis
319	Lee S, et al	2017	Lower serum potassium associated with increased mortality in dialysis patients: A nationwide prospective observational cohort study in Korea.	PLoS ONE [Electronic Resource].2017; 12 (3): e0171842.	Not relevant in the context of PD-associated peritonitis
320	Li PH, et al	2017	Epidemiology and Clinical Characteristics of Acinetobacter Peritoneal Dialysis-Related Peritonitis in Hong Kong-With a Perspective on Multi-Drug and Carbapenem Resistance.	Peritoneal Dialysis International.2017; 37 (2): 177-182.	Specific organism (Acinetobacter spp.)
321	Li W, et al	2017	Association of baseline, longitudinal serum high-sensitive C-reactive protein and its change with mortality in peritoneal dialysis patients.	BMC Nephrology.2017; 18 (1).	Duplicate population with Wu H, et al (2022)
322	Li WY, et al	2017	Comparison of outcomes between emergent-start and planned-start peritoneal dialysis in incident ESRD patients: a prospective observational study.	BMC Nephrology.2017; 18 (1): 359.	Unclear definition of PD-associated peritonitis and data insufficient to calculate effect estimate
323	Liu SH, et al	2017	The value of time-averaged serum high-sensitivity c-reactive protein in prediction of mortality and dropout in peritoneal dialysis patients.	Therapeutics and Clinical Risk Management.2017; 13 1009-1021.	No outcome reported (incidence data [no. of event and time-at-risk])
324	Ma X, et al	2017	Comparison of therapeutic effects of peritoneal dialysis on uremia patients with diabetic nephropathy and non-diabetic nephropathy.	International Journal of Clinical and Experimental Medicine.2017; 10 (12): 16645-16650.	Small sample size (less than 100 patients)
325	Ma Y, et al	2017	Influence of holidays on peritoneal dialysis patients: an observational study.	Chinese Journal of Clinical Nutrition.2017; 25 (4): 233-239.	Non-English language (article in Chinese)

Table S3 Excluded Studies (Continued)

List	Source	Year	Title	Journal	Reason for Exclusion
326	Machowska A, et al	2017	Dialysis access, infections, and hospitalisations in unplanned dialysis start patients: Results from the OPTiONS study.	International Journal of Artificial Organs.2017; 40 (2): 48-59.	Data insufficient to calculate effect estimate
327	Machowska A, et al	2017	Offering Patients Therapy Options in Unplanned Start (OPTiONS): Implementation of an educational program is feasible and effective.	BMC Nephrology.2017; 18 1-12.	Data insufficient to calculate effect estimate
328	Marinangeli G, et al	2017	[Peritoneal Dialysis in Italy: the fourth GSDP-SIN census 2012].	Giornale Italiano di Nefrologia.2017; 34 (2): 110-137.	Non-English language (article in Italian)
329	Masakane I, et al	2017	Annual peritoneal dialysis report 2014, the peritoneal dialysis registry.	Renal Replacement Therapy.2017; 3 (1).	Data insufficient to calculate effect estimate
330	Mortazavi M, et al	2017	The relationship of high-sensitivity C-reactive protein (Hs-CRP) serum level and peritonitis in patients on peritoneal dialysis.	Journal of Isfahan Medical School.2017; 35 (419): 119-123.	Non-English language (article in Persian)
331	Nakao M, et al	2017	Risk factors for encapsulating peritoneal sclerosis: Analysis of a 36-year experience in a University Hospital.	Nephrology.2017; 22 (11): 907-912.	Data insufficient to calculate effect estimate
332	Ndlovu KCZ, et al	2017	Continuous Ambulatory Peritoneal Dialysis in Patients with HIV and End-Stage Renal Failure.	Peritoneal Dialysis International.2017; 37 (3): 321-330.	Duplicate with Ndlovu KC, et al (2017)
333	Ndlovu KCZ, et al	2017	Peritonitis outcomes in patients with HIV and end-stage renal failure on peritoneal dialysis: a prospective cohort study.	BMC Nephrology.2017; 18 1-11.	Duplicate with Ndlovu KC, et al (2017)
334	Oka H, et al	2017	Modified simple peritoneal wall anchor technique (PWAT) in peritoneal dialysis.	Peritoneal Dialysis International.2017; 37 (1): 103-108.	Small sample size (less than 100 patients)
335	Phui VE, et al	2017	Causative organisms and outcomes of peritoneal dialysis-related peritonitis in Sarawak General Hospital, Kuching, Malaysia: A 3-year analysis.	Renal Replacement Therapy.2017; 3 (1).	Small sample size (less than 100 patients)
336	Ponce D, et al	2017	Peritoneal dialysis for the treatment of cardiorenal syndrome type 1: A prospective Brazilian study.	Peritoneal Dialysis International.2017; 37 (5): 578-583.	Small sample size (less than 100 patients)
337	Prasad N, et al	2017	Measured glomerular filtration rate at dialysis initiation and clinical outcomes of Indian peritoneal dialysis patients.	Indian Journal of Nephrology.2017; 27 (4): 301-306.	Unclear definition of PD-associated peritonitis and data insufficient to calculate effect estimate
338	Querido S, et al	2017	Peritoneal dialysis is a reasonable option in patients with cardiovascular disease.	Clinical Nephrology.2017; 87 (3): 111-116.	Unclear definition of PD-associated peritonitis and data insufficient to calculate effect estimate
339	See EJ, et al	2017	Early and Late Patient Outcomes in Urgent-Start Peritoneal Dialysis.	Peritoneal Dialysis International.2017; 37 (4): 414-419.	Data insufficient to calculate effect estimate
340	Shi Y, et al	2017	Six-minute walk test predicts all-cause mortality and technique failure in ambulatory peritoneal dialysis patients.	Nephrology.2017; 22 (2): 118-124.	Duplicate population with Song Q, et al (2021)
341	Taki Y, et al	2017	Predictive Factors for Withdrawal from Peritoneal Dialysis: A Retrospective Cohort Study at Two Centers in Japan.	Adv Perit Dial.2017; 33 (2017): 68-73.	Not relevant in the context of PD-associated peritonitis
342	Tian Y, et al	2017	Risk Factors and Outcomes of Early-Onset Peritonitis in Chinese Peritoneal Dialysis Patients.	Kidney & Blood Pressure Research.2017; 42 (6): 1266-1276.	Specific population (only peritonitis cases)
343	Vidal E, et al	2017	Infants Requiring Maintenance Dialysis: Outcomes of Hemodialysis and Peritoneal Dialysis.	American Journal of Kidney Diseases.2017; 69 (5): 617-625.	Data insufficient to calculate effect estimate
344	Wan Q, et al	2017	A qualitative, enzyme-linked immunospot assay is a promising diagnostic method for tuberculous peritonitis among continuous ambulatory peritoneal dialysis patients: A single-center experience.	Biomedical Research (India).2017; 28 (22): 9935-9939.	Unclear definition of PD-associated peritonitis and data insufficient to calculate effect estimate
345	Wang C, et al	2017	A Comparison between Intermittent Peritoneal Dialysis and Automatic Peritoneal Dialysis on Urgent Peritoneal Dialysis.	American Journal of Nephrology.2017; 45 (6): 540-548.	No outcome reported (incidence data [no. of event and time-at-risk])

Table S3 Excluded Studies (Continued)

List	Source	Year	Title	Journal	Reason for Exclusion
346	Wang LY, et al	2017	Risk Factors for Early-onset Peritoneal Dialysis-associated Peritonitis.	Chinese General Practice.2017; 20 (4): 454-458.	Non-English language (article in Chinese)
347	Whitty R, et al	2017	Residual Kidney Function and Peritoneal Dialysis-Associated Peritonitis Treatment Outcomes.	Clinical Journal of The American Society of Nephrology: CJASN.2017; 12 (12): 2016-2022.	Specific population (only peritonitis cases)
348	Yeung CS, et al	2017	Risk Factors and Outcomes of Vancomycin-Resistant Enterococcus Colonization in Patients on Peritoneal Dialysis: A Single-Center Study in Hong Kong.	Peritoneal Dialysis International.2017; 37 (5): 556-561.	Unclear definition of PD-associated peritonitis and data insufficient to calculate effect estimate
349	Yi C, et al	2017	Patient-Doctor Contact Interval and Clinical Outcomes in Continuous Ambulatory Peritoneal Dialysis Patients.	American Journal of Nephrology.2017; 45 (4): 346-352.	Duplicate population with Wu H, et al (2022)
350	Zelenitsky SA, et al	2017	Microbiological Trends and Antimicrobial Resistance in Peritoneal Dialysis-Related Peritonitis, 2005 to 2014.	Peritoneal Dialysis International.2017; 37 (2): 170-176.	Specific population (only peritonitis cases)
351	Buffet A, et al	2018	Safety of Peritoneal Dialysis after Nonrenal Solid-Organ Transplantation.	Peritoneal Dialysis International.2018; 38 (1): 37-43.	Data insufficient to calculate effect estimate
352	Cao F, et al	2018	Application of instant messaging software in the follow-up of patients using peritoneal dialysis, a randomised controlled trial.	Journal of Clinical Nursing (John Wiley & Sons, Inc.).2018; 27 (15-16): 3001-3007.	Intervention trials or experimental studies
353	Chaudhri S, et al	2018	Peritoneal dialysis in patients with failed kidney transplant: Single centre experience.	Nephrology.2018; 23 (2): 162-168.	Data insufficient to calculate effect estimate
354	Chen HC, et al	2018	Increasing Staphylococcus Species Resistance in Peritoneal Dialysis-Related Peritonitis over a 10-Year Period in a Single Taiwanese Center.	Peritoneal Dialysis International.2018; 38 (4): 266-270.	Data insufficient to calculate effect estimate
355	Chen JHC, et al	2018	Association between causes of peritoneal dialysis technique failure and all-cause mortality.	Scientific Reports.2018; 8 (1): 3980.	Not relevant in the context of PD-associated peritonitis
356	Crepaldi C, et al	2018	Clinical management of patients on peritoneal dialysis in Italy: Results from the ATENA study.	Clinical Kidney Journal.2018; 11 (2): 275-282.	No outcome reported (incidence data [no. of event and time-at-risk])
357	Davidson B, et al	2018	Outcomes and challenges of a PD-First program, a South-African perspective.	Peritoneal Dialysis International.2018; 38 (3): 179-186.	Data insufficient to calculate effect estimate
358	Dogra PM, et al	2018	Continuous Ambulatory Peritoneal Dialysis Catheter Insertion Technique: A Comparative Study of Percutaneous versus Surgical Insertion.	Indian Journal of Nephrology.2018; 28 (4): 291-297.	Unclear definition of PD-associated peritonitis and data insufficient to calculate effect estimate
359	Ferreira H, et al	2018	Planning Vascular Access in Peritoneal Dialysis-Defining High-Risk Patients.	Peritoneal Dialysis International.2018; 38 (4): 271-277.	Unclear definition of PD-associated peritonitis and data insufficient to calculate effect estimate
360	Guilloteau S, et al	2018	Impact of Assisted Peritoneal Dialysis Modality on Outcomes: A Cohort Study of the French Language Peritoneal Dialysis Registry.	American Journal of Nephrology.2018; 48 (6): 425-433.	Unclear definition of PD-associated peritonitis and data insufficient to calculate effect estimate
361	Hamad A, et al	2018	The epidemiology of acute peritonitis in end-stage renal disease patients on peritoneal dialysis in Qatar: An 8-year follow-up study.	Saudi Journal of Kidney Diseases & Transplantation.2018; 29 (1): 88-94.	Data insufficient to calculate effect estimate
362	Hsu H-J, et al	2018	Encapsulating Peritoneal Sclerosis in Long-Termed Peritoneal Dialysis Patients.	BioMed Research International.2018; 1-7.	Unclear definition of PD-associated peritonitis and data insufficient to calculate effect estimate
363	Htay H, et al	2018	Center Effects and Peritoneal Dialysis Peritonitis Outcomes: Analysis of a National Registry.	American Journal of Kidney Diseases.2018; 71 (6): 814-821.	Data insufficient to calculate effect estimate
364	Htay H, et al	2018	Outcomes of Acinetobacter Peritonitis in Peritoneal Dialysis Patients: A Multicenter Registry Analysis.	Peritoneal Dialysis International.2018; 38 (4): 257-265.	Specific organism (Acinetobacter)

Table S3 Excluded Studies (Continued)

List	Source	Year	Title	Journal	Reason for Exclusion
365	Iida H, et al	2018	Association between keeping home records of catheter exit-site and incidence of peritoneal dialysis-related infections.	International Urology & Nephrology.2018; 50 (4): 763-769.	Small sample size (less than 100 patients) and follow-up time/study period 1 year
366	Inoue H, et al	2018	Non-Tuberculous Mycobacterial Infections Related to Peritoneal Dialysis.	Peritoneal Dialysis International.2018; 38 (2): 147-149.	Specific organism (Nontuberculous mycobacteria)
367	Jiang J, et al	2018	Serum Albumin at Start of Peritoneal Dialysis Predicts Long-Term Outcomes in Anhui Han Patients on Continuous Ambulatory Peritoneal Dialysis: A Retrospective Cohort Study.	Kidney Diseases.2018; 4 (4): 262-268.	No outcome reported (incidence data [no. of event and time-at-risk])
368	Kaul A, et al	2018	Optimization of treatment modality in elderly end-stage renal disease population: Peritoneal dialysis versus transplant.	Indian Journal of Nephrology.2018; 28 (6): 433-440.	Duplicate population with Karthikeyan B, et al (2019)
369	Keskar V, et al	2018	Characteristics and Outcomes of Exit Sites of Buried Peritoneal Dialysis Catheters: A Cohort Study.	Peritoneal Dialysis International.2018; 38 (5): 387-389.	No outcome reported (incidence data [no. of event and time-at-risk])
370	Khalid U, et al	2018	'Educational' Deprivation is Associated with PD Peritonitis.	Peritoneal Dialysis International.2018; 38 (4): 251-256.	Unclear definition of PD-associated peritonitis and data insufficient to calculate effect estimate
371	Kim K, et al	2018	Early technical complications and long-term survival of urgent peritoneal dialysis according to break-in periods.	PLoS ONE [Electronic Resource].2018; 13 (10): e0206426.	Unclear definition of PD-associated peritonitis and data insufficient to calculate effect estimate
372	Krezalek MA, et al	2018	Peritoneal dialysis catheter function and survival are not adversely affected by obesity regardless of the operative technique used.	Surgical Endoscopy.2018; 32 (4): 1714-1723.	Unclear definition of PD-associated peritonitis and data insufficient to calculate effect estimate
373	Kunin M, et al	2018	Intraperitoneal antibiotic administration for prevention of postoperative peritoneal catheter-related infections.	Clinical & Experimental Nephrology.2018; 22 (2): 448-452.	Follow-up time/study period 1 year
374	Lai S, et al	2018	Peritoneal dialysis in older adults: Evaluation of clinical, nutritional, metabolic outcomes, and quality of life.	Medicine.2018; 97 (35): 1-6.	Unclear definition of PD-associated peritonitis and data insufficient to calculate effect estimate
375	Lanot A, et al	2018	Clusters of Practice in Peritoneal Dialysis in France: Data from the Catheter Section of the RDPLF.	Peritoneal Dialysis International.2018; 38 (2): 89-97.	No outcome reported (incidence data [no. of event and time-at-risk])
376	Liu Q, et al	2018	Prevalence of Drug Resistance and Risk Factors Associated with Gram-negative Bacteria in Patients with Peritoneal Dialysis-associated Peritonitis.	Chinese General Practice.2018; 21 (30): 3700-3703.	Non-English language (article in Chinese)
377	Liu S, et al	2018	Application of automated peritoneal dialysis in urgent-start peritoneal dialysis patients during the break-in period.	International Urology and Nephrology.2018; 50 (3): 541-549.	Small sample size (less than 100 patients)
378	Liu Y, et al	2018	Viridans streptococcus peritonitis in peritoneal dialysis: clinical characteristics and comparison with concurrent polymicrobial infection.	BMC Nephrology.2018; 19 (1): 1-8.	Specific organism (Viridans streptococcus)
379	Ma Y, et al	2018	Predictors of short-term outcomes of patients with peritoneal dialysis-associated peritonitis.	Acta Academiae Medicinae Sinicae.2018; 40 (1): 13-20.	Non-English language (article in Chinese)
380	Molnar AO, et al	2018	Hospitalizations in Dialysis Patients in Canada: A National Cohort Study.	Canadian Journal of Kidney Health and Disease.2018; 5.	No outcome reported (incidence data [no. of event and time-at-risk])
381	Munshi R, et al	2018	Fungal peritonitis in the Standardizing Care to Improve Outcomes in Pediatric End Stage Renal Disease (SCOPE) Collaborative.	Pediatric Nephrology.2018; 33 (5): 873-880.	Data insufficient to calculate effect estimate
382	Nayak KS, et al	2018	Emergent Start Peritoneal Dialysis for End-Stage Renal Disease: Outcomes and Advantages.	Blood Purification.2018; 45 (4): 313-319.	Small sample size (less than 100 patients)
383	Ning LN, et al	2018	Risk of First-onset Peritonitis in Peritoneal Dialysis Patients.	Chinese General Practice.2018; 21 (2): 150-154.	Non-English language (article in Chinese)
384	Nochaiwong S, et al	2018	A Clinical Risk Prediction Tool for Peritonitis-Associated Treatment Failure in Peritoneal Dialysis Patients.	Scientific Reports.2018; 8 (1): 14797.	Duplicate population with Noppakun K, et al (2020)

Table S3 Excluded Studies (Continued)

List	Source	Year	Title	Journal	Reason for Exclusion
385	Obi Y, et al	2018	Impact of Obesity on Modality Longevity, Residual Kidney Function, Peritonitis, and Survival Among Incident Peritoneal Dialysis Patients.	American Journal of Kidney Diseases.2018; 71 (6): 802-813.	Unclear definition of PD-associated peritonitis and data insufficient to calculate effect estimate
386	Pecoits-Filho R, et al	2018	Peritonitis as a risk factor for long-term cardiovascular mortality in peritoneal dialysis patients: The case of a friendly fire?	Nephrology.2018; 23 (3): 253-258.	Unclear definition of PD-associated peritonitis and data insufficient to calculate effect estimate
387	Peikani FA, et al	2018	The effect of health-belief-model-based training on behaviors preventing peritonitis in patients on peritoneal dialysis.	International Journal of Preventive Medicine.2018; 9 (1).	Intervention trials or experimental studies
388	Radtke J, et al	2018	Obstruction of peritoneal dialysis catheter is associated with catheter type and independent of omentectomy: A comparative data analysis from a transplant surgical and a pediatric surgical department.	J Pediatr Surg.2018; 53 (4): 640-643.	Follow-up time/study period 1 year and data insufficient to calculate effect estimate
389	Sigogne M, et al	2018	Outcome of autosomal dominant polycystic kidney disease patients on peritoneal dialysis: a national retrospective study based on two French registries (the French Language Peritoneal Dialysis Registry and the French Renal Epidemiology and Information Network).	Nephrology Dialysis Transplantation.2018; 33 (11): 2020-2026.	Unclear definition of PD-associated peritonitis and data insufficient to calculate effect estimate
390	Silva BC, et al	2018	Early start peritoneal dialysis: Technique survival in long-term follow-up.	Kidney and Blood Pressure Research.2018; 43 (6): 1699-1705.	No outcome reported (incidence data [no. of event and time-at-risk])
391	So S, et al	2018	Membrane transport status does not predict peritonitis risk in patients on peritoneal dialysis.	Nephrology.2018; 23 (7): 633-639.	Specific organism (only first episodes) and data insufficient to calculate effect estimate
392	Sumi Y, et al	2018	Current Practice and Outcomes of Peritoneal Dialysis in the Nippon Medical School Musashi Kosugi Hospital.	Journal of Nippon Medical School = Nihon Ika Daigaku Zasshi.2018; 85 (2): 102-109.	Unclear definition of PD-associated peritonitis and data insufficient to calculate effect estimate
393	Swartz SJ, et al	2018	Exit site and tunnel infections in children on chronic peritoneal dialysis: findings from the Standardizing Care to Improve Outcomes in Pediatric End Stage Renal Disease (SCOPE) Collaborative.	Pediatric Nephrology.2018; 33 (6): 1029-1035.	No outcome reported (incidence data [no. of event and time-at-risk])
394	Szeto CC, et al	2018	Treatment of enterococcal peritonitis in peritoneal dialysis patients by oral amoxicillin or intra-peritoneal vancomcyin: A retrospective study.	Kidney and Blood Pressure Research.2018; 42 (5): 837-843.	Duplicate population with Szeto CC, et al (2022)
395	Thammishetti V, et al	2018	A Retrospective Analysis of Etiology and Outcomes of Refractory CAPD Peritonitis in a Tertiary Care Center from North India.	Peritoneal Dialysis International.2018; 38 (6): 441-446.	Specific organism (only refractory episodes)
396	Tsai C-C, et al	2018	Incidence and clinical features of patients with peritoneal dialysis peritonitis complicated by bacteremia.	Medicine.2018; 97 (49): e13567-e13567.	Data insufficient to calculate effect estimate
397	Verma A, et al	2018	Fungal peritonitis in continuous ambulatory peritoneal dialysis (CAPD) patients.	Journal of Clinical and Diagnostic Research.2018; 12 (8): DC06-DC09.	Unclear definition of PD-associated peritonitis and data insufficient to calculate effect estimate
398	Wang H, et al	2018	Risk factors for peritoneal dialysis–associated peritonitis.	European Journal of Inflammation.2018; 16.	Data insufficient to calculate effect estimate
399	Wishart TFL, et al	2018	A Retrospective Sequential Comparison of Topical Application of Medicated Honey and Povidone Iodine for Preventing Peritoneal Dialysis Catheter-Related Infections.	Peritoneal Dialysis International.2018; 38 (4): 302-305.	Data insufficient to calculate effect estimate
400	Wu C, et al	2018	Peritoneal dialysis in Sichuan province of China - report from the Chinese National Renal Data System.	Renal Failure.2018; 40 (1): 577-582.	Unclear definition of PD-associated peritonitis and data insufficient to calculate effect estimate
401	Yang X, et al	2018	High Intraperitoneal Interleukin-6 Levels Predict Peritonitis in Peritoneal Dialysis Patients: A Prospective Cohort Study.	American Journal of Nephrology.2018; 47 (5): 317-324.	Specific organism (only first episodes)
402	Yi C, et al	2018	Prevalence and Prognosis of Coexisting Frailty and Cognitive Impairment in Patients on Continuous Ambulatory Peritoneal Dialysis.	Scientific Reports.2018; 8 (1): 17305.	Duplicate population with Wu H, et al (2022)

Table S3 Excluded Studies (Continued)

List	Source	Year	Title	Journal	Reason for Exclusion
403	Zaritsky JJ, et al	2018	Epidemiology of peritonitis following maintenance peritoneal dialysis catheter placement during infancy: a report of the SCOPE collaborative.	Pediatric Nephrology.2018; 33 (4): 713-722.	Data insufficient to calculate effect estimate
404	Alatab S, et al	2019	Systemic infusion of autologous adipose tissue-derived mesenchymal stem cells in peritoneal dialysis patients: Feasibility and safety.	Cell Journal.2019; 20 (4): 483-495.	Not relevant in the context of PD-associated peritonitis
405	Ambarsari CG, et al	2019	Five-year experience of continuous ambulatory peritoneal dialysis in children: a single center experience in a developing country.	Medical Journal of Indonesia.2019; 28 (4): 329-337.	Small sample size (less than 100 patients)
406	Andy Tang SO, et al	2019	Demographic characteristics and outcomes of continuous ambulatory peritoneal dialysis related peritonitis in Miri General Hospital, Malaysia.	Medical Journal of Malaysia.2019; 74 (4): 270-274.	Small sample size (less than 100 patients)
407	Arulkumaran N, et al	2019	Causes and risk factors for acute dialysis initiation among patients with end-stage kidney disease - A large retrospective observational cohort study.	Clinical Kidney Journal.2019; 12 (4): 550-558.	Not relevant in the context of PD-associated peritonitis
408	Bartosova M, et al	2019	Peritoneal dialysis vintage and glucose exposure but not peritonitis episodes drive peritoneal membrane transformation during the first years of PD.	Frontiers in Physiology.2019; 10 (APR).	Small sample size (less than 100 patients)
409	Beckwith H, et al	2019	Repeat Peritoneal Dialysis Exit-Site Infection: Definition and Outcomes.	Peritoneal Dialysis International.2019; 39 (4): 344-349.	Data insufficient to calculate effect estimate
410	Bednářová V, et al	2019	Our experience with peritoneal dialysis.	Aktuality v Nefrologii.2019; 25 (2): 52-57.	Non-English language (article in Czech)
411	Benomar M, et al	2019	Peritoneal dialysis after kidney transplant failure: a nationwide matched cohort study from the French Language Peritoneal Dialysis Registry (RDPLF).	Nephrology Dialysis Transplantation.2019; 34 (5): 858-863.	Data insufficient to calculate effect estimate
412	Boudville N, et al	2019	Regional variation in the treatment and prevention of peritoneal dialysis-related infections in the Peritoneal Dialysis Outcomes and Practice Patterns Study.	Nephrology Dialysis Transplantation.2019; 34 (12): 2118-2126.	No outcome reported (incidence data [no. of event and time-at-risk])
413	Carvalho Fiel D, et al	2019	Persistent overhydration associates a significant risk of peritoneal infection with enteric germs in patients treated with peritoneal dialysis.	Nefrologia.2019; 39 (6): 638-645.	Data insufficient to calculate effect estimate
414	Chang TI, et al	2019	Lower serum beta-2 microglobulin levels are associated with worse survival in incident peritoneal dialysis patients.	Nephrology Dialysis Transplantation.2019; 34 (1): 138-145.	No outcome reported (incidence data [no. of event and time-at-risk])
415	Chen H-L, et al	2019	Risk factors associated with outcomes of peritoneal dialysis in Taiwan: An analysis using a competing risk model.	Medicine.2019; 98 (6): e14385-e14385.	Specific organism (only first episodes)
416	Do JY and Kang SH, et al	2019	Association Between Peritonitis and Low Muscle Mass in Peritoneal Dialysis Patients.	Journal of Renal Nutrition.2019; N.PAG-N.PAG.	Data insufficient to calculate effect estimate
417	Dönmez O, et al	2019	Kronik Periton Diyalizi Uygulanan Çocuklarda Peritonitlerin Değerlendirilmesi; Tek Merkez Deneyimi.	Journal of Current Pediatrics / Guncel Pediatri.2019; 17 (1): 140-150.	Small sample size (less than 100 patients)
418	Dönmez O, et al	2019	Outcome of peritoneal-dialysis related peritonitis in children: A single center experience.	Guncel Pediatri.2019; 17 (1): 140-150.	Duplicate with Dönmez O, et al (2019)
419	Gong LF, et al	2019	Peritoneal dialysis catheter insertion using a very-low-site approach: a 5-year experience.	International Urology and Nephrology.2019; 51 (6): 1053-1058.	Small sample size (less than 100 patients)
420	Gorsane I, et al	2019	Peritoneal dialysis in diabetis patients.	Tunisie Medicale.2019; 97 (8-9): 1017-1023.	Non-English language (article in French)
421	Gweon T-G, et al	2019	Risk factors for peritonitis in patients on continuous ambulatory peritoneal dialysis who undergo colonoscopy: a retrospective multicentre study.	BMC Gastroenterology.2019; 19 (1): N.PAG-N.PAG.	Not relevant in the context of PD-associated peritonitis
422	Htay H, et al	2019	Comparison of clinical presentation and outcomes of peritonitis in the elderly and younger peritoneal dialysis patients.	Peritoneal Dialysis International.2019; 39 (2): 163-168.	Cross-sectional studies

Table S3 Excluded Studies (Continued)

List	Source	Year	Title	Journal	Reason for Exclusion
423	Janež J, et al	2019	Peritoneal dialysis-associated peritonitis.	Zdravniski Vestnik.2019; 88 (1-2): 39-49.	Review article
424	Keswani M, et al	2019	Risk factors for early onset peritonitis: the SCOPE collaborative.	Pediatric Nephrology.2019; 34 (8): 1387-1394.	Follow-up time/study period 1 year
425	Lee Y, et al	2019	Incremental Peritoneal Dialysis May be Beneficial for Preserving Residual Renal Function Compared to Full-dose Peritoneal Dialysis.	Scientific Reports.2019; 9 (1): 10105.	Specific organism (only first episodes)
426	Li M, et al	2019	Analysis of outcome and factors correlated with maintenance peritoneal dialysis.	Journal of International Medical Research.2019; 47 (10): 4683-4690.	Unclear definition of PD-associated peritonitis and data insufficient to calculate effect estimate
427	Liu X, et al	2019	Effects of prophylactic antibiotics before peritoneal dialysis catheter implantation on the clinical outcomes of peritoneal dialysis patients.	Renal Failure.2019; 41 (1): 16-23.	Data insufficient to calculate effect estimate
428	Lu W, et al	2019	Peritoneal protein clearance predicts mortality in peritoneal dialysis patients.	Clinical and Experimental Nephrology.2019; 23 (4): 551-560.	No outcome reported (incidence data [no. of event and time-at-risk])
429	Maeda S, et al	2019	Proton pump inhibitor use increases the risk of peritonitis in peritoneal dialysis patients.	PLoS ONE [Electronic Resource].2019; 14 (11): e0224859.	Specific organism (only first episodes)
430	McGill RL, et al	2019	Transfers to Hemodialysis Among US Patients Initiating Renal Replacement Therapy With Peritoneal Dialysis.	Am J Kidney Dis.2019; 74 (5): 620-628.	Unclear definition of PD-associated peritonitis (based on ICD codes) and data insufficient to calculate effect estimate
431	Meng C, et al	2019	Peritoneal Dialysis Cuff-Shaving—A Salvage Therapy For Refractory Exit-Site Infections.	Peritoneal Dialysis International.2019; 39 (3): 276-281.	Small sample size (less than 100 patients)
432	Meng C, et al	2019	Peritoneal Dialysis Cuff-Shaving-A Salvage Therapy for Refractory Exit-Site Infections.	Peritoneal Dialysis International.2019; 39 (3): 276-281.	Duplicate with Meng C, et al (2019)
433	Milan Manani S, et al	2019	Catheter-related infections in peritoneal dialysis: comparison of a single center results and the literature data.	Journal of Nephrology.2019; 32 (5): 837-841.	No outcome reported (incidence data [no. of event and time-at-risk])
434	Mok MMY, et al	2019	A Longitudinal Study on the Prevalence and Risk Factors for Depression and Anxiety, Quality of Life, and Clinical Outcomes in Incident Peritoneal Dialysis Patients.	Peritoneal Dialysis International.2019; 39 (1): 74-82.	Unclear definition of PD-associated peritonitis and data insufficient to calculate effect estimate
435	Ndlovu KCZ, et al	2019	Association of Staphylococcus nasal colonization and HIV in end-stage renal failure patients undergoing peritoneal dialysis.	Renal Failure.2019; 41 (1): 303-313.	Specific organism (Staphylococcus)
436	Oka H, et al	2019	Better Oral Hygiene Habits Are Associated With a Lower Incidence of Peritoneal Dialysis-Related Peritonitis.	Ther Apher Dial. 2019 Apr;23(2):187-194.	Small sample size (less than 100 patients)
437	Peng Y, et al	2019	Changes in Outcomes over Time Among Incident Peritoneal Dialysis Patients in Southern China.	Peritoneal Dialysis International.2019; 39 (4): 382-389.	Duplicate population with Wu H, et al (2022)
438	Peng Y, et al	2019	A half-percutaneous technique for peritoneal dialysis catheter implantation using a modified trocar: a report of 84 cases.	International Urology & Nephrology.2019; 51 (8): 1451-1457.	Small sample size (less than 100 patients)
439	Pethő Á, et al	2019	Bedside placement of peritoneal dialysis catheters - a single-center experience from Hungary.	Ren Fail.2019; 41 (1): 434-438.	Small sample size (less than 100 patients)
440	Pingli C, et al	2019	Application of simulated family wards in training illiterate rural patients with peritoneal catheterization.	Chinese Journal of Practical Nursing.2019; 35 (24): 1873-1877.	Non-English language (article in Chinese) and sample size less than 100 patients
441	Prabhakar N, et al	2019	Outcomes of laparoscopic and open CAPD catheter placement: A single-center experience.	Surgery Open Science.2019; 1 (1): 20-24.	Not relevant in the context of PD-associated peritonitis
442	Prasad N, et al	2019	Long-term outcomes in children on chronic continuous ambulatory peritoneal dialysis: a retrospective cohort study from a developing country.	Pediatric Nephrology.2019; 34 (11): 2389-2397.	Small sample size (less than 100 patients)

Table S3 Excluded Studies (Continued)

List	Source	Year	Title	Journal	Reason for Exclusion
443	Ren W, et al	2019	Clinical features and risk factors of peritoneal dialysis-associated frequent peritonitis.	Acta Medica Mediterranea.2019; 35 (6): 3331-3336.	Data insufficient to calculate effect estimate
444	Rydell H, et al	2019	Fewer hospitalizations and prolonged technique survival with home hemodialysis- a matched cohort study from the Swedish Renal Registry.	BMC Nephrology.2019; 20 (1).	Not relevant in the context of PD-associated peritonitis
445	Sanabria M, et al	2019	Remote patient monitoring program in automated peritoneal dialysis: Impact on hospitalizations.	Peritoneal Dialysis International.2019; 39 (5): 472-478.	No outcome reported (incidence data [no. of event and time-at-risk])
446	Shea YF, et al	2019	Self-Care Peritoneal Dialysis Patients with Cognitive Impairment Have a Higher Risk of Peritonitis in the Second Year.	Peritoneal Dialysis International.2019; 39 (1): 51-58.	Data insufficient to calculate effect estimate
447	Silva F, et al	2019	EXIT-SITE INFECTIONS IN PERITONEAL DIALYSIS: PREDICTIVE FACTORS FOR ADVERSE OUTCOMES.	Bulletin de la Dialyse a Domicile.2019; 2 (3): 127-134.	No outcome reported (incidence data [no. of event and time-at-risk])
448	Tang ASO, et al	2019	Demographic characteristics and outcomes of continuous ambulatory peritoneal dialysis related peritonitis in miri general hospital, Malaysia.	Medical Journal of Malaysia.2019; 74 (4): 270-274.	Small sample size (less than 100 patients)
449	Toda N, et al	2019	Urgent-start peritoneal dialysis after laparoscopic dialysis catheter implantation: A single-center experience.	Renal Replacement Therapy.2019; 5 (1).	Small sample size (less than 100 patients)
450	Tsujikawa H, et al	2019	Development of a risk prediction model for infection-related mortality in patients undergoing peritoneal dialysis.	PLoS ONE [Electronic Resource].2019; 14 (3): e0213922.	Not relevant in the context of PD-associated peritonitis
451	Uçar ZA, et al	2019	The impact of peritonitis on clinical outcomes of PD patients: A single center experience.	Turkish Journal of Nephrology.2019; 28 (1): 54-61.	Data insufficient to calculate effect estimate
452	Ueda R, et al	2019	Effect of diabetes on incidence of peritoneal dialysis-associated peritonitis.	PLoS ONE [Electronic Resource].2019; 14 (12): e0225316.	Duplicate population with Nakao M, et al (2016)
453	Vernier I, et al	2019	Peritoneal catheter infections: data from the French peritoneal dialysis registry (RDPLF) and risk factors.	Bulletin de la Dialyse a Domicile.2019; 2 (3): 135-141.	Unclear definition of PD-associated peritonitis and data insufficient to calculate effect estimate
454	Vikrant S, et al	2019	Clinical profile of tuberculosis in patients with chronic kidney disease: A report from an endemic Country.	Saudi J Kidney Dis Transpl.2019; 30 (2): 470-477.	Not relevant in the context of PD-associated peritonitis
455	Wang L, et al	2019	Peritonitis is an independent risk factor for all-cause mortality and cardiovascular mortality in patients with peritoneal dialysis.	Journal of Shanghai Jiaotong University (Medical Science).2019; 39 (9): 1024-1031.	Non-English language (article in Chinese)
456	Yang JY, et al	2019	Icodextrin Is Associated with a Lower Mortality Rate in Peritoneal Dialysis Patients.	Peritoneal Dialysis International.2019; 39 (3): 252-260.	Unclear definition of PD-associated peritonitis and data insufficient to calculate effect estimate
457	Yang L, et al	2019	The pathogen spectrum and resistance in patients with peritoneal dialysis-associated peritonitis: A single-center, observational clinical study.	Clinical Nephrology.2019; 92 (1): 44-51.	Specific population (only peritonitis cases)
458	Ye H, et al	2019	Urgent-start peritoneal dialysis for patients with end stage renal disease: a 10-year retrospective study.	BMC Nephrology.2019; 20 (1): N.PAG-N.PAG.	Unclear definition of PD-associated peritonitis and data insufficient to calculate effect estimate
459	Zhang N, et al	2019	Risk Factors and Prognosis of Early-onset Peritoneal Dialysis-associated Peritonitis.	Chinese General Practice.2019; 22 (14): 1696-1701.	Non-English language (article in Chinese)
460	Zhang Y, et al	2019	Efficacy of APD in perioperative period of nonabdominal operation for peritoneal dialysis patients.	International Journal of General Medicine.2019; 12 207-212.	Small sample size (less than 100 patients)
461	Zhao S, et al	2019	A simplified percutaneous technique for inserting Tenckhoff catheters for peritoneal dialysis.	Clinical Nephrology.2019; 91 (1): 17-24.	Data insufficient to calculate effect estimate
462	Abdel Aal AK, et al	2020	Comparative Study on the Outcomes of Elective-Start versus Urgent-Start Peritoneal Dialysis Catheter Placement.	Radiology Research and Practice.2020; 2020.	Unclear definition of PD-associated peritonitis and data insufficient to calculate effect estimate

Table S3 Excluded Studies (Continued)

List	Source	Year	Title	Journal	Reason for Exclusion
463	Aguilera-Flórez AI, et al	2020	Análisis del tiempo de entrenamiento y su relación con los episodios de peritonitis.	Enfermería Nefrológica.2020; 23 (1): 54-59.	Non-English language (article in Spanish)
464	Ahmad M, et al	2020	Intraperitoneal antibiotic utilization among continuous ambulatory peritoneal dialysis (CAPD) patients with peritonitis at a tertiary hospital setting in Malaysia.	Journal of Pharmacy and Bioallied Sciences.2020; 12 (6): S737-S742.	Unclear definition of PD-associated peritonitis and data insufficient to calculate effect estimate
465	Alsuhaibani M, et al	2020	Fungal peritonitis in children on peritoneal dialysis at a tertiary care Centre.	BMC Nephrology.2020; 21 (1): N.PAG-N.PAG.	Specific organism (fungal and bacterial episodes)
466	Asi T, et al	2020	Determinants of outcomes in chronic pediatric peritoneal dialysis: a single center experience.	Turkish Journal of Pediatrics.2020; 62 (6): 940-948.	Data insufficient to calculate effect estimate
467	Bai Q, et al	2020	Serum Sphingosine-1-phosphate level and peritonitis in peritoneal dialysis patients.	Renal Failure.2020; 42 (1): 829-835.	Small sample size (less than 100 patients)
468	Boyer A, et al	2020	Trends in assisted peritoneal dialysis over the last decade: A cohort study from the French Peritoneal Dialysis Registry.	Clinical Kidney Journal.2020; 13 (6): 1003-1011.	Unclear definition of PD-associated peritonitis and data insufficient to calculate effect estimate
469	Chan GC-K, et al	2020	Depression does not predict clinical outcome of Chinese peritoneal Dialysis patients after adjusting for the degree of frailty.	BMC Nephrology.2020; 21 (1): 1-9.	Data insufficient to calculate effect estimate
470	Chung MC, et al	2020	Is combined peritoneal dialysis and hemodialysis redundant? A nationwide study from Taiwan.	BMC Nephrology.2020; 21 (1): 348.	Unclear definition of PD-associated peritonitis (based on ICD codes)
471	Dao Bui Quy Q, et al	2020	Overhydration and low serum prealbumin predict peritoneal dialysis-related peritonitis in continuous ambulatory peritoneal dialysis patients.	BMC Nephrology.2020; 21 (1): N.PAG-N.PAG.	Data insufficient to calculate effect estimate
472	Do JY, et al	2020	Association Between Peritonitis and Low Muscle Mass in Peritoneal Dialysis Patients.	Journal of Renal Nutrition.2020; 30 (4): 341-346.	Data insufficient to calculate effect estimate
473	Goodlad C, et al	2020	Measurement of innate immune response biomarkers in peritoneal dialysis effluent using a rapid diagnostic point-of-care device as a diagnostic indicator of peritonitis.	Kidney International.2020; 97 (6): 1253-1259.	Not relevant in the context of PD-associated peritonitis
474	He P, et al	2020	Red blood cell distribution width and peritoneal dialysis-associated peritonitis prognosis.	Renal Failure.2020; 42 (1): 613-621.	Specific population (only peritonitis cases)
475	Hong YA, et al	2020	Low parathyroid hormone level predicts infection-related mortality in incident dialysis patients: a prospective cohort study.	Korean Journal of Internal Medicine.2020; 35 (1): 160-170.	No outcome reported (incidence data [no. of event and time-at-risk])
476	Htay H, et al	2020	Multicentre registry data analysis comparing outcomes of culture-negative peritonitis and different subtypes of culture-positive peritonitis in peritoneal dialysis patients.	Peritoneal Dialysis International.2020; 40 (1): 47-56.	Data insufficient to calculate effect estimate
477	Jeon Y, et al	2020	Clinical outcomes of infection-related hospitalization in incident peritoneal dialysis patients.	Kidney Research and Clinical Practice.2020; 39 (4): 460-468.	No outcome reported (incidence data [no. of event and time-at-risk])
478	Kamath N, et al	2020	Clinical and dialysis outcomes of manual chronic peritoneal dialysis in low-body-weight children from a low-to-middle-income country.	Peritoneal Dialysis International.2020; 40 (1): 6-11.	Small sample size (less than 100 patients)
479	Kanjanabuch T, et al	2020	The culture from peritoneal dialysis catheter enhances yield of microorganism identification in peritoneal dialysis-related peritonitis.	Peritoneal Dialysis International.2020; 40 (1): 93-95.	Not relevant in the context of PD-associated peritonitis
480	Kim JH, et al	2020	Percutaneous peritoneal dialysis catheter implantation with no break-in period: A viable option for patients requiring unplanned urgent-start peritoneal dialysis.	Kidney Research and Clinical Practice.2020; 39 (3): 365-372.	Follow-up time/study period 1 year
481	Kokubu M, et al	2020	Relationship between initial peritoneal dialysis modality and risk of peritonitis.	Scientific Reports.2020; 10 (1): 18763.	Data insufficient to calculate effect estimate
482	Koniman R, et al	2020	Early technique failure in peritoneal dialysis patients in a multi-ethnic Asian country.	International Urology & Nephrology.2020; 52 (10): 1987-1994.	Unclear definition of PD-associated peritonitis and data insufficient to calculate effect estimate

Table S3 Excluded Studies (Continued)

List	Source	Year	Title	Journal	Reason for Exclusion
483	Li J, et al	2020	Type D personality, medication adherence and peritonitis in continuous ambulatory peritoneal dialysis patients.	Psychology, Health & Medicine.2020; 25 (5): 541-549.	Data insufficient to calculate effect estimate
484	Lin J, et al	2020	Prevalence and risk factors of exit-site infection in incident peritoneal dialysis patients.	Peritoneal Dialysis International.2020; 40 (2): 164-170.	No outcome reported (incidence data [no. of event and time-at-risk])
485	Lin M, et al	2020	Clinical characteristics of the first episode of peritonitis in elderly patients with peritoneal dialysis.	Chinese Journal of Nephrology.2020; 36 (12): 904-910.	Non-English language (article in Chinese)
486	Ma S, et al	2020	Derivation and validation of a risk score predicting risk of early-onset peritonitis among patients initializing peritoneal dialysis: A cohort study.	International Journal of Infectious Diseases.2020; 99 301-306.	Follow-up time/study period 1 year
487	Meng LF, et al	2020	Comparison of clinical features and outcomes in peritoneal dialysis-associated peritonitis patients with and without diabetes: A multicenter retrospective cohort study.	World Journal of Diabetes.2020; 11 (10): 435-446.	Data insufficient to calculate effect estimate
488	Milan Manani S, et al	2020	Remote monitoring in peritoneal dialysis: benefits on clinical outcomes and on quality of life.	Journal of Nephrology.2020; 33 (6): 1301-1308.	Small sample size (less than 100 patients)
489	Musoke J, et al	2020	The prevalence and bacterial distribution of peritonitis amongst adults undergoing continuous ambulatory peritoneal dialysis at Universitas hospital.	Southern African Journal of Infectious Diseases.2020; 35 (1): 1-5.	Small sample size (less than 100 patients)
490	Ng JK, et al	2020	Helper-assisted continuous ambulatory peritoneal dialysis: Does the choice of helper matter?	Peritoneal Dialysis International.2020; 40 (1): 34-40.	Duplicate population with Than WH, et al (2023)
491	Nitta K, et al	2020	Annual dialysis data report for 2018, JSDT Renal Data Registry: Survey methods, facility data, incidence, prevalence, and mortality.	Renal Replacement Therapy.2020; 6 (1).	Not relevant in the context of PD-associated peritonitis
492	Oliver MJ, et al	2020	Quantifying the risk of insertion-related peritoneal dialysis catheter complications following laparoscopic placement: Results from the North American PD Catheter Registry.	Peritoneal Dialysis International.2020; 40 (2): 185-192.	Follow-up time/study period 1 year
493	Pineda-Borja V, et al	2020	Peritonitis in peritoneal dialysis patients from a hospital in Lima, Peru.	Revista Peruana de Medicina Experimental y Salud Publica.2020; 37 (3): 521-526.	Small sample size (less than 100 patients)
494	Pyart R, et al	2020	The 21st UK Renal Registry Annual Report: A Summary of Analyses of Adult Data in 2017.	Nephron.2020; 144 (2): 59-66.	Unclear definition of PD-associated peritonitis and data insufficient to calculate effect estimate
495	Qiu Y, et al	2020	Age Difference in the Association between Hyponatremia and Infection-Related Mortality in Peritoneal Dialysis Patients.	Blood Purification.2020; 49 (5): 631-640.	Not relevant in the context of PD-associated peritonitis
496	Ren M, et al	2020	Geriatric nutritional risk index is associated with mortality in peritoneal dialysis patients.	Internal Medicine Journal.2020; 50 (4): 470-476.	No outcome reported (incidence data [no. of event and time-at-risk])
497	Scalamogna A, et al	2020	Double purse-string around the inner cuff of the peritoneal catheter: A novel technique for an immediate initiation of continuous peritoneal dialysis.	International Journal of Artificial Organs.2020; 43 (6): 365-371.	Follow-up time/study period 1 year
498	Schuh MP, et al	2020	Omentectomy reduces the need for peritoneal dialysis catheter revision in children: a study from the Pediatric Nephrology Research Consortium.	Pediatric Nephrology.2020; 36 (12): 3953-3959.	Not relevant in the context of PD-associated peritonitis
499	Sharma M, et al	2020	A comparable study on the outcomes of urgent initiated peritoneal dialysis versus conventional start, a single centre study from north-east India.	Iranian Journal of Kidney Diseases.2020; 14 (6): 488-493.	Small sample size (less than 100 patients)
500	Shen Q, et al	2020	Risk factors for loss of residual renal function in children with end-stage renal disease undergoing automatic peritoneal dialysis.	Peritoneal Dialysis International.2020; 40 (4): 368-376.	Small sample size (less than 100 patients)
501	Tanaka M, et al	2020	Hospitalization for Patients on Combination Therapy With Peritoneal Dialysis and Hemodialysis Compared With Hemodialysis.	Kidney International Reports.2020; 5 (4): 468-474.	Small sample size (less than 100 patients)

Table S3 Excluded Studies (Continued)

List	Source	Year	Title	Journal	Reason for Exclusion
502	Tatiyanupanwong S, et al	2020	The Effect of Hypokalemia on Early-onset Peritoneal Dialysis-related Peritonitis.	Medico-Legal Update.2020; 20 (4): 1779-1785.	Follow-up time/study period 1 year
503	Tu YR, et al	2020	Association between initial dialytic modalities and the risks of mortality, infection death, and cardiovascular events: A nationwide population-based cohort study.	Scientific Reports.2020; 10 (1): 8066.	No outcome reported (incidence data [no. of event and time-at-risk])
504	Uiterwijk H, et al	2020	Glucose Exposure in Peritoneal Dialysis Is a Significant Factor Predicting Peritonitis.	American Journal of Nephrology.2020; 51 (3): 237-243.	Data insufficient to calculate effect estimate
505	Vachez C, et al	2020	Impact of diuresis and number of exchanges on Continuous Ambulatory Peritoneal Dialysis related peritonitis risk in RDPLF registry.	Bulletin de la Dialyse a Domicile.2020; 3 (2): 83-92.	Unclear definition of PD-associated peritonitis and data insufficient to calculate effect estimate
506	van Diepen ATN, et al	2020	Comparison of Longitudinal Membrane Function in Peritoneal Dialysis Patients According to Dialysis Fluid Biocompatibility.	Kidney International Reports.2020; 5 (12): 2183-2194.	Not relevant in the context of PD-associated peritonitis
507	Wang Y, et al	2020	Early-start and conventional-start peritoneal dialysis: a Chinese cohort study on outcome.	Renal Failure.2020; 42 (1): 305-313.	Unclear definition of PD-associated peritonitis and data insufficient to calculate effect estimate
508	Wu H, et al	2020	Incidence and risk factors of peritoneal dialysis-related peritonitis in elderly patients: A retrospective clinical study.	Peritoneal Dialysis International.2020; 40 (1): 26-33.	Duplicate population with Wu H, et al (2022)
509	Xia X, et al	2020	Ten-year survival of patients treated with peritoneal dialysis: A prospective observational cohort study.	Peritoneal Dialysis International.2020; 40 (6): 573-580.	Data insufficient to calculate effect estimate
510	Xie D, et al	2020	Percutaneous insertion of peritoneal dialysis catheter is a safe and effective technique irrespective of BMI.	BMC Nephrology.2020; 21 (1): 1-10.	Data insufficient to calculate effect estimate
511	Yan H, et al	2020	Effluent lipopolysaccharide is a prompt marker of peritoneal dialysis-related gram-negative peritonitis.	Peritoneal Dialysis International.2020; 40 (5): 455-461.	Data insufficient to calculate effect estimate
512	Yan J, et al	2020	Comparison of peritoneal dialysis-related peritonitis between patients with polycystic and non-polycystic kidney disease.	Academic Journal of Second Military Medical University.2020; 41 (12): 1394-1397.	Non-English language (article in Chinese)
513	Yang C, et al	2020	A longitudinal analysis of the relationship between serum uric acid and residual renal function loss in peritoneal dialysis patients.	Renal Failure.2020; 42 (1): 447-454.	Unclear definition of PD-associated peritonitis and data insufficient to calculate effect estimate
514	Yangyang C, et al	2020	Clinical features and treatment outcomes of early-onset peritoneal dialysis-associated peritonitis: a multi-center retrospective study.	Chinese Journal of Nephrology.2020; 36 (12): 911-917.	Non-English language (article in Chinese)
515	Ye H, et al	2020	Peritonitis Affects the Relationship Between Low-Density Lipoprotein Cholesterol and Cardiovascular Events in Peritoneal Dialysis Patients.	Canadian Journal of Cardiology.2020; 36 (1): 92-99.	Duplicate population with Wu H, et al (2022)
516	Yu C, et al	2020	Early stage elevated platelet count is an independent risk factor for the poor prognosis of peritoneal dialysis-associated peritonitis.	Chinese Journal of Nephrology.2020; 36 (8): 583-587.	Non-English language (article in Chinese)
517	Zang X, et al	2020	Complications and outcomes of urgent-start peritoneal dialysis in elderly patients with end-stage renal disease in China: a retrospective cohort study.	BMJ Open.2020; 10 (3): e032849.	Unclear definition of PD-associated peritonitis and data insufficient to calculate effect estimate
518	Zhang D, et al	2020	An analysis of the "Half-Perc" versus open surgical placement method for a peritoneal dialysis catheter: a non-inferiority cohort study.	BMC Nephrology.2020; 21 (1): 1-9.	Unclear definition of PD-associated peritonitis and data insufficient to calculate effect estimate
519	Zhang L, et al	2020	Importance of non-medical reasons for dropout in patients on peritoneal dialysis.	Clinical & Experimental Nephrology.2020; 24 (11): 1050-1057.	Unclear definition of PD-associated peritonitis and data insufficient to calculate effect estimate
520	Zhang S, et al	2020	Relationship between serum cystatin C and prognosis of nondiabetic peritoneal dialysis patients.	Therapeutic Apheresis and Dialysis.2020; 24 (6): 703-708.	Unclear definition of PD-associated peritonitis and data insufficient to calculate effect estimate
521	Zhang W, et al	2020	The Synergistic Value of Time-Averaged Serum Albumin and Globulin in Predicting the First Peritonitis in Incident Peritoneal Dialysis Patients.	Blood Purification.2020; 49 (3): 272-280.	Unclear definition of PD-associated peritonitis and data insufficient to calculate effect estimate

Table S3 Excluded Studies (Continued)

List	Source	Year	Title	Journal	Reason for Exclusion
522	Zhang Y, et al	2020	The relationship between neutrophil-to-lymphocyte ratio and the first occurrence of pneumonia in peritoneal dialysis patients.	Clinical and Experimental Nephrology.2020; 24 (9): 770-778.	Not relevant in the context of PD-associated peritonitis
523	Zhou H, et al	2020	Controlling Nutritional Status (CONUT) score as immune-nutritional predictor of outcomes in patients undergoing peritoneal dialysis.	Clinical Nutrition.2020; 39 (8): 2564-2570.	No outcome reported (incidence data [no. of event and time-at-risk])
524	Abrantes ARM, et al	2021	Urgent start peritoneal dialysis: Is there room for more?	Nefrologia.2021; 41 (5): 573-577.	Small sample size (less than 100 patients)
525	Aksoy GK, et al	2021	Evaluation of non-infectious complications of peritoneal dialysis in children: a multicenter study.	Pediatric Nephrology.2021; 36 (2): 417-423.	No outcome reported (incidence data [no. of event and time-at-risk])
526	Aldriwesh M, et al	2021	A Retrospective Analysis of Peritoneal Dialysis-associated Peritonitis at a Single-care Children's Hospital in Saudi Arabia.	Saudi Journal of Kidney Diseases & Transplantation.2021; 32 (3): 735-743.	Small sample size (less than 100 patients)
527	Alves ARV, et al	2021	Overhydration, a new risk factor for peritonitis in peritoneal dialysis.	Iranian Journal of Kidney Diseases.2021; 15 (4): 314-318.	Small sample size (less than 100 patients)
528	Alwakeel J, et al	2021	Effect of bmi on outcome in peritoneal dialysis patients: A single saudi center review.	Kuwait Medical Journal.2021; 53 (2): 140-144.	Small sample size (less than 100 patients)
529	Asai A, et al	2021	Safety and utility of the alpha-replacer for treatment of intraluminal obstruction of peritoneal catheters by fibrin clots.	Clinical and Experimental Nephrology.2021; 25 (4): 418-427.	Not relevant in the context of PD-associated peritonitis
530	Au CWH, et al	2021	Exit site infection and peritonitis due to Serratia species in patients receiving peritoneal dialysis: Epidemiology and clinical outcomes.	Nephrology.2021; 26 (3): 255-261.	Specific organism (Serratia spp.)
531	Banno T, et al	2021	Risk factors for peritoneal dialysis withdrawal due to peritoneal dialysis-related peritonitis.	Nephrologie et Therapeutique.2021; 17 (2): 108-113.	Specific population (only peritonitis cases)
532	Banshodani M, et al	2021	Association between Dialysis Modality and Infectious Diseases: Peritoneal Dialysis versus Hemodialysis.	Blood Purification.2021; 50 (3): 370-379.	Unclear definition of PD-associated peritonitis and data insufficient to calculate effect estimate
533	Basol M, et al	2021	Effect of Serum Albumin Changes on Mortality in Patients with Peritoneal Dialysis: A Joint Modeling Approach and Personalized Dynamic Risk Predictions.	BioMed Research International.2021; 1-10.	Unclear definition of PD-associated peritonitis and data insufficient to calculate effect estimate
534	Bawazir O, et al	2021	Surgical Outcomes of Pediatric Peritoneal Dialysis Catheter Function in a Referral Center.	Saudi Journal of Kidney Diseases and Transplantation.2021; 32 (6): 1586-1592.	Small sample size (less than 100 patients)
535	Ben Salem M, et al	2021	Peritoneal dialysis in tunisia: Complications, technique and patient’s survival (twenty-seven years of experience in a single center).	Pan African Medical Journal.2021; 39.	Unclear definition of PD-associated peritonitis and data insufficient to calculate effect estimate
536	Boonyakrai C, et al	2021	Association between self-reported appetite and clinical outcomes of peritoneal dialysis patients: findings from a low middle-income country.	Nephrology (Carlton, Vic.).2021; 26 (5): 454-462.	Unclear definition of PD-associated peritonitis and data insufficient to calculate effect estimate
537	Boyer A, et al	2021	Trends in Peritoneal Dialysis Technique Survival, Death, and Transfer to Hemodialysis: A Decade of Data from the RDPLF.	American Journal of Nephrology.2021; 52 (4): 318-327.	No outcome reported (incidence data [no. of event and time-at-risk])
538	Camargo CH, et al	2021	Incidence and characteristics of methicillin-resistant coagulase-negative Staphylococcus aureus in peritoneal dialysis-associated peritonitis in a single center using molecular methods.	International Urology & Nephrology.2021; 53 (2): 373-380.	Specific organism (CoNS)
539	Canney M, et al	2021	Maintaining the Uptake of Peritoneal Dialysis During the COVID-19 Pandemic: A Research Letter.	Canadian Journal of Kidney Health and Disease.2021; 8.	Not relevant in the context of PD-associated peritonitis
540	Chang HH, et al	2021	Diabetes Is the Most Critical Risk Factor of Adverse Complications After Peritoneal Dialysis Catheter Placement.	Frontiers in Medicine.2021; 8.	Not relevant in the context of PD-associated peritonitis

Table S3 Excluded Studies (Continued)

List	Source	Year	Title	Journal	Reason for Exclusion
541	Chen CH, et al	2021	Impact of kidney size on mortality in diabetic patients receiving peritoneal dialysis.	Scientific Reports.2021; 11 (1): 8203.	Small sample size (less than 100 patients) and specific population (diabetes)
542	Chen JHC, et al	2021	Associations between diabetes and sex with peritoneal dialysis technique and patient survival: Results from the Australia and New Zealand Dialysis and Transplant Registry cohort study.	Peritoneal Dialysis International.2021; 41 (1): 57-68.	Data insufficient to calculate effect estimate
543	Davies SJ, et al	2021	Low Serum Potassium Levels and Clinical Outcomes in Peritoneal Dialysis—International Results from PDOPPS.	Kidney International Reports.2021; 6 (2): 313-324.	Data insufficient to calculate effect estimate
544	Davis TK, et al	2021	Variability in Culture-Negative Peritonitis Rates in Pediatric Peritoneal Dialysis Programs in the United States.	Clinical Journal of The American Society of Nephrology: CJASN.2021; 16 (2): 233-240.	Data insufficient to calculate effect estimate
545	Díaz Cuevas M, et al	2021	Peritoneal dialysis in incident patients with primary glomerulonephritis. Results of a 20-year multicenter registry study.	Nefrologia.2021; 41 (1): 53-61.	Unclear definition of PD-associated peritonitis and data insufficient to calculate effect estimate
546	Dogra PM, et al	2021	Peritoneal dialysis catheter insertion by nephrologist using minilaparotomy: Do survival and complications vary in obese?	Indian Journal of Nephrology.2021; 31 (2): 124-129.	Unclear definition of PD-associated peritonitis and data insufficient to calculate effect estimate
547	Dzekova-Vidimliski P, et al	2021	Peritoneal Dialysis-Related Peritonitis: Rate, Clinical Outcomes and Patient Survival.	Prilozi Makedonska Akademija Na Naukite I Umetnostite Oddelenie Za Medicinski Nauki.2021; 42 (3): 47-55.	Small sample size (less than 100 patients)
548	Fang X, et al	2021	Clinical Features and Risk Factors of Fungal Peritonitis in Children on Peritoneal Dialysis.	Frontiers in Pediatrics.2021; 9.	Specific organism (fungal and bacterial)
549	González-Sanchidrián S, et al	2021	Analysis of peritoneal catheter exit-site infections. Effectiveness of a protocol based on daily topical mupirocin application.	Enfermeria Nefrologica.2021; 24 (2): 163-173.	Non-English language (article in Spanish)
550	Hayat A, et al	2021	Study of early complications associated with peritoneal dialysis catheters: an analysis of the New Zealand Peritoneal Dialysis Registry data.	International Urology & Nephrology.2021; 53 (8): 1705-1711.	Follow-up time/study period 1 year
551	Hayat A and Saweirs W, et al	2021	Predictors of technique failure and mortality on peritoneal dialysis: An analysis of New Zealand peritoneal dialysis registry data.	Nephrology.2021; 26 (6): 530-540.	Not relevant in the context of PD-associated peritonitis
552	He P, et al	2021	Neutrophil-to-Lymphocyte Ratio and Treatment Failure in Peritoneal Dialysis-Associated Peritonitis.	Frontiers in Medicine.2021; 8.	Specific population (only peritonitis cases)
553	Hepburn KS, et al	2021	Peritoneal dialysis-related peritonitis as a risk factor for cardiovascular events.	Internal Medicine Journal.2021; 51 (3): 404-410.	Not relevant in the context of PD-associated peritonitis
554	Huang X, et al	2021	Risk factors and clinical outcomes of cognitive impairment in diabetic patients undergoing peritoneal dialysis.	Kidney and Blood Pressure Research.2021; 46 (5): 531-540.	Unclear definition of PD-associated peritonitis and data insufficient to calculate effect estimate
555	Kanai H, et al	2021	Efficacy and safety of daprodustat in Japanese peritoneal dialysis patients.	Ther Apher Dial. 2021 Dec;25(6):979-987.	Small sample size (less than 100 patients)
556	Kanjanabuch T, et al	2021	Association of Local Unit Sampling and Microbiology Laboratory Culture Practices With the Ability to Identify Causative Pathogens in Peritoneal Dialysis-Associated Peritonitis in Thailand.	Kidney International Reports.2021; 6 (4): 1118-1129.	Duplicate population with Kanjanabuch T, et al (2022)
557	Kim JS, et al	2021	Safety of Endoscopy in Peritoneal Dialysis Patients.	Clinical and Translational Gastroenterology.2021; 12 (7): e00379.	Follow-up time/study period 1 year
558	Kojima S, et al	2021	Evaluation of modifiable risk factors for peritoneal dialysis-related peritonitis: a single-center retrospective cohort study.	Renal Replacement Therapy.2021; 7 (1).	Small sample size (less than 100 patients)
559	Komatsu S, et al	2021	Association between conscientiousness and peritoneal dialysis-related peritonitis.	Nephrology.2021; 26 (12): 999-1006.	Data insufficient to calculate effect estimate

Table S3 Excluded Studies (Continued)

List	Source	Year	Title	Journal	Reason for Exclusion
560	Kovačević Z, et al	2021	Clinical analysis of peritonitis in peritoneal dialysis patients.	Srpski Arhiv za Celokupno Lekarstvo.2021; 149 (5-6): 282-287.	Small sample size (less than 100 patients)
561	Kuroki Y, et al	2021	Association of blood pressure after peritoneal dialysis initiation with the decline rate of residual kidney function in newly-initiated peritoneal dialysis patients.	PLoS ONE.2021; 16 (7 July).	Unclear definition of PD-associated peritonitis and data insufficient to calculate effect estimate
562	Lanot A, et al	2021	Assisted peritoneal dialysis and transfer to haemodialysis: a cause-specific analysis with data from the RDPLF.	Nephrology Dialysis Transplantation.2021; 36 (2): 330-339.	No outcome reported (incidence data [no. of event and time-at-risk])
563	Lee SM, et al	2021	Comparison of clinical outcome between incremental peritoneal dialysis and conventional peritoneal dialysis: a propensity score matching study.	Renal Failure.2021; 43 (1): 1222-1228.	Duplicate population with Lee SM, et al (2017)
564	Leng TY, et al	2021	Factors predicting clinical outcomes of continuous ambulatory peritoneal dialysis associated peritonitis – a single centre study.	Medical Journal of Malaysia.2021; 76 (3): 382-389.	Small sample size (less than 100 patients)
565	Li L, et al	2021	Analysis of risk factors and construction of prediction model of drop out from peritoneal dialysis.	Medicine (United States).2021; 100 (3): E24195.	Unclear definition of PD-associated peritonitis and data insufficient to calculate effect estimate
566	Liao CT, et al	2021	Aberrant serum parathyroid hormone, calcium, and phosphorus as risk factors for peritonitis in peritoneal dialysis patients.	Scientific Reports.2021; 11 (1): 1171.	Data insufficient to calculate effect estimate
567	Liu L, et al	2021	Serum potassium, albumin and vitamin B₁₂ as potential oxidative stress markers of fungal peritonitis.	Annals of Medicine.2021; 53 (1): 2132-2141.	Data insufficient to calculate effect estimate
568	Liu X, et al	2021	Novel Predictors and Risk Score of Treatment Failure in Peritoneal Dialysis-Related Peritonitis.	Frontiers in Medicine.2021; 8.	Specific population (only peritonitis cases)
569	Ma X, et al	2021	Clinical outcomes, quality of life, and costs evaluation of peritoneal dialysis management models in Shanghai Songjiang District: a multi-center and prospective cohort study.	Renal Failure.2021; 43 (1): 754-765.	Data insufficient to calculate effect estimate
570	Marshall MR, et al	2021	Home Versus Facility Dialysis and Mortality in Australia and New Zealand.	American Journal of Kidney Diseases.2021; 78 (6): 826-836.e1.	Not relevant in the context of PD-associated peritonitis
571	Marshall MR, et al	2021	Peritoneal Dialysis Associated Peritonitis Rate - Validation of a Simplified Formula.	Bulletin de la Dialyse a Domicile.2021; 4 (4): 245-257.	Not relevant in the context of PD-associated peritonitis
572	Mok NMY, et al	2021	Relationship between sodium removal, hydration and outcomes in peritoneal dialysis patients.	Nephrology.2021; 26 (8): 676-683.	Not relevant in the context of PD-associated peritonitis
573	Musbahi A, et al	2021	A modified laparoscopic peritoneal dialysis insertion technique, the 'one scar technique' can minimise short and long term complications: A retrospective cohort study.	Journal of Vascular Access.2021; 22 (5): 744-748.	Not relevant in the context of PD-associated peritonitis
574	Nakayama T, et al	2021	Exercise Parameters Predict Technique Survival in Patients on Peritoneal Dialysis.	Blood Purification.2021; 50 (3): 380-389.	Small sample size (less than 100 patients)
575	Navaratnarajah A, et al	2021	Flexibility in peritoneal dialysis prescription: Impact on technique survival.	Peritoneal Dialysis International.2021; 41 (1): 49-56.	Data insufficient to calculate effect estimate
576	Oki R, et al	2021	Catheter Diversion Procedure With Exit-Site Renewal Promotes Peritoneal Dialysis Catheter Survival.	Kidney International Reports.2021; 6 (2): 325-332.	Data insufficient to calculate effect estimate
577	Phang CC, et al	2021	Comparison of outcomes of urgent-start and conventional-start peritoneal dialysis: a single-centre experience.	International Urology & Nephrology.2021; 53 (3): 583-590.	Data insufficient to calculate effect estimate
578	Portoles J, et al	2021	Is peritoneal dialysis suitable technique CKD patients over 65 years? A prospective multicenter study.	Nefrologia.2021; 41 (5): 529-538.	Unclear definition of PD-associated peritonitis and data insufficient to calculate effect estimate
579	Quero M, et al	2021	Impact of obesity on the evolution of outcomes in peritoneal dialysis patients.	Clinical Kidney Journal.2021; 14 (3): 969-982.	Unclear definition of PD-associated peritonitis and data insufficient to calculate effect estimate

Table S3 Excluded Studies (Continued)

List	Source	Year	Title	Journal	Reason for Exclusion
580	Reis M, et al	2021	Repeat and Relapsing Peritonitis Microbiological Trends and Outcomes: A 21-Year Single-Center Experience.	International Journal of Nephrology.2021; 1-5.	Specific population (only peritonitis cases)
581	Rigo M, et al	2021	Clinical utility of a traditional score system for the evaluation of the peritoneal dialysis exit-site infection in a national multicentric cohort study.	Peritoneal Dialysis International.2021; 41 (3): 292-297.	Unclear definition of PD-associated peritonitis and data insufficient to calculate effect estimate
582	Salem MB, et al	2021	Peritoneal dialysis in Tunisia: complications, technique and patient's survival (twenty-seven years of experience in a single center).	The Pan African medical journal.2021; 39 179.	Duplicate with Ben Salem M, et al (2021)
583	Silva VC, et al	2021	Urgent vs. early-start peritoneal dialysis: Patients' profile and outcomes.	Brazilian Journal of Nephrology.2021; 43 (1): 110-114.	Follow-up time/study period 1 year
584	Slavin M, et al	2021	Considerations for Improved Technique Survival Following Insertion of a Peritoneal Dialysis Catheter.	Israel Medical Association Journal: Imaj.2021; 23 (3): 174-179.	Unclear definition of PD-associated peritonitis and data insufficient to calculate effect estimate
585	Smith B, et al	2021	Outcomes of laparoscopic peritoneal dialysis catheter placement using an optimal placement technique.	Journal of the Society of Laparoendoscopic Surgeons.2021; 25 (1).	No outcome reported (incidence data [no. of event and time-at-risk])
586	Soetendorp H, et al	2021	Water avoidance and modification of exit-site care with stoma bag results in reduced exit-site infection rate in peritoneal dialysis patients.	Clinical Nephrology.2021; 95 (6): 323-331.	Small sample size (less than 100 patients)
587	Sosa Barrios RH, et al	2021	Peritoneal dialysis (PD) technique training: what features influence learning time?	Clinical & Experimental Nephrology.2021; 25 (3): 289-296.	Specific population (only peritonitis cases)
588	Suzuki R, et al	2021	Eosinophilic peritonitis in children on chronic peritoneal dialysis.	Pediatric Nephrology.2021; 36 (6): 1571-1577.	Small sample size (less than 100 patients)
589	Talwar R, et al	2021	Comparative Study of Outcomes Following Laparoscopic Versus Open Peritoneal Dialysis Catheter Insertion at a Tertiary Care Centre.	Journal of Urological Surgery.2021; 8 (1): 40-45.	Small sample size (less than 100 patients)
590	Tan R, et al	2021	Outcomes in buried versus non-buried peritoneal dialysis catheters: A retrospective cohort study.	Journal of Vascular Access.2021; 22 (2): 254-260.	Unclear definition of PD-associated peritonitis and data insufficient to calculate effect estimate
591	Tan YL, et al	2021	Factors predicting clinical outcomes of continuous ambulatory peritoneal dialysis associated peritonitis - A single centre study.	Medical Journal of Malaysia.2021; 76 (3): 382-389.	Duplicate with Leng TY, et al (2021)
592	Tanratananon D, et al	2021	Evaluation of different methods to improve culture-negative peritoneal dialysis-related peritonitis: A single-center study.	Annals of Medicine and Surgery.2021; 63.	Not relevant in the context of PD-associated peritonitis
593	Tawada M, et al	2021	Vasculopathy plays an important role during the development and relapse of encapsulating peritoneal sclerosis with conventional peritoneal dialysis solutions.	Nephrol Dial Transplant.2021; 36 (8): 1519-1526.	Unclear definition of PD-associated peritonitis and data insufficient to calculate effect estimate
594	Teo S, et al	2021	Structured re-training to reduce peritonitis in a pediatric peritoneal dialysis program: a quality improvement intervention.	Pediatric Nephrology.2021; 36 (10): 3191-3200.	Small sample size (less than 100 patients)
595	Terada K, et al	2021	Lower limb ulcer associated with peritonitis in patients undergoing peritoneal dialysis.	Int J Nephrol Renovasc Dis. 2021 Feb 11;14:33-40.	Small sample size (less than 100 patients)
596	Torres H, et al	2021	Evaluating Factors Contributing to Dropout in a Large Peritoneal Dialysis Program.	American Journal of the Medical Sciences.2021; 361 (1): 30-35.	Small sample size (less than 100 patients)
597	Vesga JI, et al	2021	Peritoneal Dialysis Modality Failure in a Middle-Income Country: A Retrospective Cohort Study.	Kidney Medicine.2021; 3 (3): 335-342.e1.	Unclear definition of PD-associated peritonitis and data insufficient to calculate effect estimate
598	Wan S, et al	2021	Baseline serum triglyceride predicts early-onset peritonitis and prognosis in incident CAPD patients.	Medicine.2021; 100 (2): e23673.	Follow-up time/study period 1 year
599	Wang FY, et al	2021	Prognostic implications of predialysis patients’ symptoms in peritoneal dialysis patients.	Renal Failure.2021; 43 (1): 216-222.	No outcome reported (incidence data [no. of event and time-at-risk])

Table S3 Excluded Studies (Continued)

List	Source	Year	Title	Journal	Reason for Exclusion
600	Xinyang L, et al	2021	A multicenter clinical study of 280 cases of staphylococcal peritoneal dialysis-associated peritonitis.	Chinese Journal of Nephrology.2021; 37 (4): 321-326.	Non-English language (article in Chinese)
601	Xu H, et al	2021	Treatment practices and outcomes in incident peritoneal dialysis patients: The Swedish Renal Registry 2006-2015.	Clinical Kidney Journal.2021; 14 (12): 2539-2547.	Unclear definition of PD-associated peritonitis (based on ICD codes) and data insufficient to calculate effect estimate
602	Xu Y, et al	2021	Burden and causes of hospital admissions and readmissions in patients undergoing hemodialysis and peritoneal dialysis: a nationwide study.	Journal of Nephrology.2021; 34 (6): 1949-1959.	No outcome reported (incidence data [no. of event and time-at-risk])
603	Yang Y, et al	2021	Low serum parathyroid hormone is a risk factor for peritonitis episodes in incident peritoneal dialysis patients: a retrospective study.	BMC Nephrology.2021; 22 (1): 1-9.	Specific organism (only first episodes)
604	Yu J, et al	2021	Non-high-density lipoprotein cholesterol and mortality among peritoneal dialysis patients.	Journal of Clinical Lipidology.2021; 15 (5): 732-742.	No outcome reported (incidence data [no. of event and time-at-risk])
605	Yuan J, et al	2021	Association between platelet/lymphocyte ratio and frequent peritoneal dialysis - associated peritonitis in peritoneal dialysis patients.	Chinese Journal of Nephrology.2021; 37 (4): 327-332.	Non-English language (article in Chinese)
606	Zeng Y, et al	2021	Peritoneal dialysis-related peritonitis caused by gram-negative organisms: ten-years experience in a single center.	Renal Failure.2021; 43 (1): 993-1003.	Data insufficient to calculate effect estimate
607	Zhang S, et al	2021	Comparison of prognosis between diabetic and nondiabetic peritoneal dialysis patients.	Acta Medica Mediterranea.2021; 37 (1): 541-546.	Unclear definition of PD-associated peritonitis (based on ICD codes) and data insufficient to calculate effect estimate
608	Zhao L, et al	2021	Risk Factors and Management of Catheter Malfunction During Urgent-Start Peritoneal Dialysis.	Frontiers in Medicine.2021; 8.	No outcome reported (incidence data [no. of event and time-at-risk])
609	Zhong Z, et al	2021	Serum Hepcidin-25 and Risk of Mortality in Patients on Peritoneal Dialysis.	Frontiers in Medicine.2021; 8.	No outcome reported (incidence data [no. of event and time-at-risk])
610	Abdul Rashid A, et al	2022	Straight versus coiled catheters in peritoneal dialysis in a Tertiary Care Center in Malaysia.	Indian Journal of Nephrology.2022; 32 (4): 307-311.	Unclear definition of PD-associated peritonitis (based on ICD codes) and data insufficient to calculate effect estimate
611	Al Sahlawi M, et al	2022	Variation in Peritoneal Dialysis-Related Peritonitis Outcomes in the Peritoneal Dialysis Outcomes and Practice Patterns Study (PDOPPS).	American Journal of Kidney Diseases.2022; 79 (1): 45-55.e1.	No outcome reported (incidence data [no. of event and time-at-risk])
612	Bakal U, et al	2022	Peritoneal dialysis in children: Infectious and mechanical complications: Experience of a tertiary hospital in Elazig, Turkey.	Nigerian Journal of Clinical Practice.2022; 25 (8): 1227-1232.	Small sample size (less than 100 patients)
613	Bakhtar P, et al	2022	Seasonal variations of enteric peritonitis in Belgium and France: from RDPLF data.	Bulletin de la Dialyse a Domicile.2022; 5 (4): 1-9.	Data insufficient to calculate effect estimate
614	Bechade C, et al	2022	Impact of assistance on peritonitis due to breach in aseptic procedure in diabetic patients: A cohort study with the RDPLF data.	Peritoneal Dialysis International.2022; 42 (2): 185-193.	Specific population (diabetes)
615	Bhalla NM, et al	2022	Urgent Start Peritoneal Dialysis: A Population-Based Cohort Study.	Kidney Medicine.2022; 4 (3).	Small sample size (less than 100 patients)
616	Burdeyna O, et al	2022	Pleiotropic effects of atorvastatin in peritoneal dialysis patients: A retrospective-prospective observational cohort study.	Ukrainian Journal of Nephrology and Dialysis.2022; (1): 70-80.	Non-English language (article in Ukraine)
617	Chan GC, et al	2022	Risk of peritonitis after gastroscopy in peritoneal dialysis patients.	Peritoneal Dialysis International.2022; 42 (2): 162-170.	Not relevant in the context of PD-associated peritonitis
618	Chang D, et al	2022	Trimethylamine-N-oxide (TMAO) and clinical outcomes in patients with end-stage kidney disease receiving peritoneal dialysis.	Peritoneal Dialysis International.2022; 42 (6): 622-630.	Specific organism (only first episodes)
619	Cheetham MS, et al	2022	International peritoneal dialysis training practices and the risk of peritonitis.	Nephrology Dialysis Transplantation.2022; 37 (5): 937-949.	Data insufficient to calculate effect estimate

Table S3 Excluded Studies (Continued)

List	Source	Year	Title	Journal	Reason for Exclusion
620	Chou YH, et al	2022	Baseline Peritoneal Membrane Transport Characteristics Are Associated with Peritonitis Risk in Incident Peritoneal Dialysis Patients.	Membranes.2022; 12 (3).	Data insufficient to calculate effect estimate
621	Chung MC, et al	2022	Impact of peritoneal dialysis-related peritonitis on PD discontinuation and mortality: A population-based national cohort study.	Peritoneal Dialysis International.2022; 42 (2): 194-203.	Unclear definition of PD-associated peritonitis (based on ICD codes)
622	Danneville I, et al	2022	Are sex differences in the outcome of peritoneal dialysis explained by nurse assistance? A cohort study with data from the Registre de Dialyse Péritonéale de Langue Française.	Nephrology Dialysis Transplantation.2022; 37 (8): 1520-1528.	Unclear definition of PD-associated peritonitis and data insufficient to calculate effect estimate
623	Diao X, et al	2022	Association of Abnormal Iron Status with the Occurrence and Prognosis of Peritoneal Dialysis-Related Peritonitis: A Longitudinal Data-Based 10-Year Retrospective Study.	Nutrients.2022; 14 (8):	Duplicate population with Wu H, et al (2022)
624	Dong X, et al	2022	Incidence and Risk Factors Associated with Technique Failure in the First Year of Peritoneal Dialysis: A Single Center Retrospective Cohort Study in Southern China.	BMC Nephrology.2022; 23 (1): 1-10.	Data insufficient to calculate effect estimate
625	Duneau G, et al	2022	Characteristics and mortality of patients with and without cardio-renal syndrome treated by peritoneal dialysis in France.	Bulletin de la Dialyse a Domicile.2022; 5 (4): 43-49.	Unclear definition of PD-associated peritonitis and data insufficient to calculate effect estimate
626	El Shamy O, et al	2022	Home Dialysis Utilization Among Underrepresented Groups.	Kidney Medicine.2022; 4 (10).	Unclear definition of PD-associated peritonitis and data insufficient to calculate effect estimate
627	Fernández-Pérez M, et al	2022	Analysis of the mupirocin application in the healthy exit site in peritoneal dialysis.	Enfermeria Nefrologica.2022; 25 (4): 337-342.	Non-English language (article in Spanish)
628	Fox DE, et al	2022	The availability of support and peritoneal dialysis survival: A cohort study.	Peritoneal Dialysis International.2022; 42 (4): 353-360.	No outcome reported (incidence data [no. of event and time-at-risk])
629	Fukunaga T, et al	2022	Safety of peritoneal dialysis catheter surgery under dexmedetomidine and local anesthesia for elderly patients in Japan: a single-center prospective cohort study.	Clinical & Experimental Nephrology.2022; 26 (7): 717-723.	Data insufficient to calculate effect estimate
630	Fukuzaki H, et al	2022	Negative-pressure wound therapy is effective for peritoneal dialysis catheter exit-site management in the early postoperative period.	Scientific Reports.2022; 12 (1): 70.	Intervention trials or experimental studies
631	Guo Q, et al	2022	Poorer clinical outcomes of early-onset peritonitis in elderly peritoneal dialysis patients: A longitudinal and multicenter study.	Ther Apher Dial. 2022 Aug;26(4):815-821.	Data insufficient to calculate effect estimate
632	Guo Q, et al	2022	Influence of Early-Onset Peritonitis on Mortality and Clinical Outcomes in ESRD Patients with Diabetes Mellitus on Peritoneal Dialysis: A Retrospective Multicenter Study.	Blood Purification.2022; 51 (3): 280-287.	Specific population (only peritonitis cases with diabetes)
633	Hama EY, et al	2022	High body mass index is a risk factor for transition to hemodialysis or hybrid therapy and peritoneal dialysis-related infection in Japanese patients undergoing peritoneal dialysis.	International Urology & Nephrology.2022; 54 (12): 3193-3202.	Unclear definition of PD-associated peritonitis and data insufficient to calculate effect estimate
634	Hangai KT, et al	2022	Impact of unplanned peritoneal dialysis start on patients' outcomes—A multicenter cohort study.	Frontiers in Medicine.2022; 9.	Follow-up time/study period 1 year
635	Hasegawa T, et al	2022	Association between the use of exchange devices for peritoneal dialysis fluids and peritonitis incidence: A nationwide cohort study.	Peritoneal Dialysis International.2022; 42 (2): 177-184.	Data insufficient to calculate effect estimate
636	Hiramatsu T, et al	2022	Higher dental care is positively associated with key prognosis factors in peritoneal dialysis patients: findings from a retrospective study.	Renal Replacement Therapy.2022; 8 (1).	Unclear definition of PD-associated peritonitis and data insufficient to calculate effect estimate
637	Hu J, et al	2022	Influence of climatic factors on single-center peritoneal dialysis-associated peritonitis.	Zhong Nan Da Xue Xue Bao Yi Xue Ban. 2022 May 28;47(5):639-649.	Non-English language (article in Chinese)
638	Hu X, et al	2022	Break-in Period <=24 Hours as an Option for Urgent-start Peritoneal Dialysis in Patients With Diabetes.	Frontiers in Endocrinology.2022; 13 936573.	Follow-up time/study period 1 year and unclear definition of PD-associated peritonitis

Table S3 Excluded Studies (Continued)

List	Source	Year	Title	Journal	Reason for Exclusion
639	Huang LL, et al	2022	Incremental peritoneal dialysis is a safe and feasible prescription in incident patients with preserved residual kidney function.	Nephrology.2022; 27 (1): 74-81.	Small sample size (less than 100 patients)
640	Huang YJ, et al	2022	Hypertriglyceridemia is a risk factor for treatment failure in patients with peritoneal dialysis-related peritonitis.	International Urology & Nephrology.2022; 54 (7): 1583-1589.	Small sample size (less than 100 patients)
641	Huo Z, et al	2022	Hypokalemia Duration in the First Year Associated with Subsequent Peritoneal Dialysis-Associated Peritonitis: A Multicenter Retrospective Cohort Study.	Journal of Clinical Medicine.2022; 11 (24).	Data insufficient to calculate effect estimate
642	Jacobs L, et al	2022	[Negative impact of COVID-19 pandemic on peritonitis rate in peritoneal dialysis patients: Pleading for a continuous educational training].	Nephrologie et Therapeutique.2022; 18 (6): 526-533.	Non-English language (article in France)
643	Jin H, et al	2022	Automated peritoneal dialysis as a cost-effective urgent-start dialysis option for ESRD patients: A prospective cohort study.	International Journal of Artificial Organs.2022; 45 (8): 672-679.	Small sample size (less than 100 patients)
644	Jung HY, et al	2022	Sex disparities in mortality among patients with kidney failure receiving dialysis.	Scientific Reports.2022; 12 (1): 18555.	No outcome reported (incidence data [no. of event and time-at-risk])
645	Lee WG, et al	2022	Long-term outcomes of peritoneal dialysis catheters inserted by laparoscopic and percutaneous techniques in a single regional dialysis centre.	ANZ J Surg.2022; 92 (7-8): 1873-1878.	Small sample size (less than 100 patients)
646	Li L, et al	2022	Risk factors of peritoneal dialysis-associated peritonitis and HIV infection.	Chinese Journal of Evidence-Based Medicine.2022; 22 (12): 1382-1385.	Non-English language (article in Chinese)
647	Li Q, et al	2022	Clinical efficacy analysis of peritoneal dialysis in end-stage renal disease with cirrhotic patients.	Chinese Journal of Nephrology.2022; 38 (7): 605-612.	Non-English language (article in Chinese)
648	Li Z, et al	2022	Success rates and safety of a modified percutaneous PD catheter placement technique: Ultrasound-guided percutaneous placement of peritoneal dialysis catheters using a multifunctional bladder paracentesis trocar.	Medicine.2022; 101 (31): e29694-e29694.	Data insufficient to calculate effect estimate
649	Liu M, et al	2022	Changing trends in pathogenic bacteria spectrum of peritoneal dialysis-related peritonitis in a single center in 14 years.	Journal of Army Medical University.2022; 44 (23): 2435-2439.	Non-English language (article in Chinese)
650	Marshall MR, et al	2022	Simplified Calculation of Month-on-Month Annualized Peritoneal Dialysis Associated Peritonitis Rate – Validation in ANZDATA, NZ PD and RDPLF registries.	Bulletin de la Dialyse a Domicile.2022; 5 (3): 179-191.	Data insufficient to calculate effect estimate
651	Meena P, et al	2022	Short- and long-term outcomes of early initiation of peritoneal dialysis following catheter insertion.	Clinical Nephrology.2022; 98 (2): 75-82.	Data insufficient to calculate effect estimate
652	Mendes ML, et al	2022	Unplanned vs. planned peritoneal dialysis as initial therapy for dialysis patients in chronic kidney replacement therapy.	International Urology & Nephrology.2022; 54 (6): 1417-1425.	Unclear definition of PD-associated peritonitis and data insufficient to calculate effect estimate
653	Meng L, et al	2022	Development and Validation of a Prediction Model for the Cure of Peritoneal Dialysis-Associated Peritonitis: A Multicenter Observational Study.	Frontiers in Medicine.2022; 9.	Specific population (only peritonitis cases)
654	Ng AKH, et al	2022	Comparison of planned-start, early-start and deferred-start strategies for peritoneal dialysis initiation in end-stage kidney disease.	Ann Acad Med Singap.2022; 51 (4): 213-220.	Unclear definition of PD-associated peritonitis and data insufficient to calculate effect estimate
655	Ni C, et al	2022	Clinical Characteristics and Treatment Outcome of Pseudomonas Peritoneal Dialysis-associated Peritonitis.	Acta Academiae Medicinae Sinicae.2022; 44 (1): 45-52.	Non-English language (article in Chinese)
656	Niu W, et al	2022	Peritoneal Protein Clearance Is Associated With Cardiovascular Events but Not Mortality in Peritoneal Dialysis Patients.	Frontiers in Medicine.2022; 9.	No outcome reported (incidence data [no. of event and time-at-risk])
657	Özdemir A, et al	2022	Peritoneal Dialysis-related Peritonitis: Microbiological Profile and Outcome.	Medical Journal of Bakirkoy.2022; 18 (1): 25-30.	Small sample size (less than 100 patients)
658	Pilatti M, et al	2022	Urgent vs. planned peritoneal dialysis initiation: complications and outcomes in the first year of therapy.	Brazilian Journal of Nephrology.2022; 44 (4): 482-489.	Unclear definition of PD-associated peritonitis and data insufficient to calculate effect estimate

Table S3 Excluded Studies (Continued)

List	Source	Year	Title	Journal	Reason for Exclusion
659	Pınarbaşı AS, et al	2022	Sociodemographic features and outcomes of peritonitis episodes in children on peritoneal dialysis.	Therapeutic Apheresis and Dialysis.2022; 26 (6): 1264-1273.	Small sample size (less than 100 patients)
660	Puapatanakul P, et al	2022	Assisted peritoneal dialysis performed by caregivers and its association with patient outcomes.	Peritoneal Dialysis International.2022; 42 (6): 602-614.	Specific organism (only first episodes)
661	Purisinsith S, et al	2022	Oral Health-Related Quality of Life, A Proxy of Poor Outcomes in Patients on Peritoneal Dialysis.	Kidney International Reports.2022; 7 (10): 2207-2218.	Duplicate population with Kanjanabuch T, et al (2022)
662	Sabiu G, et al	2022	Influence of peritoneal dialysis catheter type on dislocations and laxative use: a retrospective observational study.	Journal of Nephrology.2022; 35 (5): 1497-1503.	Small sample size (less than 100 patients)
663	Sánchez-Avilés TA, et al	2022	Association between neutrophile-lymphocyte ratio and peritonitis related with peritoneal dialysis.	Medicina Interna de Mexico.2022; 38 (2): 223-234.	Non-English language (article in Spanish)
664	Sato T, et al	2022	The Impact of Preoperative Risk Factors on Peritoneal Dialysis-Related Peritonitis: A Single-Center Prospective Study in Japan.	Medicina.2022; 58 (2): 18.	Data insufficient to calculate effect estimate
665	Sayer M, et al	2022	Intraperitoneal extension of the peritoneal dialysis catheter-a new technique for catheter implantation in patients with obesity.	Journal of Nephrology.2022; 35 (1): 311-316.	Small sample size (less than 100 patients) and no outcome reported
666	Shah N, et al	2022	A Quality Improvement Process to Increase and Sustain a Peritoneal Dialysis Programme in the United Kingdom.	Blood Purification.2022; 51 (12): 1022-1030.	Unclear definition of PD-associated peritonitis and data insufficient to calculate effect estimate
667	Shamy OE, et al	2022	Comparing the effect of peritoneal dialysis cycler type on patient-reported satisfaction, support needs and treatments.	BMC Nephrology.2022; 23 (1).	Small sample size (less than 100 patients) and no outcome reported
668	Shirai Y, et al	2022	Cumulative Dialytic Glucose Exposure is a Risk Factor for Peritoneal Fibrosis and Angiogenesis in Pediatric Patients Undergoing Peritoneal Dialysis Using Neutral-pH Fluids.	Kidney International Reports.2022; 7 (11): 2431-2445.	Small sample size (less than 100 patients)
669	Sia Y, et al	2022	Radiological versus laparoscopic insertion of peritoneal dialysis catheter: a study comparing outcomes at a single centre.	ANZ Journal of Surgery.2022; 92 (11): 2990-2995.	Small sample size (less than 100 patients)
670	Song P, et al	2022	Microbiology and Outcome of Peritoneal Dialysis-Related Peritonitis in Elderly Patients: A Retrospective Study in China.	Frontiers in Medicine.2022; 9.	Data insufficient to calculate effect estimate
671	Song Q, et al	2022	High intraperitoneal interleukin-6 levels predict ultrafiltration (UF) insufficiency in peritoneal dialysis patients: A prospective cohort study.	Frontiers in Medicine.2022; 9.	Unclear definition of PD-associated peritonitis and data insufficient to calculate effect estimate
672	Sosa Barrios RH, et al	2022	Assisted peritoneal dialysis (asPD): age is not the key.	Journal of Nephrology.2022; 35 (9): 2451-2457.	Small sample size (less than 100 patients)
673	Srivastava AK, et al	2022	Clinical profile and microbiological spectrum of patients with continuous ambulatory peritoneal dialysis-associated peritonitis at a tertiary care center.	Med J Armed Forces India. 2023 Dec;79(Suppl 1):S175-S180.	Duplicate with Srivastava AK, et al (2023)
674	Su N, et al	2022	Platelet-to-lymphocyte ratio and the first occurrence of peritonitis in peritoneal dialysis patients.	BMC Nephrology.2022; 23 (1): 415.	Data insufficient to calculate effect estimate
675	Sylvestre LDC, et al	2022	Kt/V or Bicarbonate: What Is More Important for Growth in Pediatric Peritoneal Dialysis Patients?	Blood Purification.2022; 51 68-74.	Small sample size (less than 100 patients)
676	Tan Y, et al	2022	A multi - center study on effect of low serum albumin levels and duration on first episode of peritonitis in continuous ambulatory peritoneal dialysis.	Chinese Journal of Nephrology.2022; 38 (7): 597-604.	Non-English language (article in Chinese)
677	Terada K, et al	2022	Risk Factors for Early Peritoneal Dialysis Discontinuation: Importance of Heart Failure.	Journal of Nippon Medical School = Nihon Ika Daigaku Zasshi.2022; 89 (1): 72-80.	Small sample size (less than 100 patients)
678	Uchiyama K, et al	2022	Eosinophilic Reaction at the Time of Catheter Insertion Predicts Survival in Patients Initiating Peritoneal Dialysis.	Blood Purification.2022; 51 (4): 355-364.	Unclear definition of PD-associated peritonitis and data insufficient to calculate effect estimate

Table S3 Excluded Studies (Continued)

List	Source	Year	Title	Journal	Reason for Exclusion
679	van Eck van der Sluijs A, et al	2022	Differences in hospitalisation between peritoneal dialysis and haemodialysis patients.	European Journal of Clinical Investigation.2022; 52 (6): e13758.	Unclear definition of PD-associated peritonitis and data insufficient to calculate effect estimate
680	Wang IK, et al	2022	The impact of multidisciplinary pre-dialysis care on the outcomes of incident peritoneal dialysis patients.	BMC Nephrology.2022; 23 (1): 1-14.	Specific organism (only first episodes)
681	Wang Z, et al	2022	Effect of Combining Conventional and Telehealth Methods on Managing Peritoneal Dialysis Patients: A Retrospective Single-Center Study.	International Journal of Clinical Practice.2022; 1-8.	Data insufficient to calculate effect estimate and invalid information
682	Wei Z, et al	2022	Clinical Application of Peritoneal Dialysis Catheterization without Capsular Puncture Technique.	BioMed Research International.2022; 1-6.	Article retracted
683	Wen X, et al	2022	Feasibility of a break-in period of less than 24 hours for urgent start peritoneal dialysis: a multicenter study.	Renal Failure.2022; 44 (1): 450-460.	Follow-up time or study period less than 1 year
684	Wu PY, et al	2022	Dialysis Duration and Glucose Exposure Amount Do Not Increase Mortality Risk in Peritoneal Dialysis Patients: A Population-Based Cohort Study From 2004 to 2012.	Frontiers in Medicine.2022; 9.	Unclear definition of PD-associated peritonitis (based on ICD codes)
685	Xu X, et al	2022	Telemedicine and Clinical Outcomes in Peritoneal Dialysis: A Propensity-Matched Study.	American Journal of Nephrology.2022; 53 (8-9): 663-674.	No outcome reported (incidence data [no. of event and time-at-risk])
686	Yan H, et al	2022	Incremental peritoneal dialysis in incident end-stage kidney disease patients.	Peritoneal Dialysis International.2022; 42 (4): 387-393.	Unclear definition of PD-associated peritonitis
687	Yang Y, et al	2022	Interactive effect of hypoparathyroidism and type 2 diabetes mellitus on peritoneal dialysis related peritonitis.	Zhonghua Yi Xue Za Zhi. 2022 Mar 29;102(12):864-869.	Non-English language (article in Chinese)
688	Yin S, et al	2022	Risk Factors and Pathogen Spectrum in Continuous Ambulatory Peritoneal Dialysis-Associated Peritonitis: A Single Center Retrospective Study.	Medical Science Monitor.2022; 28 e937112.	Data insufficient to calculate effect estimate
689	Zhang D, et al	2022	The "Half-Perc" technique using a simple modified metal trocar for peritoneal dialysis catheter placement: results of a 3-year follow-up of 280 patients and a literature review.	International Urology and Nephrology.2022; 54 (7): 1741-1749.	Unclear definition of PD-associated peritonitis and data insufficient to calculate effect estimate
690	Zhang R, et al	2022	The association between diabetes coexisting with low levels of high-density lipoprotein cholesterol and peritoneal dialysis-related peritonitis.	Diabetology and Metabolic Syndrome.2022; 14 (1).	Specific organism (only first episodes)
691	Zhang W, et al	2022	Comparison of risk of peritoneal dialysis-associated peritonitis between roxadustat and recombinant human erythropoietin in peritoneal dialysis patients: a retrospective comparative cohort study.	Annals of Translational Medicine.2022; 10 (22).	Data insufficient to calculate effect estimate
692	Zhang Y, et al	2022	Proton pump inhibitor usage associates with higher risk of first episodes of pneumonia and peritonitis in peritoneal dialysis patients.	Renal Failure.2022; 44 (1): 1623-1631.	Data insufficient to calculate effect estimate
693	Akbalık Kara MA, et al	2023	Outcomes of Maintenance Peritoneal Dialysis in Children: A State Hospital Experience from Southeastern Turkey.	Saudi J Kidney Dis Transpl. 2023 Jan 1;34(1):51-60.	Small sample size (less than 100 patients)
694	Ardalan M, et al	2023	THE FACTORS AFFECTING THE DROPOUT OF PERITONEAL DIALYSIS IN PATIENTS REFERRED TO A DIALYSIS CENTER IN NORTHWEST IRAN.	Journal of Population Therapeutics and Clinical Pharmacology.2023; 30 (19): 1866-1874.	Unclear definition of PD-associated peritonitis and data insufficient to calculate effect estimate
695	Balkoca M, et al	2023	Evaluation of complications in urgent start peritoneal dialysis: Single-center experience.	Ther Apher Dial. 2023 Apr;27(2):314-319.	Unclear definition of PD-associated peritonitis and data insufficient to calculate effect estimate
696	Baran AI, et al	2023	Evaluation of Peritonitis Attacks in Patients Applying Peritoneal Dialysis.	Eastern Journal of Medicine.2023; 28 (4): 745-752.	Small sample size (less than 100 patients)
697	Begin B, et al	2023	Training practices and peritonitis rates in children on maintenance peritoneal dialysis: results from the Standardizing Care to Improve Outcomes in Pediatric End Stage Kidney Disease (SCOPE) collaborative.	Pediatric Nephrology.2023; 38 (10): 3401-3406.	Data insufficient to calculate effect estimate

Table S3 Excluded Studies (Continued)

List	Source	Year	Title	Journal	Reason for Exclusion
698	Biebuyck GKM, et al	2023	Low immunoglobulin G concentrations are not associated with an increased risk of peritoneal dialysis-related peritonitis.	Clinical Nephrology.2023; 99 (4): 180-186.	Data insufficient to calculate effect estimate
699	Bonenkamp AA, et al	2023	Technique failure in peritoneal dialysis: Modifiable causes and patient-specific risk factors.	Peritoneal Dialysis International.2023; 43 (1): 73-83.	No outcome reported (incidence data [no. of event and time-at-risk])
700	Boongird S, et al	2023	Enhancing healthcare quality and outcomes for peritoneal dialysis patients in Thailand: An evaluation of key performance indicators and PDOPPS cohort representativeness.	Nephrology.2023; 28 Suppl 1 14-23.	Not relevant in the context of PD-associated peritonitis
701	Boudville N, et al	2023	A different PET test: The relationship between pet ownership and peritonitis risk in the Peritoneal Dialysis Outcomes and Practice Patterns Study (PDOPPS).	Peritoneal Dialysis International.2023; 43 (3): 263-267.	Data insufficient to calculate effect estimate for each included country
702	Cankaya E, et al	2023	Comparison of mortality according to baseline, first year, and mean albumin levels in peritoneal dialysis: a retrospective study.	Renal Failure.2023; 45 (1): 2176165.	Data insufficient to calculate effect estimate
703	Chan R, et al	2023	Long-term impact of early non-infectious complications at the initiation of peritoneal dialysis.	Peritoneal Dialysis International.2023; 43 (1): 53-63.	Data insufficient to calculate effect estimate
704	Chen J, et al	2023	Long-term survival influencing and risk factors in peritoneal dialysis patients: a single center study in Southwest China.	Chinese Journal of Nephrology.2023; 39 (5): 378-382.	Non-English language (article in Chinese)
705	Chen T, et al	2023	Personality traits and peritoneal dialysis patients' prognosis.	Renal Failure.2023; 45 (2): 2264935.	Unclear definition of PD-associated peritonitis and data insufficient to calculate effect estimate
706	Cheng CI, et al	2023	Implementation of artificial intelligence Chatbot in peritoneal dialysis nursing care: Experience from a Taiwan medical center.	Nephrology.2023; 28 (12): 655-662.	Follow-up time or study period less than 1 year and data insufficient to calculate effect estimate
707	Cheng S, et al	2023	Safety of a 24-h-or-less break-in period in elderly patients undergoing urgent-start peritoneal dialysis: A multicenter retrospective cohort study.	Therapeutic Apheresis and Dialysis.2023; 27 (2): 304-313.	Follow-up time or study period less than 1 year
708	Chong CH, et al	2023	Long-term Trends in Infection-Related Mortality in Adults Treated With Maintenance Dialysis.	American Journal of Kidney Diseases.2023; 82 (5): 597-607.	No outcome reported (incidence data [no. of event and time-at-risk])
709	Dai R, et al	2023	Construction and validation of a predictive model for the risk of peritoneal dialysis-associated peritonitis after peritoneal dialysis catheterization.	Frontiers in Medicine.2023; 10.	Data insufficient to calculate effect estimate
710	Davenport A	2023	Weight loss and weight gains in patients starting peritoneal dialysis; the effect of peritonitis.	Nephrology.2023; 28 (7): 372-378.	Unclear definition of PD-associated peritonitis and data insufficient to calculate effect estimate
711	Ferreira AC, et al	2023	Peritoneal Dialysis Exit-Site Care Protocols in Portugal and Its Association with Catheter-Related Infections.	Blood Purification.2023; 52 (4): 366-372.	Data insufficient to calculate effect estimate
712	Gao L, et al	2023	Outcomes of elderly peritoneal dialysis patients: 65-74 years old versus ≥ 75 years old.	Ren Fail.2023; 45 (2): 2264977.	Unclear definition of PD-associated peritonitis and data insufficient to calculate effect estimate
713	Grubić M, et al	2023	Peritoneal dialysis in Dalmatian County, Croatia: 21 years of a single-center experience.	Ther Apher Dial.2023; 27 (1): 91-99.	Unclear definition of PD-associated peritonitis and data insufficient to calculate effect estimate
714	Guo S, et al	2023	Multidrug-resistant organism-peritoneal dialysis associated peritonitis: clinical and microbiological features and risk factors of treatment failure.	Frontiers in Medicine.2023; 10.	Specific population (only peritonitis cases)
715	He Y, et al	2023	Decreased Peripheral Blood Lymphocyte Count Predicts Poor Treatment Response in Peritoneal Dialysis-Associated Peritonitis.	Journal of Inflammation Research.2023; 16 5327-5338.	Specific population (only peritonitis cases)
716	Hong T, et al	2023	Association between dialysis effluent leukocyte count after initial antibiotic treatment and outcomes of patients with peritoneal dialysis-associated peritonitis: a retrospective study.	Renal Failure.2023; 45 (2): 2258990.	Specific population (only peritonitis cases)
717	Hori S, et al	2023	Clinical impact of catheter insertion for peritoneal dialysis on patient survival and catheter-related complications.	Clinical and Experimental Nephrology.2023; 27 (11): 941-950.	Data insufficient to calculate effect estimate

Table S3 Excluded Studies (Continued)

List	Source	Year	Title	Journal	Reason for Exclusion
718	Kamath N, et al	2023	Pediatric peritoneal dialysis training program and its relationship to peritonitis: a study of the International Pediatric Peritoneal Dialysis Network.	Pediatric Nephrology.2023; 38 (12): 4111-4118.	Data insufficient to calculate effect estimate
719	Kunin M, et al	2023	Optimal peritoneal fluid white blood cell count for diagnosis of peritonitis in peritoneal dialysis patients.	Kidney Research and Clinical Practice.2023; 42 (1): 127-137.	Data insufficient to calculate effect estimate
720	Lei W, et al	2023	Predictive modeling of the risk of treatment failure in patients with peritoneal dialysis-associated peritonitis treated with teicoplanin administered intraperitoneally.	Chinese Journal of Hospital Pharmacy.2023; 43 (18): 2071-2099.	Non-English language (article in Chinese)
721	Li P, et al	2023	Peritoneal Dialysis Care in Mainland China: Nationwide Survey.	JMIR Public Health Surveill.2023; 9 e39568.	Not relevant in the context of PD-associated peritonitis
722	Li SY, et al	2023	Hybrid Method for Peritoneal Dialysis Catheter Insertion: A New Technique for Improved Outcomes and Reduced Costs.	American Journal of Nephrology.2023; 54 (7-8): 349-358.	Not relevant in the context of PD-associated peritonitis
723	Liang Q, et al	2023	Effect of different dialysis duration on the prognosis of peritoneal dialysis-associated peritonitis: a single-center, retrospective study.	Renal Failure.2023; 45 (1): 2177496.	Specific population (only peritonitis cases)
724	Ling CW, et al	2023	Characteristics and outcomes of hospital-acquired and community-acquired peritonitis in patients on peritoneal dialysis: a retrospective cohort study.	Journal of Nephrology.2023; 36 (7): 1877-1888.	Specific population (only peritonitis cases)
725	Liu G, et al	2023	Development and validation of a nomogram for predicting gram-negative bacterial infections in patients with peritoneal dialysis-associated peritonitis.	Heliyon.2023; 9 (8).	Specific population (only peritonitis cases)
726	Liu G, et al	2023	Elevated triglyceride-glucose index predisposes to the initial episode of peritonitis in chronic peritoneal dialysis patients.	Renal Failure.2023; 45 (2): 2267127.	Data insufficient to calculate effect estimate and included only first episode
727	Liu S, et al	2023	Clinical features and risk factors of catheter removal among 1062 episodes of peritoneal dialysis-associated peritonitis: A multicenter 7-year study.	Ther Apher Dial. 2023 Jun;27(3):540-551.	Specific population (only peritonitis cases)
728	Ljungman S, et al	2023	Factors associated with time to first dialysis-associated peritonitis episode: Data from the Peritonitis Prevention Study (PEPS).	Peritoneal dialysis international.2023; 43 (3): 241-251.	Secondary analysis of intervention trial
729	Lumlertgul D, et al	2023	Rescue plan for CAPD peritonitis: Using ultrasonography as a guide on when to remove the catheter.	Ther Apher Dial. 2023 Dec;27(6):1113-1124.	Data insufficient to calculate effect estimate
730	Mao Y, et al	2023	Development of a clinical risk score system for peritoneal dialysis-associated peritonitis treatment failure.	BMC Nephrology.2023; 24 (1): 229.	Specific population (only peritonitis cases)
731	Muller JVC and Ponce D	2023	Infectious and mechanical complications in planned-start vs. urgent-start peritoneal dialysis: a cohort study.	Jornal Brasileiro de Nefrologia.2023; 45 (1): 27-35.	Data insufficient to calculate effect estimate
732	Nardelli L, et al	2023	Incremental peritoneal dialysis allows to reduce the time spent for dialysis, glucose exposure, economic cost, plastic waste and water consumption.	Journal of Nephrology.2023; 36 (2): 263-273.	Duplicate population with Nardelli L, et al (2024)
733	Ni J, et al	2023	Clinical Characteristics of Enterococcus-Associated Peritonitis in Patients with Peritoneal Dialysis.	Infection and Drug Resistance.2023; 16 3399-3405.	Specific organism (only Enterococcus, Streptococcus, and CoNS episodes)
734	Pahwa M, et al	2023	Continuous ambulatory peritoneal dialysis catheter insertion by open technique: 20-year experience from a single center.	Indian Journal of Urology.2023; 39 (1): 46-52.	Unclear definition of PD-associated peritonitis and data insufficient to calculate effect estimate
735	Rojas JE, et al	2023	Clinical and microbiological characterization of Peritoneal Dialysis-Associated Peritonitis events in a hospital in Chile. A retrospective analysis.	Revista Chilena de Infectologia.2023; 40 (2): 94-98.	Non-English language (article in Spanish)
736	Sánchez JE, et al	2023	Impact of peritoneal dialysis strategy on technique and patient survival.	Clinical Kidney Journal.2023; 16 (12): 2523-2529.	No outcome reported (incidence data [no. of event and time-at-risk])
737	Sangthawan P, et al	2023	Time-dependent incidence rates and risk factors for transferring to hemodialysis in patients on peritoneal dialysis under the Thai PD-First Policy.	Peritoneal Dialysis International.2023; 43 (1): 64-72.	Unclear definition of PD-associated peritonitis and data insufficient to calculate effect estimate

Table S3 Excluded Studies (Continued)

List	Source	Year	Title	Journal	Reason for Exclusion
738	Shah N, et al	2023	Comparing surgeon- and nephrologist-inserted Tenckhoff catheters: experience from a metropolitan centre in Sydney.	Internal Medicine Journal.2023; 53 (10): 1890-1895.	Unclear definition of PD-associated peritonitis and data insufficient to calculate effect estimate
739	Shima H, et al	2023	Predictors of culture-negative peritoneal dialysis-associated peritonitis: a single center, retrospective study.	Renal Replacement Therapy.2023; 9 (1).	Specific population (only peritonitis cases)
740	Sunnyraj MM, et al	2023	Peritoneal dialysis outcomes in a tertiary-level state hospital in Johannesburg, South Africa: Ethnicity and HIV co-infection do not increase risk of peritonitis or discontinuation.	South African Medical Journal.2023; 113 (2): 98-103.	Unclear definition of PD-associated peritonitis and data insufficient to calculate effect estimate
741	Than WH, et al	2023	Omentin-1 Levels and Outcomes in Incident Peritoneal Dialysis Patients.	Kidney Medicine.2023; 5 (3).	No outcome reported (incidence data [no. of event and time-at-risk])
742	Than WH, et al	2023	Impact of Weight Gain on the Subsequent Survival of New Peritoneal Dialysis Patients.	Kidney Diseases.2023; 9 (3): 197-205.	Duplicate population with Szeto CC, et al (2022)
743	Thomas KL and Davies M	2023	Survival outcomes of HIV-positive adults on peritoneal dialysis at Helen Joseph renal unit.	Southern African Journal of HIV Medicine.2023; 24 (1).	Small sample size (less than 100 patients)
744	Tian N, et al	2023	Peritoneal dialysis after failed kidney allograft: Comparing patients with and without pd before transplant.	PLoS ONE.2023; 18 (7 July).	Duplicate population with Than WH, et al (2023)
745	Wasik HL, et al	2023	Assessment of potential peritonitis risk factors in pediatric patients receiving maintenance peritoneal dialysis.	Pediatric Nephrology.2023; 38 (12): 4119-4125.	Data insufficient to calculate effect estimate
746	Wu AH, et al	2023	[Predictive value of CONUT score and dialysis age for peritoneal dialysis-associated peritonitis].	Zhonghua Yi Xue Za Zhi.2023; 103 (10): 720-726.	Non-English language (article in Chinese)
747	Xie W, et al	2023	Clinical risk factors for peritoneal dialysis withdrawal at different dialysis duration.	Renal Failure.2023; 45 (2).	Data insufficient to calculate effect estimate
748	Yamaguchi M, et al	2023	Association between reduced left ventricular ejection fraction and peritoneal dialysis related peritonitis: a single center retrospective cohort study in Japan.	Scientific Reports.2023; 13 (1).	Data insufficient to calculate effect estimate
749	Ye J, et al	2023	Construction and validation of a risk prediction model for PDAP caused by gram-negative or gram-positive bacteria.	Journal of Clinical Hematology.2023; 36 (4): 265-270.	Non-English language (article in Chinese)
750	Yeter HH, et al	2023	Outcomes of early-start peritoneal dialysis (PD) and the comparison with urgent-start hemodialysis and conventional-start PD.	Ther Apher Dial. 2023 Jun;27(3):480-487.	Unclear definition of PD-associated peritonitis and data insufficient to calculate effect estimate
751	Young EW, et al	2023	Peritoneal Dialysis–Associated Peritonitis Trends Using Medicare Claims Data, 2013-2017.	American Journal of Kidney Diseases.2023; 81 (2): 179-189.	Unclear definition of PD-associated peritonitis (based on ICD codes)
752	Yu J, et al	2023	Technique failure in peritoneal dialysis-related peritonitis: risk factors and patient survival.	Renal Failure.2023; 45 (1): 2205536.	Specific population (only peritonitis cases)
753	Yuan HZ, et al	2023	[Clinical Outcome and Risk Factors of Treatment Failure of Peritoneal Dialysis Associated Peritonitis Caused by Klebsiella Pneumoniae:A Multicenter Study].	Chung-Kuo i Hsueh Ko Hsueh Yuan Hsueh Pao Acta Academiae Medicinae Sinicae.2023; 45 (2): 227-234.	Non-English language (article in Chinese)
754	Zang Z, et al	2023	Random forest can accurately predict the technique failure of peritoneal dialysis associated peritonitis patients.	Frontiers in Medicine.2023; 10.	Specific population (only peritonitis cases)
755	Zhang F, et al	2023	Pan-immune-inflammation value is associated with poor prognosis in patients undergoing peritoneal dialysis.	Renal Failure.2023; 45 (1): 2158103.	No outcome reported (incidence data [no. of event and time-at-risk])
756	Zhang Y, et al	2023	Body surface area and treatment failure in peritoneal dialysis-associated peritonitis.	Ther Apher Dial. 2023 Oct;27(5):926-936.	Specific population (only peritonitis cases)
757	Zhao Z, et al	2023	Relationship between serum iPTH and peritonitis episodes in patients undergoing continuous ambulatory peritoneal dialysis.	Frontiers in Endocrinology.2023; 14.	Data insufficient to calculate effect estimate

Table S3 Excluded Studies (Continued)

List	Source	Year	Title	Journal	Reason for Exclusion
758	Zhou D, et al	2023	Calculated inflammatory markers derived from complete blood count results, along with routine laboratory and clinical data, predict treatment failure of acute peritonitis in chronic peritoneal dialysis patients.	Renal Failure.2023; 45 (1).	Specific population (only peritonitis cases)
759	Chula DC, et al	2024	Minimally invasive peritoneal access: A new approach of catheter placement for peritoneal dialysis.	J Vasc Access. 2024 Mar;25(2):557-565.	Unclear definition of PD-associated peritonitis and data insufficient to calculate effect estimate
760	Abu Quren AM, et al	2024	Dialysis-associated pathogens at King Fahad Hospital of the University, Eastern Province, Saudi Arabia.	Acta Biomedica.2024; 95 (5).	Small sample size (less than 100 patients)
761	Akiyama M, et al	2024	Frequency and prognosis of peritoneal dialysis-associated peritonitis in children.	Clinical & Experimental Nephrology.2024; 28 (7): 692-700.	Small sample size (less than 100 patients)
762	Altunok M, et al	2024	The effect of body mass index on mortality, peritonitis, technique proficiency and residual renal function in peritoneal dialysis patients.	International Urology and Nephrology.2024; 56 (7): 2379-2389.	Data insufficient to calculate effect estimate
763	Bellanger Q, et al	2024	The impact of cat-related peritonitis on peritoneal dialysis outcomes: Results from the RDPLF.	Peritoneal Dialysis International.2024; 44 (6): 426-433.	Data insufficient to calculate effect estimate
764	Borzych-Dużałka D, et al	2024	Best Practice of Peritoneal Dialysis-Associated Gram-Negative Peritonitis in Children: Insights From the International Pediatric Peritoneal Dialysis Network Registry.	Kidney International Reports.2024; 9 (6): 1654-1663.	Specific organism (only Gram-negative episodes)
765	Cankaya E and Altunok M	2024	Comparison of young and old patients on peritoneal dialysis: A retrospective observational study.	Seminars in Dialysis.2024; 37 (2): 153-160.	Data insufficient to calculate effect estimate
766	Chen C, et al	2024	Comparison of positive rate of peritoneal dialysis-associated peritonitis pathogens cultured by traditional method and modified method and analysis of drug resistance.	Chinese Journal of Nephrology.2024; 40 (4): 300-304.	Non-English language (article in Chinese)
767	Chen JHC, et al	2024	Associations of neutral pH, low-GDP peritoneal dialysis solutions with patient survival, transfer to haemodialysis and peritonitis.	Nephrology Dialysis Transplantation.2024; 39 (2): 222-232.	Specific organism (only first episodes)
768	Demir E, et al	2024	Outcome and Risk Factors for Mortality in Peritoneal Dialysis Patients: 22 Years of Experience in a Turkish Center.	Turkish Journal of Nephrology.2024; 33 (2): 223.	Unclear definition of PD-associated peritonitis and data insufficient to calculate effect estimate
769	Ding Y, et al	2024	Analysis of risk factor for peritonitis in peritoneal dialysis patients.	American Journal of Translational Research.2024; 16 (11): 6964-6971.	Data insufficient to calculate effect estimate
770	Dong X, et al	2024	A predictive model of treatment effectiveness of refractory peritoneal dialysis-related peritonitis in patients with peritoneal dialysis: a single-center observational study in South China.	Clinical Kidney Journal.2024; 17 (12).	Duplicate population with Dong X, et al (2024)
771	Fabbri C, et al	2024	Prognostic Factors of Peritonitis in Patients on Peritoneal Dialysis: a Retrospective Observational Study.	Giornale Italiano di Nefrologia.2024; 41 (3): 121-131.	Data insufficient to calculate effect estimate
772	Francisco D, et al	2024	Transitioning to peritoneal dialysis: it does not matter where you come from.	Jornal Brasileiro de Nefrologia.2024; 46 (3): e20230139.	Unclear definition of PD-associated peritonitis and data insufficient to calculate effect estimate
773	Goldman S, et al	2024	Gastric Acid Suppression Therapy and Its Association with Peritoneal Dialysis–Associated Peritonitis in the Peritoneal Dialysis Outcomes and Practice Patterns Study (PDOPPS).	Kidney360.2024; 5 (3): 370-379.	Data insufficient to calculate effect estimate
774	He Y, et al	2024	Evolution of Peritoneal Dialysis-Associated Peritonitis: Pathogen, Antibiotic Resistance, and the Impact of Lymphocyte Count on Treatment Outcomes.	Infection and Drug Resistance.2024; 17 685-696.	Specific population (only peritonitis cases)
775	Hori S, et al	2024	Impact of nutrition and inflammation indicators on peritoneal dialysis initiation and management.	Clin Exp Nephrol.2024; 28 (4): 349-358.	No outcome reported (incidence data [no. of event and time-at-risk])
776	Hsu CM, et al	2024	Peritoneal Dialysis Technique Survival: A Cohort Study.	Am J Kidney Dis.2024; 84 (3): 298-305.e1.	Data insufficient to calculate effect estimate

Table S3 Excluded Studies (Continued)

List	Source	Year	Title	Journal	Reason for Exclusion
777	Kanjanabuch T, et al	2024	Serum Galactomannan: A Predictor of Poor Outcomes in Peritoneal Dialysis Patients With Fungal Peritonitis.	Kidney International Reports.2024; 9 (2): 287-295.	Specific organism (fungal peritonitis)
778	Keswani M, et al	2024	Interobserver agreement of peritoneal dialysis exit site scoring: Results from the Standardizing Care to Improve Outcomes in Pediatric End Stage Kidney Disease (SCOPE) collaborative.	Peritoneal Dialysis International.2024; 44 (5): 390-396.	Not relevant in the context of PD-associated peritonitis
779	Khan WA, et al	2024	Impact of Prior Abdominal Procedures on Peritoneal Dialysis Catheter Outcomes: Findings From the North American Peritoneal Dialysis Catheter Registry.	American Journal of Kidney Diseases.2024; 84 (2): 195-204.e1.	Not relevant in the context of PD-associated peritonitis
780	Koshitori Y, et al	2024	Delaying treatment for peritonitis could be related to longer hospitalization in patients on peritoneal dialysis.	Renal Replacement Therapy.2024; 10 (1).	Data insufficient to calculate effect estimate
781	Li DS, et al	2024	A time-averaged serum bicarbonate-based nomogram to predict the probability of residual kidney function preservation for patients undergoing peritoneal dialysis.	European Review for Medical and Pharmacological Sciences.2024; 28 (6): 2305-2316.	No outcome reported (incidence data [no. of event and time-at-risk])
782	Li Y, et al	2024	Prognosis and factors related to severe secondary hyperparathyroidism in long-term peritoneal dialysis patients.	Renal Failure.2024; 46 (1): 2356022.	Unclear definition of PD-associated peritonitis and data insufficient to calculate effect estimate
783	Liao R, et al	2024	Risk factors, management and outcomes for peritoneal dialysis access damage.	Renal Failure.2024; 46 (2): 2425161.	Not relevant in the context of PD-associated peritonitis
784	Liao R, et al	2024	COVID-19 and outcomes in Chinese peritoneal dialysis patients.	Peritoneal Dialysis International.2024; 44 (2): 117-124.	Not relevant in the context of PD-associated peritonitis
785	Lim JH, et al	2024	The impact of shortening patient–doctor contact duration on early peritoneal dialysis-related infections.	Scientific Reports.2024; 14 (1).	Duplicate population with Kim JH, et al (2025)
786	Ling Y, et al	2024	Development and validation of prediction model for technique failure in peritoneal dialysis patients: An observational study.	Nephrology.2024; 29 (7): 383-393.	No outcome reported (incidence data [no. of event and time-at-risk])
787	Liu D, et al	2024	Microbiological characteristics and treatment outcome analysis of exit-site infection in peritoneal dialysis patients.	Chinese Journal of Nephrology.2024; 40 (10): 798-803.	Non-English language (article in Chinese)
788	Ma N, et al	2024	Higher N-terminal pro-brain natriuretic peptide level at onset of peritoneal dialysis-related peritonitis is a risk factor for technique failure.	BMC Nephrology.2024; 25 (1): 168.	Specific population (only peritonitis cases)
789	Mao Y, et al	2024	Influencing factors of peritoneal dialysis associated peritonitis and distribution characteristics of pathogenic bacteria.	Journal of Xinxiang Medical University.2024; 41 (3): 257-261.	Non-English language (article in Chinese)
790	Nardelli L, et al	2024	Relationship between number of daily exchanges at CAPD start with clinical outcomes.	Peritoneal Dialysis International.2024; 44 (2): 98-108.	Duplicate population with Nardelli L, et al (2024)
791	Nel H, et al	2024	A retrospective clinical and economic analysis of an assisted automated peritoneal dialysis programme in Western Australia	Perit Dial Int.2024; 44 (3): 203-210.	Small sample size (less than 100 patients)
792	Neri L, et al	2024	Peritoneal Dialysis in Italy: the 8th GPDP-SIN Census 2022 – 2nd Part: the Centers.	Giornale Italiano di Nefrologia.2024; 41 (1): 3-14.	Data insufficient to calculate effect estimate
793	Ren DD, et al	2024	Linking clinical manifestations and causative organisms may provide clues for the treatment of peritoneal dialysis-associated peritonitis.	BMC Nephrology.2024; 25 (1): 322.	Specific population (only peritonitis cases)
794	Sakurada T, et al	2024	Effects of initial peritoneal dialysis prescription on clinical outcomes in Japanese peritoneal dialysis patients: a cohort study.	Scientific Reports.2024; 14 (1): 30109.	Data insufficient to calculate effect estimate
795	Shah AD, et al	2024	Trends, Outcomes, and Economic Implications of Peritoneal Dialysis-Associated Peritonitis Hospitalizations: A National Cohort Study.	American Journal of Nephrology.2024; 55 (4): 472-476.	Unclear definition of PD-associated peritonitis (based on ICD codes) and included only hospitalization cases

Table S3 Excluded Studies (Continued)

List	Source	Year	Title	Journal	Reason for Exclusion
796	Sharba LF, et al	2024	Outcomes and trends in continuous ambulatory peritoneal dialysis: a retrospective analysis at Najaf Centers (2014-2022).	Romanian Journal of Medical Practice.2024; 19 (1): 52-57.	No outcome reported (incidence data [no. of event and time-at-risk])
797	Shen Y, et al	2024	Association between sarcopenic obesity and mortality in patients on peritoneal dialysis: a prospective cohort study.	Frontiers in Medicine.2024; 11.	Unclear definition of PD-associated peritonitis and data insufficient to calculate effect estimate
798	Sirayut W	2024	Fungal peritonitis in peritoneal dialysis: a 5 years retrospective review in Northeast Thailand.	Medical Research Journal (2451-2591).2024; 9 (2): 192-197.	Specific organism (fungal peritonitis)
799	Sugita K, et al	2024	Prognosis and characteristics of Corynebacterium exit site infection: a single-center retrospective study.	Renal Replacement Therapy.2024; 10 (1).	Data insufficient to calculate effect estimate
800	Tang J, et al	2024	The association between new inflammation markers and frequent peritoneal dialysis-associated peritonitis.	BMC Nephrology.2024; 25 (1): 81.	Data insufficient to calculate effect estimate
801	Treamtrakanpon W, et al	2024	Different outcomes between downward and lateral peritoneal dialysis catheter exit-site directions.	Renal Replacement Therapy.2024; 10 (1).	No outcome reported (incidence data [no. of event and time-at-risk])
802	Tsihlis G, et al	2024	Pericatheter Leak Associated With Earlier Peritoneal Dialysis Initiation Does Not Influence Long-Term Outcomes.	Kidney International Reports.2024; 9 (9): 2727-2738.	Data insufficient to calculate effect estimate
803	Tu S, et al	2024	Early Anuria in Incident Peritoneal Dialysis Patients: Incidence, Risk Factors, and Associated Clinical Outcomes.	Kidney Medicine.2024; 6 (10).	Unclear definition of PD-associated peritonitis and data insufficient to calculate effect estimate
804	Wang IK, et al	2024	The impact of icodextrin on the outcomes of incident peritoneal dialysis patients.	PLoS ONE.2024; 19 (3 March).	Data insufficient to calculate effect estimate
805	Wang Z, et al	2024	Association between serum potassium, risk and prognosis of peritonitis in peritoneal dialysis patients - results from the Peritoneal Dialysis Telemedicine-assisted Platform Cohort (PDTAP) Study.	Journal of Nephrology.2024; 37 (7): 1985-1996.	Data insufficient to calculate effect estimate
806	Wongpraphairot S, et al	2024	Comparison of immediate-start peritoneal dialysis without break-in period and conventional-start peritoneal dialysis: a two-center retrospective audit.	International Urology & Nephrology.2024; 56 (7): 2403-2409.	Data insufficient to calculate effect estimate
807	Xia Y, et al	2024	Prognosis of Peritoneal Dialysis Patients with Different Peritoneal Transport Characteristics: A Retrospective Cohort Study.	Iranian Journal of Kidney Diseases.2024; 1 (1): 56-64.	No outcome reported (incidence data [no. of event and time-at-risk])
808	Yan Q, et al	2024	Development and validation of a nomogram for predicting refractory peritoneal dialysis related peritonitis.	Renal Failure.2024; 46 (2): 2368083.	Data insufficient to calculate effect estimate
809	Yang L	2024	Patent report on peritoneal dialysis from representative medical institutions of China in the field of nephrology.	Chinese Journal of Nephrology.2024; 40 (9): 765-770.	Non-English language (article in Chinese)
810	Yaxley J, et al	2024	Peritoneal dialysis catheterization with an upward tunnel and exit site: An observational study.	Journal of Vascular Access.2024.	Duplicate with Yaxley J, et al (2025)
811	Yi C, et al	2024	Effect of a Management Algorithm for Wet Contamination of Peritoneal Dialysis System on the Prevention of Peritonitis: A Prospective Observational Study.	Kidney Diseases.2024; 10 (4): 295-302.	Data insufficient to calculate effect estimate
812	Yooyen M, et al	2024	Incidence and Factors Associated with Peritoneal Dialysis-Related Peritonitis in Children.	Journal of the Medical Association of Thailand.2024; 107 (5): 343-348.	Small sample size (less than 100 patients)
813	You L, et al	2024	Pathogenic spectrum and risk factors of peritoneal dialysis-associated peritonitis: a single-center retrospective study.	BMC Infectious Diseases.2024; 24 (1): 440.	Data insufficient to calculate effect estimate
814	Yu J, et al	2024	Higher Platelet Count Mostly in the Normal Range Is Associated with the First Episode of Peritonitis Risk in Incident Peritoneal Dialysis Patients.	Kidney & Blood Pressure Research.2024; 49 (1): 863-873.	Data insufficient to calculate effect estimate
815	Yuan D, et al	2024	Analysis of risk factors for refractory peritonitis in patients undergoing peritoneal dialysis.	American Journal of Translational Research.2024; 16 (8): 3832-3841.	Specific population (only peritonitis cases)

Table S3 Excluded Studies (Continued)

List	Source	Year	Title	Journal	Reason for Exclusion
816	Zheng S, et al	2024	Advanced Image-Guided Percutaneous Technique Versus Advanced Laparoscopic Surgical Technique for Peritoneal Dialysis Catheter Placement.	Kidney Medicine.2024; 6 (1).	Not relevant in the context of PD-associated peritonitis
817	Zhong Z, et al	2024	Serum fibroblast growth factor 23 concentration and the risk of mortality in patients undergoing peritoneal dialysis.	Peritoneal Dialysis International.2024; 44 (3): 194-202.	Unclear definition of PD-associated peritonitis and data insufficient to calculate effect estimate
818	Zhou J, et al	2024	Intraperitoneal vancomycin plus levofloxacin for the treatment of peritoneal dialysis-related peritonitis in patients with no response to cefazolin plus ceftazidime.	Clinical Nephrology.2024; 101 (4): 164-170.	Specific population (only peritonitis cases)
819	Al Atbee MYN, et al	2025	Spectrum of infectious complications in continuous ambulatory peritoneal dialysis and the role of metabolic derangement to infections.	Clinical Nephrology.2025; 104 (1): 13-22.	Unclear definition of PD-associated peritonitis and data insufficient to calculate effect estimate
820	Al Atbee MYN, et al	2025	Prevalence of Peritonitis in Continuous Ambulatory Peritoneal Dialysis Patients: Bacteriology Analysis.	Clinical Infectology and Parasitology.2025; 14 (1): 78-88.	Duplicate with Al Atbee MYN, et al (2025)
821	Albakr R, et al	2025	Sex-Specific Outcomes in The Peritoneal Dialysis Outcomes and Practice Patterns Study.	Clin J Am Soc Nephrol. 2025 Dec 10. doi: 10.2215/CJN.0000000937.	Specific organism (only first episodes)
822	Albert A, et al	2025	Microbiological Surveillance and Antimicrobial Susceptibility Observations on Peritoneal Dialysis-Associated Peritonitis in an Outpatient German Reference Center.	Infectious Disease Reports.2025; 17 (3).	Data insufficient to calculate effect estimate
823	Bach N, et al	2025	Prevalence, microbiology, and outcome of peritonitis in peritoneal dialysis patients in vietnam: a multicenter study.	BMC Nephrology.2025; 26 (1): 134.	Data insufficient to calculate effect estimate
824	Berke I, et al	2025	Clinical outcomes in peritoneal dialysis with refractory peritonitis: significance of the day 5 cell count.	Clinical & Experimental Nephrology.2025; 29 (2): 221-226.	Specific organism (only refractory episodes)
825	Betancourt L, et al	2025	Prognostic value of high intraperitoneal pressure in patients on peritoneal dialysis.	International Urology and Nephrology.2025; 57 (9): 2927-2934.	No outcome reported (incidence data [no. of event and time-at-risk])
826	Bıldacı YD, et al	2025	Increased Peritoneal Dialysis Associated Peritonitis Episodes During Novel Coronavirus Pandemics: Experience from Western Region in Türkiye.	Turkish Journal of Nephrology.2025; 34 (3): 201-205.	Small sample size (less than 100 patients)
827	Cao Y, et al	2025	Analysis of prognostic prediction and nursing intervention value in PD patients based on nomogram model.	Frontiers in Medicine.2025; 12.	No outcome reported (incidence data [no. of event and time-at-risk])
828	Cheikh Hassan HI, et al	2025	Impact of type of vascular access on clinical outcomes in peritoneal dialysis patients transitioning to haemodialysis: an ANZDATA study.	Clinical Kidney Journal.2025; 18 (3).	No outcome reported (incidence data [no. of event and time-at-risk])
829	Chung MC, et al	2025	Three Years Outcome Comparison of Remote Monitoring Versus Conventional Automated Peritoneal Dialysis in Incident APD Patients: A Retrospective Study From Taiwan.	Nephrology.2025; 30 (7): e70061.	Data insufficient to calculate effect estimate
830	Davenport A	2025	The Effect of Low-grade Inflammation on Body Composition in Peritoneal Dialysis Patients.	J Ren Nutr.2025; 35 (6): 787-794.	No outcome reported (incidence data [no. of event and time-at-risk])
831	Davy A, et al	2025	Bimodal solutions in peritoneal dialysis: what can we expect from different glucose volumes added to the icodextrin bag? Data from a simulation procedure.	J Nephrol.2025; 38 (8): 2189-2197.	Small sample size (less than 100 patients)
832	Díaz-Muñoz JC, et al	2025	Antimicrobial resistance of microorganisms in peritonitis secondary to peritoneal dialysis at Almanzor Aguinaga - Asenjo Hospital, EsSalud-Lambayeque 2019- 2021.	Acta Medica Peruana.2025; 42 (1): 55-60.	Non-English language (article in Spanish)
833	Ekart R, et al	2025	24-years' experience of peritoneal dialysis in a university hospital dialysis center: Management and outcome.	Clinical Nephrology.2025; 104 (7): S41-S46.	Small sample size (less than 100 patients)
834	Fajar JK, et al	2025	The association between serum calcium levels and the calcium-to-phosphate ratio and their potential impact on the incidence of peritonitis in patients undergoing peritoneal dialysis.	Archives of Hellenic Medicine.2025; 42 (3): 347-356.	Unclear definition of PD-associated peritonitis and data insufficient to calculate effect estimate

Table S3 Excluded Studies (Continued)

List	Source	Year	Title	Journal	Reason for Exclusion
835	Faucon AL, et al	2025	Burden of CKD-Associated Pruritus and Adverse Clinical Outcomes in Patients Receiving Dialysis: The Stockholm Creatinine Measurements (SCREAM) Project.	American Journal of Kidney Diseases.2025; 85 (1): 45-54.e1.	Data insufficient to calculate effect estimate
836	Gao S, et al	2025	Association of air pollutants exposure and increased risk of peritoneal dialysis-related peritonitis: An observational study from PDTAP cohort.	Peritoneal Dialysis International.2025.	Data insufficient to calculate effect estimate
837	Hmaidouch N, et al	2025	Peritoneal Dialysis in Morocco: Nineteen Years of Experience from the First National Center and Evaluation of Technique Survival and Mortality.	Epidemiology and Health System Journal.2025; 12 (3): 114-119.	Unclear definition of PD-associated peritonitis and data insufficient to calculate effect estimate
838	Hopkins J, et al	2025	Trends in peritoneal dialysis demographics, peritonitis and HD transfer in Australia from 2013–2022.	Peritoneal Dialysis International.2025.	Data insufficient to calculate effect estimate
839	Hori S, et al	2025	Current position of Moncrief and Popovich's technique in patients on peritoneal dialysis: who really benefits from this technique from the perspective of mortality and peritonitis development?	Clinical & Experimental Nephrology.2025; 29 (12): 1871-1880.	Data insufficient to calculate effect estimate
840	Huang H, et al	2025	Factors influencing the symptom-to-contact time in patients with peritoneal dialysis-associated peritonitis: a multi-center retrospective study.	BMC Nephrology.2025; 26 (1): 517.	Data insufficient to calculate effect estimate
841	Kalla S, et al	2025	Hypokalaemia and peritoneal dialysis-related peritonitis: Association, risk factors and outcomes.	Peritoneal Dialysis International.2025.	Data insufficient to calculate effect estimate
842	Khandelwal P, et al	2025	Early-Start Versus Late-Start Icodextrin for Children Receiving Chronic Peritoneal Dialysis.	Clinical Journal of the American Society of Nephrology.2025; 20 (12): 1729-1743.	Data insufficient to calculate effect estimate
843	Kim DE, et al	2025	Impact of glycemic control on residual kidney function and technique failure associated with volume overload in diabetic patients on peritoneal dialysis.	Kidney Research and Clinical Practice.2025; 44 (3): 481-490.	Specific population (diabetic patients)
844	Kim K, et al	2025	Clinical effects of a home care program for patients with peritoneal dialysis in a tertiary care hospital.	Kidney Research and Clinical Practice.2025; 44 (3): 510-521.	Unclear definition of PD-associated peritonitis and data insufficient to calculate effect estimate
845	Kim SM, et al	2025	Exploring the Possibility of Medical Device Surveillance in Patients on Peritoneal Dialysis Using a Common Data Model.	Medicina.2025; 61 (5): 28.	Not relevant in the context of PD-associated peritonitis
846	Kinnunen S, et al	2025	Time Trends and Causes of Infection-Related Mortality Among Patients Starting Dialysis in Finland: A Nationwide Cohort Study.	Kidney Medicine.2025; 7 (6).	No outcome reported (incidence data [no. of event and time-at-risk])
847	Knapp C, et al	2025	Peritoneal Dialysis-Associated Peritonitis Rates in the Outpatient and Hospital Setting Among Incident Dialysis Patients With Medicare, 2009–2018.	Kidney Medicine.2025; 7 (1).	No outcome reported (incidence data [no. of event and time-at-risk])
848	Kunin M, et al	2025	Acute asymptomatic C-reactive protein rise predicts short-term adverse events in peritoneal dialysis patients.	American Journal of the Medical Sciences.2025; 370 (6): 529-534.	Unclear definition of PD-associated peritonitis and data insufficient to calculate effect estimate
849	Kunin M, et al	2025	Fungal peritonitis in peritoneal dialysis patients: a nationwide cohort study.	Eur J Clin Microbiol Infect Dis. 2025 Dec;44(12):2943-2953.	Unclear definition of PD-associated peritonitis and data insufficient to calculate effect estimate
850	Lan J, et al	2025	Association Between Atherogenic Index of Plasma and Clinical Outcomes in Peritoneal Dialysis Population.	Journal of Clinical Medicine.2025; 14 (14).	Data insufficient to calculate effect estimate
851	Lee CC, et al	2025	Longitudinal Changes in Peritoneal Transport and Their Impact on Dialysis Outcomes: A Machine Learning Approach Integrating Clinical and Biomarker Data.	American Journal of Nephrology.2025; 56 (6): 688-701.	No outcome reported (incidence data [no. of event and time-at-risk])
852	Lee JY, et al	2025	Immediate-start peritoneal dialysis without break-in-period: an 18-year retrospective cohort study on patient survival.	Kidney Research and Clinical Practice.2025; 44 (2): 338-348.	Data insufficient to calculate effect estimate
853	Li J, et al	2025	Dynamic changes in serum albumin and ferritin indicate higher risk of early-onset peritonitis in peritoneal dialysis patients.	Ther Apher Dial. 2025 Jan 2. doi: 10.1111/1744-9987.14244.	Follow-up time or study period less than 1 year

Table S3 Excluded Studies (Continued)

List	Source	Year	Title	Journal	Reason for Exclusion
854	Li L, et al	2025	Lower time-averaged serum uric acid was associated with increased mortality in peritoneal dialysis patients.	Ther Apher Dial. 2025 Jun;29(3):479-490.	No outcome reported (incidence data [no. of event and time-at-risk])
855	Liu M, et al	2025	Establishment of a prediction model for long-term infections in patients undergoing peritoneal dialysis.	Frontiers in Medicine.2025; 12.	Unclear definition of PD-associated peritonitis and data insufficient to calculate effect estimate
856	Liu Y, et al	2025	Peritoneal dialysis related peritonitis caused by commensal neisseria species: a retrospective case series.	BMC Nephrology.2025; 26 (1): 313.	Specific organism (Neisseria species)
857	Liu Y, et al	2025	Hierarchical management and precision intervention reduce peritonitis and improve hand hygiene in diabetic nephropathy peritoneal dialysis patients.	Scientific Reports.2025; 15 (1): 44976.	Specific population (diabetic patients)
858	Liu Y, et al	2025	Diagnostic value of NLR, PLR and SIRI in peritoneal dialysis-associated peritonitis.	American Journal of Translational Research.2025; 17 (3): 2250-2257.	Data insufficient to calculate effect estimate
859	Ljungquist O, et al	2025	Modifiable risk factors for peritoneal dialysis-related infections - a population-based cohort study on risk factors and outcomes in South Sweden.	Frontiers in Nephrology.2025; 5.	Duplicate population with Tobijaszewska M, et al (2024)
860	Lwin KZL, et al	2025	Factors influencing diagnostic imaging in peritoneal dialysis-associated peritonitis.	Peritoneal Dialysis International.2025.	Data insufficient to calculate effect estimate
861	Maxia S, et al	2025	Controversial Aspects of Peritoneal Dialysis in Italy. Results of the First National Audit of PD.	Giornale Italiano di Nefrologia.2025; 42 (5): 24.	Non-English language (article in Italian)
862	Mitsuno R, et al	2025	Intraabdominal fat-free volume, but not fat volume, predicts the development of peritonitis in patients on peritoneal dialysis: a retrospective cohort study.	Renal Replacement Therapy.2025; 11 (1): 1-10.	Data insufficient to calculate effect estimate
863	Mitsuno R, et al	2025	Early Initiation of Icodextrin Reduces Peritoneal Dialysis-Associated Peritonitis Risk: A Retrospective Cohort Study.	Blood Purification.2025; 54 (3): 174-183.	Small sample size (less than 100 patients)
864	Munairdjy Debeh FG, et al	2025	Multicenter Insights into Peritoneal Dialysis for Autosomal Dominant Polycystic Kidney Disease.	Kidney360. 2025 Nov 1;6(11):1918-1927.	Specific population (ADPKD patients)
865	Natejumnong C, et al	2025	The Outcome and Return to Peritoneal Dialysis Following Fungal Peritonitis: A 9-Year Experience at Uttaradit Hospital, Thailand.	Journal of the Medical Association of Thailand.2025; 108 (11): 863-869.	Specific organism (fungal peritonitis)
866	Nochaiwong S, et al	2025	Association Between Peritoneal Dialysis-Associated Peritonitis and the Risk of All-Cause Mortality and Cardiovascular Death: A Time-Matched Retrospective Cohort Study.	Medical Sciences.2025; 13 (4): 30.	No outcome reported (incidence data [no. of event and time-at-risk])
867	Núñez-Delgado S, et al	2025	Long-Term Impact of Peritoneal Dialysis Ultrafiltration on Cardiorenal Patients.	CardioRenal Medicine.2025; 15 (1): 560-571.	No outcome reported (incidence data [no. of event and time-at-risk])
868	Nyabera A, et al	2025	Peritoneal dialysis in patients with severe obesity: A successful single center experience.	Peritoneal Dialysis International.2025; 45 (5): 298-302.	Small sample size (less than 100 patients)
869	Othman NIE, et al	2025	Peritoneal Dialysis in Brunei Darussalam: A Historical Review and Survival Analysis (1993-2020).	Brunei International Medical Journal (BIMJ).2025; 21 34-41.	No outcome reported (incidence data [no. of event and time-at-risk])
870	Özdemir YE, et al	2025	Clinical Outcomes and Microbiological Profiles of Patients with Culture-Confirmed Peritonitis.	Infectious Diseases and Clinical Microbiology.2025; 7 (1): 88-96.	Not population of interest
871	Pak WLW, et al	2025	Diagnostic accuracy and clinical value of polymerase chain reaction tests for Mycobacterium tuberculosis in peritoneal dialysis effluent: A 20-year single-centre retrospective study.	Peritoneal Dialysis International.2025.	Specific organism (M. tuberculosis)
872	Pan L, et al	2025	Risk Factors for Peritoneal Dialysis-Associated Peritonitis Due to Home-Based Operation Management: A Retrospective Cohort Study.	Journal of Clinical Nursing.2025; 34 (9): 3848-3856.	Data insufficient to calculate effect estimate
873	Poulain L, et al	2025	Early-Onset Peritonitis and Outcomes of Peritoneal Dialysis: A Cohort Study with Data from the RDPLF.	American Journal of Nephrology.2025; 56 (4): 391-402.	Data insufficient to calculate effect estimate

Table S3 Excluded Studies (Continued)

List	Source	Year	Title	Journal	Reason for Exclusion
874	Qiao Y, et al	2025	The association between empirical antibiotic regimens and the outcome of peritoneal dialysis-related peritonitis: a multi-center, large-scale cohort study.	Journal of Nephrology.2025; 38 (9): 2673-2685.	Data insufficient to calculate effect estimate
875	Qiu W, et al	2025	Effectiveness of CONUT and NRI as nutritional risk screening tools in peritoneal dialysis: a multicenter study.	Frontiers in Nutrition.2025; 12.	No outcome reported (incidence data [no. of event and time-at-risk])
876	Richards K and Vilayur E	2025	Proton pump inhibitors and peritoneal dialysis-associated peritonitis: a cohort study in Newcastle, Australia.	Internal Medicine Journal.2025; 55 (9): 1524-1531.	Specific organism (only first episodes)
877	Ruzic A, et al	2025	Peritoneal dialysis catheter in renal failure - a retrospective study.	Tidsskrift for Den Norske Laegeforening.2025; 145 (14): 25.	Data insufficient to calculate effect estimate
878	Ryou S, et al	2025	Successful management of catheter injury or refractory infection by partial replantation of peritoneal dialysis catheters: a retrospective observational study.	BMC Nephrology.2025; 26 (1): 52.	No outcome reported (incidence data [no. of event and time-at-risk])
879	Ryuzaki M, et al	2025	Patient education status regarding peritoneal dialysis: a questionnaire-based survey study.	Renal Replacement Therapy.2025; 11 (1).	Not relevant in the context of PD-associated peritonitis
880	Sabanayagam D, et al	2025	Association of Sex With All-Cause and Cause-Specific Peritoneal Dialysis Discontinuation.	American Journal of Kidney Diseases.2025; 86 (4): 475-486.e1.	No outcome reported (incidence data [no. of event and time-at-risk])
881	Sevignani G, et al	2025	Comparison of three strategies (urgent vs. early vs. planned-start) of peritoneal dialysis initiation in kidney failure patients requiring dialysis.	Peritoneal Dialysis International.2025.	Follow-up time or study period less than 1 year
882	Sikhipha TB, et al	2025	Continuous ambulatory peritoneal dialysis technique failure in adult patients treated at a tertiary hospital in central South Africa: a retrospective analytical study.	BMC Nephrology.2025; 26 (1).	Data insufficient to calculate effect estimate
883	Sun C, et al	2025	Analysis of causes and risk factors for early withdrawal from peritoneal dialysis therapy and development of a prediction nomogram.	Clinical Nephrology.2025; 104 (1): 23-33.	No outcome reported (incidence data [no. of event and time-at-risk])
884	Suppadungsuk S, et al	2025	Comparative clinical and economic outcomes of peritoneal dialysis in urban teaching, urban non-teaching, and rural hospitals in the United States: a nationwide analysis from the National Inpatient Sample.	Hosp Pract (1995).2025; 53 (1): 2593813.	Duplicate population with Thongprayoon C, et al (2025)
885	Teitelbaum I, et al	2025	Associations Between Serum Sodium, Peritoneal Dialysis-Associated Peritonitis, and Mortality in the Peritoneal Dialysis Outcomes and Practice Patterns Study (PDOPPS).	American Journal of Kidney Diseases.2025; 86 (1): 84-96.e1.	Data insufficient to calculate effect estimate
886	Thongprayoon C, et al	2025	Racial disparities in in-hospital outcomes and costs among U.S. patients on peritoneal dialysis: a 15-year national cohort study.	Sci Rep.2025; 16 (1): 2426.	Data insufficient to calculate effect estimate
887	Virtanen J, et al	2025	Survival, cumulative hospital days and infectious complications in urgent-start PD compared with urgent-start HD.	Peritoneal Dialysis International.2025; 45 (4): 224-233.	Small sample size (less than 100 patients)
888	Wan S, et al	2025	Prognostic value of the triglyceride-glucose index for continuous ambulatory peritoneal dialysis patients.	BMC Nephrology.2025; 26 (1): 356.	Data insufficient to calculate effect estimate
889	Wang L, et al	2025	Impact of roxadustat on anemia management in infected patients undergoing long-term dialysis: a retrospective cohort analysis.	Frontiers in Pharmacology.2025; 16.	Not relevant in the context of PD-associated peritonitis
890	Wang L, et al	2025	The association between changes in muscle mass and function and mortality in individuals receiving peritoneal dialysis.	Peritoneal Dialysis International.2025; 45 (2): 84-92.	No outcome reported (incidence data [no. of event and time-at-risk])
891	Wang Q, et al	2025	Hospital type and clinical outcomes in peritoneal dialysis: Findings from the PDTAP study.	Peritoneal Dialysis International.2025.	Data insufficient to calculate effect estimate
892	Wang Y, et al	2025	Association between prealbumin levels and pulmonary hypertension in peritoneal dialysis patients: a single-center retrospective cohort study.	BMC Nephrology.2025; 26 (1).	No outcome reported (incidence data [no. of event and time-at-risk])

Table S3 Excluded Studies (Continued)

List	Source	Year	Title	Journal	Reason for Exclusion
893	Wang Y, et al	2025	Prognostic outcomes and influencing factors of peritoneal dialysis-related peritonitis: A retrospective single-center study.	Clinical Nephrology.2025; 103 (6): 359-365.	Specific population (only peritonitis cases)
894	Wang Y, et al	2025	A nomogram for predicting the risk of peritoneal dialysis-associated peritonitis in patients with end-stage renal disease undergoing peritoneal dialysis: model development and validation study.	BMC Nephrology.2025; 26 (1): 248.	Data insufficient to calculate effect estimate
895	Xie J, et al	2025	Association between serum magnesium level at catheter insertion and peritoneal dialysis technique failure in peritoneal dialysis patients.	Chinese Journal of Nephrology.2025; 41 (11): 841-848.	Non-English language (article in Chinese)
896	Xu L, et al	2025	Impact of diabetes on survival and clinical outcomes in elderly patients receiving peritoneal dialysis.	Renal Failure.2025; 47 (1): 2589586.	Data insufficient to calculate effect estimate
897	Xu QJ, et al	2025	Unveiling risk factors: a prognostic model of frequent peritonitis in peritoneal dialysis patients.	Frontiers in Medicine.2025; 12.	Data insufficient to calculate effect estimate
898	Yamagishi I, et al	2025	Species-specific outcomes and role of infectious disease consultation in peritoneal dialysis infections caused by nontuberculous mycobacteria: A 10-year retrospective analysis.	Peritoneal Dialysis International.2025.	Specific organism (NTM)
899	Yan Q, et al	2025	Relationship between glucose to lymphocyte ratio and the first peritonitis episode in patients treated with peritoneal dialysis.	Scientific Reports.2025; 15 (1): 6834.	Data insufficient to calculate effect estimate
900	Yao J, et al	2025	Risk Factors and a Nomogram Construction for Prolonged Length of Hospital Stay in Patients With Peritoneal Dialysis Associated Peritonitis.	Chung-Kuo i Hsueh Ko Hsueh Yuan Hsueh Pao Acta Academiae Medicinae Sinicae.2025; 47 (2): 244-250.	Non-English language (article in Chinese)
901	Yaxley J, et al	2025	Peritoneal dialysis catheterization with an upward tunnel and exit site: An observational study.	Journal of Vascular Access.2025; 26 (4): 1318-1323.	Not relevant in the context of PD-associated peritonitis
902	Zeng Y, et al	2025	Effectiveness of a smart management system in improving adherence and clinical outcomes of patients receiving peritoneal dialysis: a retrospective cohort analysis.	BMC Nursing.2025; 24 (1).	No outcome reported (incidence data [no. of event and time-at-risk])
903	Zhang L, et al	2025	Elevated Monocyte-to-Lymphocyte Ratio at Peritoneal Dialysis Initiation Predicts Long-Term Peritonitis Risk: A Retrospective Cohort Study.	Blood Purification.2025; 54 (9-10): 554-564.	Data insufficient to calculate effect estimate
904	Zhang M, et al	2025	Epidemiology, drug resistance, and clinical risk factors of peritoneal dialysis-associated peritonitis: a five-year multicenter study.	Frontiers in Cellular & Infection Microbiology.2025; 15 1654246.	Data insufficient to calculate effect estimate
905	Zhou H, et al	2025	Machine learning-based prediction of early-onset peritoneal dialysis-associated peritonitis: the role of the CONUT score.	Frontiers in Nutrition.2025; 12.	Data insufficient to calculate effect estimate
906	Zhou M, et al	2025	The SII-PNI score: A novel composite biomarker for personalized mortality risk prediction in peritoneal dialysis patients.	PLoS ONE [Electronic Resource].2025; 20 (12): e0338086.	No outcome reported (incidence data [no. of event and time-at-risk])
907	Béchade C, et al	2026	Effect of the centre volume on time on peritoneal dialysis therapy: A cause-specific analysis with the retrospective data from the RDPLF.	Peritoneal Dialysis International.2026.	No outcome reported (incidence data [no. of event and time-at-risk])
908	Beckwith H, et al	2026	Epidemiology of peritoneal dialysis exit site infections: Does sex play a role?	Peritoneal Dialysis International.2026; 46 (3): 261-264.	No outcome reported (incidence data [no. of event and time-at-risk])
909	Chan CE, et al	2026	Ultrasound-Guided Percutaneous Peritoneal Dialysis Catheter Insertion With Musculofascial Tunnelling Reduces Catheter Migration-Observational Retrospective Cohort Study.	Nephrology (Carlton).2026; 31 (3): e70180.	No outcome reported (incidence data [no. of event and time-at-risk])
910	Chan GCK, et al	2026	Zinc alpha-2-glycoprotein in peritoneal dialysis effluent correlates with peritoneal transport characteristics, peritonitis rate, and outcome.	Kidney Research and Clinical Practice.2026; 45 (3): 393-403.	Duplicate population with Szeto CC, et al (2022)
911	Chao ACW, et al	2026	Outcome of Peritonitis in Automated Peritoneal Dialysis: A Cohort Study.	Kidney Medicine.2026; 8 (1).	Data insufficient to calculate effect estimate

Table S3 Excluded Studies (Continued)

List	Source	Year	Title	Journal	Reason for Exclusion
912	Cheikh Hassan HI, et al	2026	Back-up arteriovenous fistula in peritoneal dialysis patients: a retrospective cohort study of long-term meaningful outcomes.	Internal Medicine Journal.2026.	Data insufficient to calculate effect estimate
913	Chen FY, et al	2026	Far-infrared therapy improves cardiovascular and infectious outcomes in peritoneal dialysis patients.	Journal of the Formosan Medical Association.2026; 125 (1): 30-36.	Small sample size (less than 100 patients)
914	Chen S, et al	2026	Development and validation of a nomogram prediction model for treatment failure of peritoneal dialysis-associated peritonitis.	Chinese Journal of Nephrology.2026; 42 (1): 1-9.	Non-English language (article in Chinese)
915	Chen XY, et al	2026	Value of different nutritional and inflammatory markers for diagnosis of peritonitis in patients undergoing peritoneal dialysis.	Renal Failure.2026; 48 (1): 2650574.	Data insufficient to calculate effect estimate
916	Chen YA, et al	2026	Comparison of outcomes between kidney transplant-naive and post-transplant graft failure peritoneal dialysis patients.	Journal of the Formosan Medical Association.2026; 125 (4): 449-453.	Data insufficient to calculate effect estimate
917	Davenport A	2026	Does lower dietary protein intake result in lower muscle mass in patients with end-stage kidney failure treated by peritoneal dialysis? A retrospective study.	Nutrition in Clinical Practice.2026; 41 (2): 530-539.	No outcome reported (incidence data [no. of event and time-at-risk])
918	Goh JX, et al	2026	Effect of Renin-Angiotensin System Inhibition on Residual Kidney Function in Peritoneal Dialysis.	Medicina.2026; 62 (2): 30.	Unclear definition of PD-associated peritonitis and data insufficient to calculate effect estimate
919	Hammer V, et al	2026	Retrospective review of assisted peritoneal dialysis: The Alberta, Canada experience.	Peritoneal Dialysis International.2026; 46 (2): 124-129.	Unclear definition of PD-associated peritonitis and data insufficient to calculate effect estimate
920	Hayat A, et al	2026	Incremental start and clinical outcomes in peritoneal dialysis: International results from PDOPPS.	Peritoneal Dialysis International.2026; 46 (2): 163-173.	Data insufficient to calculate effect estimate
921	Houle O, et al	2026	The Effect of Obesity on Outcomes of Percutaneous Peritoneal Dialysis Catheter Insertion by Interventional Radiologists.	Journal of Vascular and Interventional Radiology.2026; 37 (3).	Unclear definition of PD-associated peritonitis and data insufficient to calculate effect estimate
922	Htay H, et al	2026	Optimizing exit-site care for hospitalized peritoneal dialysis patients with weekly chlorhexidine-impregnated dressing.	Peritoneal Dialysis International.2026; 46 (3): 265-269.	No outcome reported (incidence data [no. of event and time-at-risk])
923	Ingham I, et al	2026	Peritoneal dialysis-related peritonitis due to Pasteurella multocida in Australia.	Perit Dial Int. 2026 Jan;46(1):40-44.	Specific organism (P. multocida)
924	Khachroumi N, et al	2026	Outcomes of diabetic patients undergoing peritoneal dialysis in the French Language Peritoneal Dialysis Registry.	Clinical Kidney Journal.2026; 19 (1).	No outcome reported (incidence data [no. of event and time-at-risk]) and data insufficient to calculate effect estimate
925	Kraft L, et al	2026	Changing microbiology and outcomes of PD-associated peritonitis over four decades.	Clinical Kidney Journal.2026; 19 (1).	Data insufficient to calculate effect estimate
926	Kuo PH, et al	2026	Risk Factors for Peritonitis in Patients With Systemic Lupus Erythematosus Undergoing Peritoneal Dialysis.	International Journal of Rheumatic Diseases.2026; 29 (3): e70597.	Specific population (SLE)
927	Liu R, et al	2026	The feasibility of incremental peritoneal dialysis for older patients on peritoneal dialysis.	Kidney Research and Clinical Practice.2026; 45 (3): 426-437.	Data insufficient to calculate effect estimate
928	Liu Y, et al	2026	Risk factors and microbiological characteristics of bacterial peritonitis in patients undergoing peritoneal dialysis.	BMC Nephrology.2026; 27 (1).	Data insufficient to calculate effect estimate
929	Mai J, et al	2026	Association of high-density lipoprotein cholesterol lipid with peritoneal dialysis related peritonitis.	BMC Nephrology.2026; 27 (1).	Data insufficient to calculate effect estimate
930	Nagashima A, et al	2026	Facility-based educational systems and peritonitis incidence in peritoneal dialysis: findings from a nationwide survey in Japan.	Clinical & Experimental Nephrology.2026; 30 (5): 780-791.	Data insufficient to calculate effect estimate
931	Niu L, et al	2026	Development and validation of a nomogram for predicting treatment failure in culture-negative peritoneal dialysis–associated peritonitis.	Clinical Kidney Journal.2026; 19 (2).	Data insufficient to calculate effect estimate
932	Parapiboon W, et al	2026	Incremental peritoneal dialysis with days off vs standard-dose peritoneal dialysis: a propensity-matched study (PD holiday).	International Urology and Nephrology.2026.	Small sample size (less than 100 patients)

Table S3 Excluded Studies (Continued)

List	Source	Year	Title	Journal	Reason for Exclusion
933	Shang S, et al	2026	The synergistic effect of diabetes mellitus and low high-density lipoprotein cholesterol on the prediction of pneumonia in peritoneal dialysis patients: a multicenter retrospective study.	Frontiers in Nutrition.2026; 12.	No outcome reported (incidence data [no. of event and time-at-risk])
934	Sharma M, et al	2026	Peritonitis in Continuous Ambulatory Peritoneal Dialysis: A One-Year Prospective Case Series on Microbiology and Outcomes.	International Journal of Medical Science and Advanced Clinical Research (IJMACR).2026; 9 (2): 63-66.	Small sample size (less than 100 patients)
935	Spagnol R, et al	2026	Hospitalization burden in children on dialysis: insights from the Italian Registry of Pediatric Chronic Dialysis (IRPCD).	Pediatric Nephrology.2026; 41 (6): 1785-1795.	Unclear definition of PD-associated peritonitis and data insufficient to calculate effect estimate
936	Tang W, et al	2026	Association between serum potassium levels and peritonitis in peritoneal dialysis patients: a longitudinal study.	BMC Nephrology.2026; 27 (1).	Data insufficient to calculate effect estimate
937	Thangavel J, et al	2026	Survival Outcomes and Mortality Risk Factors in Peritoneal Dialysis.	Indian Journal of Nephrology.2026; 36 (3): 314-320.	No outcome reported (incidence data [no. of event and time-at-risk])
938	Urate S, et al	2026	Exposure to Shower Water Without Dressings Is Associated With Increased Risk of Catheter-Related Infection in Peritoneal Dialysis Patients: A Retrospective Cohort Study.	Ther Apher Dial. 2026 Jun;30(3):397-402.	No outcome reported (incidence data [no. of event and time-at-risk])
939	Wang IK, et al	2026	The Impact of Balance Solution on the Outcomes of Incident Peritoneal Dialysis Patients.	Pharmacoepidemiology & Drug Safety.2026; 35 (2): e70325.	Unclear definition of PD-associated peritonitis and data insufficient to calculate effect estimate
940	Wang L, et al	2026	Serum 25-Hydroxyvitamin D Levels and Longitudinal Changes in Muscle Mass and Function in Peritoneal Dialysis Patients: A Prospective Cohort Study.	Journal of Nutritional Science and Vitaminology.2026; 72 (1): 1-8.	No outcome reported (incidence data [no. of event and time-at-risk])
941	Wang Q, et al	2026	Prediction of Imminent Peritoneal Dialysis-Associated Peritonitis Using Time-Updated Electronic Health Records and Machine Learning: A Temporal Validation Study.	Journal of Inflammation Research.2026; 19.	Unclear definition of PD-associated peritonitis and data insufficient to calculate effect estimate
942	Wu SF, et al	2026	Effect of common salt on granulomas at exit sites of peritoneal dialysis catheters: A prospective study.	Peritoneal Dialysis International.2026.	Experimental studies
943	Xie J, et al	2026	Serum bilirubin concentrations in peritoneal dialysis patients are correlated with the initial onset of peritonitis.	BMC Nephrology.2026; 27 (1).	Data insufficient to calculate effect estimate
944	Xin Y, et al	2026	Peritoneal dialysis therapy is a viable dialysis option for anuric patients: A retrospective cohort study.	Peritoneal Dialysis International.2026.	No outcome reported (incidence data [no. of event and time-at-risk])
945	Xu L, et al	2026	Body roundness Index on the clinical outcome of peritoneal dialysis patients: Analysis of two cohorts.	Peritoneal Dialysis International.2026.	Unclear definition of PD-associated peritonitis and data insufficient to calculate effect estimate
946	Xu L, et al	2026	Prognostic Significance of Baseline Lean Tissue Mass Percentage in Incident Peritoneal Dialysis Patients.	Kidney Medicine.2026; 8 (3).	Unclear definition of PD-associated peritonitis and data insufficient to calculate effect estimate
947	Xu L, et al	2026	Intramuscular fat content and the risk of peritonitis in peritoneal dialysis patients.	Clinical Kidney Journal.2026; 19 (4).	Small sample size (less than 100 patients)
948	Zheng X, et al	2026	Risk factors and outcomes of culture-negative peritonitis in peritoneal dialysis: 10 years' insight from a large center in northern China.	BMC Nephrology.2026; 27 (1).	Data insufficient to calculate effect estimate
949	Zheng Y, et al	2026	Change trajectory of fluid load management behavior ability in peritoneal dialysis patients and its association with physical activity.	Frontiers in Medicine.2026; 12.	No outcome reported (incidence data [no. of event and time-at-risk])

Table S4 Description of the 153 Unique Study Populations of PD Patients Included in Meta-Analysis

Source, Year	Country	Study Setting	Data Collection: Study Period	Total Sample Size: No. PD Center	Study Population: Mean Age, Year	Male, %	PD Modality
Balasubramanian et al, 2011[1]	United Kingdom	The Royal London and St Bartholomew's Hospitals, London	Retrospective: Jan 1, 2003, to Jan 1, 2008	372: 1	Adult: 54.4	61.8	Mixed modalities
Brown et al, 2011[2]	United Kingdom	Scottish Renal Registry	Registry data: Jan 1, 2000, to Dec 31, 2007	Not specified: 10	Adult: 60.0	60	Mixed modalities
Davenport et al, 2011[3]	United Kingdom	The Pan Thames Renal Audit (Pan Themes area and South Eastern England)	Registry data: Period 1 (Jan 1, 2002, to Dec 31, 2003); period 2 (Jan 1, 2005, to Dec 31, 2008)	4095: 12	Not specified: Not specified	Not specified	Not specified
Huang et al, 2011[4]	Taiwan	Taichung Veterans General Hospital	Retrospective: Jan 1, 1984, to June 1, 2010	630: 1	Not specified: 47.4	49.7	CAPD
Kanjanabuch et al, 2011[5]	Thailand	Nationwide PD centers in Thailand	Retrospective: Oct 1, 2009, to Sep 30, 2010	8201: 96	Not specified: Not specified	Not specified	Mixed modalities
Liawnoraset, 2011[6]	Thailand	Maharat Nakhon Ratchasima Hospital, Nakhon Ratchasima	Retrospective: Jan 1, 2008, to Dec 31, 2010	318: 1	Mixed population: 49.9	50.9	CAPD
Luvira et al, 2011[7]	Thailand	Phramongkutklao Hospital and College of Medicine, Bangkok	Retrospective: Jan 1, 1983, to Dec 31, 2007	333: 1	Adult: 57.2	61.5	CAPD
Martin et al, 2011[8]	Brazil	BRAZPD database, a Brazilian Cohort	Registry data: Dec 31, 2004, to Oct 31, 2007	2032: 114	Adult: Not specified	46.4	Mixed modalities
Muñoz de Bustillo et al, 2011[9]	Spain	Levante registry, the public sector in the 3 provinces of the Community of Valencia (Alicante, Castellon, and Valencia) and Murcia, and the provinces of Albacete and Cuenca	Registry data: Jan 1, 1993, to Dec 31, 2005	1515: 18	Not specified: 52.9	55.7	Mixed modalities
Oygar et al, 2011[10]	Türkiye	Istanbul University Cerrahpasa Medical Faculty, Istanbul	Retrospective: Not specified	109: 1	Not specified: 43.4	61.5	Mixed modalities
Quintanar Lartundo et al, 2011[11]	Spain	University Hospital Marqués de Valdecilla, Santander	Retrospective: Jan 1, 2004, to Dec 31, 2009	411: 1	Adult: 64.9	71.6	Mixed modalities
Topanthanont et al, 2011[12]	Thailand	Renal Unit, Faculty of Medicine, Ramathibodi Hospital	Retrospective: Jan 1, 2000, to Dec 31, 2010	103: 1	Not specified: 56.19	38.8	Mixed modalities
Aksu et al, 2012[13]	Türkiye	Izmir Tepecik Training and Research Hospital	Retrospective: Nov 1, 1995, to Nov 30, 2008	110: 1	Pediatric: 9.8	46.4	Mixed modalities
Barone et al, 2012[14]	Argentina	Servicio de Terapia Renal Hurlingham SRL, Buenos Aires	Retrospective: Aug 4, 1995, to Mar 31, 2011	118: 1	Mixed population: 51.9	37.3	Mixed modalities
Basturk et al, 2012[15]	Türkiye	Sisli Etfal Research and Education Hospital, Istanbul	Retrospective: Jun 1, 2001, to Dec 31, 2011	355: 1	Adult: Not specified	Not specified	Mixed modalities

Abbreviations: APD, automated peritoneal dialysis; CAPD, continuous ambulatory peritoneal dialysis; PD, peritoneal dialysis.

Table S4 Description of the 153 Unique Study Populations of PD Patients Included in Meta-Analysis (Continued)

Source, Year	Country	Study Setting	Data Collection: Study Period	Total Sample Size: No. PD Center	Study Population: Mean Age, Year	Male, %	PD Modality
Chen et al, 2012[16]	Canada/United States	North American Pediatric Renal Trials and Collaborative Studies (NAPRTCS) database: Canada and United States	Registry data: Jan 1, 1992, to Dec 31, 2007	2829: 109	Pediatric: Not specified	52.9	Not specified
Levallois et al, 2012[17]	Canada	Hôpital Maisonneuve-Rosemont, Université de Montréal	Retrospective: Aug 1, 1996, to Jul 31, 2006	288: 1	Adult: Not specified	Not specified	Not specified
Nayak et al, 2012[18]	India	Lazarus Hospital, Hyderabad	Retrospective: May 1, 2003, to Apr 30, 2008	246: 1	Adult: 51.9	69.1	Not specified
Oliveira et al, 2012[19]	Brazil	Universidade Estadual Paulista, Botucatu, São Paulo	Retrospective: Jan 1, 2004, to Dec 31, 2009	189: 1	Not specified: 53.3	52.1	Mixed modalities
Pierce et al, 2012[20]	United States	Wake Forest Baptist Health and the affiliated outpatients PD units of the Wake Forest University Outpatients Dialysis centers	Retrospective: May 1, 2004, to Jun 30, 2009	377: 18	Adult: 52.0	45.4	Not specified
Rocha et al, 2012[21]	Portugal	Hospital Santo António in Porto	Prospective: Oct 1, 1985, to June 30, 2010	438: 1	Adult: 48.1	38.4	Mixed modalities
Su et al, 2012[22]	China	Peking University Third Hospital, Beijing	Prospective: Dec 31, 2008, to Dec 31, 2010	158: 1	Not specified: 62.5	51.3	CAPD
Xu et al, 2012[23]	China	Peking University First Hospital, Beijing	Prospective: Jan 1, 2008, to Apr 30, 2011	124: 1	Adult	Not specified	CAPD
Xu et al, 2012[24]	China	Peking University First Hospital, Beijing	Prospective: Jul 1, 2002, to Feb 28, 2007	313: 1	Adult: 61.1	42.5	CAPD
Yap et al, 2012[25]	Hong Kong	Tung Wah Hospital, University of Hong Kong	Retrospective: Jan 1, 1995, to Dec 31, 2010	296: 1	Adult: 63.9	50.3	Not specified
Yap et al, 2012[26]	Hong Kong	Tung Wah Hospital and Queen Mary Hospital, University of Hong Kong	Retrospective: Jan 1, 2000, to Sep 30, 2010	1285: 2	Adult: Not specified	Not specified	Not specified
Chern et al, 2013[27]	Taiwan	Kaohsiung Chang Gung Memorial Hospital and Chang Gung University College of Medicine	Retrospective: Feb 1, 1999, to Jul 31, 2010	404: 1	Adult: 47.1	42.3	Mixed modalities
Huang et al, 2013[28]	Taiwan	National Taiwan University College of Medicine and Hospital	Prospective: Jan 1, 2009, to Feb 29, 2012	183: 1	Adult: 53.0	45.9	Not specified
Kerschbaum et al, 2013[29]	Austria	Medical University Innsburuck; Medical University Vienna; Academic Teaching Hospital Feldkirch; Klinikum Wels-Grieskirchen; A.ö. Krankenhaus St. Pölten; Wilhelminenspital; Krankenhaus der Elisabethinen	Retrospective: Jan 1, 2006, to Dec 31, 2009	726: 7	Mixed population: 55.7	58.8	CAPD
Nessim et al, 2013[30]	Canada	Manitoba (PD-MAN) database	Retrospective: Jan 1, 1997, to Dec 31, 2007	938: 2	Adult: 59.5	55.0	Mixed modalities
Özbalci et al, 2013[31]	Türkiye	Süleyman Demirel University School of Medicine, Isparta	Retrospective: Jan 1, 1998, to Dec 31, 2009	153: 1	Adult: 50.8	49.0	Mixed modalities

Abbreviations: APD, automated peritoneal dialysis; CAPD, continuous ambulatory peritoneal dialysis; PD, peritoneal dialysis.

Table S4 Description of the 153 Unique Study Populations of PD Patients Included in Meta-Analysis (Continued)

Source, Year	Country	Study Setting	Data Collection: Study Period	Total Sample Size: No. PD Center	Study Population: Mean Age, Year	Male, %	PD Modality
Portolés et al, 2013[32]	Spain	Multicenter in the Peritoneal Dialysis Center Group, public hospitals in central Spain	Retrospective: Jan 1, 2003, to Jan 1, 2010	1177: 22	Adult: 54.8	64.4	Mixed modalities
Tsai et al, 2013[33]	Taiwan	Mackay Memorial Hospital, Taiwan	Retrospective: Jan 1, 2001, to Apr 30, 2011	349: 1	Adult: 53.8	41.7	CAPD
Hsieh et al, 2014[34]	Taiwan	Changhua Christian Hospital	Retrospective: Jan 1, 2001, to Dec 31, 2010	391: 1	Adult: 55.8	44.2	CAPD
Isla et al, 2014[35]	South Africa	Polokwane Kidney and Dialysis Center, part of the Pietersburg Provincial Hospital Limpopo	Retrospective: Jan 1, 2008, to Dec 31, 2012	152: 1	Not specified: 36.8	52.0	CAPD
Kumar et al, 2014[36]	India	St. John's Medical College and Hospital, Bengaluru, Karnataka	Retrospective: Jan 1, 2005, to Jan 1, 2012	224: 1	Adult: 51.9	69.2	CAPD
Najafi et al, 2014[37]	Iran	Iranian CAPD registry	Registry data: Jan 1, 1995, to Dec 31, 2010	2239: 36	Not specified: 46.5	45.5	CAPD
Nieto-Ríos et al, 2014[38]	Colombia	Hospital Universitario San Vicente de Paúl	Retrospective: Mar 1, 1981, to Dec 31, 2008	1497: 1	Mixed population: 42.0	48.8	CAPD
Nishina et al, 2014[39]	Japan	Tokai University Hospital, Kanagawa	Retrospective: Apr 1, 2001, to Mar 31, 2011	192: 1	Not specified: 50.3	67.2	Mixed modalities
Núñez-Moral et al, 2014[40]	Spain	Hospital Universitario Central de Asturias, Oviedo	Retrospective: Jan 1, 2007, to Dec 31, 2011	201: 1	Not specified: 58.0	59.2	Mixed modalities
Prasad et al, 2014[41]	India	Sanjay Gandhi Postgraduate Institute of Medical Sciences, Lucknow, India	Prospective: Jan 1, 2002, to Dec 31, 2011	560: 1	Mixed population: 51.3	73.8	CAPD
Chang et al, 2015[42]	South Korea	Yonsei University Severance Hospital, Seoul and NHIS Ilsan Hospital, Goyangshi, Gyeonggi-do	Retrospective: Jan 1, 2000, to Dec 31, 2005	405: 2	Adult: 59.4	53.8	Mixed modalities
Courivaud et al, 2015[43]	France	University Hospital of Besançon	Prospective: Jan 1, 2005, to Dec 31, 2010	240: 1	Adult: 65.0	59.0	Mixed modalities
Figueiredo et al, 2015[44]	Brazil	BRAZPD database, a Brazilian Cohort	Registry data: Jan 1, 2008, to Jan 1, 2011	2243: 122	Adult: 59.4	48.2	Mixed modalities
Lei et al, 2015[45]	China	Ren Ji Hospital, School of Medicine, Shanghai Jiao Tong University	Retrospective: Jan 1, 1995, to Jun 30, 2012	163: 1	Adult: 63.4	57.7	Not specified
Ozisik et al, 2015[46]	Türkiye	Baskent University Hospital, Ankara	Retrospective: Jan 1, 2000, to Dec 31, 2006	440: 1	Not specified: 46.3	54.1	Mixed modalities
Pi et al, 2015[47]	China	Peking University First Hospital and the Second Affiliated Hospital of Harbin Medical University	Prospective: Mar 1, 2011, to May 31, 2013	346: 2	Adult: 59.0	44.8	Not specified
Sanabria et al, 2015[48]	Colombia	The Baxter Inc Renal Therapy Services network, remote PD centers	Retrospective: Jan 1, 2008, to Dec 31, 2009	345: 9	Adult: 53.3	54.2	Mixed modalities
Buttigieg et al, 2016[49]	Malta	Mater Dei Hospital, Malta	Retrospective: Jan 1, 2008, to Dec 31, 2012	137: 1	Adult: 60.0	62.0	Not specified

Abbreviations: APD, automated peritoneal dialysis; CAPD, continuous ambulatory peritoneal dialysis; PD, peritoneal dialysis.

Table S4 Description of the 153 Unique Study Populations of PD Patients Included in Meta-Analysis (Continued)

Source, Year	Country	Study Setting	Data Collection: Study Period	Total Sample Size: No. PD Center	Study Population: Mean Age, Year	Male, %	PD Modality
Duquennoy et al, 2016[50]	Multinational	French Peritoneal Dialysis Registry (RDPLF): France (core contributor), Belgium, Switzerland, Algeria, Morocco, Tunisia, and Luxembourg	Registry data: Jan 1, 2003, to Dec 31, 2012	8396: 64	Adult: 67.2	57.6	Mixed modalities
El-Reshaid et al, 2016[51]	Kuwait	Al-Nafisi Renal Center, Kuwait	Retrospective: Jan 1, 2005, to Dec 31, 2014	308: 1	Adult: 62.0	48.7	Mixed modalities
Higuchi et al, 2016[52]	Japan	Japanese Society for Peritonitis Dialysis database	Retrospective: Jan 1, 2013, to Dec 31, 2013	3042: 114	Mixed population: 65.2	Not specified	Mixed modalities
Kotera et al, 2016[53]	Japan	Mitsui Memorial Hospital, Tokyo	Retrospective: Jan 1, 2002, to Jan 31, 2013	119: 1	Adult: 61.7	65.5	Mixed modalities
Mizuno et al, 2016[54]	Japan	PD centers in Tokai area and affiliated with Nagoya University Renal Replacement Therapy Association	Retrospective: Jan 1, 2005, to Dec 31, 2012	1237: 15	Not specified: 60.8	66.6	Mixed modalities
Nakao et al, 2016[55]	Japan	Jikei University Hospital, Tokyo	Retrospective: Jan 1, 1980, to Dec 31, 2012	527: 1	Not specified: 52.0	76.0	CAPD
Pai et al, 2016[56]	Taiwan	Far Eastern Memorial Hospital, New Taipei City	Retrospective: Jan 1, 2006, to Dec 31, 2012	149: 1	Not specified: 55.6	44.3	Not specified
Pérez-Fontan et al, 2016[57]	Spain	University Hospital A Coruña, A Coruña	Retrospective: Jan 1, 1995, to Dec 31, 2013	691: 1	Mixed population: 59.0	60.2	Not specified
Perl et al, 2016[58]	Canada	The Peritonitis Organism Exit sites Tunnel infections (POET) database (Baxter Healthcare)	Ambispective: Early 1990 to Sep 12, 2005	4247: 25	Not specified: 59.0	55.0	Mixed modalities
Sandrini et al, 2016[59]	Italy	Università di Brescia, Brescia	Prospective: Jan 1, 2002, to Dec 31, 2007	178: 1	Not specified: 60.1	60.0	Mixed modalities
Santhakumaran et al, 2016[60]	United Kingdom	Barts Health NHS Trust, London	Prospective: Jan 1, 2008, to Oct 1, 2012	580: 1	Not specified: 55.8	63.8	Mixed modalities
Shea et al, 2016[61]	Hong Kong	Tung Wah Hospital and Queen Mary Hospital, University of Hong Kong	Prospective: Feb 1, 2011, to July 31, 2013	114: 2	Adult: 59.0	53.5	Mixed modalities
Tian et al, 2016[62]	China	The First Affiliated Hospital of Zhejiang University	Retrospective: Mar 1, 2002, to Jul 31, 2014	294: 1	Adult: 50.8	62.2	CAPD
Alatab et al, 2017[63]	Iran	Shafa center and Shariati hospital, affiliated with Tehran University of Medical Sciences, Tehran	Retrospective: Jan 1, 1995, to Dec 31, 2012	134: 2	Not specified: 48.1	53.0	Not specified
Htay et al, 2017[64]	Singapore	Singapore General Hospital	Retrospective: Jan 1, 2012, to Dec 31, 2015	337: 1	Adult: 61.7	48.7	Mixed modalities
Lee et al, 2017[65]	South Korea	Dong-A University Hospital, Busan	Retrospective: Jan 1, 2007, to Dec 31, 2014	295: 1	Adult: 59.1	59.0	Not specified
Ndlovu et al, 2017[66]	South Africa	King Edward VIII Hospital (KEH) and Inkosi Albert Luthuli Central Hospital	Prospective: Sep 1, 2012, to Feb 28, 2015	140: 2	Adult: 38.1	52.1	CAPD
Ong et al, 2017[67]	Malaysia	Ministry of Health (MOH) dialysis centers	Prospective: Jan 1, 2009, to Dec 31, 2009	1603: 15	Adult: 51.6	47.4	Mixed modalities

Abbreviations: APD, automated peritoneal dialysis; CAPD, continuous ambulatory peritoneal dialysis; PD, peritoneal dialysis.

Table S4 Description of the 153 Unique Study Populations of PD Patients Included in Meta-Analysis (Continued)

Source, Year	Country	Study Setting	Data Collection: Study Period	Total Sample Size: No. PD Center	Study Population: Mean Age, Year	Male, %	PD Modality
Vargas et al, 2017[68]	Colombia	Renal Therapy Services network	Prospective: Jan 1, 2012, to Dec 31, 2013	3525: 49	Adult: Not specified	55.2	Mixed modalities
Al Wakeel et al, 2018[69]	Saudi Arabia	King Khalid University Hospital, Riyad	Retrospective: Jan 1, 2009, to Dec 31, 2013	111: 1	Adult: 47.4	45.1	Mixed modalities
Fang et al, 2018[70]	China	Second Xiangya Hospital of Central South University, Changsha, Hunan	Retrospective: Oct 1, 2014, to Mar 31, 2017	462: 1	Mixed population: 48.7	49.4	CAPD
Hsu et al, 2018[71]	Taiwan	Chang Gung Memorial Hospital, Keelung	Prospective: Jul 1, 2007, to Dec 31, 2016	398: 1	Not specified: 60.4	46.5	Mixed modalities
Ponce et al, 2018[72]	Brazil	BRAZPD II nationwide multicentre cohort	Prospective: Dec 31, 2004, to Jan 31, 2011	491: 75	Pediatric: 10.2	59.1	Mixed modalities
Rope et al, 2018[73]	Sri Lanka	Kandy Teaching Hospital and Girandurukotte CAPD clinic	Prospective: Jul 1, 2016, to Jun 30, 2017	116: 2	Adult: 52.0	62.0	CAPD
Alfayez et al, 2019[74]	Saudi Arabia	King Fahad Medical City	Retrospective: Jan 1, 2006, to Mar 31, 2016	100: 1	Mixed population: 40.7	53.0	Mixed modalities
Balzer et al, 2019[75]	Germany	Hannover Medical School	Retrospective: Jan 1, 2003, to Dec 31, 2017	275: 1	Adult: 56.3	57.8	Mixed modalities
Einbinder et al, 2019[76]	Israel	Meir Medical Center, Kfar Saba	Prospective: Jan 1, 2009, to Dec 31, 2017	201: 1	Not specified: 65.1	74.1	Mixed modalities
Gadola et al, 2019[77]	Uruguay	Universidad de la República, Montevideo	Retrospective: Jan 1, 1999, to Dec 31, 2016	219: 1	Mixed population: 59.3	57.7	Mixed modalities
Hu et al, 2019[78]	China	First Teaching Hospital of Tianjin University of Traditional Chinese Medicine	Retrospective: Jun 1, 2012, to Jun 30, 2017	730: 1	Adult: 58.0	50.4	CAPD
Jahromi et al, 2019[79]	Iran	Emam-Reza Clinic Peritoneal Dialysis Center	Retrospective: Apr 1, 2006, to Apr 30, 2012	235: 1	Mixed population: 48.5	46.4	CAPD
Karthikeyan et al, 2019[80]	India	Sanjay Gandhi Postgraduate Institute of Medical Sciences, Lucknow	Retrospective: Jan 1, 2001, to Dec 31, 2016	120: 1	Adult: 70.3	80.0	Mixed modalities
Lasfar et al, 2019[81]	Tunisia	Sahloul Hospital, Sousse	Retrospective: Jan 1, 2006, to Jun 30, 2016	182: 1	Mixed population: 43.9	59.3	Mixed modalities
Liao et al, 2019[82]	China	Peking University Shenzhen Hospital, Shenzhen; Peking University First Hospital; the Second hospital of Anhui Medical University, Anhui; the Second Affiliated Hospital of Harbin Medical University, Heilongjiand; Handan Central Hospital, Hebei	Prospective: Mar 1, 2013, to May 31, 2016	458: 5	Adult: 51.6	53.1	CAPD
Lichodziejewska-Niemierko et al, 2019[83]	Poland	The PD Working Group of the Polish Society of Nephrology	Retrospective: Jan 1, 2014, to Dec 31, 2015	559: 11	Not specified: 56.3	49.4	Mixed modalities
Wang et al, 2019[84]	Taiwan	Kaohsiung Medical University Hospital	Retrospective: Jan 1, 2007, to Dec 31, 2016	336: 1	Adult: Not specified	Not specified	Mixed modalities

Abbreviations: APD, automated peritoneal dialysis; CAPD, continuous ambulatory peritoneal dialysis; PD, peritoneal dialysis.

Table S4 Description of the 153 Unique Study Populations of PD Patients Included in Meta-Analysis (Continued)

Source, Year	Country	Study Setting	Data Collection: Study Period	Total Sample Size: No. PD Center	Study Population: Mean Age, Year	Male, %	PD Modality
Wojtaszek et al, 2019[85]	Poland	Medical University of Warsaw	Prospective: Jan 1, 2005, to Dec 31, 2015	129: 1	Not specified: 50.8	56.0	Mixed modalities
Bonnal et al, 2020[86]	France	The French Peritoneal Dialysis Registry (RDPLF) database	Registry data: Jan 1, 2012, to Dec 31, 2016	1035: 74	Adult: 57.7	64.4	Mixed modalities
Hwang et al, 2020[87]	South Korea	Korea University Anam Hospital, Seoul	Retrospective: Jan 1, 2001, to Dec 31, 2015	643: 1	Not specified: Not specified	Not specified	Not specified
Ma et al, 2020[88]	China	Baoshan Branch of Shanghai First People's Hospital, Shanghai Songjiang District Central Hospital, and Shanghai East Hospital	Retrospective: Jun 1, 2006, to Dec 31, 2018	357: 3	Adult: 63.0	59.1	CAPD
Noppakun et al, 2020[89]	Thailand	Maharaj Nakorn Chiang Mai Hospital, Nakornping General Hospital, and Songklanagarind Hospital	Retrospective: Jan 1, 2006, to May 31, 2018	1169: 3	Adult: 54.4	54.8	Mixed modalities
Perl et al, 2020[90]	Australia/New Zealand	PDOPPS-Australia/New Zealand	Registry data: Jan 1, 2014, to Dec 31, 2017	510: 17	Adult: 63.0	67.0	Mixed modalities
Perl et al, 2020[90]	Canada	PDOPPS-Canada	Registry data: Jan 1, 2014, to Dec 31, 2017	917: 20	Adult: 61.0	62.0	Mixed modalities
Perl et al, 2020[90]	United Kingdom	PDOPPS-United Kingdom	Registry data: Jan 1, 2014, to Dec 31, 2017	342: 18	Adult: 61.0	65.0	Mixed modalities
Perl et al, 2020[90]	United States	PDOPPS-United States	Registry data: Jan 1, 2014, to Dec 31, 2017	3635: 103	Adult: 58.0	56.0	Mixed modalities
Tekkarismaz et al, 2020[91]	Türkiye	Başkent University, Adana	Retrospective: Jan 1, 2011, to May 30, 2019	242: 1	Adult: 52.5	47.5	Mixed modalities
Viglino et al, 2020[92]	Italy	San Lazzaro Hospital, Alba	Retrospective: Jan 1, 2009, to Dec 31, 2018	107: 1	Not specified: 72.2	58.9	Not specified
Zeng et al, 2020[93]	China	Second Affiliated Hospital of Soochow University, Suzhou	Retrospective: Jan 1, 2011, to Dec 31, 2019	909: 1	Not specified: 61.7	47.9	CAPD
AlZabli et al, 2021[94]	Saudi Arabia	Children Specialized Hospital, King Fahad Medical City, Riyadh	Retrospective: Jan 1, 2007, to Dec 31, 2018	131: 1	Pediatric: Not specified	51.9	APD
de la Espada Pina et al, 2021[95]	Spain	SICATA database, including all hospitals providing PD in Andalusia	Registry data: Jan 1, 1999, to Dec 31, 2017	2904: 12	Not specified: 56.7	60.2	Mixed modalities
Htay et al, 2021[96]	Singapore	Singapore General Hospital	Ambispective: Jan 1, 2016, to Aug 31, 2019	288: 1	Adult: 61.8	46.5	Mixed modalities
Hu et al, 2021[97]	China	Third Xiangya Hospital of Central South University, Changsha, Hunan	Retrospective: Jan 1, 2009, to Sep 1, 2018	591: 1	Adult: 46.9	57.0	CAPD
Kang et al, 2021[98]	South Korea	Seoul National University Hospital	Retrospective: Jan 1, 1990, to Dec 31, 2019	1203: 1	Adult: 47.9	58.3	Not specified
Li et al, 2021[99]	China	Guangdong Provincial Hospital of Chinese Medicine, Guangzhou	Retrospective: Jan 1, 2011, to Dec 31, 2020	739: 1	Adult: 50.8	56.4	Not specified

Abbreviations: APD, automated peritoneal dialysis; CAPD, continuous ambulatory peritoneal dialysis; PD, peritoneal dialysis.

Table S4 Description of the 153 Unique Study Populations of PD Patients Included in Meta-Analysis (Continued)

Source, Year	Country	Study Setting	Data Collection: Study Period	Total Sample Size: No. PD Center	Study Population: Mean Age, Year	Male, %	PD Modality
Liu et al, 2021[100]	China	Nanfang Hospital, Southern Medical University, Guangzhou	Retrospective: Jan 1, 2009, to Dec 31, 2019	602: 1	Adult: 45.0	58.2	Not specified
Neu et al, 2021[101]	United States	SCOPE Collaborative database	Prospective: Oct 1, 2011, to Sep 30, 2018	1055: 1	Pediatric: 7.7	54.1	Not specified
Oki et al, 2021[102]	Japan	The University of Tokyo Hospital, Tokyo	Retrospective: Jan 1, 2001, to Dec 31, 2019	159: 1	Not specified: Not specified	Not specified	Not specified
Sakurada et al, 2021[103]	Japan	St. Marianna University School of Medicine, Kanagawa	Retrospective: Jan 1, 2009, to Dec 31, 2018	126: 1	Not specified: 60.7	59.5	Not specified
Santos et al, 2021[104]	Spain	University Hospital A Coruña, A Coruña	Retrospective: Jan 1, 1990, to Dec 31, 2019	878: 1	Adult: 59.2	59.5	Mixed modalities
Song et al, 2021[105]	China	Renji Hospital, Shanghai Jiao Tong University School of Medicine, Shanghai	Retrospective: Jan 1, 2009, to Dec 31, 2019	180: 1	Adult: 78.6	58.9	CAPD
Tangjittrong, 2021[106]	Thailand	Pranangklao Hospital, Nonthaburi	Retrospective: Oct 1, 2011, to Mar 1, 2021	411: 1	Not specified: 57.5	55.2	CAPD
Witoon et al, 2021[107]	Thailand	Burapha University Hospital and Chonburi Hospital, Chonburi	Retrospective: Jan 1, 2013, to Dec 31, 2017	532: 2	Not specified: 58.1	46.6	CAPD
Cheikh Hassan et al, 2022[108]	Australia	ANZDATA database	Registry data: Oct 1, 2003, to Dec 31, 2019	9699: 68	Adult: 60.0	61.0	Mixed modalities
Kanjanabuch et al, 2022[109]	Thailand	PDOPPS-Thailand	Registry data: May 2, 2016, to Feb 1, 2018	848: 22	Adult: 56.2	51.0	Mixed modalities
Kolland et al, 2022[110]	Austria	Medical University of Graz	Retrospective: Jan 1, 2007, to Dec 31, 2017	250: 1	Not specified: 56.1	67.6	Mixed modalities
Lau et al, 2022[111]	Malaysia	Selayang Hospital, Selayang	Retrospective: Jan 1, 2011, to Dec 31, 2019	Not specified: 1	Adult: 61.5	53.0	Mixed modalities
Melanson et al, 2022[112]	Canada	Maisonneuve-Rosemont hospital, Montreal, Quebec	Retrospective: Jan 1, 2015, to Dec 31, 2020	124: 1	Adult: 60.5	66.6	Not specified
Özbek et al, 2022[113]	Türkiye	Aydın Adnan Menderes University, Faculty of Medicine, Department of Medical Education, Aydın, Turquía	Retrospective: Jan 1, 2009, to Dec 31, 2018	352: 1	Not specified: 56.2	67.5	Mixed modalities
Szeto et al, 2022[114]	Hong Kong	Prince of Wales Hospital, The Chinese University of Hong Kong, Shatin	Retrospective: Jan 1, 2000, to Dec 31, 2019	1193: 1	Not specified: 61.7	57.21	Mixed modalities
Wu et al, 2022[115]	China	First Affiliated Hospital, Sun Yat-sen University, Guangzhou, Guangdong	Retrospective: Jan 1, 2006, to Dec 31, 2018	2570: 1	Adult: 50.4	47.8	CAPD
Zhai et al, 2022[116]	China	Children's Hospital of Fudan University, Shanghai	Retrospective: Jan 1, 2001, to Dec 31, 2018	283: 1	Pediatric: 9.2	53.7	Not specified
Alves et al, 2023[117]	Brazil	Botucatu Medical School and Bauru State Hospital, Sao Paulo; and Pro-Rim Foundation, Santa Catarina	Prospective: Jul 1, 2014, and Jul 31, 2020	370: 3	Not specified: 57.8	54.6	Not specified

Abbreviations: APD, automated peritoneal dialysis; CAPD, continuous ambulatory peritoneal dialysis; PD, peritoneal dialysis.

Table S4 Description of the 153 Unique Study Populations of PD Patients Included in Meta-Analysis (Continued)

Source, Year	Country	Study Setting	Data Collection: Study Period	Total Sample Size: No. PD Center	Study Population: Mean Age, Year	Male, %	PD Modality
Çankaya et al, 2023[118]	Türkiye	Atatürk University, Erzurum	Retrospective: Mar 1, 2010, to Aug 31, 2020	202: 1	Not specified: 53.8	53.0	Mixed modalities
Chienwichai et al, 2023[119]	Thailand	Hatyai Hospital, Songkhla	Retrospective: Mar 1, 2021, to Oct 31, 2022	336: 1	Adult: 57.0	44.4	Mixed modalities
Forté et al, 2023[120]	France	The French Peritoneal Dialysis Registry (RDPLF) database	Registry data: Jan 1, 2014, to Nov 12, 2020	8848: 158	Adult: 68.4	64.0	Mixed modalities
Guo et al, 2023[121]	China	Zhongshan Hospital, Fudan University, Shanghai	Prospective: Feb 1, 2018, to Jul 31, 2019	101: 1	Adult: 57.1	47.5	CAPD
Pak et al, 2023[122]	Hong Kong	United Christian Hospital, Kwun Tong of New Kowloon	Retrospective: Jan 1, 2007, to Dec 31, 2016	172: 1	Not specified: 63.6	65.1	CAPD
Rivera et al, 2023[123]	Colombia	Network of Baxter Renal Care Services (BRCS)	Retrospective: Jan 1, 2017, to Dec 31, 2020	4743: 45	Adult: 61.1	56.8	Mixed modalities
Srivastava et al, 2023[124]	India	Base Hospital, Delhi Cantt	Retrospective: Jul 1, 2018, to Dec 31, 2019	200: 1	Adult: 55.7	57.0	CAPD
Taher et al, 2023[125]	Palestine	An-Najah National University Hospital, Nablus	Retrospective: Nov 1, 2016, to Dec 31, 2021	158: 1	Adult: 51.8	53.8	CAPD
Yang et al, 2023[126]	Taiwan	Linkou Chang Gung Memorial Hospital, Taoyuan	Retrospective: Jan 1, 1985, to Dec 31, 2019	2084: 1	Not specified: Not specified	Not specified	Not specified
Ye et al, 2023[127]	China	Yiwu Central Hospital, Zhejiang	Retrospective: Jan 1, 2015, to Apr 30, 2020	138: 1	Not specified: 55.8	48.7	Mixed modalities
Aldriwesh et al, 2024[128]	Saudi Arabia	King Abdullah International Medical Research Centre, Riyadh	Retrospective: Jan 1, 2009, to Dec 31, 2019	100: 1	Mixed population: 42.4	45.0	Mixed modalities
Centellas-Pérez et al, 2024[129]	Spain	16 hospitals across Spain	Prospective: Jun 1 to Dec 31, 2021	232: 16	Adult: 55.6	65.5	APD
Dandecha et al, 2024[130]	Thailand	Songklanagarind Hospital, Songkla	Retrospective: Jan 1, 2009, to Dec 31, 2021	120: 1	Adult: 62.8	49.2	CAPD
Doğan et al, 2024[131]	Türkiye	Hitit University, Corum; Ankara University, Ankara; 19 Mayis University, Samsun; Bursa City Hospital, Bursa; Hacettepe University, Ankara; Uludag University, Bursa; and Eskisehir Osmangazi University, Eskisehir	Prospective: Jun 1, 2019, to Jun 31, 2021	120: 7	Adult: 54.9	52.5	Mixed modalities
Dong et al, 2024[132]	China	First Affiliated Hospital of Sun Yat-sen University, Guangzhou and Foshan First People's Hospital, Guangdong	Retrospective: Jan 1, 2008, to Dec 31, 2022	3459: 2	Adult: 52.7	47.3	Not specified
Gül, 2024[133]	Türkiye	Bursa Yüksek İhtisas Training and Research Hospital, Bursa	Retrospective: Jan 1, 2009, to Jul 31, 2021	171: 1	Not specified: 51.9	43.3	CAPD
Ito et al, 2024[134]	Japan	PDOPPS-Japan	Registry data: Jan 1, 2014, to Dec 31, 2022	1225: 30	Adult: 64.3	67.0	Mixed modalities

Abbreviations: APD, automated peritoneal dialysis; CAPD, continuous ambulatory peritoneal dialysis; PD, peritoneal dialysis.

Table S4 Description of the 153 Unique Study Populations of PD Patients Included in Meta-Analysis (Continued)

Source, Year	Country	Study Setting	Data Collection: Study Period	Total Sample Size: No. PD Center	Study Population: Mean Age, Year	Male, %	PD Modality
Liang et al, 2024[135]	China	Peking University People's Hospital, Beijing	Retrospective: Jan 1, 2007, to Oct 31, 2021	166: 1	Not specified: 63.7	46.5	Mixed modalities
Mehrotra et al, 2024[136]	United States	CloudCath system database	Prospective: Aug 1, 2020, to Jun 31, 2022	243: 19	Adult: 59.0	58.9	Not specified
Nabil et al, 2024[137]	Morocco	Ibn Sina University Hospital Center, Rabat; alongside the French Language Peritoneal Dialysis Registry (RDPLF) database	Registry data: Jan 1, 2006, to Jan 31, 2024	235: 1	Mixed population: 45.9	59.4	Not specified
Nardelli et al, 2024[138]	Italy	IRCCS Ca' Granda Foundation Maggiore Policlinico Hospital, Milan	Retrospective: Jan 1, 2009, to May 31, 2023	267: 1	Adult: 63.1	66.7	Mixed modalities
Park et al, 2024[139]	South Korea	Seoul National University Children's Hospital, Seoul	Retrospective: Jan 1, 2000, to Dec 31, 2017	184: 1	Pediatric: 10.2	58.2	Mixed modalities
Tobijaszewska et al, 2024[140]	Sweden	Population-based of all PD patients in the county of Skåne, south Sweden: Malmö, Lund, Helsingborg, Hässleholm, and Ystad	Retrospective: Jan 1, 2011, to Dec 31, 2020	675: 5	Mixed population: 56.0	69.2	Mixed modalities
Alfano et al, 2025[141]	Italy	Azienda Ospedaliera Universitaria of Modena, Modena	Retrospective: Jan 1, 1999, to Dec 31, 2023	Not specified: 1	Adult: 67.0	55.8	Mixed modalities
Andrew et al, 2025[142]	United Kingdom	Single region at the Northern Ireland Regional Nephrology and Transplant Centre	Prospective: Jan 1, 2020, to Dec 31, 2024	157: 5	Adult: 55.5	58.0	Not specified
Guo et al, 2025[143]	China	Hospitals in Jiangsu Province: Affiliated Hospital of Nantong University, Nantong; Yancheng Third People's Hospital, Yancheng; the Affiliated Hospital of Xuzhou Medical University, Xuzhou	Retrospective: Jan 1, 2016, to Dec 31, 2024	392: 3	Adult: 49.7	59.7	Mixed modalities
Kim et al, 2025[144]	South Korea	PDOPPS-Korea	Registry data: Jul 1, 2019, to Dec 31, 2021	766: 20	Adult: 54.8	59.5	Mixed modalities
Soetendorp et al, 2025[145]	Israel	Tel Aviv Sourasky Medical Center, Tel Aviv	Ambispective: Jan 1, 2016, to May 31, 2021	113: 1	Not specified: 70.0	61.1	Not specified
Tsuchiya et al, 2025[146]	Japan	Jikei University Hospital and Katsushika Medical Center, Tokyo	Retrospective: Jan 1, 2016, to Dec 31, 2022	180: 2	Adult: 60.0	74.8	Mixed modalities
Yu et al, 2025[147]	China	First Affiliated Hospital of Xi'an Jiaotong University, Xi'an	Retrospective: Nov 1, 2022, to Jul 31, 2024	506: 1	Adult: 55.9	65.0	Not specified
Al Laham et al, 2026[148]	Oman	Nizwa Hospital, Nizwa	Retrospective: Mar 1, 2005, to Mar 31, 2023	311: 1	Mixed population: Not specified	Not specified	Not specified
Cho et al, 2026[149]	South Korea	Soonchunhyang University Cheonan Hospital, Cheonan	Retrospective: Jan 1, 2016, to Dec 31, 2023	162: 1	Not specified: 52.2	53.7	Mixed modalities
Okamoto et al, 2026[150]	Japan	Nara Medical University Hospital, Nara	Retrospective: Jan 1, 2007, to Dec 31, 2020	105: 1	Adult: 60.7	71.4	Mixed modalities

Abbreviations: APD, automated peritoneal dialysis; CAPD, continuous ambulatory peritoneal dialysis; PD, peritoneal dialysis.

Table S5 Quality Assessment of the Included Studies for Incidence Rate Estimation of PD-Associated Peritonitis[a]

Source, Year	External Validity					Internal Validity					Summary Score (Overall Study Quality)
	Q1	Q2	Q3	Q4	Q5	Q6	Q7	Q8	Q9	Q10	
Balasubramanian et al, 2011[1]	No	Yes	Yes	Yes	Yes	Yes	Yes	Yes	Yes	Yes	9 (high quality)
Brown et al, 2011[2]	Yes	Yes	Yes	Yes	Yes	Yes	Yes	Yes	Yes	Yes	10 (very high quality)
Davenport et al, 2011[3]	No	Yes	Yes	Yes	Yes	Yes	Yes	Yes	Yes	Yes	9 (high quality)
Huang et al, 2011[4]	No	Yes	Yes	Yes	Yes	Yes	Yes	Yes	Yes	Yes	9 (high quality)
Kanjanabuch et al, 2011[5]	Yes	Yes	Yes	Yes	Yes	Yes	Yes	Yes	Yes	Yes	10 (very high quality)
Liawnoraset, 2011[6]	No	Yes	Yes	Yes	Yes	Yes	Yes	Yes	Yes	Yes	9 (high quality)
Luvira et al, 2011[7]	No	Yes	Yes	Yes	Yes	Yes	Yes	Yes	Yes	Yes	9 (high quality)
Martin et al, 2011[8]	Yes	Yes	Yes	Yes	Yes	Yes	Yes	Yes	Yes	Yes	10 (very high quality)
Muñoz de Bustillo et al, 2011[9]	Yes	Yes	Yes	Yes	Yes	Yes	Yes	Yes	Yes	Yes	10 (very high quality)
Oygar et al, 2011[10]	No	Yes	Yes	Yes	Yes	Yes	Yes	Yes	Yes	Yes	9 (high quality)
Quintanar Lartundo et al, 2011[11]	No	Yes	Yes	Yes	Yes	Yes	Yes	Yes	Yes	Yes	9 (high quality)
Topanthanont et al, 2011[12]	No	Yes	Yes	Yes	Yes	Yes	Yes	Yes	Yes	Yes	9 (high quality)
Aksu et al, 2012[13]	No	Yes	Yes	Yes	Yes	Yes	Yes	Yes	Yes	Yes	9 (high quality)
Barone et al, 2012[14]	No	Yes	Yes	Yes	Yes	Yes	Yes	Yes	Yes	Yes	9 (high quality)
Basturk et al, 2012[15]	No	Yes	Yes	Yes	Yes	Yes	Yes	Yes	Yes	Yes	9 (high quality)
Chen et al, 2012[16]	Yes	Yes	Yes	Yes	Yes	Yes	Yes	Yes	Yes	Yes	10 (very high quality)
Levallois et al, 2012[17]	Yes	Yes	Yes	Yes	Yes	Yes	Yes	Yes	Yes	Yes	10 (very high quality)
Nayak et al, 2012[18]	No	Yes	Yes	Yes	Yes	Yes	Yes	Yes	Yes	Yes	9 (high quality)
Oliveira et al, 2012[19]	No	Yes	Yes	Yes	Yes	Yes	Yes	Yes	Yes	Yes	9 (high quality)
Pierce et al, 2012[20]	No	Yes	Yes	Yes	Yes	Yes	Yes	Yes	Yes	Yes	9 (high quality)
Rocha et al, 2012[21]	No	Yes	Yes	Yes	Yes	Yes	Yes	Yes	Yes	Yes	9 (high quality)
Su et al, 2012[22]	No	Yes	Yes	Yes	Yes	Yes	Yes	Yes	Yes	Yes	9 (high quality)
Xu et al, 2012[23]	No	Yes	Yes	Yes	Yes	Yes	Yes	Yes	Yes	Yes	9 (high quality)
Xu et al, 2012[24]	No	Yes	Yes	Yes	Yes	Yes	Yes	Yes	Yes	Yes	9 (high quality)
Yap et al, 2012[25]	No	Yes	Yes	Yes	Yes	Yes	Yes	Yes	Yes	Yes	9 (high quality)
Yap et al, 2012[26]	No	Yes	Yes	Yes	Yes	Yes	Yes	Yes	Yes	Yes	9 (high quality)
Chern et al, 2013[27]	No	Yes	Yes	Yes	Yes	Yes	Yes	Yes	Yes	Yes	9 (high quality)

[a]See details of assessment criteria in Supplementary File S2—Study Quality Appraisal Tool.

Table S5 Quality Assessment of the Included Studies for Incidence Rate Estimation of PD-Associated Peritonitis[a]

**Source, Year**	**External Validity**				**Internal Validity**						**Summary Score (Overall Study Quality)**
	**Q1**	**Q2**	**Q3**	**Q4**	**Q5**	**Q6**	**Q7**	**Q8**	**Q9**	**Q10**	
Huang et al, 2013[28]	No	Yes	Yes	Yes	Yes	Yes	Yes	Yes	Yes	Yes	9 (high quality)
Kerschbaum et al, 2013[29]	Yes	Yes	Yes	Yes	Yes	Yes	Yes	Yes	Yes	Yes	10 (very high quality)
Nessim et al, 2013[30]	No	Yes	Yes	Yes	Yes	Yes	Yes	Yes	Yes	Yes	9 (high quality)
Özbalci et al, 2013[31]	No	Yes	Yes	Yes	Yes	Yes	Yes	Yes	Yes	Yes	9 (high quality)
Portolés et al, 2013[32]	Yes	Yes	Yes	Yes	Yes	Yes	Yes	Yes	Yes	Yes	10 (very high quality)
Tsai et al, 2013[33]	No	Yes	Yes	Yes	Yes	Yes	Yes	Yes	Yes	Yes	9 (high quality)
Hsieh et al, 2014[34]	No	Yes	Yes	Yes	Yes	Yes	Yes	Yes	Yes	Yes	9 (high quality)
Isla et al, 2014[35]	No	Yes	Yes	Yes	Yes	Yes	Yes	Yes	Yes	Yes	9 (high quality)
Kumar et al, 2014[36]	No	Yes	Yes	Yes	Yes	Yes	Yes	Yes	Yes	Yes	9 (high quality)
Najafi et al, 2014[37]	Yes	Yes	Yes	Yes	Yes	Yes	Yes	Yes	Yes	Yes	10 (very high quality)
Nieto-Ríos et al, 2014[38]	No	Yes	Yes	Yes	Yes	Yes	Yes	Yes	Yes	Yes	9 (high quality)
Nishina et al, 2014[39]	No	Yes	Yes	Yes	Yes	Yes	Yes	Yes	Yes	Yes	9 (high quality)
Núñez-Moral et al, 2014[40]	No	Yes	Yes	Yes	Yes	Yes	Yes	Yes	Yes	Yes	9 (high quality)
Prasad et al, 2014[41]	No	Yes	Yes	Yes	Yes	Yes	Yes	Yes	Yes	Yes	9 (high quality)
Chang et al, 2015[42]	No	Yes	Yes	Yes	Yes	Yes	Yes	Yes	Yes	Yes	9 (high quality)
Courivaud et al, 2015[43]	No	Yes	Yes	Yes	Yes	Yes	Yes	Yes	Yes	Yes	9 (high quality)
Figueiredo et al, 2015[44]	Yes	Yes	Yes	Yes	Yes	Yes	Yes	Yes	Yes	Yes	10 (very high quality)
Lei et al, 2015[45]	No	Yes	Yes	Yes	Yes	Yes	Yes	Yes	Yes	Yes	9 (high quality)
Ozisik et al, 2015[46]	No	Yes	Yes	Yes	Yes	Yes	Yes	Yes	Yes	Yes	9 (high quality)
Pi et al, 2015[47]	No	Yes	Yes	Yes	Yes	Yes	Yes	Yes	Yes	Yes	9 (high quality)
Sanabria et al, 2015[48]	Yes	Yes	Yes	Yes	Yes	Yes	Yes	Yes	Yes	Yes	10 (very high quality)
Buttigieg et al, 2016[49]	No	Yes	Yes	Yes	Yes	Yes	Yes	Yes	Yes	Yes	9 (high quality)
Duquennoy et al, 2016[50]	Yes	Yes	Yes	Yes	Yes	Yes	Yes	Yes	Yes	Yes	10 (very high quality)
El-Reshaid et al, 2016[51]	No	Yes	Yes	Yes	Yes	Yes	Yes	Yes	Yes	Yes	9 (high quality)
Higuchi et al, 2016[52]	Yes	Yes	Yes	Yes	Yes	Yes	Yes	Yes	Yes	Yes	10 (very high quality)
Kotera et al, 2016[53]	No	Yes	Yes	Yes	Yes	Yes	Yes	Yes	Yes	Yes	9 (high quality)
Mizuno et al, 2016[54]	No	Yes	Yes	Yes	Yes	Yes	Yes	Yes	Yes	Yes	9 (high quality)

[a]See details of assessment criteria in Supplementary File S2—Study Quality Appraisal Tool.

Table S5 Quality Assessment of the Included Studies for Incidence Rate Estimation of PD-Associated Peritonitis[a]

**Source, Year**	**External Validity**					**Internal Validity**					**Summary Score (Overall Study Quality)**
	**Q1**	**Q2**	**Q3**	**Q4**	**Q5**	**Q6**	**Q7**	**Q8**	**Q9**	**Q10**	
Nakao et al, 2016[55]	No	Yes	Yes	Yes	Yes	Yes	Yes	Yes	Yes	Yes	9 (high quality)
Pai et al, 2016[56]	No	Yes	Yes	Yes	Yes	Yes	Yes	Yes	Yes	Yes	9 (high quality)
Pérez-Fontan et al, 2016[57]	No	Yes	Yes	Yes	Yes	Yes	Yes	Yes	Yes	Yes	9 (high quality)
Perl et al, 2016[58]	Yes	Yes	Yes	Yes	Yes	Yes	Yes	Yes	Yes	Yes	10 (very high quality)
Sandrini et al, 2016[59]	No	Yes	Yes	Yes	Yes	Yes	Yes	Yes	Yes	Yes	9 (high quality)
Santhakumaran et al, 2016[60]	Yes	Yes	Yes	Yes	Yes	Yes	Yes	Yes	Yes	Yes	10 (very high quality)
Shea et al, 2016[61]	No	Yes	Yes	Yes	Yes	Yes	Yes	Yes	Yes	Yes	9 (high quality)
Tian et al, 2016[62]	No	Yes	Yes	Yes	Yes	Yes	Yes	Yes	Yes	Yes	9 (high quality)
Alatab et al, 2017[63]	No	Yes	Yes	Yes	Yes	Yes	Yes	Yes	Yes	Yes	9 (high quality)
Htay et al, 2017[64]	No	Yes	Yes	Yes	Yes	Yes	Yes	Yes	Yes	Yes	9 (high quality)
Lee et al, 2017[65]	No	Yes	Yes	Yes	Yes	Yes	Yes	Yes	Yes	Yes	9 (high quality)
Ndlovu et al, 2017[66]	No	Yes	Yes	Yes	Yes	Yes	Yes	Yes	Yes	Yes	9 (high quality)
Ong et al, 2017[67]	Yes	Yes	Yes	Yes	Yes	Yes	Yes	Yes	Yes	Yes	10 (very high quality)
Vargas et al, 2017[68]	Yes	Yes	Yes	Yes	Yes	Yes	Yes	Yes	Yes	Yes	10 (very high quality)
Al Wakeel et al, 2018[69]	No	Yes	Yes	Yes	Yes	Yes	Yes	Yes	Yes	Yes	9 (high quality)
Fang et al, 2018[70]	No	Yes	Yes	Yes	Yes	Yes	Yes	Yes	Yes	Yes	9 (high quality)
Hsu et al, 2018[71]	No	Yes	Yes	Yes	Yes	Yes	Yes	Yes	Yes	Yes	9 (high quality)
Ponce et al, 2018[72]	Yes	Yes	Yes	Yes	Yes	Yes	Yes	Yes	Yes	Yes	10 (very high quality)
Rope et al, 2018[73]	No	Yes	Yes	Yes	Yes	Yes	Yes	Yes	Yes	Yes	9 (high quality)
Alfayez et al, 2019[74]	No	Yes	Yes	Yes	Yes	Yes	Yes	Yes	Yes	Yes	9 (high quality)
Balzer et al, 2019[75]	No	Yes	Yes	Yes	Yes	Yes	Yes	Yes	Yes	Yes	9 (high quality)
Einbinder et al, 2019[76]	No	Yes	Yes	Yes	Yes	Yes	Yes	Yes	Yes	Yes	9 (high quality)
Gadola et al, 2019[77]	No	Yes	Yes	Yes	Yes	Yes	Yes	Yes	Yes	Yes	9 (high quality)
Hu et al, 2019[78]	No	Yes	Yes	Yes	Yes	Yes	Yes	Yes	Yes	Yes	9 (high quality)
Jahromi et al, 2019[79]	No	Yes	Yes	Yes	Yes	Yes	Yes	Yes	Yes	Yes	9 (high quality)
Karthikeyan et al, 2019[80]	No	Yes	Yes	Yes	Yes	Yes	Yes	Yes	Yes	Yes	9 (high quality)
Lasfar et al, 2019[81]	No	Yes	Yes	Yes	Yes	Yes	Yes	Yes	Yes	Yes	9 (high quality)

[a]See details of assessment criteria in Supplementary File S2—Study Quality Appraisal Tool.

Table S5 Quality Assessment of the Included Studies for Incidence Rate Estimation of PD-Associated Peritonitis[a]

**Source, Year**	**External Validity**					**Internal Validity**					**Summary Score (Overall Study Quality)**
	**Q1**	**Q2**	**Q3**	**Q4**	**Q5**	**Q6**	**Q7**	**Q8**	**Q9**	**Q10**	
Liao et al, 2019[82]	Yes	Yes	Yes	Yes	Yes	Yes	Yes	Yes	Yes	Yes	10 (very high quality)
Lichodziejewska-Niemierko et al, 2019[83]	Yes	Yes	Yes	Yes	Yes	Yes	Yes	Yes	Yes	Yes	10 (very high quality)
Wang et al, 2019[84]	No	Yes	Yes	Yes	Yes	Yes	Yes	Yes	Yes	Yes	9 (high quality)
Wojtaszek et al, 2019[85]	No	Yes	Yes	Yes	Yes	Yes	Yes	Yes	Yes	Yes	9 (high quality)
Bonnal et al, 2020[86]	Yes	Yes	Yes	Yes	Yes	Yes	Yes	Yes	Yes	Yes	10 (very high quality)
Hwang et al, 2020[87]	No	Yes	Yes	Yes	Yes	Yes	Yes	Yes	Yes	Yes	9 (high quality)
Ma et al, 2020[88]	Yes	Yes	Yes	Yes	Yes	Yes	Yes	Yes	Yes	Yes	10 (very high quality)
Noppakun et al, 2020[89]	No	Yes	Yes	Yes	Yes	Yes	Yes	Yes	Yes	Yes	9 (high quality)
Perl et al, 2020[90]	Yes	Yes	Yes	Yes	Yes	Yes	Yes	Yes	Yes	Yes	10 (very high quality)
Perl et al, 2020[90]	Yes	Yes	Yes	Yes	Yes	Yes	Yes	Yes	Yes	Yes	10 (very high quality)
Perl et al, 2020[90]	Yes	Yes	Yes	Yes	Yes	Yes	Yes	Yes	Yes	Yes	10 (very high quality)
Perl et al, 2020[90]	Yes	Yes	Yes	Yes	Yes	Yes	Yes	Yes	Yes	Yes	10 (very high quality)
Tekkarismaz et al, 2020[91]	No	Yes	Yes	Yes	Yes	Yes	Yes	Yes	Yes	Yes	9 (high quality)
Viglino et al, 2020[92]	No	Yes	Yes	Yes	Yes	Yes	Yes	Yes	Yes	Yes	9 (high quality)
Zeng et al, 2020[93]	No	Yes	Yes	Yes	Yes	Yes	Yes	Yes	Yes	Yes	9 (high quality)
AlZabli et al, 2021[94]	No	Yes	Yes	Yes	Yes	Yes	Yes	Yes	Yes	Yes	9 (high quality)
de la Espada Pina et al, 2021[95]	Yes	Yes	Yes	Yes	Yes	Yes	Yes	Yes	Yes	Yes	10 (very high quality)
Htay et al, 2021[96]	No	Yes	Yes	Yes	Yes	Yes	Yes	Yes	Yes	Yes	9 (high quality)
Hu et al, 2021[97]	No	Yes	Yes	Yes	Yes	Yes	Yes	Yes	Yes	Yes	9 (high quality)
Kang et al, 2021[98]	No	Yes	Yes	Yes	Yes	Yes	Yes	Yes	Yes	Yes	9 (high quality)
Li et al, 2021[99]	No	Yes	Yes	Yes	Yes	Yes	Yes	Yes	Yes	Yes	9 (high quality)
Liu et al, 2021[100]	No	Yes	Yes	Yes	Yes	Yes	Yes	Yes	Yes	Yes	9 (high quality)
Neu et al, 2021[101]	Yes	Yes	Yes	Yes	Yes	Yes	Yes	Yes	Yes	Yes	10 (very high quality)
Oki et al, 2021[102]	No	Yes	Yes	Yes	Yes	Yes	Yes	Yes	Yes	Yes	9 (high quality)
Sakurada et al, 2021[103]	No	Yes	Yes	Yes	Yes	Yes	Yes	Yes	Yes	Yes	9 (high quality)
Santos et al, 2021[104]	No	Yes	Yes	Yes	Yes	Yes	Yes	Yes	Yes	Yes	9 (high quality)

[a]See details of assessment criteria in Supplementary File S2—Study Quality Appraisal Tool.

Table S5 Quality Assessment of the Included Studies for Incidence Rate Estimation of PD-Associated Peritonitis[a]

**Source, Year**	**External Validity**					**Internal Validity**					**Summary Score (Overall Study Quality)**
	**Q1**	**Q2**	**Q3**	**Q4**	**Q5**	**Q6**	**Q7**	**Q8**	**Q9**	**Q10**	
Song et al, 2021[105]	No	Yes	Yes	Yes	Yes	Yes	Yes	Yes	Yes	Yes	9 (high quality)
Tangjittrong, 2021[106]	No	Yes	Yes	Yes	Yes	Yes	Yes	Yes	Yes	Yes	9 (high quality)
Witoon et al, 2021[107]	No	Yes	Yes	Yes	Yes	Yes	Yes	Yes	Yes	Yes	9 (high quality)
Cheikh Hassan et al, 2022[108]	Yes	Yes	Yes	Yes	Yes	Yes	Yes	Yes	Yes	Yes	10 (very high quality)
Kanjanabuch et al, 2022[109]	Yes	Yes	Yes	Yes	Yes	Yes	Yes	Yes	Yes	Yes	10 (very high quality)
Kolland et al, 2022[110]	No	Yes	Yes	Yes	Yes	Yes	Yes	Yes	Yes	Yes	9 (high quality)
Lau et al, 2022[111]	No	Yes	Yes	Yes	Yes	Yes	Yes	Yes	Yes	Yes	9 (high quality)
Melanson et al, 2022[112]	No	Yes	Yes	Yes	Yes	Yes	Yes	Yes	Yes	Yes	9 (high quality)
Özbek et al, 2022[113]	No	Yes	Yes	Yes	Yes	Yes	Yes	Yes	Yes	Yes	9 (high quality)
Szeto et al, 2022[114]	No	Yes	Yes	Yes	Yes	Yes	Yes	Yes	Yes	Yes	9 (high quality)
Wu et al, 2022[115]	No	Yes	Yes	Yes	Yes	Yes	Yes	Yes	Yes	Yes	9 (high quality)
Zhai et al, 2022[116]	No	Yes	Yes	Yes	Yes	Yes	Yes	Yes	Yes	Yes	9 (high quality)
Alves et al, 2023[117]	Yes	Yes	Yes	Yes	Yes	Yes	Yes	Yes	Yes	Yes	10 (very high quality)
Çankaya et al, 2023[118]	No	Yes	Yes	Yes	Yes	Yes	Yes	Yes	Yes	Yes	9 (high quality)
Chienwichai et al, 2023[119]	No	Yes	Yes	Yes	Yes	Yes	Yes	Yes	Yes	Yes	9 (high quality)
Forté et al, 2023[120]	Yes	Yes	Yes	Yes	Yes	Yes	Yes	Yes	Yes	Yes	10 (very high quality)
Guo et al, 2023[121]	No	Yes	Yes	Yes	Yes	Yes	Yes	Yes	Yes	Yes	9 (high quality)
Pak et al, 2023[122]	No	Yes	Yes	Yes	Yes	Yes	Yes	Yes	Yes	Yes	9 (high quality)
Rivera et al, 2023[123]	Yes	Yes	Yes	Yes	Yes	Yes	Yes	Yes	Yes	Yes	10 (very high quality)
Srivastava et al, 2023[124]	No	Yes	Yes	Yes	Yes	Yes	Yes	Yes	Yes	Yes	9 (high quality)
Taher et al, 2023[125]	No	Yes	Yes	Yes	Yes	Yes	Yes	Yes	Yes	Yes	9 (high quality)
Yang et al, 2023[126]	No	Yes	Yes	Yes	Yes	Yes	Yes	Yes	Yes	Yes	9 (high quality)
Ye et al, 2023[127]	No	No	Yes	Yes	Yes	Yes	Yes	Yes	Yes	Yes	8 (high quality)
Aldriwesh et al, 2024[128]	No	No	Yes	Yes	Yes	Yes	Yes	Yes	Yes	Yes	8 (high quality)
Centellas-Pérez et al, 2024[129]	Yes	Yes	Yes	Yes	Yes	Yes	Yes	Yes	Yes	Yes	10 (very high quality)
Dandecha et al, 2024[130]	No	Yes	Yes	Yes	Yes	Yes	Yes	Yes	Yes	Yes	9 (high quality)
Doğan et al, 2024[131]	No	No	Yes	Yes	Yes	Yes	Yes	Yes	Yes	Yes	8 (high quality)

[a]See details of assessment criteria in Supplementary File S2—Study Quality Appraisal Tool.

Table S5 Quality Assessment of the Included Studies for Incidence Rate Estimation of PD-Associated Peritonitis[a]

Source, Year	External Validity				Internal Validity						Summary Score (Overall Study Quality)
	Q1	Q2	Q3	Q4	Q5	Q6	Q7	Q8	Q9	Q10	
Dong et al, 2024[132]	No	Yes	Yes	Yes	Yes	Yes	Yes	Yes	Yes	Yes	9 (high quality)
Gül, 2024[133]	No	Yes	Yes	Yes	Yes	Yes	Yes	Yes	Yes	Yes	9 (high quality)
Ito et al, 2024[134]	Yes	Yes	Yes	Yes	Yes	Yes	Yes	Yes	Yes	Yes	10 (very high quality)
Liang et al, 2024[135]	No	Yes	Yes	Yes	Yes	Yes	Yes	Yes	Yes	Yes	9 (high quality)
Mehrotra et al, 2024[136]	Yes	Yes	Yes	Yes	Yes	Yes	Yes	Yes	Yes	Yes	10 (very high quality)
Nabil et al, 2024[137]	No	Yes	Yes	Yes	Yes	Yes	Yes	Yes	Yes	Yes	9 (high quality)
Nardelli et al, 2024[138]	No	Yes	Yes	Yes	Yes	Yes	Yes	Yes	Yes	Yes	9 (high quality)
Park et al, 2024[139]	No	Yes	Yes	Yes	Yes	Yes	Yes	Yes	Yes	Yes	9 (high quality)
Tobijaszewska et al, 2024[140]	Yes	Yes	Yes	Yes	Yes	Yes	Yes	Yes	Yes	Yes	10 (very high quality)
Alfano et al, 2025[141]	No	Yes	Yes	Yes	Yes	Yes	Yes	Yes	Yes	Yes	9 (high quality)
Andrew et al, 2025[142]	No	Yes	Yes	Yes	Yes	Yes	Yes	Yes	Yes	Yes	9 (high quality)
Guo et al, 2025[143]	No	Yes	Yes	Yes	Yes	Yes	Yes	Yes	Yes	Yes	9 (high quality)
Kim et al, 2025[144]	Yes	Yes	Yes	Yes	Yes	Yes	Yes	Yes	Yes	Yes	10 (very high quality)
Soetendorp et al, 2025[145]	No	Yes	Yes	Yes	Yes	Yes	Yes	Yes	Yes	Yes	9 (high quality)
Tsuchiya et al, 2025[146]	No	Yes	Yes	Yes	Yes	Yes	Yes	Yes	Yes	Yes	9 (high quality)
Yu et al, 2025[147]	No	Yes	Yes	Yes	Yes	Yes	Yes	Yes	Yes	Yes	9 (high quality)
Al Laham et al, 2026[148]	No	Yes	Yes	Yes	Yes	Yes	Yes	Yes	Yes	Yes	9 (high quality)
Cho et al, 2026[149]	No	Yes	Yes	Yes	Yes	Yes	Yes	Yes	Yes	Yes	9 (high quality)
Okamoto et al, 2026[150]	No	Yes	Yes	Yes	Yes	Yes	Yes	Yes	Yes	Yes	9 (high quality)

[a]See details of assessment criteria in Supplementary File S2—Study Quality Appraisal Tool.

Table S6 Meta-Analysis of Incidence Rate Estimation of PD-Associated Peritonitis, by Country

Country	No. of Estimates (Total Sample Size)	Total PD-Associated Peritonitis Episode: Total Patient-Year at Risk	Incidence Rate Estimation (95% CI), Episode per Patient-Year	95% Prediction Interval	Heterogeneity			
					Q statistic	*P* value	I^2 (95% CI), %	τ^2
Argentina	1 (n=118)	157: 441.42	0.36 (0.30 – 0.42)	NA	NA	NA	NA	NA
Australia	1 (n=9699)	8936: 24270.00	0.37 (0.36 – 0.38)	NA	NA	NA	NA	NA
Austria	2 (n=976)	707: 2365.7	0.28 (0.20 – 0.38)	NA	12.88	<0.001	92.2 (NA)	0.049
Brazil	5 (n=5325)	1881: 5500.25	0.38 (0.30 – 0.49)	0.15 – 0.99	100.60	<0.001	96.02 (93.12 – 97.70)	0.073
Canada	5 (n=6514)	4655: 11359.57	0.40 (0.30 – 0.55)	0.12 – 1.36	346.99	<0.001	98.85 (98.33 – 99.20)	0.122
China	23 (n=14041)	8577: 48926.17	0.19 (0.16 – 0.22)	0.09 – 0.38	822.96	<0.001	97.33 (96.71 – 97.83)	0.107
Colombia	4 (n=10110)	3589: 7777.03	0.34 (0.15 – 0.76)	0.01 – 17.70	1146.90	<0.001	99.74 (99.66 – 99.80)	0.675
France	3 (n=10123)	4826: 15119.54	0.31 (0.27 – 0.35)	0.07 – 1.33	12.99	0.002	84.60 (54.22 – 94.82)	0.009
Germany	1 (n=275)	160: 660.67	0.24 (0.21 – 0.28)	NA	NA	NA	NA	NA
Hong Kong	5 (n=3060)	5767: 15342.69	0.36 (0.27 – 0.49)	0.11 – 1.14	371.27	<0.001	98.92 (98.45 – 99.25)	0.108
India	5 (n=1350)	701: 1855.44	0.39 (0.29 – 0.52)	0.13 – 1.16	52.42	<0.001	92.37 (85.18 – 96.07)	0.095
Iran	3 (n=2608)	1478: 3423.75	0.39 (0.26 – 0.57)	0.00 – 51.34	65.67	<0.001	96.95 (93.84 – 98.49)	0.110
Israel	2 (n=314)	283: 390.41	0.53 (0.17 – 1.69)	NA	47.13	<0.001	97.9 (NA)	0.686
Italy	4 (n=552[a])	689: 2904.23	0.24 (0.22 – 0.26)	0.20 – 0.28	1.03	0.795	0.00 (0.00 – 84.69)	<0.001
Japan	10 (n=6912)	2756: 12840.76	0.20 (0.18 – 0.23)	0.12 – 0.34	109.29	<0.001	91.76 (86.97 – 94.80)	0.043
Kuwait	1 (n=308)	145: 522.42	0.28 (0.24 – 0.33)	NA	NA	NA	NA	NA
Malaysia	2 (n=1603[a])	1260: 5238.13	0.25 (0.21 – 0.29)	NA	7.32	0.007	86.3 (NA)	0.012
Malta	1 (n=137)	144: 369.23	0.39 (0.33 – 0.46)	NA	NA	NA	NA	NA
Morocco	1 (n=235)	378: 782.42	0.48 (0.44 – 0.53)	NA	NA	NA	NA	NA
Oman	1 (n=311)	490: 2333.33	0.21 (0.19 – 0.23)	NA	NA	NA	NA	NA

[a]Missing information for 1 study.

Abbreviations: CI, confidence interval; NA, not applicable; PD, peritoneal dialysis.

Table S6 Meta-Analysis of Incidence Rate Estimation of PD-Associated Peritonitis, by Country (Continued)

Country	No. of Estimates (Total Sample Size)	Total PD-Associated Peritonitis Episode: Total Patient-Year at Risk	Incidence Rate Estimation (95% CI), Episode per Patient-Year	95% Prediction Interval	Heterogeneity			
					Q statistic	*P* value	I^2 (95% CI), %	τ^2
Palestine	1 (n=158)	48: 152.80	0.31 (0.24 – 0.42)	NA	NA	NA	NA	NA
Poland	2 (n=688)	239: 906.53	0.25 (0.18 – 0.35)	NA	5.42	0.020	81.5 (NA)	0.052
Portugal	1 (n=438)	588: 985.30	0.60 (0.55 – 0.65)	NA	NA	NA	NA	NA
Saudi Arabia	4 (n=442)	472: 921.94	0.49 (0.36 – 0.67)	0.11 – 2.16	34.00	<0.001	91.18 (80.52 – 96.00)	0.094
Singapore	2 (n=625)	143: 536.30	0.26 (0.18 – 0.38)	NA	5.24	0.022	80.9 (NA)	0.063
South Africa	2 (n=292)	340: 363.82	0.99 (0.68 – 1.45)	NA	11.99	0.001	91.7 (NA)	0.068
South Korea	7 (n=3658)	2133: 13171.49	0.17 (0.12 – 0.26)	0.04 – 0.75	472.46	<0.001	98.73 (98.26 – 99.07)	0.286
Spain	8 (n=8009)	7435: 11097.97	0.56 (0.30 – 1.02)	0.06 – 5.40	4440.85	<0.001	99.84 (99.82 – 99.86)	0.768
Sri Lanka	1 (n=116)	47: 80.86	0.58 (0.44 – 0.77)	NA	NA	NA	NA	NA
Sweden	1 (n=675)	484: 1273.68	0.38 (0.34 – 0.42)	NA	NA	NA	NA	NA
Taiwan	9 (n=4924)	3935: 16704.41	0.22 (0.19 – 0.26)	0.12 – 0.40	155.53	<0.001	94.86 (92.15 – 96.63)	0.054
Thailand	10 (n=12371)	6068: 13872.53	0.41 (0.33 – 0.50)	0.19 – 0.87	469.33	<0.001	98.08 (97.43 – 98.57)	0.099
Tunisia	1 (n=182)	186: 422.38	0.44 (0.38 – 0.51)	NA	NA	NA	NA	NA
Türkiye	10 (n=2254)	2466: 7047.48	0.34 (0.25 – 0.48)	0.09 – 1.25	601.07	<0.001	98.50 (98.04 – 98.86)	0.284
United Kingdom	6 (n=5546[a])	5992: 11081.19	0.43 (0.36 – 0.51)	0.23 – 0.81	156.54	<0.001	96.81 (94.94 – 97.98)	0.043
United States	4 (n=5310)	1892: 6104.68	0.34 (0.25 – 0.44)	0.09 – 1.26	88.59	<0.001	96.61 (93.83 – 98.14)	0.075
Uruguay	1 (n=219)	171: 469.08	0.36 (0.31 – 0.42)	NA	NA	NA	NA	NA

[a]Missing information for 1 study.

Abbreviations: CI, confidence interval; NA, not applicable; PD, peritoneal dialysis.

Table S7 Sensitivity Analysis of Incidence Rate Estimation of PD-Associated Peritonitis

Meta-Synthesis of Evidence	No. of Estimates (Total Sample Size)	Total PD-Associated Peritonitis Episode: Total Patient-Year at Risk	Incidence Rate Estimation (95% CI), Episode per Patient-Year	95% Prediction Interval	*P* for Difference Compared to Main Analysis	Heterogeneity			
						Q statistic	*P* value	I^2 (95% CI)	τ^2
Main analysis	153 (n=132213[a])	88769: 264296.30	0.31 (0.28 – 0.34)	0.10 – 0.91	NA	25973.90	<0.001	99.41 (99.39 – 99.44)	0.300
Sensitivity analysis									
• Limited analysis to studies that directness reported PD-associated peritonitis episodes with causative organism	77 (n=54102[b])	34956: 104173.10	0.31 (0.28 – 0.34)	0.11 – 0.83	1.000	8229.70	<0.001	99.08 (99.00 – 99.15)	0.244
• Limited analysis to studies that represent national PD population	38 (n=82900[c])	45007: 112120.80	0.35 (0.30 – 0.42)	0.12 – 1.02	0.243	11096.90	<0.001	100 (100 – 100)	0.269
• Limited analysis to studies with very high study quality (overall score of 10 points)	38 (n=82900[c])	45007: 112120.80	0.35 (0.30 – 0.42)	0.12 – 1.02	0.243	11096.90	<0.001	100 (100 – 100)	0.269

[a]Missing information for 3 studies.
[b]Missing information for 2 studies.
[c]Missing information for 1 study.
Note: results from both the sensitivity analysis restricted to studies representing the national PD population and the sensitivity analysis restricted to studies achieving the maximum quality score of 10 points included the same set of studies.
Abbreviations: CI, confidence interval; NA, not applicable; PD, peritoneal dialysis.

Table S8 Meta-Analysis of Incidence Rate Estimation of PD-Associated Peritonitis with Calibration for Publication Bias[a]

Meta-Synthesis of Evidence	No. of Estimates	LFK Index[b]	*P* for Begg's Test	*P* for Egger's Test	No. of Imputed	Incidence Rate Estimation with Imputed (95% CI), Episode per Patient-Year	Observed Incidence Rate Estimation with Imputed (95% CI), Episode per Patient-Year	*P* for Between Observed vs Observed + Imputed
Overall global estimation	153	-1.80	0.359	<0.001	29	0.36 (0.33 – 0.40)	0.31 (0.28 – 0.34)	0.065
According to WHO region								
• African region	2	NA	NA	NA	NA	NA	0.99 (0.68 – 1.45)	NA
• Region of the Americas	21	-1.33	0.880	0.074	3	0.41 (0.35 – 0.47)	0.38 (0.31 – 0.45)	0.524
• South-East Asia region	16	-1.89	0.964	0.369	0	Not detected	0.41 (0.35 – 0.48)	NA
• European region	41	-2.71	0.387	0.028	9	0.44 (0.37 – 0.51)	0.37 (0.30 – 0.46)	0.197
• Eastern Mediterranean region	12	-1.56	0.304	0.768	0	Not detected	0.39 (0.31 – 0.49)	NA
• Western Pacific region	60	-3.16	0.588	<0.001	9	0.24 (0.21 – 0.26)	0.21 (0.19 – 0.24)	0.096
According to causative organism								
• Gram-positive bacteria	94	-4.80	0.398	<0.001	1	0.127 (0.111 – 0.145)	0.127 (0.113 – 0.142)	1.000
• Gram-negative bacteria	96	-4.27	0.152	<0.001	20	0.081 (0.070 – 0.094)	0.065 (0.058 – 0.073)	0.070
• Fungus	99	-4.22	0.005	<0.001	25	0.013 (0.011 – 0.017)	0.009 (0.008 – 0.011)	0.057
• Mycobacterium	29	-2.52	0.273	0.030	0	NA	0.004 (0.003 – 0.006)	NA
• Polymicrobial	55	-6.06	0.215	<0.001	15	0.025 (0.019 – 0.034)	0.017 (0.014 – 0.021)	0.058
• Culture negative	94	-2.13	0.371	0.002	21	0.093 (0.080 – 0.108)	0.073 (0.065 – 0.083)	0.068
Study population								
• Adult	86	-2.26	0.911	0.010	17	0.34 (0.30 – 0.38)	0.29 (0.26 – 0.32)	0.052
• Pediatric	7	-3.36	0.649	0.236	1	0.50 (0.37 – 0.67)	0.46 (0.36 – 0.58)	0.673
• Mixed	16	-1.55	0.300	0.030	0	Not detected	0.37 (0.28 – 0.48)	NA
• Not specified	44	-2.57	0.537	0.012	10	0.38 (0.31 – 0.45)	0.31 (0.25 – 0.38)	0.151

[a]Calibration for publication was carried out, if indicated in trim and fill analysis.
[b]For LFK Index, a value exceeding 1 or -1 was indicated to be publication bias.
Abbreviations: CI, confidence interval; LFK, Luis Furuya-Kanamori; NA, not applicable; PD, peritoneal dialysis; WHO, World Health Organization.

Table S8 Meta-Analysis of Incidence Rate Estimation of PD-Associated Peritonitis with Calibration for Publication Bias (Continued)[a]

Meta-Synthesis of Evidence	No. of Estimates	LFK Index[b]	*P* for Begg's Test	*P* for Egger's Test	No. of Imputed	Incidence Rate Estimation with Imputed (95% CI), Episode per Patient-Year	Observed Incidence Rate Estimation with Imputed (95% CI), Episode per Patient-Year	*P* for Between Observed vs Observed + Imputed
PD modality								
• CAPD	35	-1.20	0.887	0.133	3	0.34 (0.27 – 0.41)	0.31 (0.25 – 0.38)	0.538
• APD	2	NA	NA	NA	NA	NA	0.42 (0.16 – 1.12)	NA
• Mixed	82	-1.81	0.785	0.009	17	0.38 (0.35 – 0.42)	0.33 (0.29 – 0.37)	0.065
• Not specified	34	-3.50	0.929	0.052	0	Not detected	0.25 (0.20 – 0.30)	NA

[a]Calibration for publication was carried out, if indicated in trim and fill analysis.

[b]For LFK Index, a value exceeding 1 or -1 was indicated to be publication bias.

Abbreviations: APD, automated peritoneal dialysis; CAPD, continuous ambulatory peritoneal dialysis; CI, confidence interval; LFK, Luis Furuya-Kanamori; NA, not applicable; PD, peritoneal dialysis.

Figure S1 Meta-Analysis of Global Incidence Rate Estimation of PD-Associated Peritonitis

First_Author	Country	Sample_Size	Total_Episode	Total_PY	Episode per patient-year (IV, Random) (95% CI)	% Weight
Balasubramanian et al, 2011	United Kingdom	372	310	839.583	0.3692 [0.3303, 0.4127]	0.66
Brown et al, 2011	United Kingdom		1918	3175.5	0.6040 [0.5776, 0.6316]	0.67
Davenport et al, 2011	United Kingdom	4095	3322	5819.5	0.5708 [0.5518, 0.5906]	0.67
Huang et al, 2011	Taiwan	630	599	1688.4	0.3548 [0.3275, 0.3844]	0.66
Kanjanabuch et al, 2011	Thailand	8201	2420	5147.25	0.4702 [0.4518, 0.4893]	0.67
Liawnoraset, 2011	Thailand	318	128	271.85	0.4708 [0.3960, 0.5599]	0.65
Luvira et al, 2011	Thailand	333	633	732.639	0.8640 [0.7992, 0.9340]	0.66
Martin et al, 2011	Brazil	2032	637	1835.5	0.3470 [0.3211, 0.3751]	0.66
Muñoz de Bustillo et al, 2011	Spain	1515	1609	3213.7	0.5007 [0.4768, 0.5257]	0.67
Oygar et al, 2011	Türkiye	109	79	360.1	0.2194 [0.1760, 0.2735]	0.64
Quintanar Lartundo et al, 2011	Spain	411	229	346.97	0.6600 [0.5798, 0.7513]	0.66
Topanthanont et al, 2011	Thailand	103	112	436.5	0.2566 [0.2132, 0.3088]	0.65
Aksu et al, 2012	Türkiye	110	210	290.5	0.7229 [0.6314, 0.8276]	0.66
Barone et al, 2012	Argentina	118	157	441.417	0.3557 [0.3042, 0.4159]	0.65
Basturk et al, 2012	Türkiye	355	506	1070.17	0.4728 [0.4334, 0.5159]	0.66
Chen et al, 2012	Canada and United States	2829	2406	3995	0.6023 [0.5787, 0.6268]	0.67
Levallois et al, 2012	Canada	288	289	604.833	0.4778 [0.4258, 0.5362]	0.66
Nayak et al, 2012	India	246	116	358	0.3240 [0.2701, 0.3887]	0.65
Oliveira et al, 2012	Brazil	189	170	262.25	0.6482 [0.5578, 0.7534]	0.65
Pierce et al, 2012	United States	377	221	550	0.4018 [0.3522, 0.4584]	0.66
Rocha et al, 2012	Portugal	438	588	985.3	0.5968 [0.5504, 0.6470]	0.66
Su et al, 2012	China	158	46	290.833	0.1582 [0.1185, 0.2112]	0.62
Xu et al, 2012	China	124	248	1147	0.2162 [0.1909, 0.2449]	0.66
Xu et al, 2012	China	313	245	1133.13	0.2162 [0.1908, 0.2451]	0.66
Yap et al, 2012	Hong Kong	296	548	3023.58	0.1812 [0.1667, 0.1971]	0.66
Yap et al, 2012	Hong Kong	1285	1797	4059.25	0.4427 [0.4227, 0.4636]	0.67
Chern et al, 2013	Taiwan	404	288	1276.42	0.2256 [0.2010, 0.2533]	0.66
Huang et al, 2013	Taiwan	183	92	630.167	0.1460 [0.1190, 0.1791]	0.64
Kerschbaum et al, 2013	Austria	726	552	1703	0.3241 [0.2982, 0.3523]	0.66
Nessim et al, 2013	Canada	938	1338	2110.9	0.6339 [0.6008, 0.6687]	0.67
Özbalci et al, 2013	Türkiye	153	175	616.197	0.2840 [0.2449, 0.3294]	0.66
Portoles et al, 2013	Spain	1177	1091	1989.5	0.5484 [0.5168, 0.5819]	0.67
Tsai et al, 2013	Taiwan	349	204	1009.42	0.2021 [0.1762, 0.2318]	0.66
Hsieh et al, 2014	Taiwan	391	190	969.5	0.1960 [0.1700, 0.2259]	0.66
Isla et al, 2014	South Africa	152	210	256.098	0.8200 [0.7163, 0.9388]	0.66
Kumar et al, 2014	India	224	142	452.95	0.3135 [0.2660, 0.3695]	0.65
Najafi et al, 2014	Iran	2239	1125	2343.75	0.4800 [0.4528, 0.5089]	0.67
Nieto-Ríos et al, 2014	Colombia	1497	2469	3068	0.8048 [0.7736, 0.8371]	0.67
Nishina et al, 2014	Japan	192	155	752.667	0.2059 [0.1759, 0.2410]	0.65
Núñez-Moral et al, 2014	Spain	201	171	343.583	0.4977 [0.4284, 0.5782]	0.65
Prasad et al, 2014	India	560	303	742.33	0.4082 [0.3647, 0.4568]	0.66
Chang et al, 2015	South Korea	405	299	871.3	0.3432 [0.3064, 0.3844]	0.66
Courivaud et al, 2015	France	240	100	434.783	0.2300 [0.1891, 0.2798]	0.65
Figueiredo et al, 2015	Brazil	2243	736	2514.67	0.2927 [0.2723, 0.3146]	0.66
Lei et al, 2015	China	163	115	467.092	0.2462 [0.2051, 0.2956]	0.65
Ozisik et al, 2015	Türkiye	440	620	1001.08	0.6193 [0.5724, 0.6700]	0.66
Pi et al, 2015	China	346	96	467.04	0.2055 [0.1683, 0.2511]	0.65
Sanabria et al, 2015	Colombia	345	137	448.333	0.3056 [0.2585, 0.3613]	0.65
Buttigieg et al, 2016	Malta	137	144	369.231	0.3900 [0.3312, 0.4592]	0.65
Duquennoy et al, 2016	Multinational	8396	5958	12150.9	0.4903 [0.4780, 0.5029]	0.67
El-Reshaid et al, 2016	Kuwait	308	145	522.417	0.2776 [0.2359, 0.3266]	0.65
Higuchi et al, 2016	Japan	3042	544	2640.5	0.2060 [0.1894, 0.2241]	0.66
Kotera et al, 2016	Japan	119	63	261.5	0.2409 [0.1882, 0.3084]	0.63
Mizuno et al, 2016	Japan	1237	570	2147.76	0.2654 [0.2445, 0.2881]	0.66
Nakao et al, 2016	Japan	527	377	2268.28	0.1662 [0.1502, 0.1839]	0.66
Pai et al, 2016	Taiwan	149	60	375	0.1600 [0.1242, 0.2061]	0.63
Pérez-Fontan et al, 2016	Spain	691	578	1658.1	0.3486 [0.3213, 0.3782]	0.66
Perl J, et al	Canada	4247	2555	7026.25	0.3636 [0.3498, 0.3780]	0.67
Sandrini et al, 2016	Italy	178	91	383.583	0.2372 [0.1932, 0.2913]	0.64
Santhakumaran et al, 2016	United Kingdom	580	289	826.058	0.3499 [0.3118, 0.3926]	0.66
Shea et al, 2016	Hong Kong	114	44	113.667	0.3871 [0.2881, 0.5202]	0.62
Tian et al, 2016	China	294	489	2917.5	0.1676 [0.1534, 0.1831]	0.66
Alatab et al, 2017	Iran	134	183	723.583	0.2529 [0.2188, 0.2923]	0.66
Htay et al, 2017	Singapore	337	88	278.4	0.3161 [0.2565, 0.3895]	0.64
Lee et al, 2017	South Korea	295	138	1829.67	0.0754 [0.0638, 0.0891]	0.65
Ndlovu et al, 2017	South Africa	140	130	107.718	1.2069 [1.0162, 1.4332]	0.65
Ong et al, 2017	Malaysia	1603	354	1299	0.2725 [0.2456, 0.3024]	0.66
Vargas et al, 2017	Colombia	3525	176	752	0.2340 [0.2019, 0.2713]	0.66
Al Wakeel et al, 2018	Saudi Arabia	111	81	238.125	0.3402 [0.2736, 0.4229]	0.64
Fang et al, 2018	China	462	99	737	0.1343 [0.1103, 0.1636]	0.65
Hsu et al, 2018	Taiwan	398	316	1124.4	0.2810 [0.2517, 0.3138]	0.66
Ponce et al, 2018	Brazil	491	237	551.16	0.4300 [0.3786, 0.4884]	0.66
Rope et al, 2018	Sri Lanka	116	47	80.86	0.5813 [0.4367, 0.7736]	0.62
Alfayez et al, 2019	Saudi Arabia	100	90	212.75	0.4230 [0.3441, 0.5201]	0.64
Balzer et al, 2019	Germany	275	160	660.67	0.2422 [0.2074, 0.2828]	0.65
Einbinder et al, 2019	Israel	201	244	256.48	0.9513 [0.8392, 1.0785]	0.66
Gadola et al, 2019	Uruguay	219	171	469.08	0.3645 [0.3138, 0.4235]	0.65
Hu et al, 2019	China	730	436	1638.75	0.2661 [0.2422, 0.2922]	0.66
Jahromi et al, 2019	Iran	235	170	356.42	0.4770 [0.4104, 0.5543]	0.65
Karthikeyan et al, 2019	India	120	55	188.83	0.2913 [0.2236, 0.3794]	0.63
Lasfar et al, 2019	Tunisia	182	186	422.38	0.4404 [0.3814, 0.5084]	0.66
Liao et al, 2019	China	458	120	1185.2	0.1012 [0.0847, 0.1211]	0.65
Lichodziejewska-Niemierko et al, 2019	Poland	559	183	631.03	0.2900 [0.2509, 0.3352]	0.66
Wang et al, 2019	Taiwan	336	190	760	0.2500 [0.2169, 0.2882]	0.66
Wojtaszek et al, 2019	Poland	129	56	275.5	0.2033 [0.1564, 0.2641]	0.63
Bonnal et al, 2020	France	1035	565	1661.76	0.3400 [0.3131, 0.3692]	0.66
Hwang et al, 2020	South Korea	643	308	3537	0.0871 [0.0779, 0.0974]	0.66
Ma et al, 2020	China	357	509	1039.58	0.4896 [0.4489, 0.5341]	0.66
Noppakun et al, 2020	Thailand	1169	1257	3418.4	0.3677 [0.3479, 0.3886]	0.67
Perl et al, 2020	Australia/New Zealand	510	187	534.8	0.3497 [0.3030, 0.4035]	0.66
Perl et al, 2020	Canada	917	393	1376.8	0.2854 [0.2586, 0.3151]	0.66
Perl et al, 2020	United Kingdom	342	120	314.1	0.3820 [0.3195, 0.4569]	0.65
Perl et al, 2020	United States	3635	969	3754.6	0.2581 [0.2423, 0.2749]	0.67
Tekkarismaz et al, 2020	Türkiye	242	297	1996.5	0.1488 [0.1328, 0.1667]	0.66
Viglino et al, 2020	Italy	107	34	155.75	0.2183 [0.1560, 0.3055]	0.61
Zeng et al, 2020	China	909	667	3927.52	0.1698 [0.1574, 0.1832]	0.66
AlZabli et al, 2021	Saudi Arabia	131	208	305	0.6820 [0.5953, 0.7812]	0.66
de la Espada Pina et al, 2021	Spain	2904	2656	1115.42	2.3812 [2.2923, 2.4735]	0.67
Htay et al, 2021	Singapore	288	55	257.9	0.2133 [0.1637, 0.2778]	0.63
Hu et al, 2021	China	591	261	1535.29	0.1700 [0.1506, 0.1919]	0.66
Kang et al, 2021	South Korea	1203	879	4554.4	0.1930 [0.1807, 0.2062]	0.66
Li et al, 2021	China	739	268	2105	0.1273 [0.1130, 0.1435]	0.66
Liu et al, 2021	China	602	230	1486.67	0.1547 [0.1360, 0.1761]	0.66
Neu et al, 2021	United States	1055	648	1620.08	0.4000 [0.3703, 0.4320]	0.66
Oki et al, 2021	Japan	159	128	581.82	0.2200 [0.1850, 0.2616]	0.65
Sakurada et al, 2021	Japan	126	42	302	0.1391 [0.1028, 0.1882]	0.62
Santos et al, 2021	Spain	878	1061	2270.7	0.4673 [0.4400, 0.4962]	0.67
Song et al, 2021	China	180	101	517.95	0.1950 [0.1604, 0.2370]	0.65
Tangjittrong, 2021	Thailand	411	233	909.74	0.2561 [0.2253, 0.2912]	0.66
Witoon et al, 2021	Thailand	532	675	1350	0.5000 [0.4637, 0.5392]	0.66
Cheikh Hassan et al, 2022	Australia	9699	8936	24270	0.3682 [0.3606, 0.3759]	0.67
Kanjanabuch et al, 2022	Thailand	848	360	878.05	0.4100 [0.3698, 0.4546]	0.66
Kolland et al, 2022	Austria	250	155	662.7	0.2339 [0.1998, 0.2738]	0.65
Lau et al, 2022	Malaysia		906	3939.13	0.2300 [0.2155, 0.2455]	0.67
Melanson et al, 2022	Canada	124	80	240.787	0.3322 [0.2669, 0.4136]	0.64
Özbek et al, 2022	Türkiye	352	80	274.083	0.2919 [0.2344, 0.3634]	0.64
Szeto et al, 2022	Hong Kong	1193	3160	7707.32	0.4100 [0.3960, 0.4245]	0.67
Wu et al, 2022	China	2570	1597	9919.25	0.1610 [0.1533, 0.1691]	0.67
Zhai et al, 2022	China	283	117	408	0.2868 [0.2392, 0.3437]	0.65
Alves et al, 2023	Brazil	370	101	336.67	0.3000 [0.2468, 0.3646]	0.65
Cankaya et al, 2023	Türkiye	202	335	791	0.4235 [0.3805, 0.4714]	0.66
Chienwichai et al, 2023	Thailand	336	121	288.1	0.4200 [0.3514, 0.5019]	0.65
Forte et al, 2023	France	8848	4161	13023	0.3195 [0.3099, 0.3294]	0.67
Guo et al, 2023	China	101	52	288.89	0.1800 [0.1372, 0.2362]	0.63
Pak et al, 2023	Hong Kong	172	218	438.87	0.4967 [0.4350, 0.5672]	0.66
Rivera et al, 2023	Colombia	4743	807	3508.7	0.2300 [0.2147, 0.2464]	0.66
Srivastava et al, 2023	India	200	85	113.33	0.7500 [0.6064, 0.9277]	0.64
Taher et al, 2023	Palestine	158	48	152.8	0.3141 [0.2367, 0.4168]	0.62
Yang et al, 2023	Taiwan	2084	1996	8871.11	0.2250 [0.2153, 0.2351]	0.67
Ye et al, 2023	China	138	119	665.4	0.1788 [0.1494, 0.2140]	0.65
Aldriwesh et al, 2024	Saudi Arabia	100	93	166.07	0.5600 [0.4570, 0.6862]	0.64
Centellas-Pérez et al, 2024	Spain	232	40	160	0.2500 [0.1834, 0.3408]	0.62
Dandecha et al, 2024	Thailand	120	129	440	0.2932 [0.2467, 0.3484]	0.65
Do?an et al, 2024	Türkiye	120	53	120.45	0.4400 [0.3362, 0.5760]	0.63
Dong et al, 2024	China	3459	2242	14035.4	0.1597 [0.1533, 0.1665]	0.67
Gül, 2024	Türkiye	171	111	527.4	0.2105 [0.1747, 0.2535]	0.65
Ito et al, 2024	Japan	1225	539	1996.3	0.2700 [0.2481, 0.2938]	0.66
Liang et al, 2024	China	166	282	1944.83	0.1450 [0.1290, 0.1630]	0.66
Mehrotra et al, 2024	United States	243	54	180	0.3000 [0.2298, 0.3917]	0.63
Nabil et al, 2024	Morocco	235	378	782.417	0.4831 [0.4368, 0.5344]	0.66
Nardelli et al, 2024	Italy	267	219	876	0.2500 [0.2190, 0.2854]	0.66
Park et al, 2024	South Korea	184	210	750	0.2800 [0.2446, 0.3206]	0.66
Tobijaszewska et al, 2024	Sweden	675	484	1273.68	0.3800 [0.3476, 0.4154]	0.66
Alfano et al, 2025	Italy		345	1488.9	0.2317 [0.2085, 0.2575]	0.66
Andrew et al, 2025	United Kingdom	157	33	106.45	0.3100 [0.2204, 0.4361]	0.61
Guo et al, 2025	China	392	120	387.1	0.3100 [0.2592, 0.3707]	0.65
Kim et al, 2025	South Korea	766	142	1044.12	0.1360 [0.1154, 0.1603]	0.65
Soetendorp et al, 2025	Israel	113	39	133.929	0.2912 [0.2128, 0.3986]	0.61
Tsuchiya et al, 2025	Japan	180	254	1243.78	0.2042 [0.1806, 0.2309]	0.66
Yu et al, 2025	China	506	118	681.75	0.1731 [0.1445, 0.2073]	0.65
Al Laham et al, 2026	Oman	311	490	2333.33	0.2100 [0.1922, 0.2294]	0.66
Cho et al, 2026	South Korea	162	157	585	0.2684 [0.2295, 0.3138]	0.65
Okamoto et al, 2026	Japan	105	84	646.15	0.1300 [0.1050, 0.1610]	0.64
Overall (I-squared = 99.4%, p = 0.000)					0.3077 [0.2819, 0.3359]	100.00
with estimated predictive interval					. (0.1040, 0.9107)	

NOTE: Weights are from random effects analysis

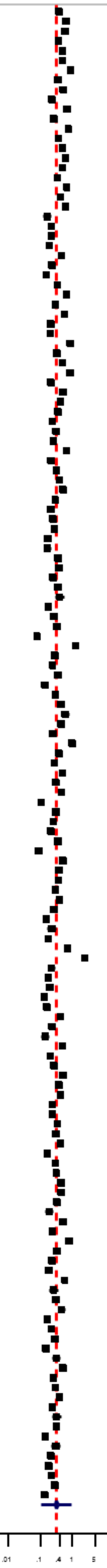

Figure S2 Meta-Analysis of Incidence Rate Estimation of PD-Associated Peritonitis, by WHO Regions

A. African Region

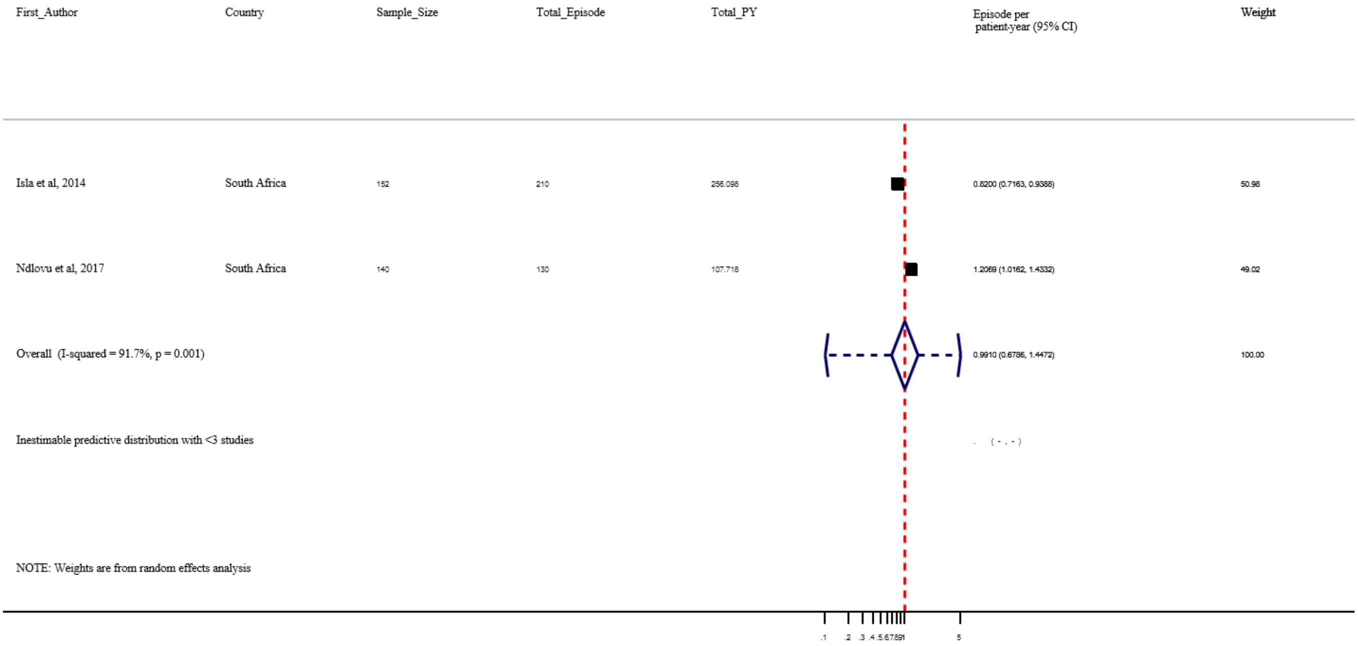

Figure S2 Meta-Analysis of Incidence Rate Estimation of PD-Associated Peritonitis, by WHO Regions (Continued)

B. Region of the Americas

First_Author	Country	Sample_Size	Total_Episode	Total_PY	Episode per patient-year (95% CI)	% Weight
Martin et al, 2011	Brazil	2032	637	1835.5	0.3470 (0.3211, 0.3751)	4.84
Barone et al, 2012	Argentina	118	157	441.417	0.3557 (0.3042, 0.4159)	4.71
Chen et al, 2012	Canada and United States	2829	2406	3995	0.6023 (0.5787, 0.6268)	4.87
Levallois et al, 2012	Canada	288	289	604.833	0.4778 (0.4258, 0.5362)	4.79
Oliveira et al, 2012	Brazil	189	170	262.25	0.6482 (0.5578, 0.7534)	4.73
Pierce et al, 2012	United States	377	221	550	0.4018 (0.3522, 0.4584)	4.76
Nessim et al, 2013	Canada	938	1338	2110.9	0.6339 (0.6008, 0.6687)	4.86
Nieto-Ríos et al, 2014	Colombia	1497	2469	3068	0.8048 (0.7736, 0.8371)	4.87
Figueiredo et al, 2015	Brazil	2243	736	2514.67	0.2927 (0.2723, 0.3146)	4.84
Sanabria et al, 2015	Colombia	345	137	448.333	0.3056 (0.2585, 0.3613)	4.69
Perl J, et al	Canada	4247	2555	7026.25	0.3636 (0.3498, 0.3780)	4.87
Vargas et al, 2017	Colombia	3525	176	752	0.2340 (0.2019, 0.2713)	4.73
Ponce et al, 2018	Brazil	491	237	551.16	0.4300 (0.3786, 0.4884)	4.77
Gadola et al, 2019	Uruguay	219	171	469.08	0.3645 (0.3138, 0.4235)	4.73
Perl et al, 2020	Canada	917	393	1376.8	0.2854 (0.2586, 0.3151)	4.81
Perl et al, 2020	United States	3635	969	3754.6	0.2581 (0.2423, 0.2749)	4.85
Neu et al, 2021	United States	1055	648	1620.08	0.4000 (0.3703, 0.4320)	4.84
Melanson et al, 2022	Canada	124	80	240.787	0.3322 (0.2669, 0.4136)	4.56
Alves et al, 2023	Brazil	370	101	336.67	0.3000 (0.2468, 0.3646)	4.63
Rivera et al, 2023	Colombia	4743	807	3508.7	0.2300 (0.2147, 0.2464)	4.84
Mehrotra et al, 2024	United States	243	54	180	0.3000 (0.2298, 0.3917)	4.43
Overall (I-squared = 99.2%, p = 0.000)					0.3759 (0.3125, 0.4522)	100.00
with estimated predictive interval					. (0.1506, 0.9386)	

NOTE: Weights are from random effects analysis

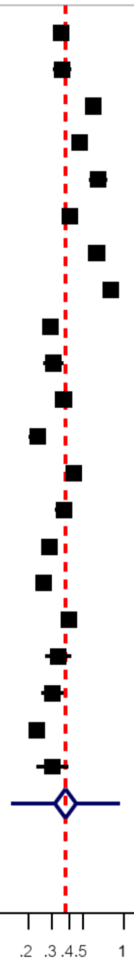

Figure S2 Meta-Analysis of Incidence Rate Estimation of PD-Associated Peritonitis, by WHO Regions (Continued)

C. South-East Asia Region

First_Author	Country	Sample_Size	Total_Episode	Total_PY	Episode per patient-year (95% CI)	% Weight
Kanjanabuch et al, 2011	Thailand	8201	2420	5147.25	0.4702 (0.4518, 0.4893)	6.67
Liawnoraset, 2011	Thailand	318	128	271.85	0.4708 (0.3960, 0.5599)	6.19
Luvira et al, 2011	Thailand	333	633	732.639	0.8640 (0.7992, 0.9340)	6.59
Topanthanont et al, 2011	Thailand	103	112	436.5	0.2566 (0.2132, 0.3088)	6.12
Nayak et al, 2012	India	246	116	358	0.3240 (0.2701, 0.3887)	6.14
Kumar et al, 2014	India	224	142	452.95	0.3135 (0.2660, 0.3695)	6.24
Prasad et al, 2014	India	560	303	742.33	0.4082 (0.3647, 0.4568)	6.48
Rope et al, 2018	Sri Lanka	116	47	80.86	0.5813 (0.4367, 0.7736)	5.47
Karthikeyan et al, 2019	India	120	55	188.83	0.2913 (0.2236, 0.3794)	5.62
Noppakun et al, 2020	Thailand	1169	1257	3418.4	0.3677 (0.3479, 0.3886)	6.65
Tangjittrong, 2021	Thailand	411	233	909.74	0.2561 (0.2253, 0.2912)	6.41
Witoon et al, 2021	Thailand	532	675	1350	0.5000 (0.4637, 0.5392)	6.60
Kanjanabuch et al, 2022	Thailand	848	360	878.05	0.4100 (0.3698, 0.4546)	6.51
Chienwichai et al, 2023	Thailand	336	121	288.1	0.4200 (0.3514, 0.5019)	6.16
Srivastava et al, 2023	India	200	85	113.33	0.7500 (0.6064, 0.9277)	5.96
Dandecha et al, 2024	Thailand	120	129	440	0.2932 (0.2467, 0.3484)	6.19
Overall (I-squared = 97.2%, p = 0.000)					0.4096 (0.3504, 0.4787)	100.00
with estimated predictive interval					. (0.2072, 0.8095)	

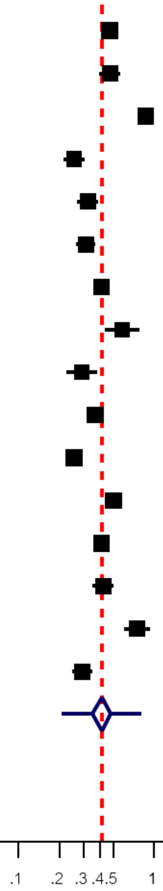

NOTE: Weights are from random effects analysis

Figure S2 Meta-Analysis of Incidence Rate Estimation of PD-Associated Peritonitis, by WHO Regions (Continued)

D. European Region

First_Author	Country	Sample_Size	Total_Episode	Total_PY	Episode per patient-year (95% CI)	% Weight
Balasubramanian et al, 2011	United Kingdom	372	310	839.583	0.3692 (0.3303, 0.4127)	2.46
Brown et al, 2011	United Kingdom		1918	3175.5	0.6040 (0.5776, 0.6316)	2.48
Davenport et al, 2011	United Kingdom	4095	3322	5819.5	0.5708 (0.5518, 0.5906)	2.48
Muñoz de Bustillo et al, 2011	Spain	1515	1609	3213.7	0.5007 (0.4768, 0.5257)	2.48
Oygar et al, 2011	Türkiye	109	79	360.1	0.2194 (0.1760, 0.2735)	2.41
Quintanar Lartundo et al, 2011	Spain	411	229	346.97	0.6600 (0.5798, 0.7513)	2.45
Aksu et al, 2012	Türkiye	110	210	290.5	0.7229 (0.6314, 0.8276)	2.45
Basturk et al, 2012	Türkiye	355	506	1070.17	0.4728 (0.4334, 0.5159)	2.47
Rocha et al, 2012	Portugal	438	588	985.3	0.5968 (0.5504, 0.6470)	2.47
Kerschbaum et al, 2013	Austria	726	552	1703	0.3241 (0.2982, 0.3523)	2.47
Özbalci et al, 2013	Türkiye	153	175	616.197	0.2840 (0.2449, 0.3294)	2.45
Portoles et al, 2013	Spain	1177	1091	1989.5	0.5484 (0.5168, 0.5819)	2.47
Núñez-Moral et al, 2014	Spain	201	171	343.583	0.4977 (0.4284, 0.5782)	2.45
Courivaud et al, 2015	France	240	100	434.783	0.2300 (0.1891, 0.2798)	2.42
Ozisik et al, 2015	Türkiye	440	620	1001.08	0.6193 (0.5724, 0.6700)	2.47
Buttigieg et al, 2016	Malta	137	144	369.231	0.3900 (0.3312, 0.4592)	2.44
Pérez-Fontan et al, 2016	Spain	691	578	1658.1	0.3486 (0.3213, 0.3782)	2.47
Sandrini et al, 2016	Italy	178	91	383.583	0.2372 (0.1932, 0.2913)	2.42
Santhakumaran et al, 2016	United Kingdom	580	289	826.058	0.3499 (0.3118, 0.3926)	2.46
Balzer et al, 2019	Germany	275	160	660.67	0.2422 (0.2074, 0.2828)	2.44
Einbinder et al, 2019	Israel	201	244	256.48	0.9513 (0.8392, 1.0785)	2.46
Lichodziejewska-Niemierko et al, 2019	Poland	559	183	631.03	0.2900 (0.2509, 0.3352)	2.45
Wojtaszek et al, 2019	Poland	129	56	275.5	0.2033 (0.1564, 0.2641)	2.38
Bonnal et al, 2020	France	1035	565	1661.76	0.3400 (0.3131, 0.3692)	2.47
Perl et al, 2020	United Kingdom	342	120	314.1	0.3820 (0.3195, 0.4569)	2.43
Tekkarismaz et al, 2020	Türkiye	242	297	1996.5	0.1488 (0.1328, 0.1667)	2.46
Viglino et al, 2020	Italy	107	34	155.75	0.2183 (0.1560, 0.3055)	2.32
de la Espada Pina et al, 2021	Spain	2904	2656	1115.42	2.3812 (2.2923, 2.4735)	2.48
Santos et al, 2021	Spain	878	1061	2270.7	0.4673 (0.4400, 0.4962)	2.47
Kolland et al, 2022	Austria	250	155	662.7	0.2339 (0.1998, 0.2738)	2.44
Özbek et al, 2022	Türkiye	352	80	274.083	0.2919 (0.2344, 0.3634)	2.41
Cankaya et al, 2023	Türkiye	202	335	791	0.4235 (0.3805, 0.4714)	2.46
Forte et al, 2023	France	8848	4161	13023	0.3195 (0.3099, 0.3294)	2.48
Centellas-Pérez et al, 2024	Spain	232	40	160	0.2500 (0.1834, 0.3408)	2.34
Do?an et al, 2024	Türkiye	120	53	120.45	0.4400 (0.3362, 0.5760)	2.37
Gül, 2024	Türkiye	171	111	527.4	0.2105 (0.1747, 0.2535)	2.43
Nardelli et al, 2024	Italy	267	219	876	0.2500 (0.2190, 0.2854)	2.45
Tobijaszewska et al, 2024	Sweden	675	484	1273.68	0.3800 (0.3476, 0.4154)	2.47
Alfano et al, 2025	Italy		345	1488.9	0.2317 (0.2085, 0.2575)	2.46
Andrew et al, 2025	United Kingdom	157	33	106.45	0.3100 (0.2204, 0.4361)	2.31
Soetendorp et al, 2025	Israel	113	39	133.929	0.2912 (0.2128, 0.3986)	2.34
Overall (I-squared = 99.6%, p = 0.000)					0.3718 (0.3040, 0.4546)	100.00
with estimated predictive interval					. (0.0978, 1.4128)	

NOTE: Weights are from random effects analysis

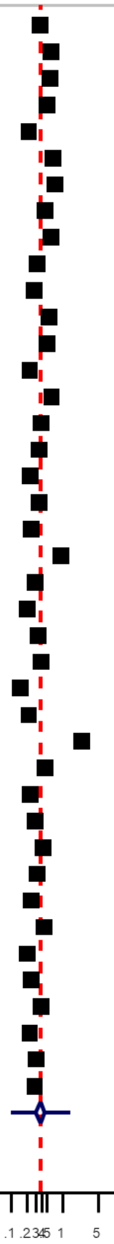

Figure S2 Meta-Analysis of Incidence Rate Estimation of PD-Associated Peritonitis, by WHO Regions (Continued)

E. Eastern Mediterranean Region

First_Author	Country	Sample_Size	Total_Episode	Total_PY	Episode per patient-year (95% CI)	% Weight
Najafi et al, 2014	Iran	2239	1125	2343.75	0.4800 (0.4528, 0.5089)	8.69
El-Reshaid et al, 2016	Kuwait	308	145	522.417	0.2776 (0.2359, 0.3266)	8.35
Alatab et al, 2017	Iran	134	183	723.583	0.2529 (0.2188, 0.2923)	8.43
Al Wakeel et al, 2018	Saudi Arabia	111	81	238.125	0.3402 (0.2736, 0.4229)	8.06
Alfayez et al, 2019	Saudi Arabia	100	90	212.75	0.4230 (0.3441, 0.5201)	8.13
Jahromi et al, 2019	Iran	235	170	356.42	0.4770 (0.4104, 0.5543)	8.41
Lasfar et al, 2019	Tunisia	182	186	422.38	0.4404 (0.3814, 0.5084)	8.44
AlZabli et al, 2021	Saudi Arabia	131	208	305	0.6820 (0.5953, 0.7812)	8.47
Taher et al, 2023	Palestine	158	48	152.8	0.3141 (0.2367, 0.4168)	7.65
Aldriwesh et al, 2024	Saudi Arabia	100	93	166.07	0.5600 (0.4570, 0.6862)	8.15
Nabil et al, 2024	Morocco	235	378	782.417	0.4831 (0.4368, 0.5344)	8.59
Al Laham et al, 2026	Oman	311	490	2333.33	0.2100 (0.1922, 0.2294)	8.63
Overall (I-squared = 97.2%, p = 0.000)					0.3904 (0.3129, 0.4873)	100.00
with estimated predictive interval					. (0.1607, 0.9488)	

NOTE: Weights are from random effects analysis

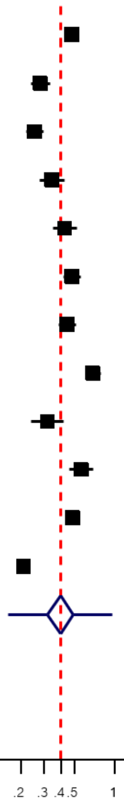

Figure S2 Meta-Analysis of Incidence Rate Estimation of PD-Associated Peritonitis, by WHO Regions (Continued)

F. Western Pacific Region

First_Author	Country	Sample_Size	Total_Episode	Total_PY	Episode per patient-year (95% CI)	% Weight
Huang et al, 2011	Taiwan	630	599	1688.4	0.3548 (0.3275, 0.3844)	1.71
Su et al, 2012	China	158	46	290.833	0.1582 (0.1185, 0.2112)	1.54
Xu et al, 2012	China	124	248	1147	0.2162 (0.1909, 0.2449)	1.69
Xu et al, 2012	China	313	245	1133.13	0.2162 (0.1908, 0.2451)	1.69
Yap et al, 2012	Hong Kong	296	548	3023.58	0.1812 (0.1667, 0.1971)	1.71
Yap et al, 2012	Hong Kong	1285	1797	4059.25	0.4427 (0.4227, 0.4636)	1.72
Chern et al, 2013	Taiwan	404	288	1276.42	0.2256 (0.2010, 0.2533)	1.69
Huang et al, 2013	Taiwan	183	92	630.167	0.1460 (0.1190, 0.1791)	1.63
Tsai et al, 2013	Taiwan	349	204	1009.42	0.2021 (0.1762, 0.2318)	1.68
Hsieh et al, 2014	Taiwan	391	190	969.5	0.1960 (0.1700, 0.2259)	1.68
Nishina et al, 2014	Japan	192	155	752.667	0.2059 (0.1759, 0.2410)	1.66
Chang et al, 2015	South Korea	405	299	871.3	0.3432 (0.3064, 0.3844)	1.69
Lei et al, 2015	China	163	115	467.092	0.2462 (0.2051, 0.2956)	1.64
Pi et al, 2015	China	346	96	467.04	0.2055 (0.1683, 0.2511)	1.63
Higuchi et al, 2016	Japan	3042	544	2640.5	0.2060 (0.1894, 0.2241)	1.71
Kotera et al, 2016	Japan	119	63	261.5	0.2409 (0.1882, 0.3084)	1.58
Mizuno et al, 2016	Japan	1237	570	2147.76	0.2654 (0.2445, 0.2881)	1.71
Nakao et al, 2016	Japan	527	377	2268.28	0.1662 (0.1502, 0.1839)	1.70
Pai et al, 2016	Taiwan	149	60	375	0.1600 (0.1242, 0.2061)	1.58
Shea et al, 2016	Hong Kong	114	44	113.667	0.3871 (0.2881, 0.5202)	1.53
Tian et al, 2016	China	294	489	2917.5	0.1676 (0.1534, 0.1831)	1.71
Htay et al, 2017	Singapore	337	88	278.4	0.3161 (0.2565, 0.3895)	1.62
Lee et al, 2017	South Korea	295	138	1829.67	0.0754 (0.0638, 0.0891)	1.66
Ong et al, 2017	Malaysia	1603	354	1299	0.2725 (0.2456, 0.3024)	1.70
Fang et al, 2018	China	462	99	737	0.1343 (0.1103, 0.1636)	1.63
Hsu et al, 2018	Taiwan	398	316	1124.4	0.2810 (0.2517, 0.3138)	1.70
Hu et al, 2019	China	730	436	1638.75	0.2661 (0.2422, 0.2922)	1.70
Liao et al, 2019	China	458	120	1185.2	0.1012 (0.0847, 0.1211)	1.65
Wang et al, 2019	Taiwan	336	190	760	0.2500 (0.2169, 0.2882)	1.68
Hwang et al, 2020	South Korea	643	308	3537	0.0871 (0.0779, 0.0974)	1.69
Ma et al, 2020	China	357	509	1039.58	0.4896 (0.4489, 0.5341)	1.71
Perl et al, 2020	Australia/New Zealand	510	187	534.8	0.3497 (0.3030, 0.4035)	1.68
Zeng et al, 2020	China	909	667	3927.52	0.1698 (0.1574, 0.1832)	1.71
Htay et al, 2021	Singapore	288	55	257.9	0.2133 (0.1637, 0.2778)	1.56
Hu et al, 2021	China	591	261	1535.29	0.1700 (0.1506, 0.1919)	1.69
Kang et al, 2021	South Korea	1203	879	4554.4	0.1930 (0.1807, 0.2062)	1.71
Li et al, 2021	China	739	268	2105	0.1273 (0.1130, 0.1435)	1.69
Liu et al, 2021	China	602	230	1486.67	0.1547 (0.1360, 0.1761)	1.68
Oki et al, 2021	Japan	159	128	581.82	0.2200 (0.1850, 0.2616)	1.65
Sakurada et al, 2021	Japan	126	42	302	0.1391 (0.1028, 0.1882)	1.52
Song et al, 2021	China	180	101	517.95	0.1950 (0.1604, 0.2370)	1.63
Cheikh Hassan et al, 2022	Australia	9699	8936	24270	0.3682 (0.3606, 0.3759)	1.72
Lau et al, 2022	Malaysia		906	3939.13	0.2300 (0.2155, 0.2455)	1.71
Szeto et al, 2022	Hong Kong	1193	3160	7707.32	0.4100 (0.3960, 0.4245)	1.72
Wu et al, 2022	China	2570	1597	9919.25	0.1610 (0.1533, 0.1691)	1.72
Zhai et al, 2022	China	283	117	408	0.2868 (0.2392, 0.3437)	1.65
Guo et al, 2023	China	101	52	288.89	0.1800 (0.1372, 0.2362)	1.56
Pak et al, 2023	Hong Kong	172	218	438.87	0.4967 (0.4350, 0.5672)	1.68
Yang et al, 2023	Taiwan	2084	1996	8871.11	0.2250 (0.2153, 0.2351)	1.72
Ye et al, 2023	China	138	119	665.4	0.1788 (0.1494, 0.2140)	1.65
Dong et al, 2024	China	3459	2242	14035.4	0.1597 (0.1533, 0.1665)	1.72
Ito et al, 2024	Japan	1225	539	1996.3	0.2700 (0.2481, 0.2938)	1.71
Liang et al, 2024	China	166	282	1944.83	0.1450 (0.1290, 0.1630)	1.69
Park et al, 2024	South Korea	184	210	750	0.2800 (0.2446, 0.3206)	1.68
Guo et al, 2025	China	392	120	387.1	0.3100 (0.2592, 0.3707)	1.65
Kim et al, 2025	South Korea	766	142	1044.12	0.1360 (0.1154, 0.1603)	1.66
Tsuchiya et al, 2025	Japan	180	254	1243.78	0.2042 (0.1806, 0.2309)	1.69
Yu et al, 2025	China	506	118	681.75	0.1731 (0.1445, 0.2073)	1.65
Cho et al, 2026	South Korea	162	157	585	0.2684 (0.2295, 0.3138)	1.67
Okamoto et al, 2026	Japan	105	84	646.15	0.1300 (0.1050, 0.1610)	1.62
Overall (I-squared = 98.9%, p = 0.000)					0.2127 (0.1909, 0.2370)	100.00
with estimated predictive interval					. (0.0910, 0.4974)	

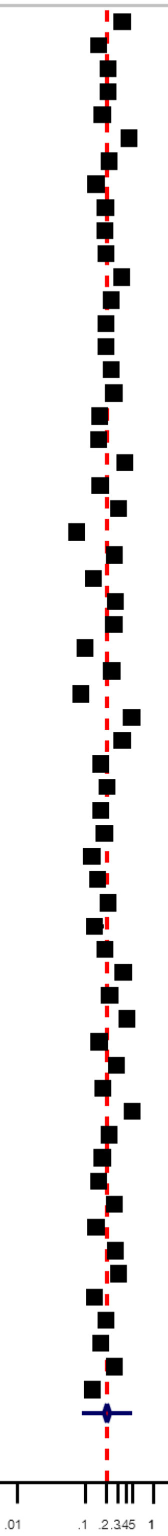

NOTE: Weights are from random effects analysis

Figure S3 Meta-Analysis of Incidence Rate Estimation of PD-Associated Peritonitis, by Causative Organisms

A. Gram-Positive Bacteria

First_Author	Country	Sample_Size	Gram_Positive	Total_PY	Episode per patient-year (95% CI)	% Weight
Huang et al, 2011	Taiwan	630	256	1688.4	0.1516 (0.1341, 0.1714)	1.12
Liawnoraset, 2011	Thailand	318	31	271.85	0.1140 (0.0802, 0.1621)	1.02
Luvira et al, 2011	Thailand	333	142	732.639	0.1938 (0.1644, 0.2285)	1.11
Martin et al, 2011	Brazil	2032	161	1835.5	0.0877 (0.0752, 0.1024)	1.11
Muñoz de Bustillo et al, 2011	Spain	1515	902	3213.7	0.2807 (0.2629, 0.2996)	1.13
Quintanar Lartundo et al, 2011	Spain	411	148	346.97	0.4266 (0.3631, 0.5011)	1.11
Topanthanont et al, 2011	Thailand	103	37	436.5	0.0848 (0.0614, 0.1170)	1.04
Oliveira et al, 2012	Brazil	189	82	262.25	0.3127 (0.2518, 0.3882)	1.09
Pierce et al, 2012	United States	377	104	550	0.1891 (0.1560, 0.2292)	1.10
Rocha et al, 2012	Portugal	438	286	985.3	0.2903 (0.2585, 0.3259)	1.12
Su et al, 2012	China	158	17	290.833	0.0585 (0.0363, 0.0940)	0.94
Xu et al, 2012	China	313	103	1133.13	0.0909 (0.0749, 0.1103)	1.10
Kerschbaum et al, 2013	Austria	726	335	1703	0.1967 (0.1767, 0.2189)	1.12
Nessim et al, 2013	Canada	938	735	2110.9	0.3482 (0.3239, 0.3743)	1.13
Özbalci et al, 2013	Türkiye	153	79	616.197	0.1282 (0.1028, 0.1598)	1.09
Tsai et al, 2013	Taiwan	349	84	1009.42	0.0832 (0.0672, 0.1031)	1.09
Hsieh et al, 2014	Taiwan	391	78	969.5	0.0805 (0.0644, 0.1004)	1.08
Isla et al, 2014	South Africa	152	40	256.098	0.1562 (0.1146, 0.2129)	1.04
Najafi et al, 2014	Iran	2239	341	2343.75	0.1455 (0.1308, 0.1618)	1.12
Nieto-Ríos et al, 2014	Colombia	1497	1180	3068	0.3846 (0.3633, 0.4072)	1.13
Nishina et al, 2014	Japan	192	106	752.667	0.1408 (0.1164, 0.1704)	1.10
Prasad et al, 2014	India	560	102	742.33	0.1374 (0.1132, 0.1668)	1.10
Courivaud et al, 2015	France	240	63	434.783	0.1449 (0.1132, 0.1855)	1.07
Ozisik et al, 2015	Türkiye	440	233	1001.08	0.2327 (0.2047, 0.2646)	1.12
Pi et al, 2015	China	346	30	467.04	0.0642 (0.0449, 0.0919)	1.02
Buttigieg et al, 2016	Malta	137	79	369.231	0.2140 (0.1716, 0.2667)	1.09
Duquennoy et al, 2016	Multinational	8396	2784	12150.9	0.2291 (0.2208, 0.2378)	1.13
El-Reshaid et al, 2016	Kuwait	308	61	522.417	0.1168 (0.0909, 0.1501)	1.07
Higuchi et al, 2016	Japan	3042	270	2640.5	0.1023 (0.0908, 0.1152)	1.12
Kotera et al, 2016	Japan	119	23	261.5	0.0880 (0.0584, 0.1324)	0.98
Mizuno et al, 2016	Japan	1237	263	2147.76	0.1225 (0.1085, 0.1382)	1.12
Nakao et al, 2016	Japan	527	168	2268.28	0.0741 (0.0637, 0.0862)	1.11
Pèrez-Fontan et al, 2016	Spain	691	282	1658.1	0.1701 (0.1513, 0.1911)	1.12
Santhakumaran et al, 2016	United Kingdom	580	164	826.058	0.1985 (0.1704, 0.2314)	1.11
Shea et al, 2016	Hong Kong	114	12	113.667	0.1056 (0.0600, 0.1859)	0.88
Tian et al, 2016	China	294	115	2917.5	0.0394 (0.0328, 0.0473)	1.10
Lee et al, 2017	South Korea	295	33	1829.67	0.0180 (0.0128, 0.0254)	1.02
Ndlovu et al, 2017	South Africa	140	52	107.718	0.4827 (0.3679, 0.6335)	1.06
Ong et al, 2017	Malaysia	1603	88	1299	0.0677 (0.0550, 0.0835)	1.09
Vargas et al, 2017	Colombia	3525	69	752	0.0918 (0.0725, 0.1162)	1.08
Al Wakeel et al, 2018	Saudi Arabia	111	40	238.125	0.1680 (0.1232, 0.2290)	1.04
Fang et al, 2018	China	462	28	737	0.0380 (0.0262, 0.0550)	1.01
Hsu et al, 2018	Taiwan	398	129	1124.4	0.1147 (0.0965, 0.1363)	1.10
Ponce et al, 2018	Brazil	491	30	551.16	0.0544 (0.0381, 0.0778)	1.02
Alfayez et al, 2019	Saudi Arabia	100	37	212.75	0.1739 (0.1260, 0.2400)	1.04
Einbinder et al, 2019	Israel	201	121	256.48	0.4718 (0.3948, 0.5638)	1.10
Gadola et al, 2019	Uruguay	219	84	469.08	0.1791 (0.1446, 0.2218)	1.09
Karthikeyan et al, 2019	India	120	4	188.83	0.0212 (0.0080, 0.0564)	0.61
Liao et al, 2019	China	458	52	1185.2	0.0439 (0.0334, 0.0576)	1.06
Wang et al, 2019	Taiwan	336	100	760	0.1316 (0.1082, 0.1601)	1.10
Hwang et al, 2020	South Korea	643	123	3537	0.0348 (0.0291, 0.0415)	1.10
Noppakun et al, 2020	Thailand	1169	435	3418.4	0.1273 (0.1158, 0.1398)	1.12
Perl et al, 2020	Australia/New Zealand	510	73	534.8	0.1365 (0.1085, 0.1717)	1.08
Perl et al, 2020	Canada	917	177	1376.8	0.1286 (0.1109, 0.1490)	1.11
Perl et al, 2020	United Kingdom	342	46	314.1	0.1465 (0.1097, 0.1955)	1.05
Perl et al, 2020	United States	3635	358	3754.6	0.0953 (0.0860, 0.1058)	1.12
Zeng et al, 2020	China	909	326	3927.52	0.0830 (0.0745, 0.0925)	1.12
AlZabli et al, 2021	Saudi Arabia	131	98	305	0.3213 (0.2636, 0.3917)	1.09
Htay et al, 2021	Singapore	288	19	257.9	0.0737 (0.0470, 0.1155)	0.96
Neu et al, 2021	United States	1055	259	1620.08	0.1599 (0.1415, 0.1806)	1.12
Oki et al, 2021	Japan	159	53	581.82	0.0911 (0.0696, 0.1192)	1.06
Sakurada et al, 2021	Japan	126	14	302	0.0464 (0.0275, 0.0783)	0.91
Witoon et al, 2021	Thailand	532	232	1350	0.1719 (0.1511, 0.1955)	1.12
Kanjanabuch et al, 2022	Thailand	848	111	878.05	0.1264 (0.1050, 0.1523)	1.10
Kolland et al, 2022	Austria	250	69	662.7	0.1041 (0.0822, 0.1318)	1.08
Özbek et al, 2022	Türkiye	352	47	274.083	0.1715 (0.1288, 0.2282)	1.05
Szeto et al, 2022	Hong Kong	1193	1284	7707.32	0.1666 (0.1577, 0.1760)	1.13
Wu et al, 2022	China	2570	526	9919.25	0.0530 (0.0487, 0.0578)	1.13
Zhai et al, 2022	China	283	39	408	0.0956 (0.0698, 0.1308)	1.04
Chienwichai et al, 2023	Thailand	336	10	288.1	0.0347 (0.0187, 0.0645)	0.84
Forte et al, 2023	France	8848	1943	13023	0.1492 (0.1427, 0.1560)	1.13
Pak et al, 2023	Hong Kong	172	98	438.87	0.2233 (0.1832, 0.2722)	1.09
Srivastava et al, 2023	India	200	1	113.33	0.0088 (0.0012, 0.0626)	0.25
Aldriwesh et al, 2024	Saudi Arabia	100	57	166.07	0.3432 (0.2648, 0.4450)	1.07
Dandecha et al, 2024	Thailand	120	48	440	0.1091 (0.0822, 0.1448)	1.06
Do?an et al, 2024	Türkiye	120	34	120.45	0.2823 (0.2017, 0.3950)	1.03
Gül, 2024	Türkiye	171	68	527.4	0.1289 (0.1017, 0.1635)	1.08
Ito et al, 2024	Japan	1225	210	1996.3	0.1052 (0.0919, 0.1204)	1.11
Liang et al, 2024	China	166	135	1944.83	0.0694 (0.0586, 0.0822)	1.10
Nardelli et al, 2024	Italy	267	106	876	0.1210 (0.1000, 0.1464)	1.10
Park et al, 2024	South Korea	184	109	750	0.1453 (0.1205, 0.1753)	1.10
Tobijaszewska et al, 2024	Sweden	675	341	1273.68	0.2677 (0.2408, 0.2977)	1.12
Alfano et al, 2025	Italy		156	1488.9	0.1048 (0.0896, 0.1226)	1.11
Kim et al, 2025	South Korea	766	58	1044.12	0.0555 (0.0429, 0.0719)	1.07
Soetendorp et al, 2025	Israel	113	21	133.929	0.1568 (0.1022, 0.2405)	0.97
Tsuchiya et al, 2025	Japan	180	141	1243.78	0.1134 (0.0961, 0.1337)	1.11
Yu et al, 2025	China	506	62	681.75	0.0909 (0.0709, 0.1166)	1.07
Cho et al, 2026	South Korea	162	71	585	0.1214 (0.0962, 0.1532)	1.08
Okamoto et al, 2026	Japan	105	43	646.15	0.0665 (0.0494, 0.0897)	1.05
Cho Y, et al (Supplement to Cheikh Hassan HI, 2022)	Australia	6610	3413	10470	0.3260 (0.3152, 0.3371)	1.13
Wang J, et al (Supplement to Hu J, 2021)	China	354	50	600.117	0.0833 (0.0631, 0.1099)	1.06
Hu S, et al (Supplement to Hu S, 2019)	China	278	115	807.41	0.1424 (0.1186, 0.1710)	1.10
Jegatheesan D, et al (Supplement to Cheikh Hassan HI, 2022)	Australia	8343	3900	14688	0.2655 (0.2573, 0.2740)	1.13
Hayat A, et al (Supplement to Cheikh Hassan HI, 2022)	Australia	665	594	2084.91	0.2849 (0.2629, 0.3088)	1.13
Overall (I-squared = 98.7%, p = 0.000)					0.1267 (0.1133, 0.1417)	100.00
with estimated predictive interval					. (0.0435, 0.3689)	

NOTE: Weights are from random effects analysis

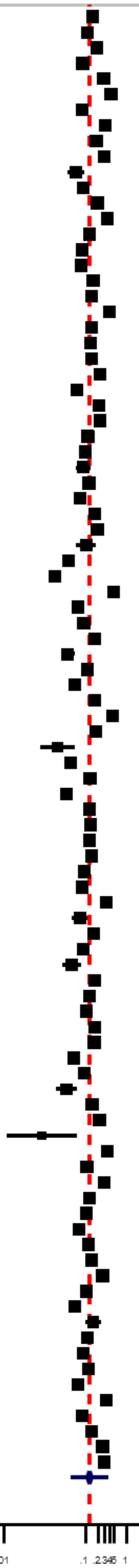

Figure S3 Meta-Analysis of Incidence Rate Estimation of PD-Associated Peritonitis, by Causative Organisms (Continued)

B. Gram-Negative Bacteria

First_Author	Country	Sample_Size	Gram_Negative	Total_PY	Episode per patient-year (95% CI)	% Weight
Brown et al, 2011	United Kingdom		190	3175.5	0.0598 (0.0519, 0.0690)	1.13
Huang et al, 2011	Taiwan	630	132	1688.4	0.0782 (0.0659, 0.0927)	1.12
Liawnoraset, 2011	Thailand	318	20	271.85	0.0736 (0.0475, 0.1140)	0.98
Luvira et al, 2011	Thailand	333	129	732.639	0.1761 (0.1482, 0.2092)	1.12
Martin et al, 2011	Brazil	2032	90	1835.5	0.0490 (0.0399, 0.0603)	1.11
Muñoz de Bustillo et al, 2011	Spain	1515	339	3213.7	0.1055 (0.0948, 0.1173)	1.14
Quintanar Lartundo et al, 2011	Spain	411	47	346.97	0.1355 (0.1018, 0.1803)	1.07
Topanthanont et al, 2011	Thailand	103	12	436.5	0.0275 (0.0156, 0.0484)	0.89
Oliveira et al, 2012	Brazil	189	51	262.25	0.1945 (0.1478, 0.2559)	1.08
Pierce et al, 2012	United States	377	44	550	0.0800 (0.0595, 0.1075)	1.06
Rocha et al, 2012	Portugal	438	89	985.3	0.0903 (0.0734, 0.1112)	1.11
Su et al, 2012	China	158	6	290.833	0.0206 (0.0093, 0.0459)	0.72
Xu et al, 2012	China	313	50	1133.13	0.0441 (0.0334, 0.0582)	1.07
Kerschbaum et al, 2013	Austria	726	123	1703	0.0722 (0.0605, 0.0862)	1.12
Nessim et al, 2013	Canada	938	252	2110.9	0.1194 (0.1055, 0.1351)	1.13
Özbalci et al, 2013	Türkiye	153	23	616.197	0.0373 (0.0248, 0.0562)	1.00
Tsai et al, 2013	Taiwan	349	66	1009.42	0.0654 (0.0514, 0.0832)	1.09
Hsieh et al, 2014	Taiwan	391	49	969.5	0.0505 (0.0382, 0.0669)	1.07
Isla et al, 2014	South Africa	152	30	256.098	0.1171 (0.0819, 0.1675)	1.03
Najafi et al, 2014	Iran	2239	113	2343.75	0.0482 (0.0401, 0.0580)	1.12
Nieto-Ríos et al, 2014	Colombia	1497	451	3068	0.1470 (0.1340, 0.1612)	1.14
Nishina et al, 2014	Japan	192	26	752.667	0.0345 (0.0235, 0.0507)	1.01
Prasad et al, 2014	India	560	89	742.33	0.1199 (0.0974, 0.1476)	1.11
Courivaud et al, 2015	France	240	27	434.783	0.0621 (0.0426, 0.0906)	1.02
Ozisik et al, 2015	Türkiye	440	79	1001.08	0.0789 (0.0633, 0.0984)	1.10
Pi et al, 2015	China	346	23	467.04	0.0492 (0.0327, 0.0741)	1.00
Buttigieg et al, 2016	Malta	137	36	369.231	0.0975 (0.0703, 0.1352)	1.05
Duquennoy et al, 2016	Multinational	8396	1372	12150.9	0.1129 (0.1071, 0.1190)	1.15
El-Reshaid et al, 2016	Kuwait	308	21	522.417	0.0402 (0.0262, 0.0617)	0.98
Higuchi et al, 2016	Japan	3042	116	2640.5	0.0439 (0.0366, 0.0527)	1.12
Kotera et al, 2016	Japan	119	18	261.5	0.0688 (0.0434, 0.1093)	0.96
Mizuno et al, 2016	Japan	1237	93	2147.76	0.0433 (0.0353, 0.0531)	1.11
Nakao et al, 2016	Japan	527	66	2268.28	0.0291 (0.0229, 0.0370)	1.09
Pérez-Fontan et al, 2016	Spain	691	108	1658.1	0.0651 (0.0539, 0.0787)	1.11
Santhakumaran et al, 2016	United Kingdom	580	46	826.058	0.0557 (0.0417, 0.0743)	1.07
Shea et al, 2016	Hong Kong	114	10	113.667	0.0880 (0.0473, 0.1635)	0.85
Tian et al, 2016	China	294	53	2917.5	0.0182 (0.0139, 0.0238)	1.08
Lee et al, 2017	South Korea	295	28	1829.67	0.0153 (0.0106, 0.0222)	1.02
Ndlovu et al, 2017	South Africa	140	50	107.718	0.4642 (0.3518, 0.6124)	1.07
Ong et al, 2017	Malaysia	1603	108	1299	0.0831 (0.0689, 0.1004)	1.11
Vargas et al, 2017	Colombia	3525	44	752	0.0585 (0.0435, 0.0786)	1.06
Al Wakeel et al, 2018	Saudi Arabia	111	24	238.125	0.1008 (0.0676, 0.1504)	1.00
Fang et al, 2018	China	462	24	737	0.0326 (0.0218, 0.0486)	1.00
Hsu et al, 2018	Taiwan	398	114	1124.4	0.1014 (0.0844, 0.1218)	1.12
Ponce et al, 2018	Brazil	491	19	551.16	0.0345 (0.0220, 0.0540)	0.97
Alfayez et al, 2019	Saudi Arabia	100	16	212.75	0.0752 (0.0461, 0.1228)	0.94
Einbinder et al, 2019	Israel	201	83	256.48	0.3236 (0.2610, 0.4013)	1.10
Gadola et al, 2019	Uruguay	219	36	469.08	0.0767 (0.0554, 0.1064)	1.05
Karthikeyan et al, 2019	India	120	12	188.83	0.0635 (0.0361, 0.1119)	0.89
Liao et al, 2019	China	458	12	1185.2	0.0101 (0.0058, 0.0178)	0.89
Wang et al, 2019	Taiwan	336	40	760	0.0526 (0.0386, 0.0718)	1.06
Hwang et al, 2020	South Korea	643	64	3537	0.0181 (0.0142, 0.0231)	1.09
Perl et al, 2020	Australia/New Zealand	510	37	534.8	0.0692 (0.0501, 0.0955)	1.05
Perl et al, 2020	Canada	917	63	1376.8	0.0458 (0.0357, 0.0586)	1.09
Perl et al, 2020	United Kingdom	342	23	314.1	0.0732 (0.0487, 0.1102)	1.00
Perl et al, 2020	United States	3635	126	3754.6	0.0336 (0.0282, 0.0400)	1.12
Zeng et al, 2020	China	909	182	3927.52	0.0463 (0.0401, 0.0536)	1.13
AlZabli et al, 2021	Saudi Arabia	131	47	305	0.1541 (0.1158, 0.2051)	1.07
Htay et al, 2021	Singapore	288	21	257.9	0.0814 (0.0531, 0.1249)	0.98
Neu et al, 2021	United States	1055	115	1620.08	0.0710 (0.0591, 0.0852)	1.12
Oki et al, 2021	Japan	159	14	581.82	0.0241 (0.0143, 0.0406)	0.92
Sakurada et al, 2021	Japan	126	15	302	0.0497 (0.0299, 0.0824)	0.93
Santos et al, 2021	Spain	878	175	2270.7	0.0771 (0.0665, 0.0894)	1.13
Witoon et al, 2021	Thailand	532	222	1350	0.1644 (0.1442, 0.1876)	1.13
Kanjanabuch et al, 2022	Thailand	848	104	878.05	0.1184 (0.0977, 0.1435)	1.11
Kolland et al, 2022	Austria	250	41	662.7	0.0619 (0.0456, 0.0840)	1.06
Özbek et al, 2022	Türkiye	352	17	274.083	0.0620 (0.0386, 0.0998)	0.95
Szeto et al, 2022	Hong Kong	1193	902	7707.32	0.1170 (0.1096, 0.1249)	1.15
Wu et al, 2022	China	2570	377	9919.25	0.0380 (0.0344, 0.0420)	1.14
Zhai et al, 2022	China	283	31	408	0.0760 (0.0534, 0.1080)	1.03
Chienwichai et al, 2023	Thailand	336	33	288.1	0.1145 (0.0814, 0.1611)	1.04
Forte et al, 2023	France	8848	982	13023	0.0754 (0.0708, 0.0803)	1.15
Pak et al, 2023	Hong Kong	172	55	438.87	0.1253 (0.0962, 0.1632)	1.08
Srivastava et al, 2023	India	200	5	113.33	0.0441 (0.0184, 0.1060)	0.67
Ye et al, 2023	China	138	24	665.4	0.0361 (0.0242, 0.0538)	1.00
Aldriwesh et al, 2024	Saudi Arabia	100	35	166.07	0.2108 (0.1513, 0.2935)	1.04
Dandecha et al, 2024	Thailand	120	41	440	0.0932 (0.0686, 0.1266)	1.06
Do?an et al, 2024	Türkiye	120	3	120.45	0.0249 (0.0080, 0.0772)	0.53
Gül, 2024	Türkiye	171	18	527.4	0.0341 (0.0215, 0.0542)	0.96
Ito et al, 2024	Japan	1225	75	1996.3	0.0376 (0.0300, 0.0471)	1.10
Liang et al, 2024	China	166	66	1944.83	0.0339 (0.0267, 0.0432)	1.09
Nardelli et al, 2024	Italy	267	37	876	0.0422 (0.0306, 0.0583)	1.05
Park et al, 2024	South Korea	184	75	750	0.1000 (0.0797, 0.1254)	1.10
Tobijaszewska et al, 2024	Sweden	675	168	1273.68	0.1319 (0.1134, 0.1534)	1.13
Alfano et al, 2025	Italy		99	1488.9	0.0665 (0.0546, 0.0810)	1.11
Kim et al, 2025	South Korea	766	30	1044.12	0.0287 (0.0201, 0.0411)	1.03
Soetendorp et al, 2025	Israel	113	10	133.929	0.0747 (0.0402, 0.1388)	0.85
Tsuchiya et al, 2025	Japan	180	70	1243.78	0.0563 (0.0445, 0.0711)	1.09
Yu et al, 2025	China	506	20	681.75	0.0293 (0.0189, 0.0455)	0.98
Cho et al, 2026	South Korea	162	26	585	0.0444 (0.0303, 0.0653)	1.01
Okamoto et al, 2026	Japan	105	10	646.15	0.0155 (0.0083, 0.0288)	0.85
Cho Y, et al (Supplement to Cheikh Hassan HI, 2022)	Australia	6610	1759	10470	0.1680 (0.1603, 0.1760)	1.15
Wang J, et al (Supplement to Hu J, 2021)	China	354	27	600.117	0.0450 (0.0309, 0.0656)	1.02
Hu S, et al (Supplement to Hu S, 2019)	China	278	38	807.41	0.0471 (0.0342, 0.0647)	1.05
Jegatheesan D, et al (Supplement to Cheikh Hassan HI, 2022)	Australia	8343	2045	14688	0.1392 (0.1333, 0.1454)	1.15
Hayat A, et al (Supplement to Cheikh Hassan HI, 2022)	Australia	665	250	2084.91	0.1199 (0.1059, 0.1357)	1.13
Overall (I-squared = 97.5%, p = 0.000)					0.0651 (0.0582, 0.0728)	100.00
with estimated predictive interval					. (0.0225, 0.1888)	

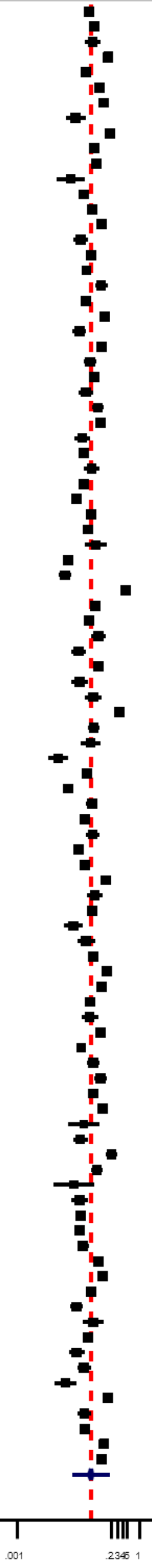

NOTE: Weights are from random effects analysis

Figure S3 Meta-Analysis of Incidence Rate Estimation of PD-Associated Peritonitis, by Causative Organisms (Continued)

C. Fungus

First_Author	Country	Sample_Size	Fungus	Total_PY	Episode per patient-year (95% CI)	% Weight
Brown et al, 2011	United Kingdom		64	3175.5	0.0202 (0.0158, 0.0257)	1.41
Davenport et al, 2011	United Kingdom	4095	49	5819.5	0.0084 (0.0064, 0.0111)	1.40
Huang et al, 2011	Taiwan	630	19	1688.4	0.0113 (0.0072, 0.0176)	1.33
Luvira et al, 2011	Thailand	333	15	732.639	0.0205 (0.0123, 0.0340)	1.30
Martin et al, 2011	Brazil	2032	9	1835.5	0.0049 (0.0026, 0.0094)	1.21
Muñoz de Bustillo et al, 2011	Spain	1515	59	3213.7	0.0184 (0.0142, 0.0237)	1.41
Quintanar Lartundo et al, 2011	Spain	411	5	346.97	0.0144 (0.0060, 0.0346)	1.07
Topanthanont et al, 2011	Thailand	103	3	436.5	0.0069 (0.0022, 0.0213)	0.91
Basturk et al, 2012	Türkiye	355	16	1070.17	0.0150 (0.0092, 0.0244)	1.31
Levallois et al, 2012	Canada	288	9	604.833	0.0149 (0.0077, 0.0286)	1.21
Oliveira et al, 2012	Brazil	189	4	262.25	0.0153 (0.0057, 0.0406)	1.00
Pierce et al, 2012	United States	377	13	550	0.0236 (0.0137, 0.0407)	1.28
Rocha et al, 2012	Portugal	438	28	985.3	0.0284 (0.0196, 0.0412)	1.37
Su et al, 2012	China	158	1	290.833	0.0034 (0.0005, 0.0244)	0.52
Xu et al, 2012	China	313	3	1133.13	0.0026 (0.0009, 0.0082)	0.91
Kerschbaum et al, 2013	Austria	726	20	1703	0.0117 (0.0076, 0.0182)	1.33
Nessim et al, 2013	Canada	938	37	2110.9	0.0175 (0.0127, 0.0242)	1.39
Özbalci et al, 2013	Türkiye	153	9	616.197	0.0146 (0.0076, 0.0281)	1.21
Tsai et al, 2013	Taiwan	349	5	1009.42	0.0050 (0.0021, 0.0119)	1.07
Hsieh et al, 2014	Taiwan	391	3	969.5	0.0031 (0.0010, 0.0096)	0.91
Isla et al, 2014	South Africa	152	3	256.098	0.0117 (0.0038, 0.0363)	0.91
Kumar et al, 2014	India	224	20	452.95	0.0442 (0.0285, 0.0684)	1.33
Najafi et al, 2014	Iran	2239	45	2343.75	0.0192 (0.0143, 0.0257)	1.40
Nieto-Ríos et al, 2014	Colombia	1497	95	3068	0.0310 (0.0253, 0.0379)	1.43
Nishina et al, 2014	Japan	192	4	752.667	0.0053 (0.0020, 0.0142)	1.00
Prasad et al, 2014	India	560	41	742.33	0.0552 (0.0407, 0.0750)	1.39
Ozisik et al, 2015	Türkiye	440	3	1001.08	0.0030 (0.0010, 0.0093)	0.91
Pi et al, 2015	China	346	3	467.04	0.0064 (0.0021, 0.0199)	0.91
Duquennoy et al, 2016	Multinational	8396	83	12150.9	0.0068 (0.0055, 0.0085)	1.42
El-Reshaid et al, 2016	Kuwait	308	17	522.417	0.0325 (0.0202, 0.0523)	1.31
Higuchi et al, 2016	Japan	3042	7	2640.5	0.0027 (0.0013, 0.0056)	1.16
Kotera et al, 2016	Japan	119	1	261.5	0.0038 (0.0005, 0.0271)	0.52
Mizuno et al, 2016	Japan	1237	17	2147.76	0.0079 (0.0049, 0.0127)	1.31
Nakao et al, 2016	Japan	527	8	2268.28	0.0035 (0.0018, 0.0071)	1.19
Pérez-Fontan et al, 2016	Spain	691	24	1658.1	0.0145 (0.0097, 0.0216)	1.35
Shea et al, 2016	Hong Kong	114	1	113.667	0.0088 (0.0012, 0.0625)	0.52
Tian et al, 2016	China	294	6	2917.5	0.0021 (0.0009, 0.0046)	1.12
Lee et al, 2017	South Korea	295	3	1829.67	0.0016 (0.0005, 0.0051)	0.91
Ndlovu et al, 2017	South Africa	140	7	107.718	0.0650 (0.0310, 0.1363)	1.16
Ong et al, 2017	Malaysia	1603	12	1299	0.0092 (0.0052, 0.0163)	1.26
Vargas et al, 2017	Colombia	3525	2	752	0.0027 (0.0007, 0.0106)	0.76
Fang et al, 2018	China	462	8	737	0.0109 (0.0054, 0.0217)	1.19
Hsu et al, 2018	Taiwan	398	6	1124.4	0.0053 (0.0024, 0.0119)	1.12
Ponce et al, 2018	Brazil	491	2	551.16	0.0036 (0.0009, 0.0145)	0.76
Einbinder et al, 2019	Israel	201	5	256.48	0.0195 (0.0081, 0.0468)	1.07
Hu et al, 2019	China	730	11	1638.75	0.0067 (0.0037, 0.0121)	1.25
Karthikeyan et al, 2019	India	120	5	188.83	0.0265 (0.0110, 0.0636)	1.07
Liao et al, 2019	China	458	6	1185.2	0.0051 (0.0023, 0.0113)	1.12
Wang et al, 2019	Taiwan	336	6	760	0.0079 (0.0035, 0.0176)	1.12
Hwang et al, 2020	South Korea	643	6	3537	0.0017 (0.0008, 0.0038)	1.12
Perl et al, 2020	Australia/New Zealand	510	6	534.8	0.0112 (0.0050, 0.0250)	1.12
Perl et al, 2020	Canada	917	8	1376.8	0.0058 (0.0029, 0.0116)	1.19
Perl et al, 2020	United Kingdom	342	4	314.1	0.0127 (0.0048, 0.0339)	1.00
Perl et al, 2020	United States	3635	9	3754.6	0.0024 (0.0012, 0.0046)	1.21
Zeng et al, 2020	China	909	20	3927.52	0.0051 (0.0033, 0.0079)	1.33
AlZabli et al, 2021	Saudi Arabia	131	11	305	0.0361 (0.0200, 0.0651)	1.25
Htay et al, 2021	Singapore	288	1	257.9	0.0039 (0.0005, 0.0275)	0.52
Li et al, 2021	China	739	15	2105	0.0071 (0.0043, 0.0118)	1.30
Neu et al, 2021	United States	1055	33	1620.08	0.0204 (0.0145, 0.0287)	1.38
Oki et al, 2021	Japan	159	2	581.82	0.0034 (0.0009, 0.0137)	0.76
Santos et al, 2021	Spain	878	32	2270.7	0.0141 (0.0100, 0.0199)	1.38
Witoon et al, 2021	Thailand	532	13	1350	0.0096 (0.0056, 0.0166)	1.28
Kanjanabuch et al, 2022	Thailand	848	14	878.05	0.0159 (0.0094, 0.0269)	1.29
Kolland et al, 2022	Austria	250	3	662.7	0.0045 (0.0015, 0.0140)	0.91
Özbek et al, 2022	Türkiye	352	1	274.083	0.0036 (0.0005, 0.0259)	0.52
Szeto et al, 2022	Hong Kong	1193	52	7707.32	0.0067 (0.0051, 0.0089)	1.40
Zhai et al, 2022	China	283	6	408	0.0147 (0.0066, 0.0327)	1.12
Chienwichai et al, 2023	Thailand	336	3	288.1	0.0104 (0.0034, 0.0323)	0.91
Forte et al, 2023	France	8848	44	13023	0.0034 (0.0025, 0.0045)	1.40
Pak et al, 2023	Hong Kong	172	5	438.87	0.0114 (0.0047, 0.0274)	1.07
Srivastava et al, 2023	India	200	22	113.33	0.1941 (0.1278, 0.2948)	1.34
Aldriwesh et al, 2024	Saudi Arabia	100	1	166.07	0.0060 (0.0008, 0.0427)	0.52
Dandecha et al, 2024	Thailand	120	6	440	0.0136 (0.0061, 0.0304)	1.12
Do?an et al, 2024	Türkiye	120	1	120.45	0.0083 (0.0012, 0.0589)	0.52
Ito et al, 2024	Japan	1225	2	1996.3	0.0010 (0.0003, 0.0040)	0.76
Liang et al, 2024	China	166	4	1944.83	0.0021 (0.0008, 0.0055)	1.00
Nardelli et al, 2024	Italy	267	2	876	0.0023 (0.0006, 0.0091)	0.76
Park et al, 2024	South Korea	184	6	750	0.0080 (0.0036, 0.0178)	1.12
Tobijaszewska et al, 2024	Sweden	675	19	1273.68	0.0149 (0.0095, 0.0234)	1.33
Alfano et al, 2025	Italy		3	1488.9	0.0020 (0.0006, 0.0062)	0.91
Kim et al, 2025	South Korea	766	3	1044.12	0.0029 (0.0009, 0.0089)	0.91
Soetendorp et al, 2025	Israel	113	2	133.929	0.0149 (0.0037, 0.0597)	0.76
Tsuchiya et al, 2025	Japan	180	3	1243.78	0.0024 (0.0008, 0.0075)	0.91
Al Laham et al, 2026	Oman	311	10	2333.33	0.0043 (0.0023, 0.0080)	1.23
Cho et al, 2026	South Korea	162	3	585	0.0051 (0.0017, 0.0159)	0.91
Okamoto et al, 2026	Japan	105	1	646.15	0.0015 (0.0002, 0.0110)	0.52
Cho Y, et al (Supplement to Cheikh Hassan HI, 2022)	Australia	6610	251	10470	0.0240 (0.0212, 0.0271)	1.44
Wang J, et al (Supplement to Hu J, 2021)	China	354	5	600.117	0.0083 (0.0035, 0.0200)	1.07
Hu S, et al (Supplement to Hu S, 2019)	China	278	6	807.41	0.0074 (0.0033, 0.0165)	1.12
Jegatheesan D, et al (Supplement to Cheikh Hassan HI, 2022)	Australia	8343	291	14688	0.0198 (0.0177, 0.0222)	1.44
Hayat A, et al (Supplement to Cheikh Hassan HI, 2022)	Australia	665	44	2084.91	0.0211 (0.0157, 0.0284)	1.40
Overall (I-squared = 91.2%, p = 0.000)					0.0094 (0.0079, 0.0112)	100.00
with estimated predictive interval					. (0.0021, 0.0416)	

NOTE: Weights are from random effects analysis

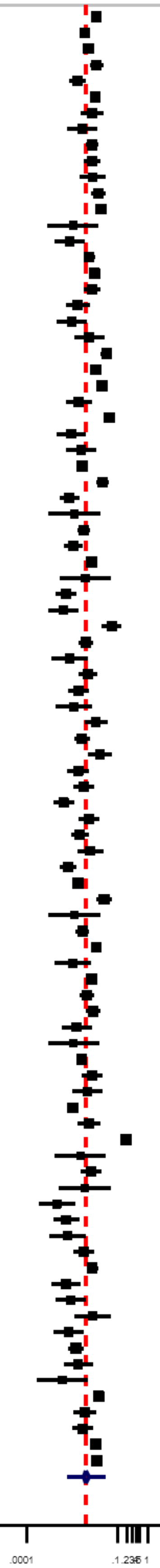

Figure S3 Meta-Analysis of Incidence Rate Estimation of PD-Associated Peritonitis, by Causative Organisms (Continued)

D. Mycobacterium

First_Author	Country	Sample_Size	Mycobacterium	Total_PY	Episode per patient-year (95% CI)	% Weight
Huang et al, 2011	Taiwan	630	5	1688.4	0.0030 (0.0012, 0.0071)	4.49
Muñoz de Bustillo et al, 2011	Spain	1515	7	3213.7	0.0022 (0.0010, 0.0046)	4.82
Rocha et al, 2012	Portugal	438	5	985.3	0.0051 (0.0021, 0.0122)	4.49
Özbalci et al, 2013	Türkiye	153	2	616.197	0.0032 (0.0008, 0.0130)	3.30
Tsai et al, 2013	Taiwan	349	2	1009.42	0.0020 (0.0005, 0.0079)	3.30
Isla et al, 2014	South Africa	152	6	256.098	0.0234 (0.0105, 0.0521)	4.67
Najafi et al, 2014	Iran	2239	5	2343.75	0.0021 (0.0009, 0.0051)	4.49
Prasad et al, 2014	India	560	11	742.33	0.0148 (0.0082, 0.0268)	5.16
Mizuno et al, 2016	Japan	1237	3	2147.76	0.0014 (0.0005, 0.0043)	3.87
Nakao et al, 2016	Japan	527	1	2268.28	0.0004 (0.0001, 0.0031)	2.29
Pérez-Fontan et al, 2016	Spain	691	1	1658.1	0.0006 (0.0001, 0.0043)	2.29
Shea et al, 2016	Hong Kong	114	1	113.667	0.0088 (0.0012, 0.0625)	2.29
Ndlovu et al, 2017	South Africa	140	1	107.718	0.0093 (0.0013, 0.0659)	2.29
Ong et al, 2017	Malaysia	1603	1	1299	0.0008 (0.0001, 0.0055)	2.29
Al Wakeel et al, 2018	Saudi Arabia	111	1	238.125	0.0042 (0.0006, 0.0298)	2.29
Alfayez et al, 2019	Saudi Arabia	100	1	212.75	0.0047 (0.0007, 0.0334)	2.29
Karthikeyan et al, 2019	India	120	2	188.83	0.0106 (0.0026, 0.0423)	3.30
Liao et al, 2019	China	458	1	1185.2	0.0008 (0.0001, 0.0060)	2.29
Wang et al, 2019	Taiwan	336	3	760	0.0039 (0.0013, 0.0122)	3.87
Htay et al, 2021	Singapore	288	1	257.9	0.0039 (0.0005, 0.0275)	2.29
Oki et al, 2021	Japan	159	1	581.82	0.0017 (0.0002, 0.0122)	2.29
Kanjanabuch et al, 2022	Thailand	848	4	878.05	0.0046 (0.0017, 0.0121)	4.23
Szeto et al, 2022	Hong Kong	1193	61	7707.32	0.0079 (0.0062, 0.0102)	5.76
Pak et al, 2023	Hong Kong	172	7	438.87	0.0160 (0.0076, 0.0335)	4.82
Srivastava et al, 2023	India	200	2	113.33	0.0176 (0.0044, 0.0706)	3.30
Yang et al, 2023	Taiwan	2084	18	8871.11	0.0020 (0.0013, 0.0032)	5.43
Alfano et al, 2025	Italy		1	1488.9	0.0007 (0.0001, 0.0048)	2.29
Cho et al, 2026	South Korea	162	2	585	0.0034 (0.0009, 0.0137)	3.30
Okamoto et al, 2026	Japan	105	1	646.15	0.0015 (0.0002, 0.0110)	2.29
Overall (I-squared = 74.4%, p = 0.000)					0.0039 (0.0026, 0.0056)	100.00
with estimated predictive interval					. (0.0007, 0.0206)	

NOTE: Weights are from random effects analysis

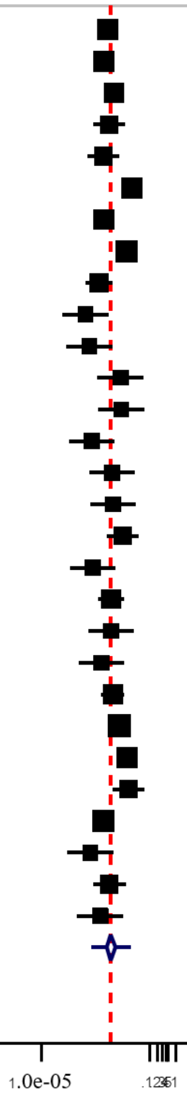

Figure S3 Meta-Analysis of Incidence Rate Estimation of PD-Associated Peritonitis, by Causative Organisms (Continued)

E. Polymicrobial

First_Author	Country	Sample_Size	Polymicrobial	Total_PY	Episode per patient-year (95% CI)	% Weight
Huang et al, 2011	Taiwan	630	44	1688.4	0.0261 (0.0194, 0.0350)	2.33
Muñoz de Bustillo et al, 2011	Spain	1515	63	3213.7	0.0196 (0.0153, 0.0251)	2.37
Topanthanont et al, 2011	Thailand	103	8	436.5	0.0183 (0.0092, 0.0366)	1.91
Oliveira et al, 2012	Brazil	189	4	262.25	0.0153 (0.0057, 0.0406)	1.56
Rocha et al, 2012	Portugal	438	24	985.3	0.0244 (0.0163, 0.0363)	2.24
Su et al, 2012	China	158	1	290.833	0.0034 (0.0005, 0.0244)	0.74
Tsai et al, 2013	Taiwan	349	11	1009.42	0.0109 (0.0060, 0.0197)	2.03
Hsieh et al, 2014	Taiwan	391	21	969.5	0.0217 (0.0141, 0.0332)	2.21
Nishina et al, 2014	Japan	192	17	752.667	0.0226 (0.0140, 0.0363)	2.16
Prasad et al, 2014	India	560	5	742.33	0.0067 (0.0028, 0.0162)	1.68
Pi et al, 2015	China	346	4	467.04	0.0086 (0.0032, 0.0228)	1.56
Buttigieg et al, 2016	Malta	137	18	369.231	0.0487 (0.0307, 0.0774)	2.18
Duquennoy et al, 2016	Multinational	8396	445	12150.9	0.0366 (0.0334, 0.0402)	2.44
El-Reshaid et al, 2016	Kuwait	308	14	522.417	0.0268 (0.0159, 0.0452)	2.11
Higuchi et al, 2016	Japan	3042	32	2640.5	0.0121 (0.0086, 0.0171)	2.29
Kotera et al, 2016	Japan	119	7	261.5	0.0268 (0.0128, 0.0562)	1.85
Mizuno et al, 2016	Japan	1237	12	2147.76	0.0056 (0.0032, 0.0098)	2.06
Nakao et al, 2016	Japan	527	10	2268.28	0.0044 (0.0024, 0.0082)	2.00
Pérez-Fontan et al, 2016	Spain	691	84	1658.1	0.0507 (0.0409, 0.0627)	2.39
Shea et al, 2016	Hong Kong	114	1	113.667	0.0088 (0.0012, 0.0625)	0.74
Tian et al, 2016	China	294	2	2917.5	0.0007 (0.0002, 0.0027)	1.14
Ndlovu et al, 2017	South Africa	140	1	107.718	0.0093 (0.0013, 0.0659)	0.74
Ong et al, 2017	Malaysia	1603	11	1299	0.0085 (0.0047, 0.0153)	2.03
Al Wakeel et al, 2018	Saudi Arabia	111	3	238.125	0.0126 (0.0041, 0.0391)	1.39
Alfayez et al, 2019	Saudi Arabia	100	5	212.75	0.0235 (0.0098, 0.0565)	1.68
Liao et al, 2019	China	458	2	1185.2	0.0017 (0.0004, 0.0067)	1.14
Wang et al, 2019	Taiwan	336	11	760	0.0145 (0.0080, 0.0261)	2.03
Hwang et al, 2020	South Korea	643	6	3537	0.0017 (0.0008, 0.0038)	1.78
Perl et al, 2020	Australia/New Zealand	510	19	534.8	0.0355 (0.0227, 0.0557)	2.19
Perl et al, 2020	Canada	917	35	1376.8	0.0254 (0.0183, 0.0354)	2.30
Perl et al, 2020	United Kingdom	342	10	314.1	0.0318 (0.0171, 0.0592)	2.00
Perl et al, 2020	United States	3635	48	3754.6	0.0128 (0.0096, 0.0170)	2.34
AlZabli et al, 2021	Saudi Arabia	131	11	305	0.0361 (0.0200, 0.0651)	2.03
Htay et al, 2021	Singapore	288	6	257.9	0.0233 (0.0105, 0.0518)	1.78
Neu et al, 2021	United States	1055	70	1620.08	0.0432 (0.0342, 0.0546)	2.38
Santos et al, 2021	Spain	878	133	2270.7	0.0586 (0.0494, 0.0694)	2.41
Witoon et al, 2021	Thailand	532	5	1350	0.0037 (0.0015, 0.0089)	1.68
Kanjanabuch et al, 2022	Thailand	848	29	878.05	0.0330 (0.0230, 0.0475)	2.27
Szeto et al, 2022	Hong Kong	1193	462	7707.32	0.0599 (0.0547, 0.0657)	2.44
Forte et al, 2023	France	8848	251	13023	0.0193 (0.0170, 0.0218)	2.43
Pak et al, 2023	Hong Kong	172	20	438.87	0.0456 (0.0294, 0.0706)	2.20
Aldriwesh et al, 2024	Saudi Arabia	100	3	166.07	0.0181 (0.0058, 0.0560)	1.39
Ito et al, 2024	Japan	1225	27	1996.3	0.0135 (0.0093, 0.0197)	2.26
Liang et al, 2024	China	166	12	1944.83	0.0062 (0.0035, 0.0109)	2.06
Nardelli et al, 2024	Italy	267	4	876	0.0046 (0.0017, 0.0122)	1.56
Alfano et al, 2025	Italy		20	1488.9	0.0134 (0.0087, 0.0208)	2.20
Soetendorp et al, 2025	Israel	113	5	133.929	0.0373 (0.0155, 0.0897)	1.68
Yu et al, 2025	China	506	5	681.75	0.0073 (0.0031, 0.0176)	1.68
Cho et al, 2026	South Korea	162	12	585	0.0205 (0.0116, 0.0361)	2.06
Okamoto et al, 2026	Japan	105	10	646.15	0.0155 (0.0083, 0.0288)	2.00
Cho Y, et al (Supplement to Cheikh Hassan HI, 2022)	Australia	6610	691	10470	0.0660 (0.0613, 0.0711)	2.45
Hu S, et al (Supplement to Hu S, 2019)	China	278	3	807.41	0.0037 (0.0012, 0.0115)	1.39
Overall (I-squared = 95.3%, p = 0.000)					0.0169 (0.0138, 0.0207)	100.00
with estimated predictive interval					. (0.0044, 0.0648)	

NOTE: Weights are from random effects analysis

.0001 .1.2.3.5 1

Figure S3 Meta-Analysis of Incidence Rate Estimation of PD-Associated Peritonitis, by Causative Organisms (Continued)

F. Culture Negative

First_Author	Country	Sample_Size	Culture_Negative	Total_PY	Episode per patient-year (95% CI)	% Weight
Brown et al, 2011	United Kingdom		381	3175.5	0.1200 (0.1085, 0.1327)	1.13
Huang et al, 2011	Taiwan	630	142	1688.4	0.0841 (0.0713, 0.0991)	1.12
Liawnoraset, 2011	Thailand	318	77	271.85	0.2832 (0.2265, 0.3541)	1.10
Martin et al, 2011	Brazil	2032	181	1835.5	0.0986 (0.0852, 0.1141)	1.12
Muñoz de Bustillo et al, 2011	Spain	1515	239	3213.7	0.0744 (0.0655, 0.0844)	1.12
Quintanar Lartundo et al, 2011	Spain	411	27	346.97	0.0778 (0.0534, 0.1135)	1.03
Topanthanont et al, 2011	Thailand	103	19	436.5	0.0435 (0.0278, 0.0682)	1.00
Oliveira et al, 2012	Brazil	189	29	262.25	0.1106 (0.0768, 0.1591)	1.04
Pierce et al, 2012	United States	377	58	550	0.1055 (0.0815, 0.1364)	1.09
Rocha et al, 2012	Portugal	438	156	985.3	0.1583 (0.1353, 0.1852)	1.12
Su et al, 2012	China	158	21	290.833	0.0722 (0.0471, 0.1107)	1.01
Xu et al, 2012	China	313	64	1133.13	0.0565 (0.0442, 0.0722)	1.09
Kerschbaum et al, 2013	Austria	726	97	1703	0.0570 (0.0467, 0.0695)	1.11
Nessim et al, 2013	Canada	938	341	2110.9	0.1615 (0.1453, 0.1796)	1.13
Özbalci et al, 2013	Türkiye	153	62	616.197	0.1006 (0.0784, 0.1291)	1.09
Tsai et al, 2013	Taiwan	349	57	1009.42	0.0565 (0.0436, 0.0732)	1.09
Hsieh et al, 2014	Taiwan	391	39	969.5	0.0402 (0.0294, 0.0551)	1.06
Isla et al, 2014	South Africa	152	131	256.098	0.5115 (0.4310, 0.6071)	1.11
Najafi et al, 2014	Iran	2239	614	2343.75	0.2620 (0.2421, 0.2835)	1.13
Nieto-Ríos et al, 2014	Colombia	1497	637	3068	0.2076 (0.1921, 0.2244)	1.13
Nishina et al, 2014	Japan	192	23	752.667	0.0306 (0.0203, 0.0460)	1.02
Prasad et al, 2014	India	560	55	742.33	0.0741 (0.0569, 0.0965)	1.08
Courivaud et al, 2015	France	240	10	434.783	0.0230 (0.0124, 0.0427)	0.90
Ozisik et al, 2015	Türkiye	440	305	1001.08	0.3047 (0.2723, 0.3409)	1.13
Pi et al, 2015	China	346	29	467.04	0.0621 (0.0431, 0.0894)	1.04
Duquennoy et al, 2016	Multinational	8396	1087	12150.9	0.0895 (0.0843, 0.0949)	1.13
El-Reshaid et al, 2016	Kuwait	308	15	522.417	0.0287 (0.0173, 0.0476)	0.96
Higuchi et al, 2016	Japan	3042	131	2640.5	0.0496 (0.0418, 0.0589)	1.11
Kotera et al, 2016	Japan	119	14	261.5	0.0535 (0.0317, 0.0904)	0.95
Mizuno et al, 2016	Japan	1237	167	2147.76	0.0778 (0.0668, 0.0905)	1.12
Nakao et al, 2016	Japan	527	72	2268.28	0.0317 (0.0252, 0.0400)	1.10
Pérez-Fontan et al, 2016	Spain	691	79	1658.1	0.0476 (0.0382, 0.0594)	1.10
Santhakumaran et al, 2016	United Kingdom	580	66	826.058	0.0799 (0.0628, 0.1017)	1.09
Shea et al, 2016	Hong Kong	114	20	113.667	0.1760 (0.1135, 0.2727)	1.00
Tian et al, 2016	China	294	118	2917.5	0.0404 (0.0338, 0.0484)	1.11
Lee et al, 2017	South Korea	295	74	1829.67	0.0404 (0.0322, 0.0508)	1.10
Ndlovu et al, 2017	South Africa	140	37	107.718	0.3435 (0.2489, 0.4741)	1.06
Ong et al, 2017	Malaysia	1603	116	1299	0.0893 (0.0744, 0.1071)	1.11
Vargas et al, 2017	Colombia	3525	56	752	0.0745 (0.0573, 0.0968)	1.08
Al Wakeel et al, 2018	Saudi Arabia	111	17	238.125	0.0714 (0.0444, 0.1148)	0.98
Fang et al, 2018	China	462	39	737	0.0529 (0.0387, 0.0724)	1.06
Hsu et al, 2018	Taiwan	398	95	1124.4	0.0845 (0.0691, 0.1033)	1.11
Ponce et al, 2018	Brazil	491	74	551.16	0.1343 (0.1069, 0.1686)	1.10
Alfayez et al, 2019	Saudi Arabia	100	22	212.75	0.1034 (0.0681, 0.1570)	1.01
Einbinder et al, 2019	Israel	201	31	256.48	0.1209 (0.0850, 0.1719)	1.05
Jahromi et al, 2019	Iran	235	37	356.42	0.1038 (0.0752, 0.1433)	1.06
Karthikeyan et al, 2019	India	120	32	188.83	0.1695 (0.1198, 0.2396)	1.05
Liao et al, 2019	China	458	28	1185.2	0.0236 (0.0163, 0.0342)	1.04
Wang et al, 2019	Taiwan	336	50	760	0.0658 (0.0499, 0.0868)	1.08
Hwang et al, 2020	South Korea	643	108	3537	0.0305 (0.0253, 0.0369)	1.11
Noppakun et al, 2020	Thailand	1169	382	3418.4	0.1117 (0.1011, 0.1235)	1.13
Perl et al, 2020	Australia/New Zealand	510	26	534.8	0.0486 (0.0331, 0.0714)	1.03
Perl et al, 2020	Canada	917	62	1376.8	0.0450 (0.0351, 0.0578)	1.09
Perl et al, 2020	United Kingdom	342	17	314.1	0.0541 (0.0336, 0.0871)	0.98
Perl et al, 2020	United States	3635	155	3754.6	0.0413 (0.0353, 0.0483)	1.12
Zeng et al, 2020	China	909	92	3927.52	0.0234 (0.0191, 0.0287)	1.10
AlZabli et al, 2021	Saudi Arabia	131	43	305	0.1410 (0.1046, 0.1901)	1.07
Htay et al, 2021	Singapore	288	7	257.9	0.0271 (0.0129, 0.0569)	0.82
Li et al, 2021	China	739	98	2105	0.0466 (0.0382, 0.0567)	1.11
Neu et al, 2021	United States	1055	150	1620.08	0.0926 (0.0789, 0.1087)	1.12
Oki et al, 2021	Japan	159	42	581.82	0.0722 (0.0533, 0.0977)	1.07
Sakurada et al, 2021	Japan	126	13	302	0.0430 (0.0250, 0.0741)	0.94
Santos et al, 2021	Spain	878	126	2270.7	0.0555 (0.0466, 0.0661)	1.11
Witoon et al, 2021	Thailand	532	181	1350	0.1341 (0.1159, 0.1551)	1.12
Kanjanabuch et al, 2022	Thailand	848	98	878.05	0.1116 (0.0916, 0.1360)	1.11
Kolland et al, 2022	Austria	250	41	662.7	0.0619 (0.0456, 0.0840)	1.07
Özbek et al, 2022	Türkiye	352	15	274.083	0.0547 (0.0330, 0.0908)	0.96
Szeto et al, 2022	Hong Kong	1193	400	7707.32	0.0519 (0.0471, 0.0572)	1.13
Zhai et al, 2022	China	283	41	408	0.1005 (0.0740, 0.1365)	1.07
Chienwichai et al, 2023	Thailand	336	75	288.1	0.2603 (0.2076, 0.3264)	1.10
Pak et al, 2023	Hong Kong	172	33	438.87	0.0752 (0.0535, 0.1058)	1.05
Srivastava et al, 2023	India	200	55	113.33	0.4853 (0.3726, 0.6321)	1.08
Ye et al, 2023	China	138	36	665.4	0.0541 (0.0390, 0.0750)	1.06
Aldriwesh et al, 2024	Saudi Arabia	100	17	166.07	0.1024 (0.0636, 0.1647)	0.98
Dandecha et al, 2024	Thailand	120	31	440	0.0705 (0.0495, 0.1002)	1.05
Do?an et al, 2024	Türkiye	120	15	120.45	0.1245 (0.0751, 0.2066)	0.96
Gül, 2024	Türkiye	171	66	527.4	0.1251 (0.0983, 0.1593)	1.09
Ito et al, 2024	Japan	1225	108	1996.3	0.0541 (0.0448, 0.0653)	1.11
Liang et al, 2024	China	166	62	1944.83	0.0319 (0.0249, 0.0409)	1.09
Nardelli et al, 2024	Italy	267	52	876	0.0594 (0.0452, 0.0779)	1.08
Park et al, 2024	South Korea	184	21	750	0.0280 (0.0183, 0.0429)	1.01
Tobijaszewska et al, 2024	Sweden	675	26	1273.68	0.0204 (0.0139, 0.0300)	1.03
Alfano et al, 2025	Italy		66	1488.9	0.0443 (0.0348, 0.0564)	1.09
Kim et al, 2025	South Korea	766	33	1044.12	0.0316 (0.0225, 0.0445)	1.05
Soetendorp et al, 2025	Israel	113	4	133.929	0.0299 (0.0112, 0.0796)	0.68
Tsuchiya et al, 2025	Japan	180	50	1243.78	0.0402 (0.0305, 0.0530)	1.08
Yu et al, 2025	China	506	17	681.75	0.0249 (0.0155, 0.0401)	0.98
Cho et al, 2026	South Korea	162	38	585	0.0650 (0.0473, 0.0893)	1.06
Okamoto et al, 2026	Japan	105	19	646.15	0.0294 (0.0188, 0.0461)	1.00
Cho Y, et al (Supplement to Cheikh Hassan HI, 2022)	Australia	6610	817	10470	0.0780 (0.0729, 0.0836)	1.13
Wang J, et al (Supplement to Hu J, 2021)	China	354	50	600.117	0.0833 (0.0631, 0.1099)	1.08
Hu S, et al (Supplement to Hu S, 2019)	China	278	56	807.41	0.0694 (0.0534, 0.0901)	1.08
Jegatheesan D, et al (Supplement to Cheikh Hassan HI, 2022)	Australia	8343	1287	14688	0.0876 (0.0830, 0.0925)	1.13
Hayat A, et al (Supplement to Cheikh Hassan HI, 2022)	Australia	665	187	2084.91	0.0897 (0.0777, 0.1035)	1.12
Overall (I-squared = 97.8%, p = 0.000)					0.0735 (0.0647, 0.0835)	100.00
with estimated predictive interval					. (0.0217, 0.2487)	

NOTE: Weights are from random effects analysis

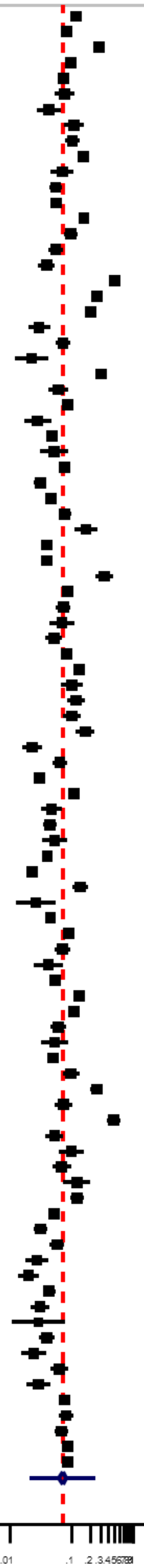

Figure S4 Funnel Plot and Doi Plot of Incidence Rate Estimation of PD-Associated Peritonitis

A. Overall

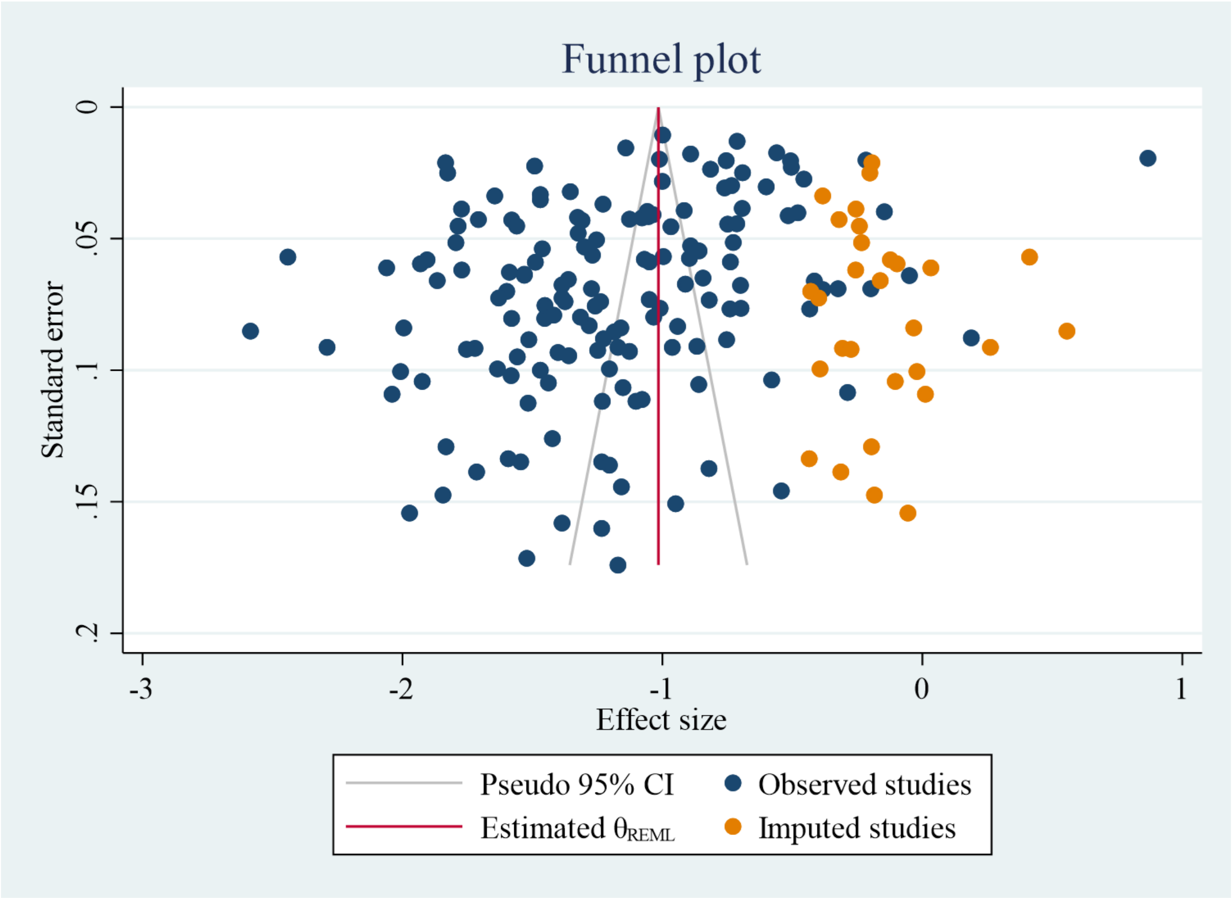

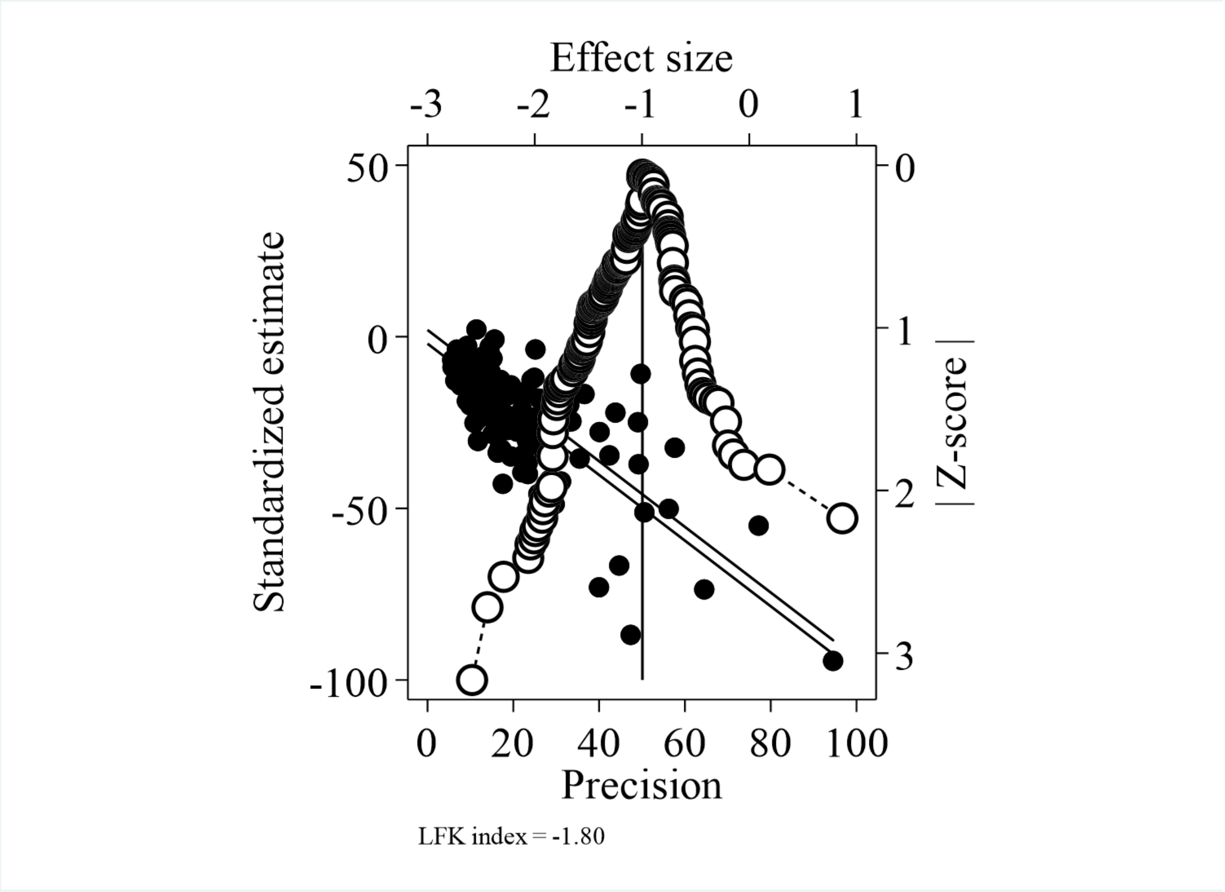

Figure S4 Funnel Plot and Doi Plot of Incidence Rate Estimation of PD-Associated Peritonitis (Continued)

B. Region of the Americas

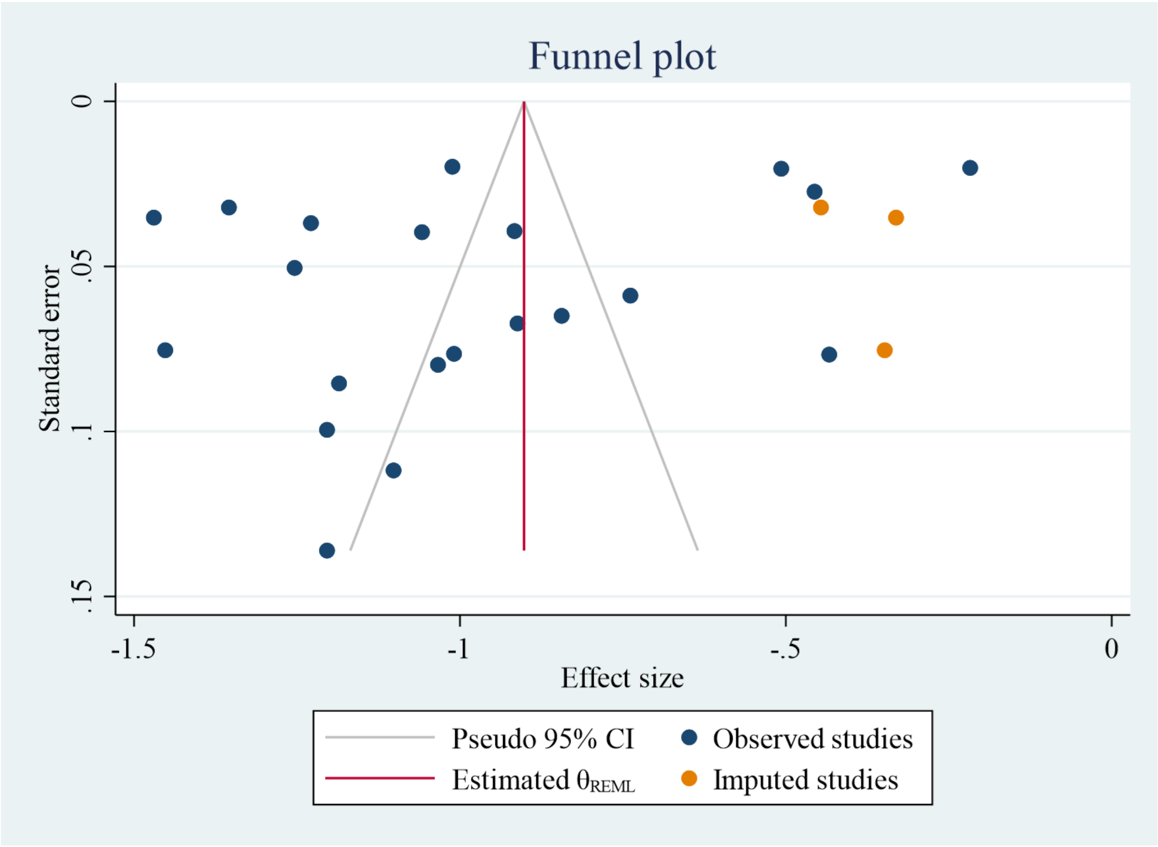

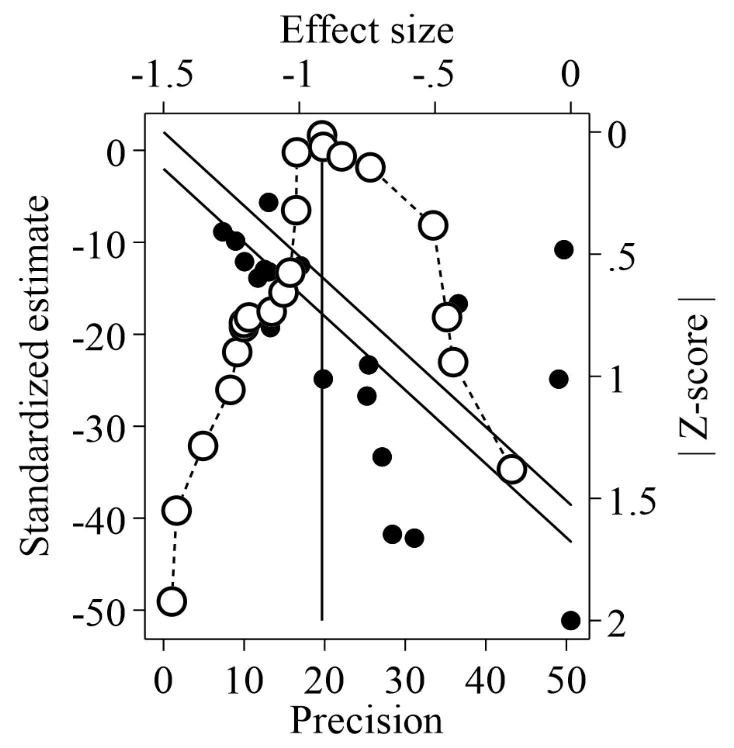

LFK index = -1.33

Figure S4 Funnel Plot and Doi Plot of Incidence Rate Estimation of PD-Associated Peritonitis (Continued)

C. South-East Asia Region

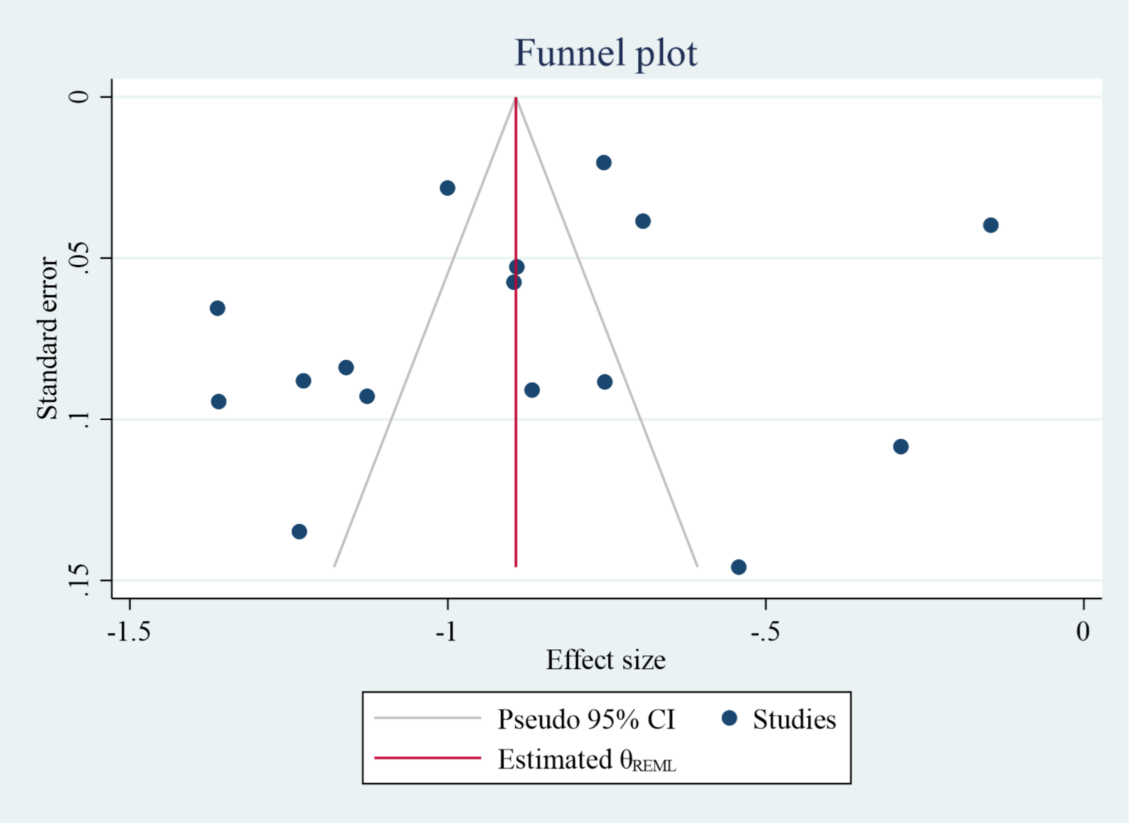

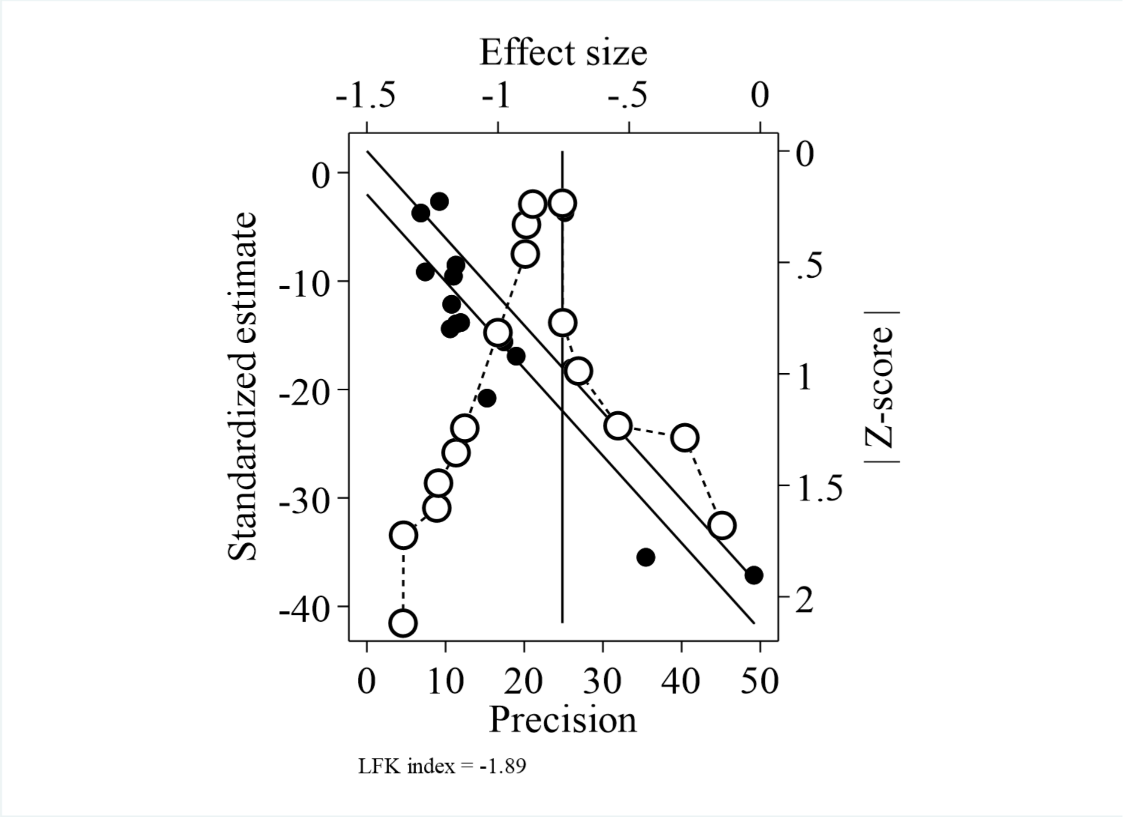

Figure S4 Funnel Plot and Doi Plot of Incidence Rate Estimation of PD-Associated Peritonitis (Continued)

D. European Region

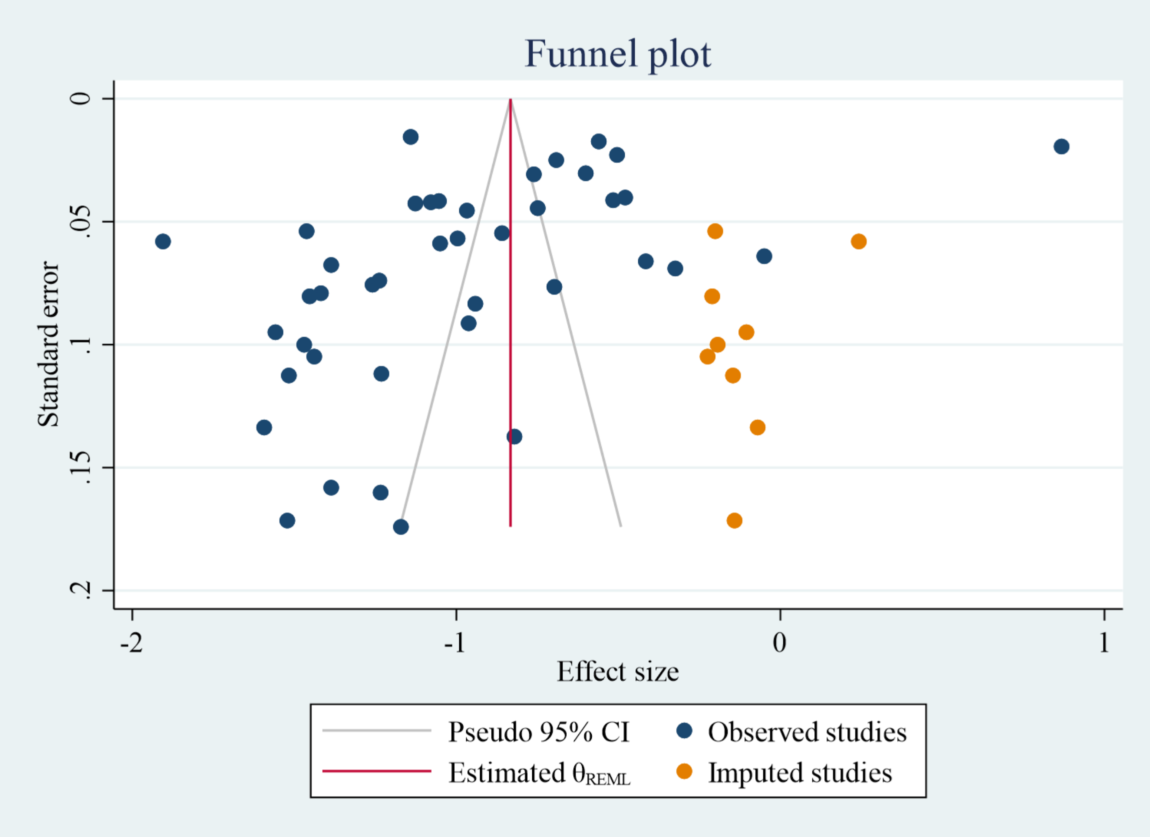

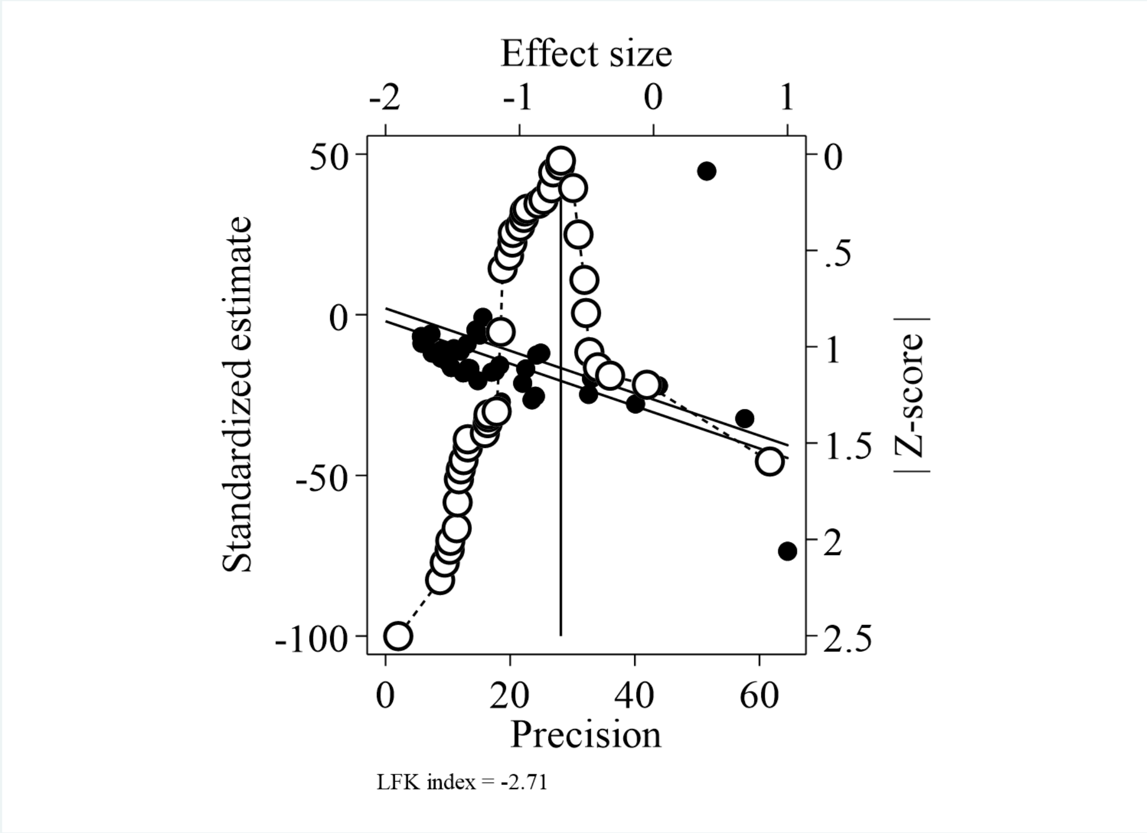

Figure S4 Funnel Plot and Doi Plot of Incidence Rate Estimation of PD-Associated Peritonitis (Continued)

E. Eastern Mediterranean Region

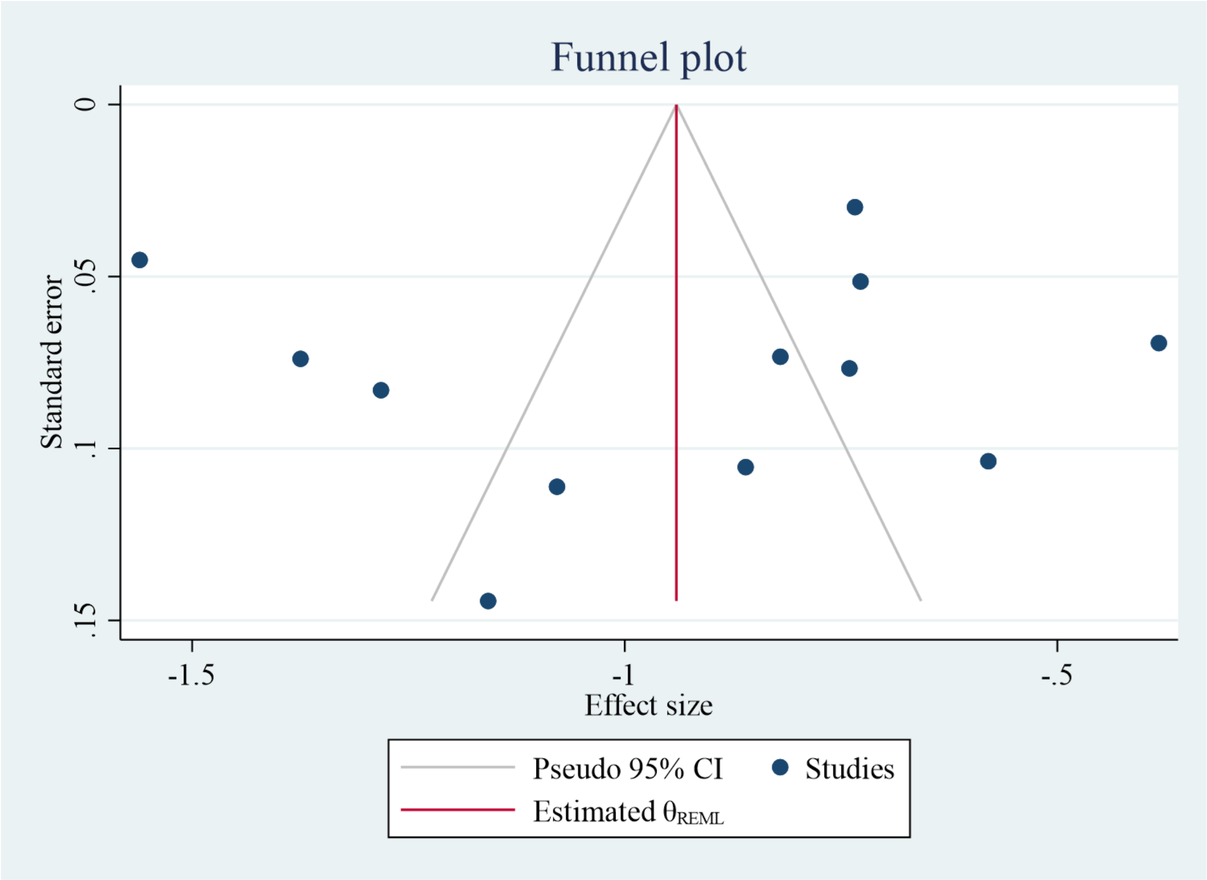

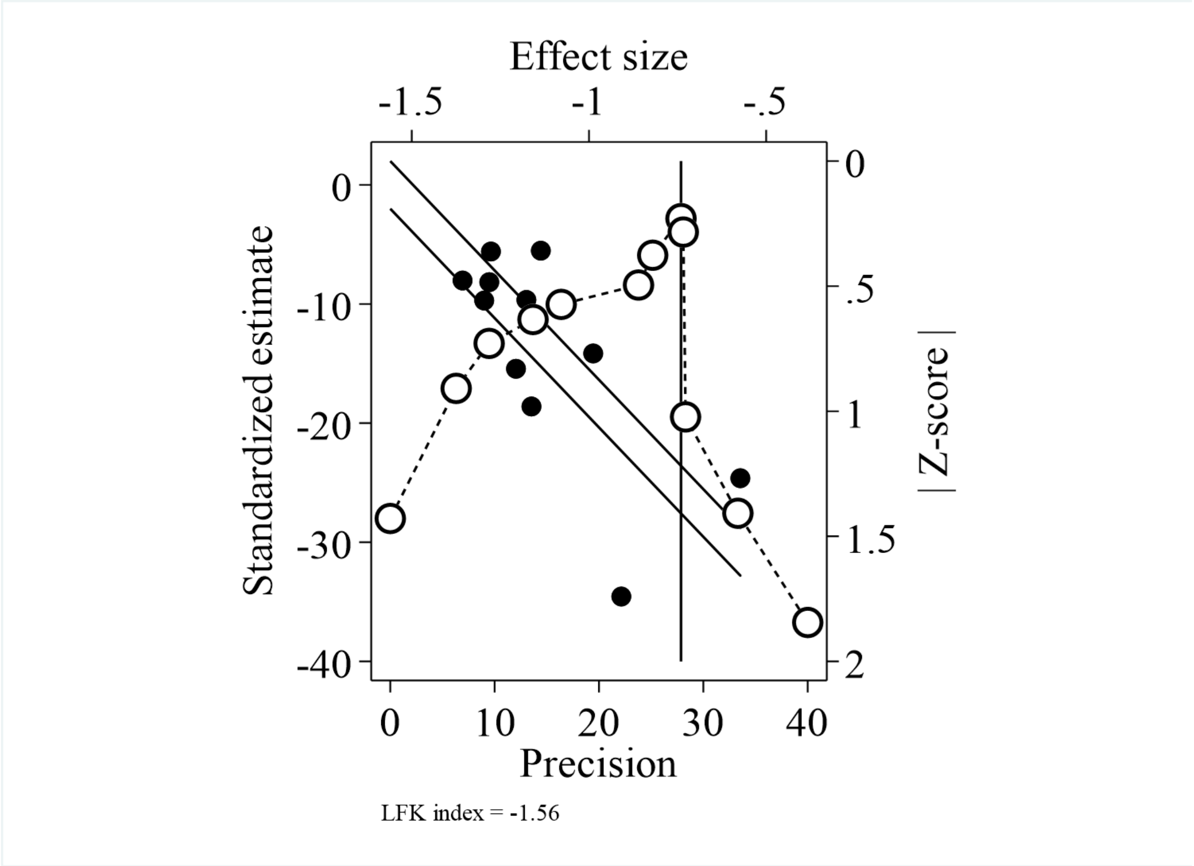

Figure S4 Funnel Plot and Doi Plot of Incidence Rate Estimation of PD-Associated Peritonitis (Continued)

F. Western Pacific Region

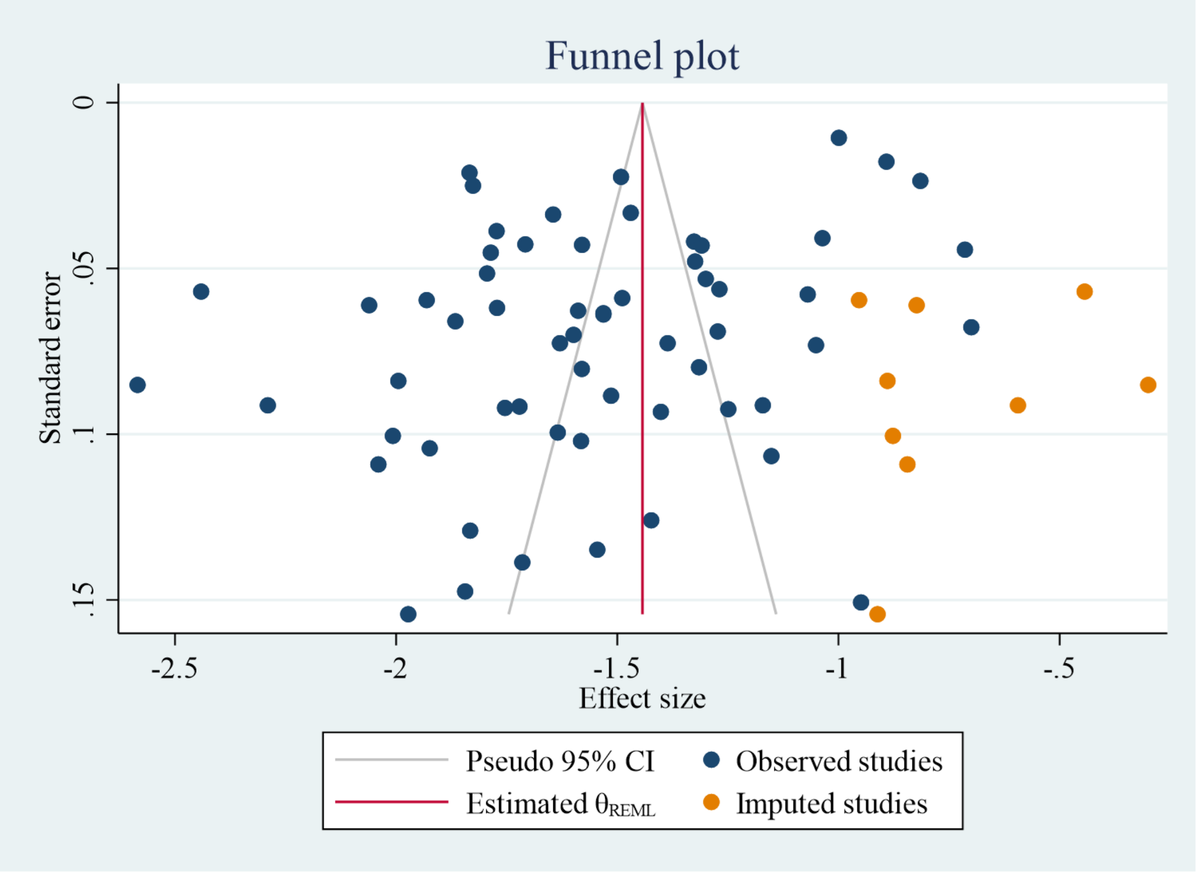

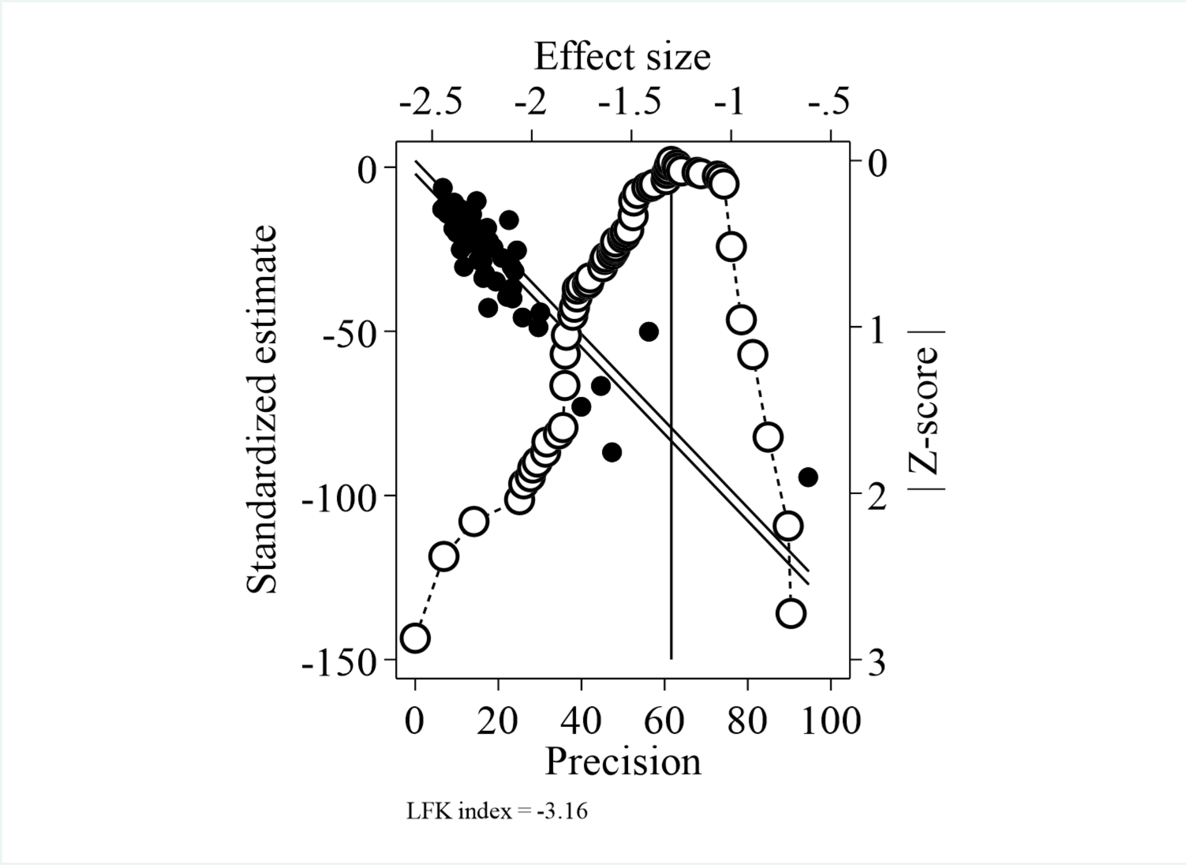

Figure S4 Funnel Plot and Doi Plot of Incidence Rate Estimation of PD-Associated Peritonitis (Continued)

G. Adult

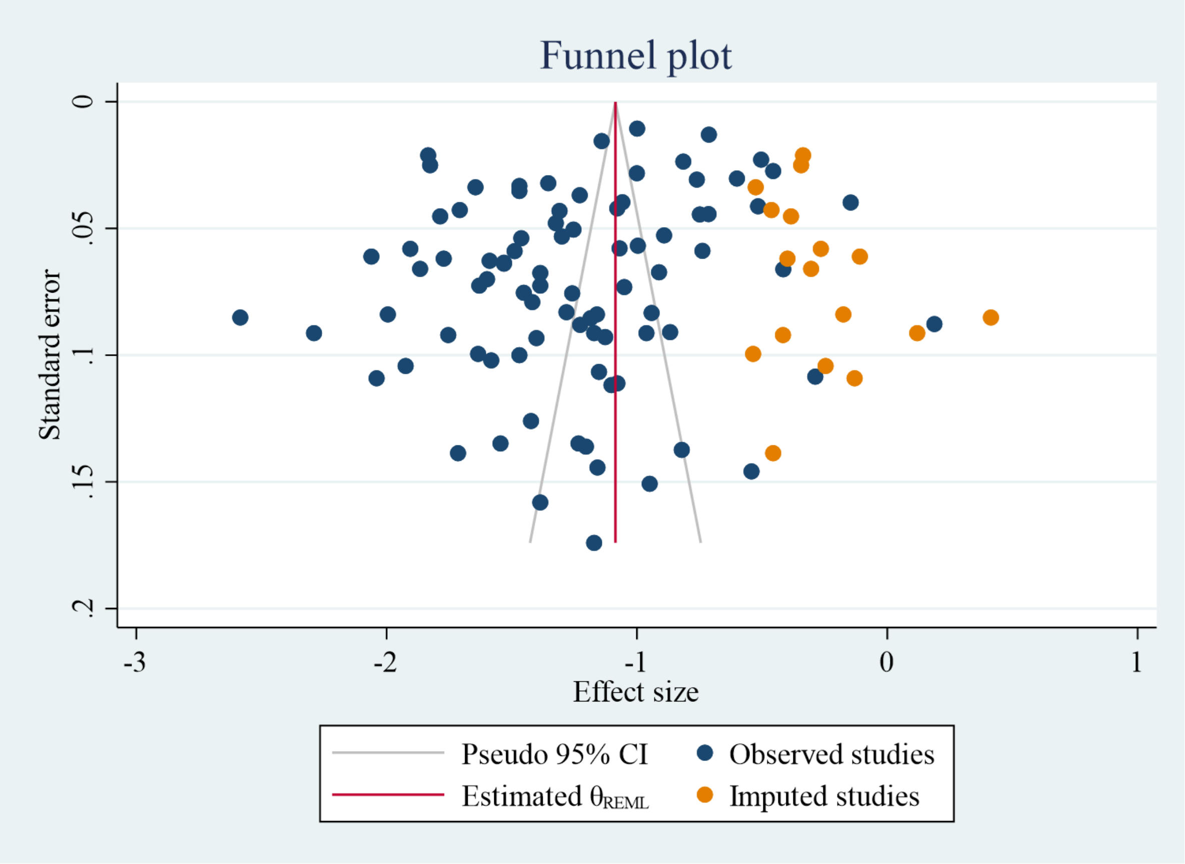

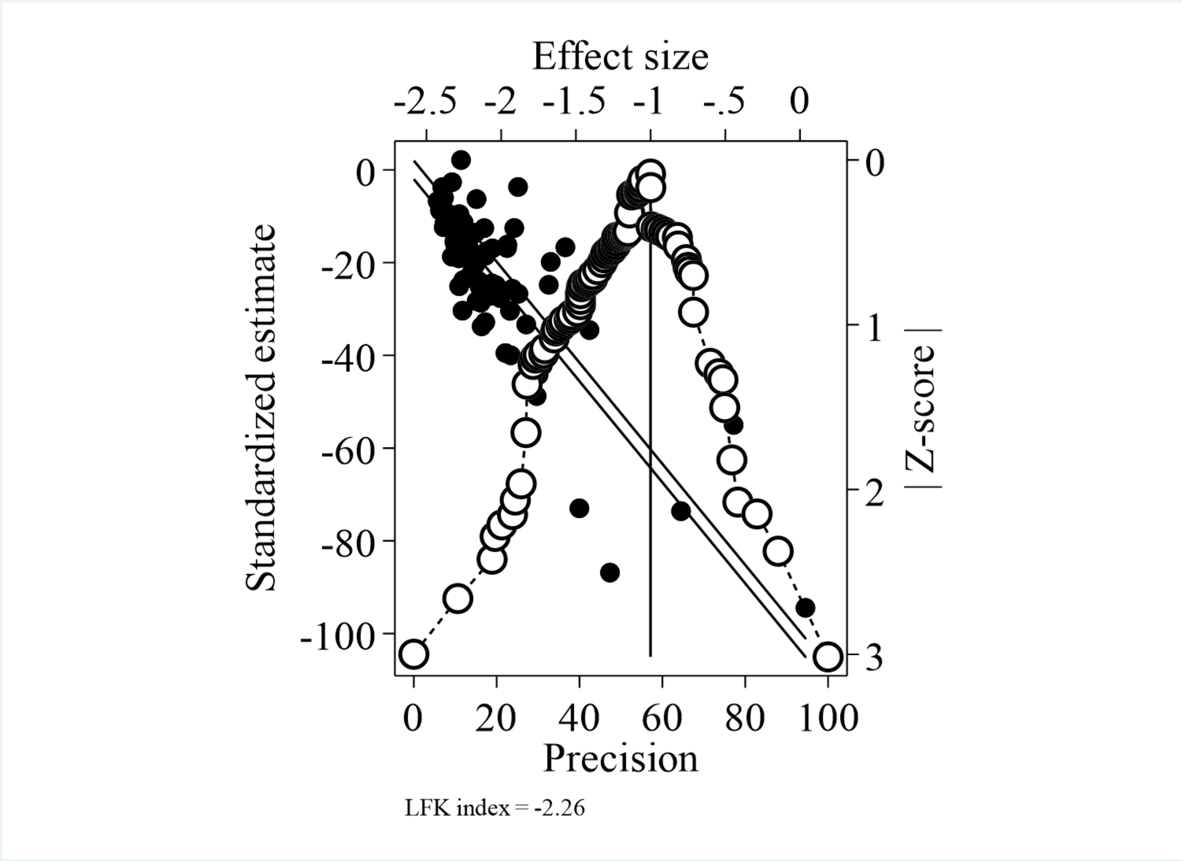

Figure S4 Funnel Plot and Doi Plot of Incidence Rate Estimation of PD-Associated Peritonitis (Continued)

H. Pediatric

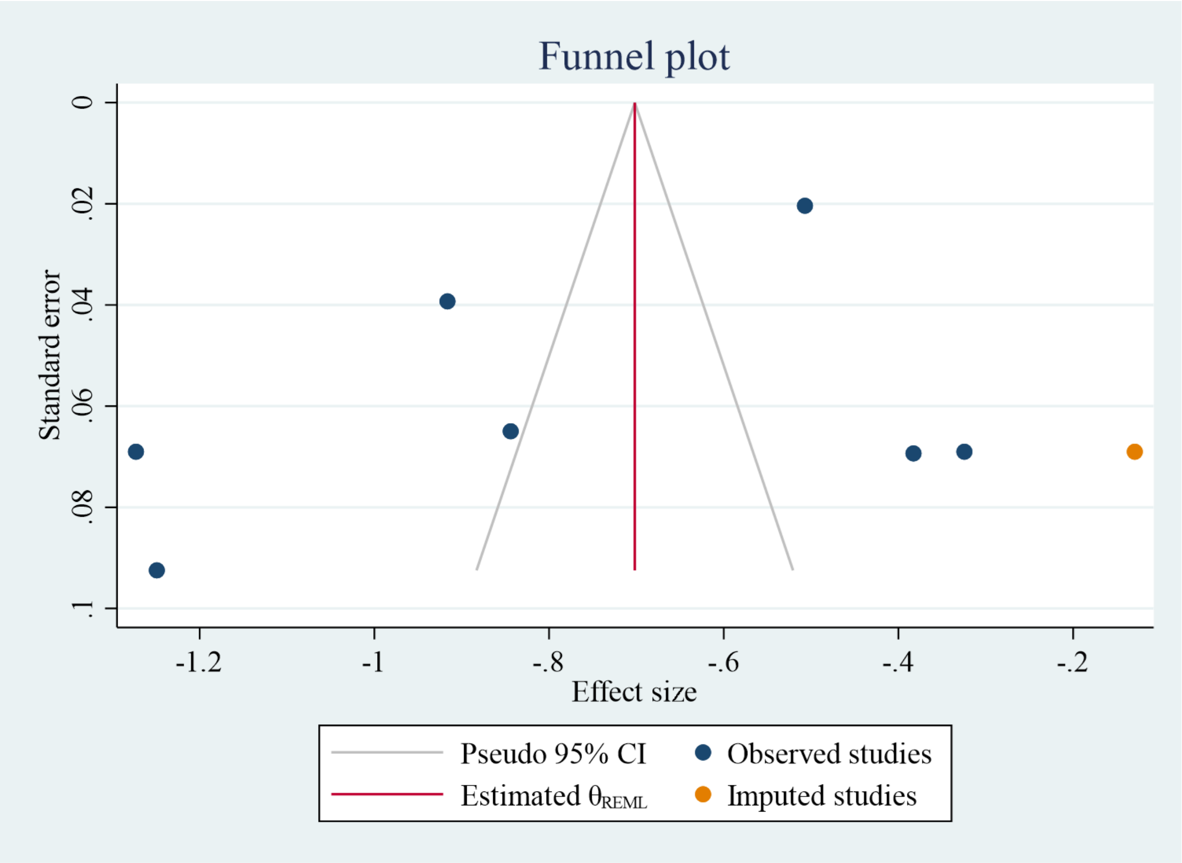

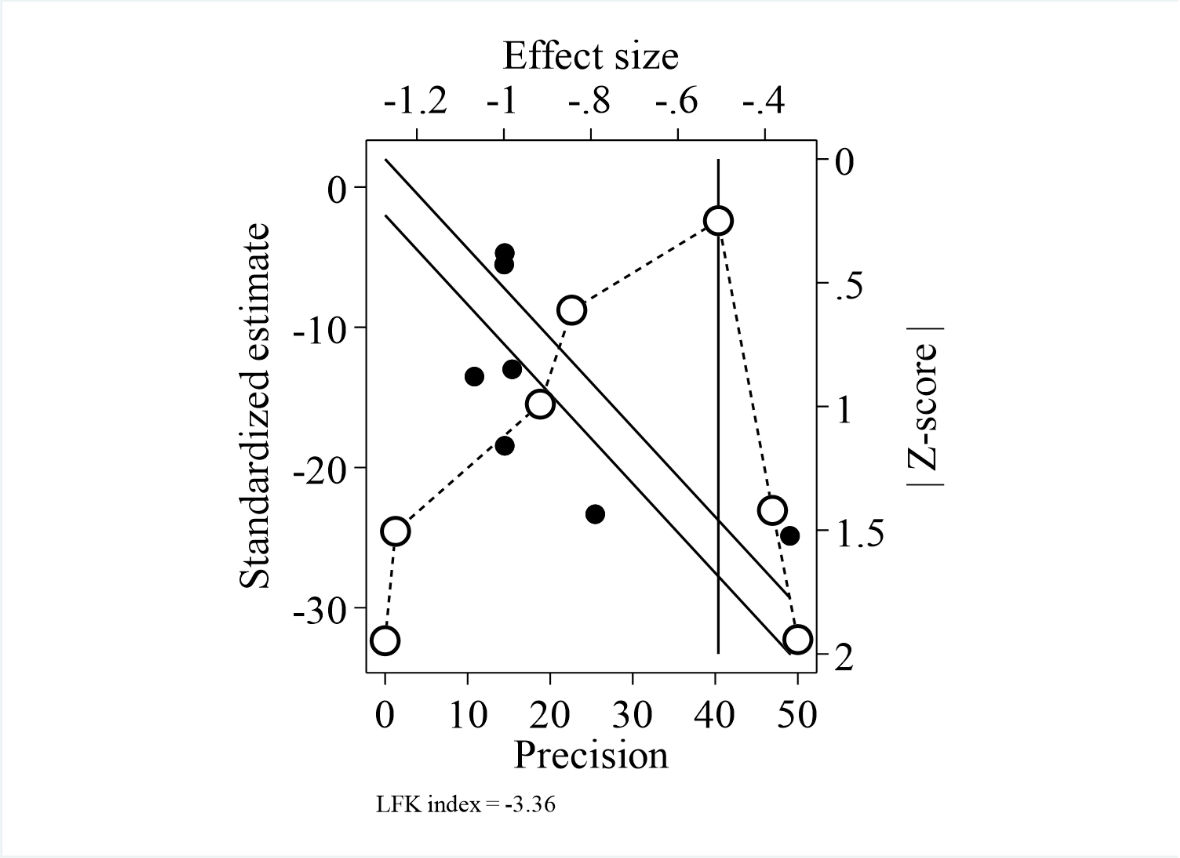

Figure S4 Funnel Plot and Doi Plot of Incidence Rate Estimation of PD-Associated Peritonitis (Continued)

I. Mixed Population

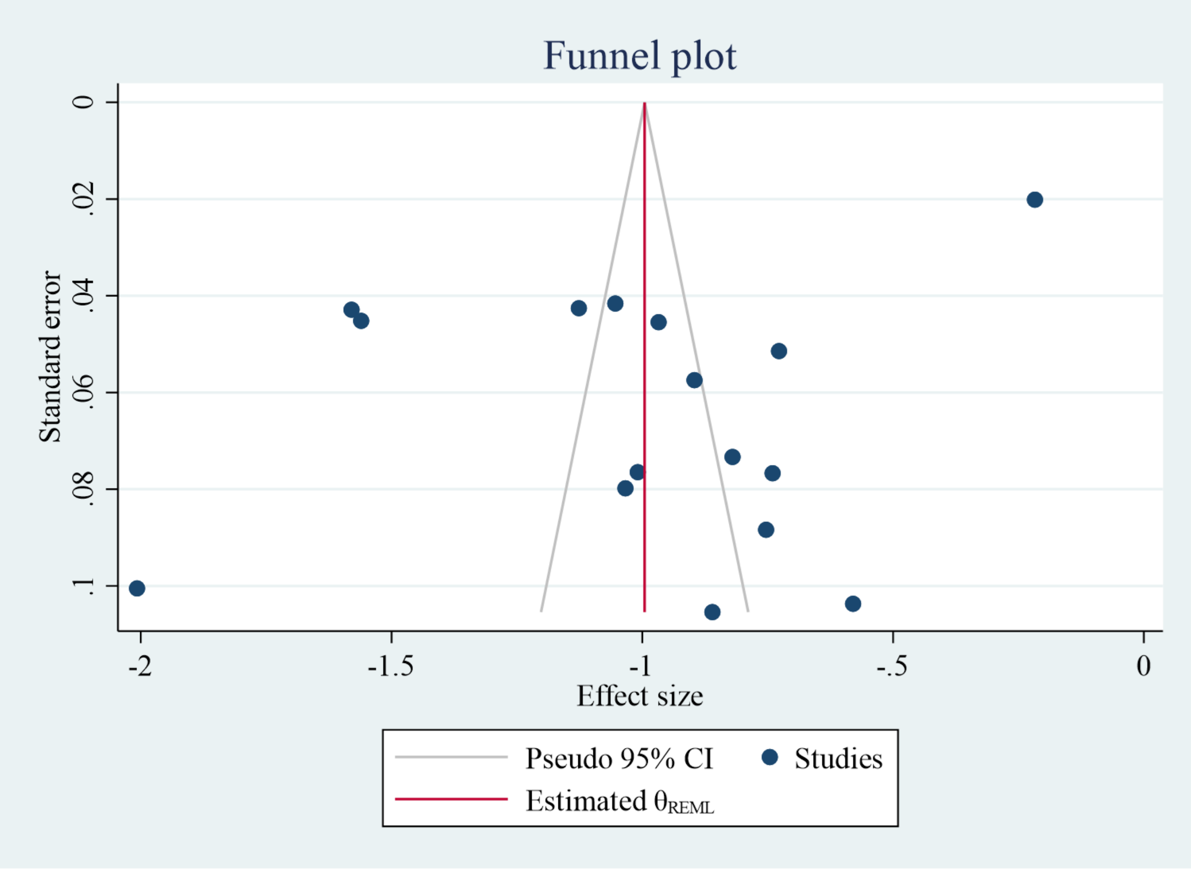

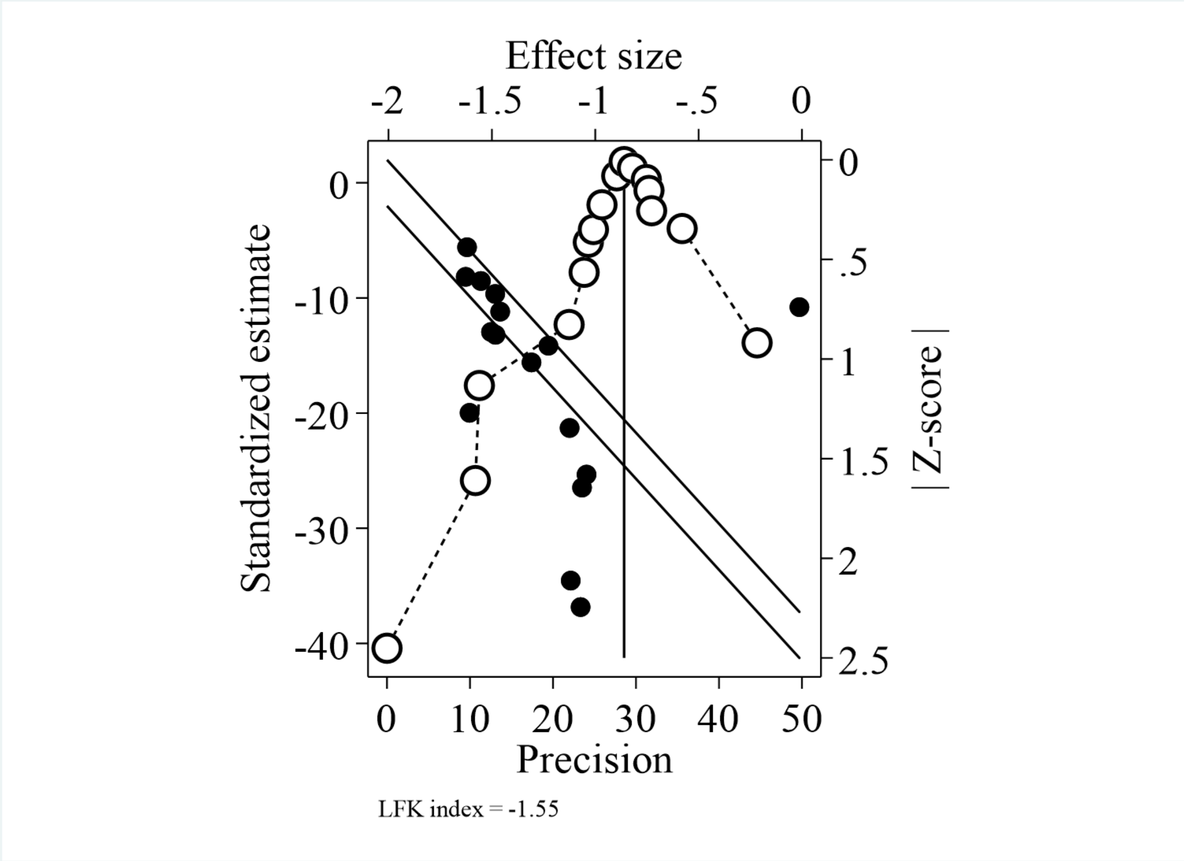

Figure S4 Funnel Plot and Doi Plot of Incidence Rate Estimation of PD-Associated Peritonitis (Continued)

J. Not Specified Population

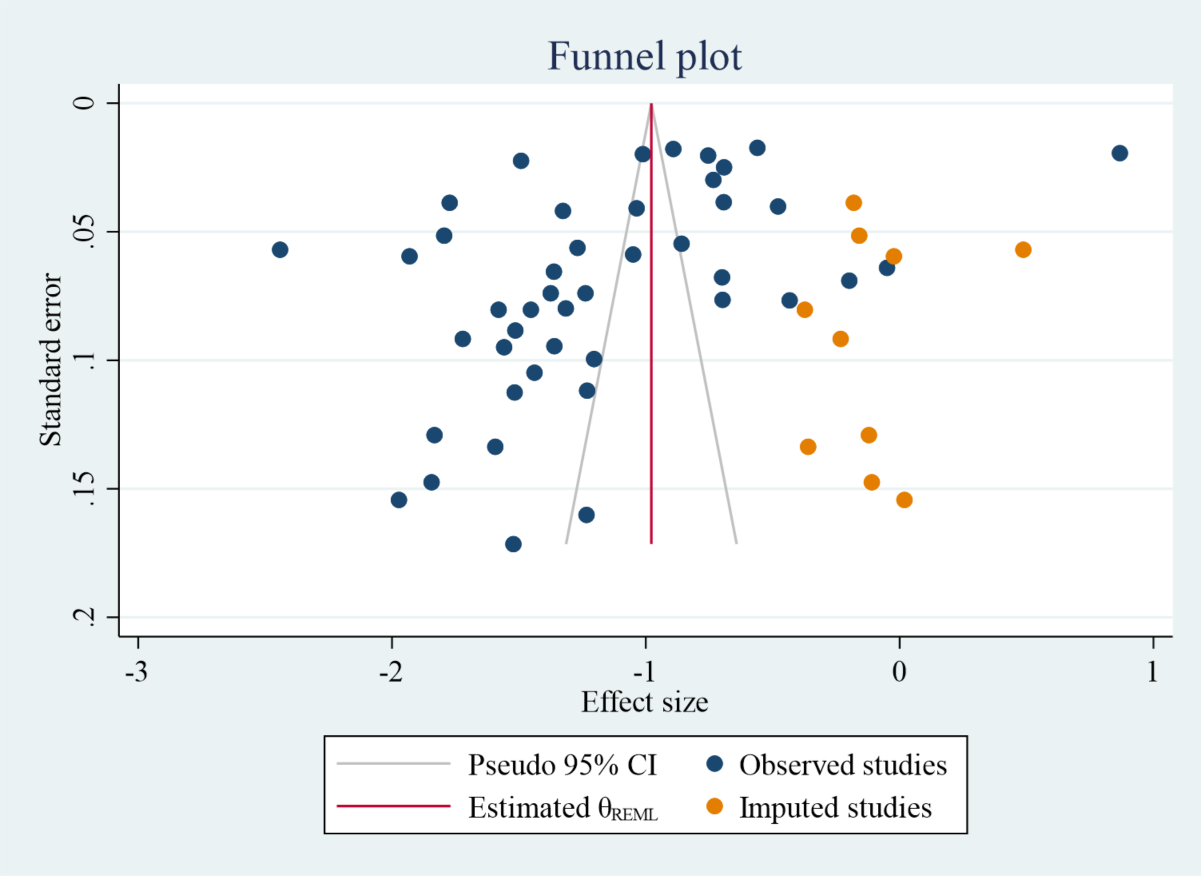

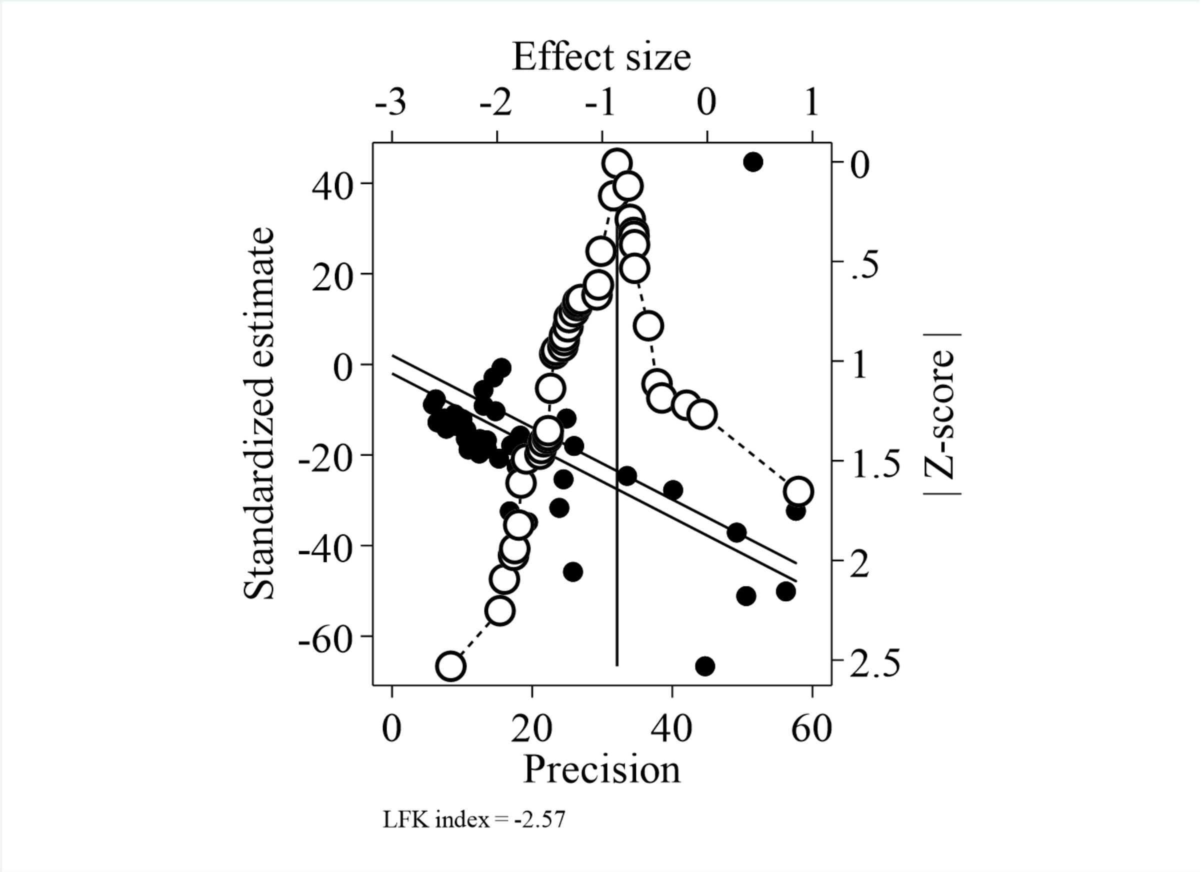

Figure S4 Funnel Plot and Doi Plot of Incidence Rate Estimation of PD-Associated Peritonitis (Continued)

K. CAPD

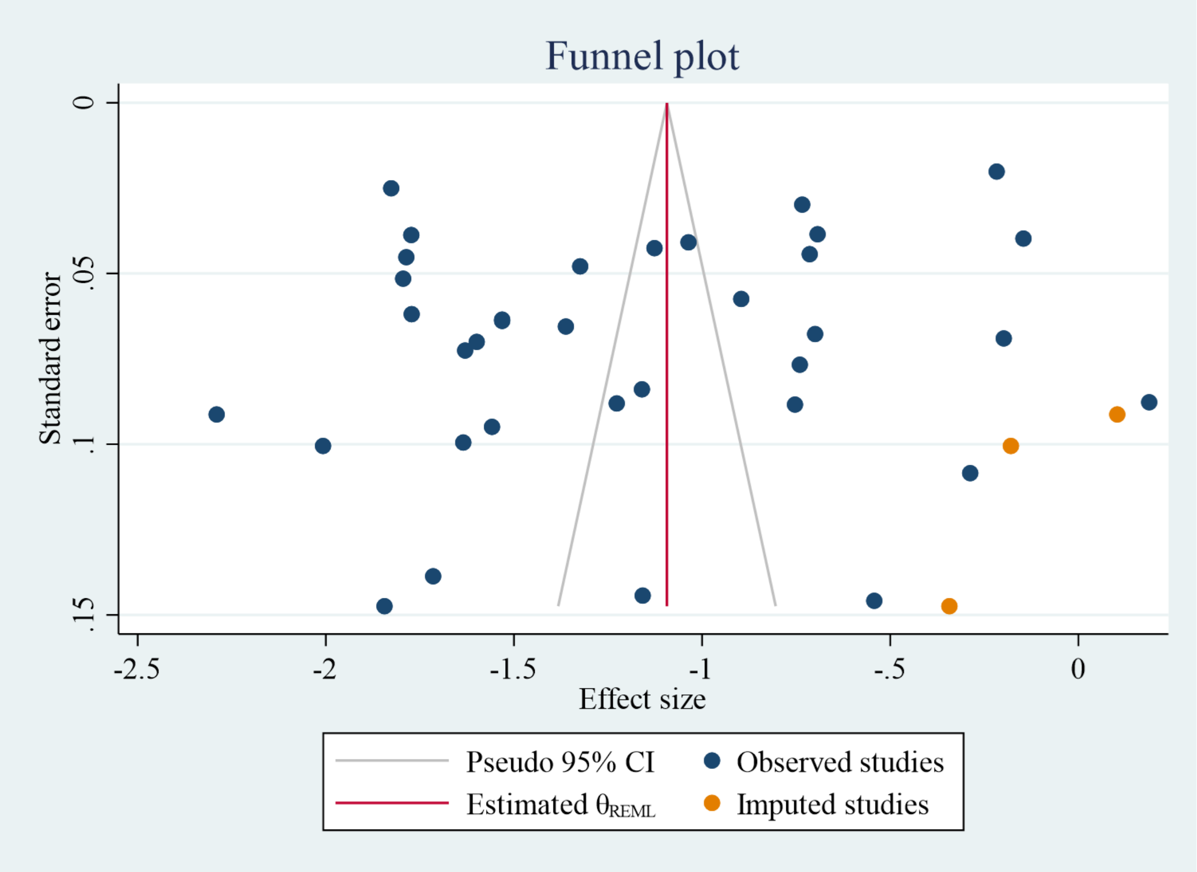

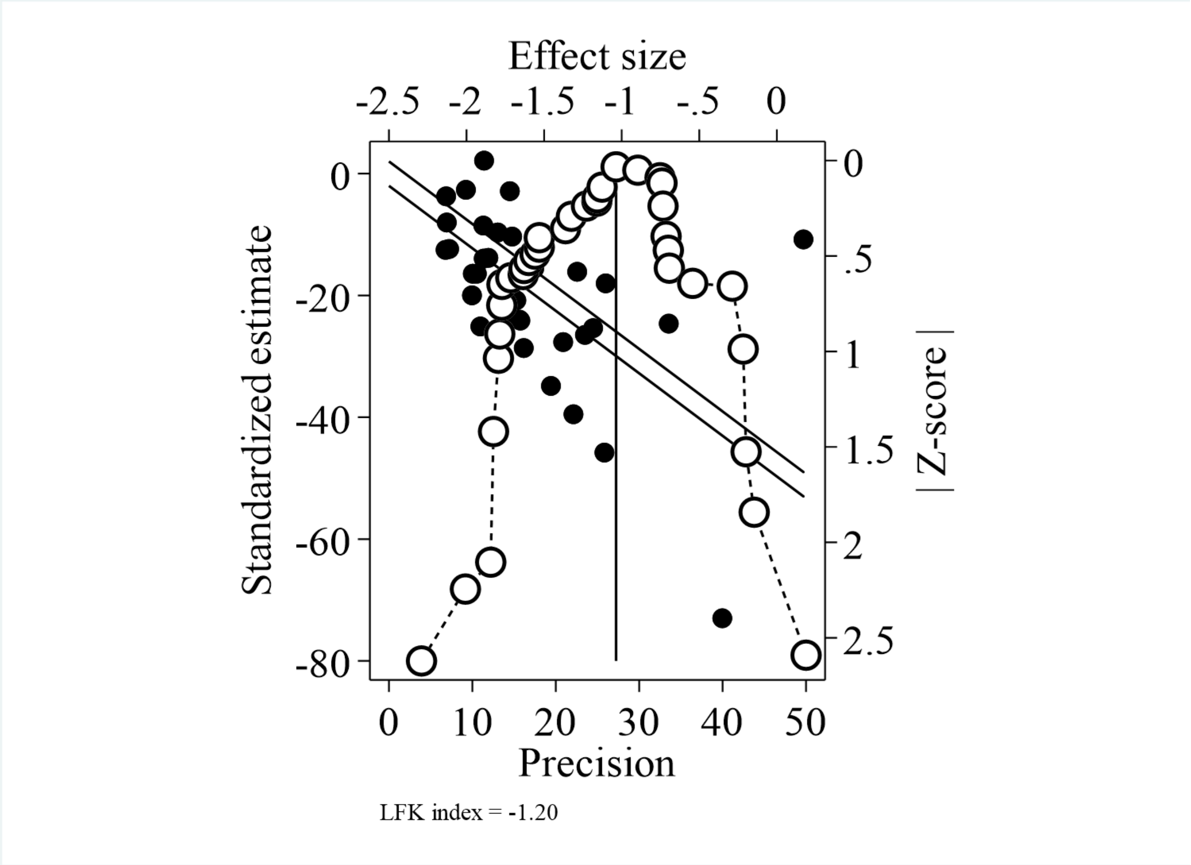

Figure S4 Funnel Plot and Doi Plot of Incidence Rate Estimation of PD-Associated Peritonitis (Continued)

L. Mixed Modality

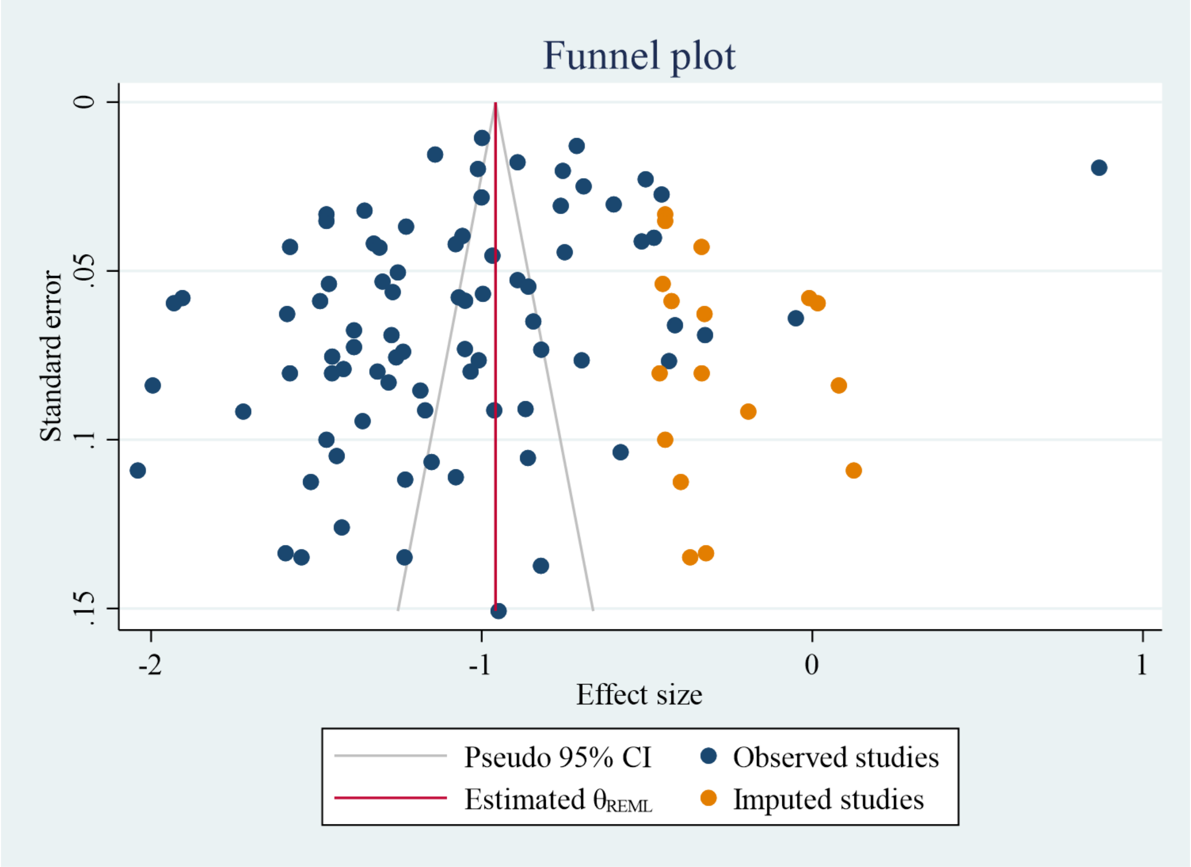

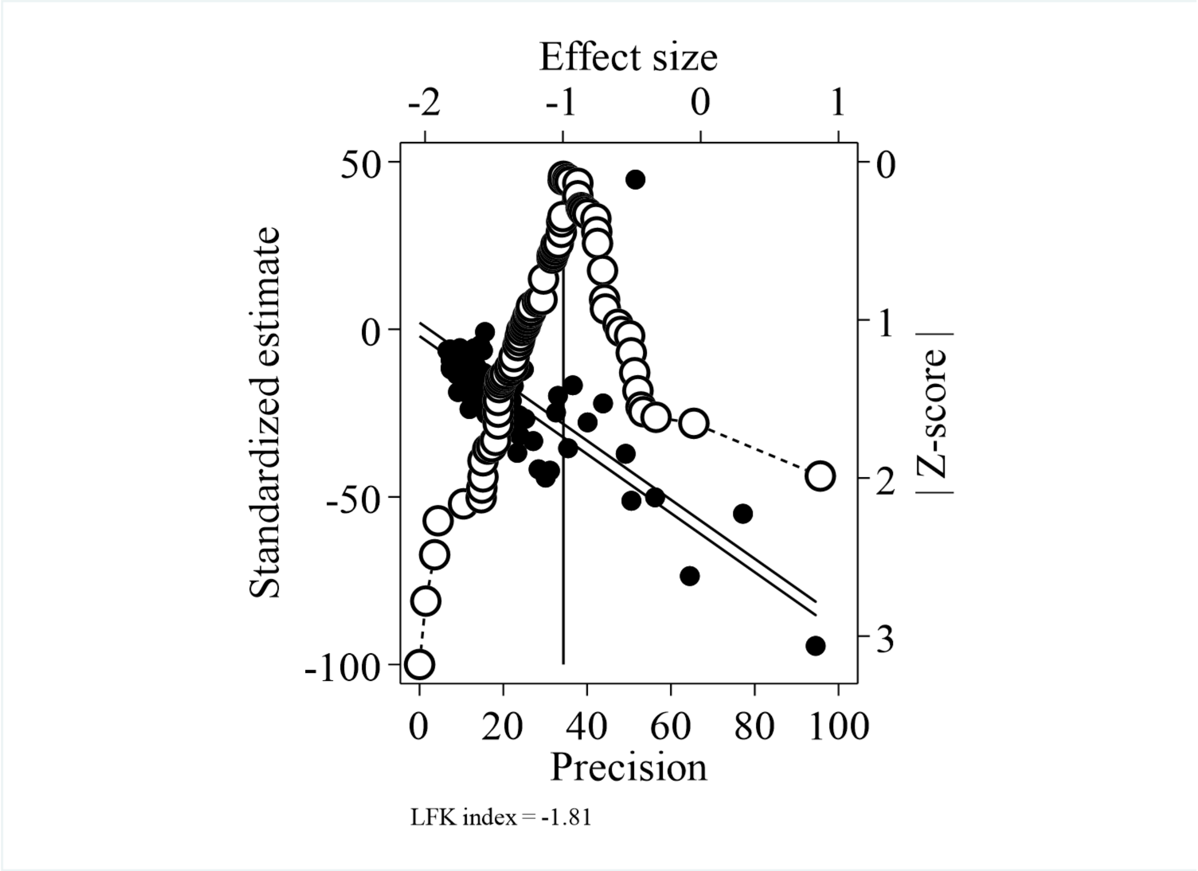

Figure S4 Funnel Plot and Doi Plot of Incidence Rate Estimation of PD-Associated Peritonitis (Continued)

M. Not Specified Modality

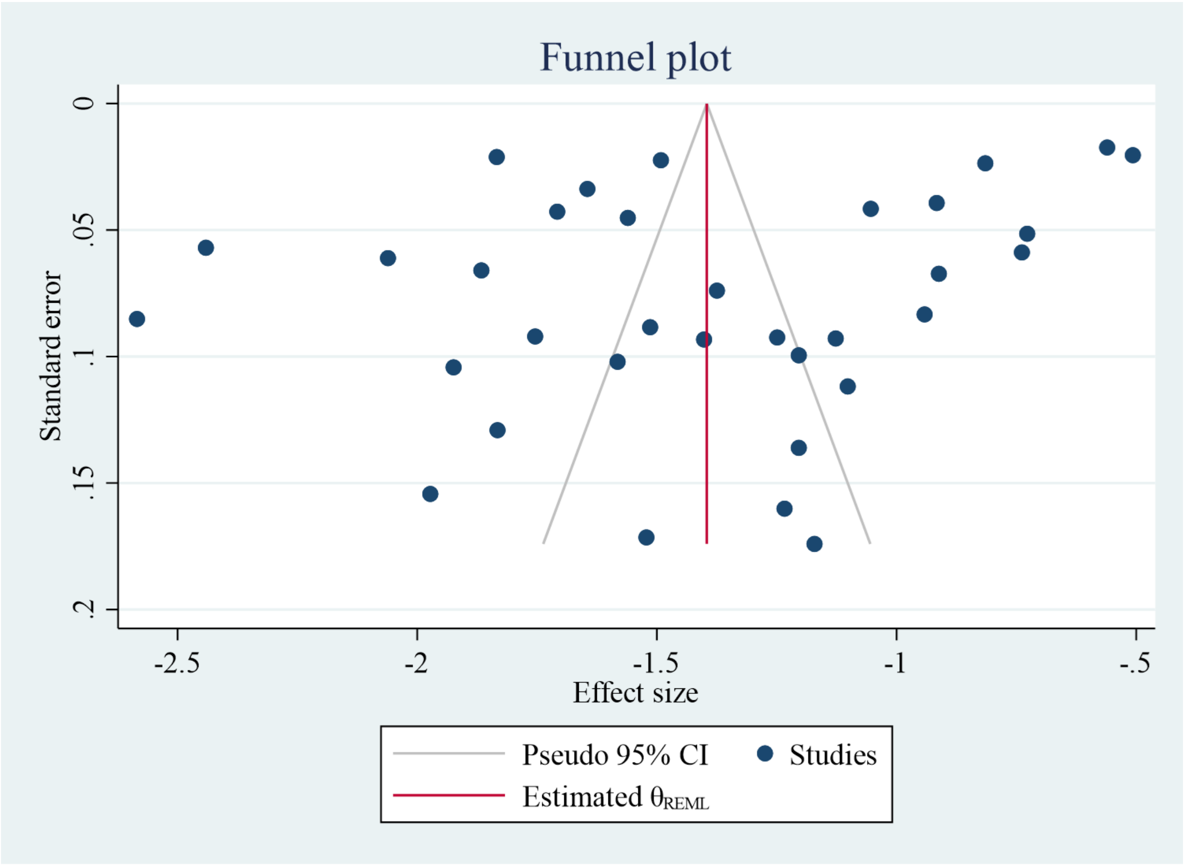

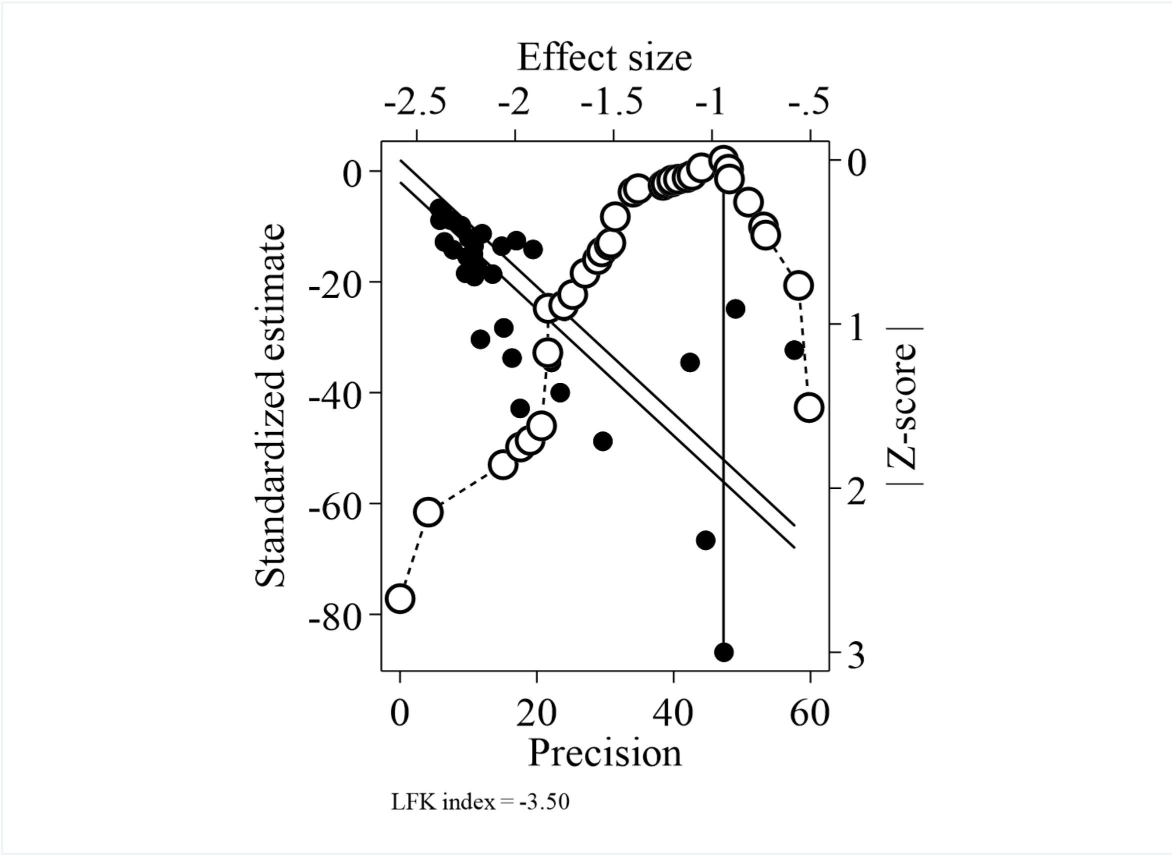

Figure S4 Funnel Plot and Doi Plot of Incidence Rate Estimation of PD-Associated Peritonitis (Continued)

M. Gram-Positive Bacteria

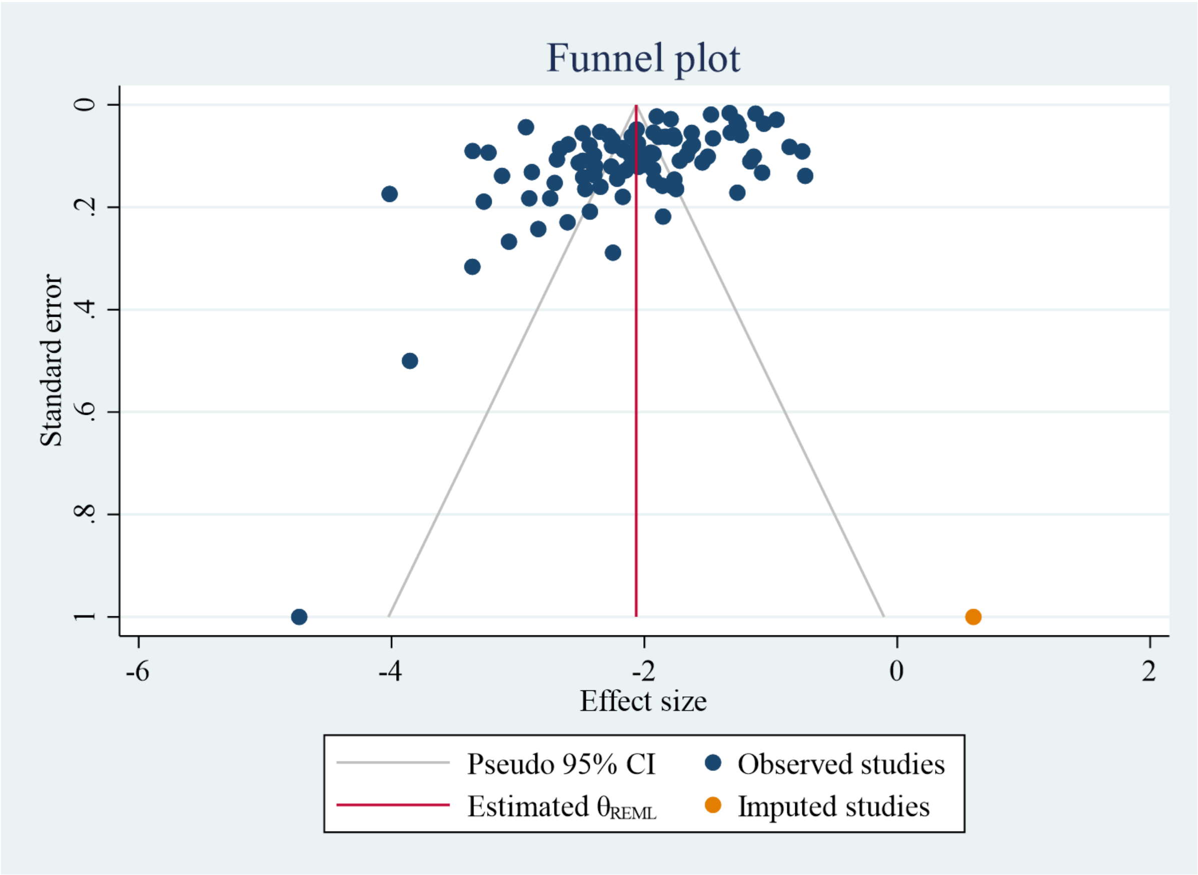

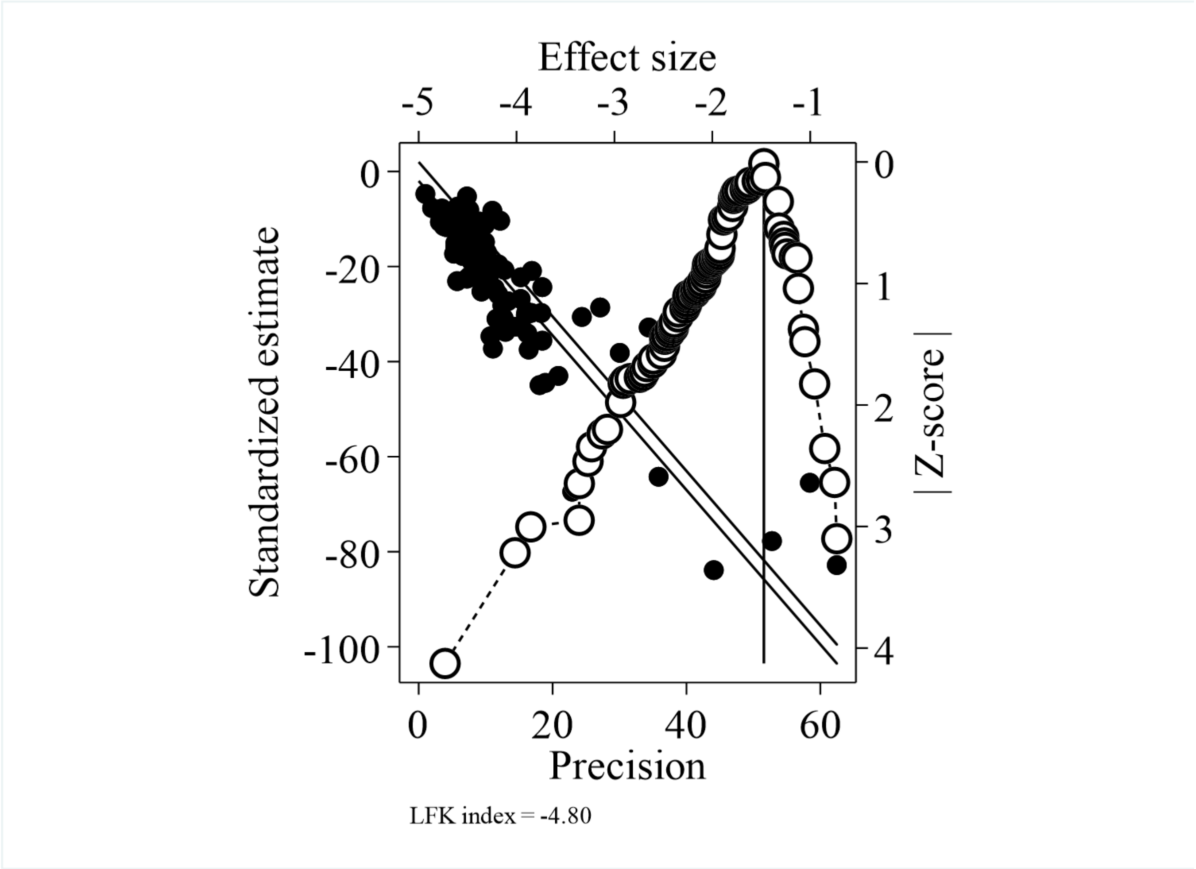

Figure S4 Funnel Plot and Doi Plot of Incidence Rate Estimation of PD-Associated Peritonitis (Continued)

N. Gram-Negative Bacteria

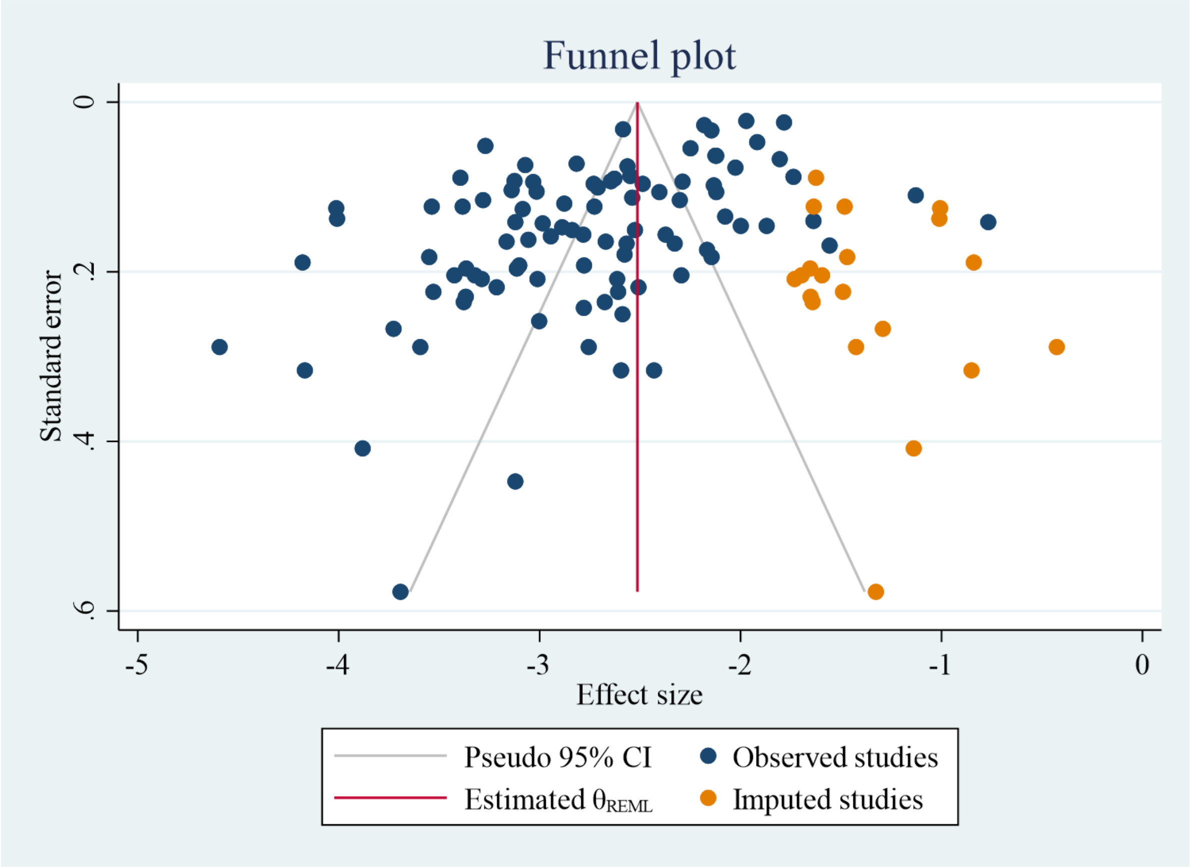

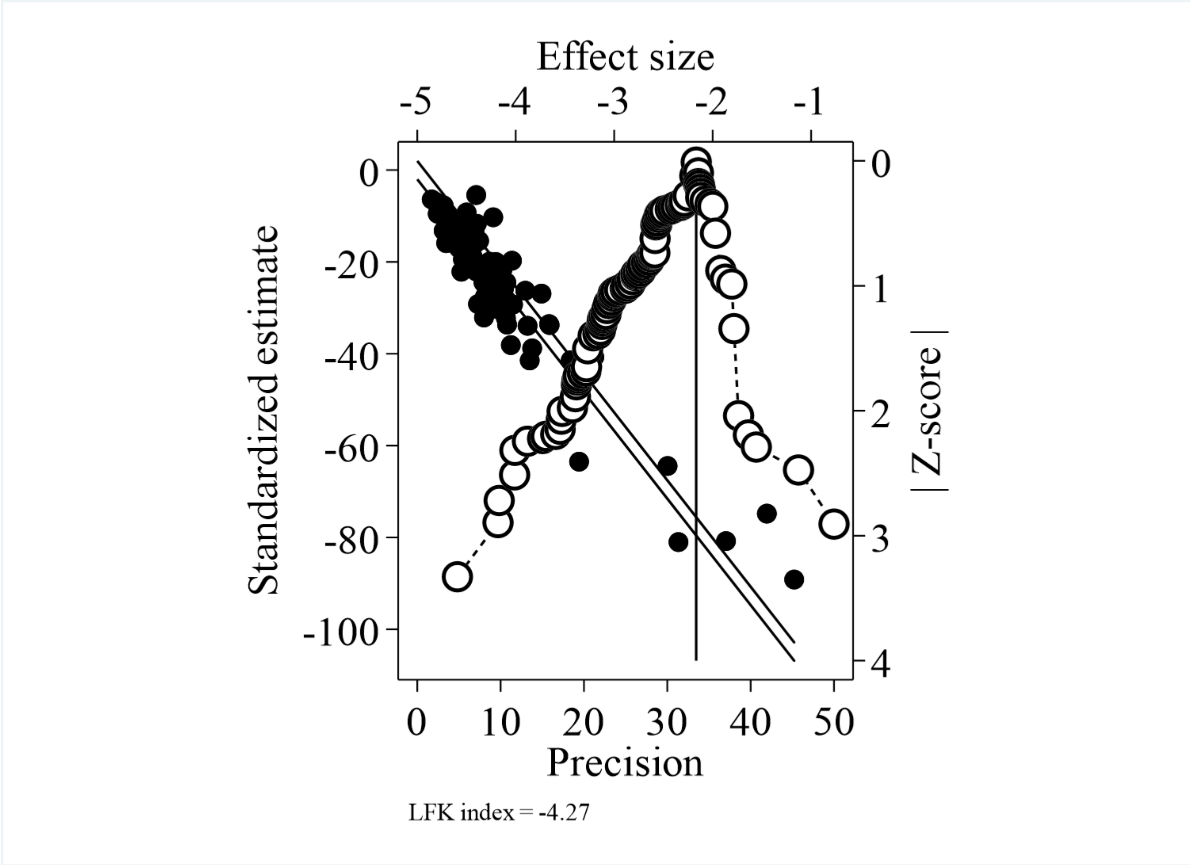

Figure S4 Funnel Plot and Doi Plot of Incidence Rate Estimation of PD-Associated Peritonitis (Continued)

O. Fungus

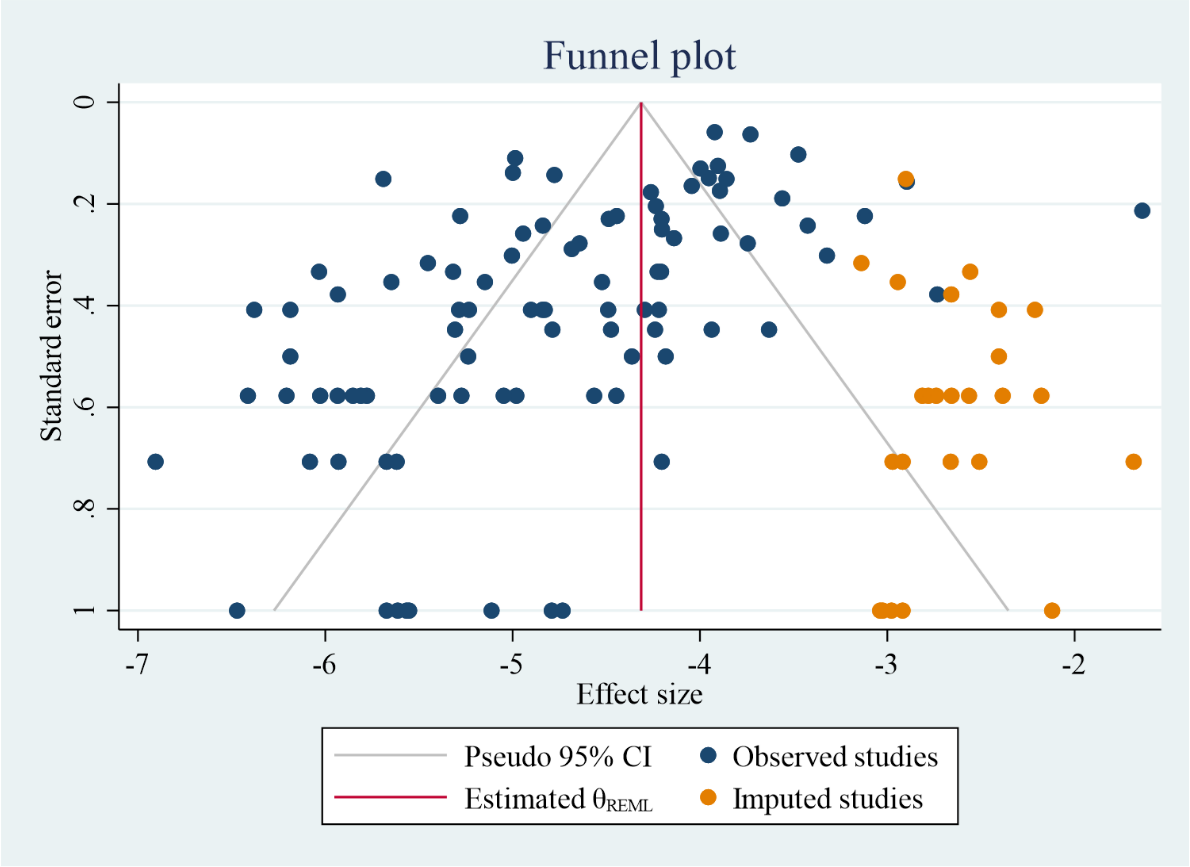

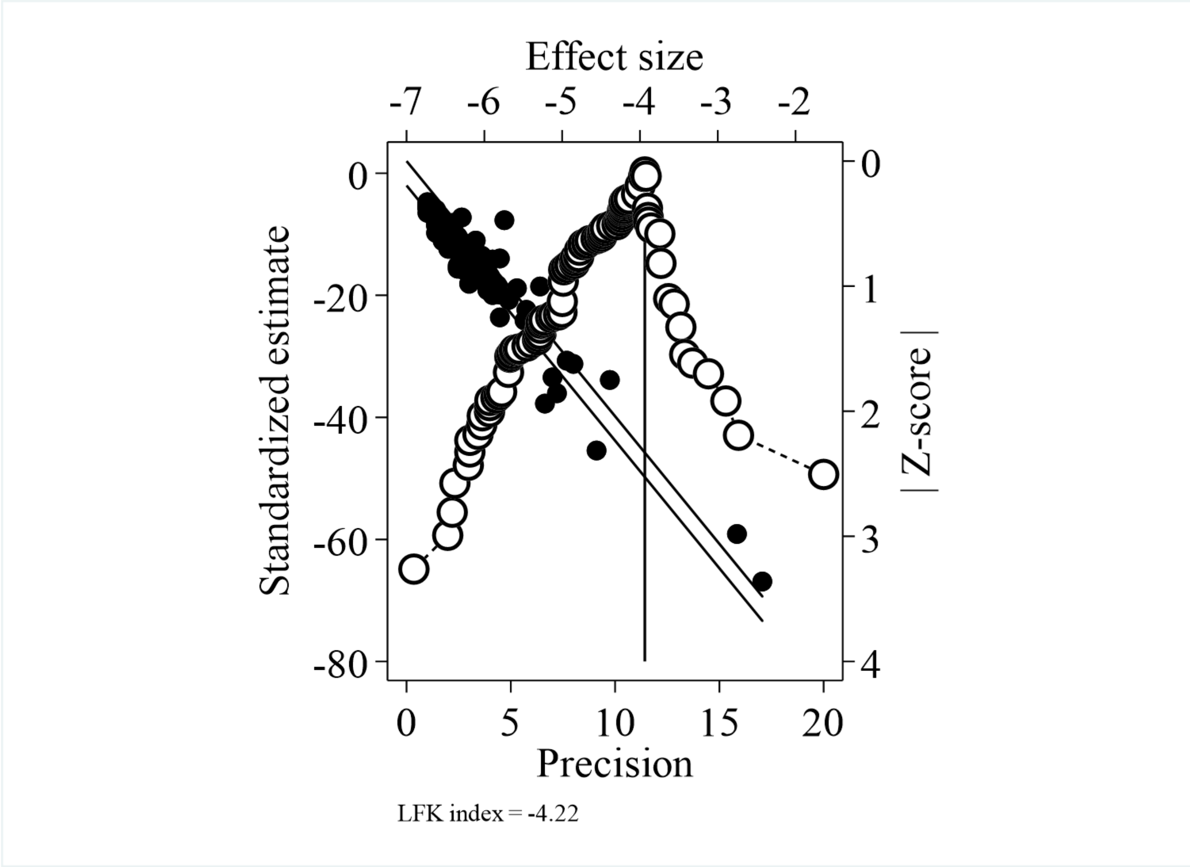

Figure S4 Funnel Plot and Doi Plot of Incidence Rate Estimation of PD-Associated Peritonitis (Continued)

P. Mycobacterium

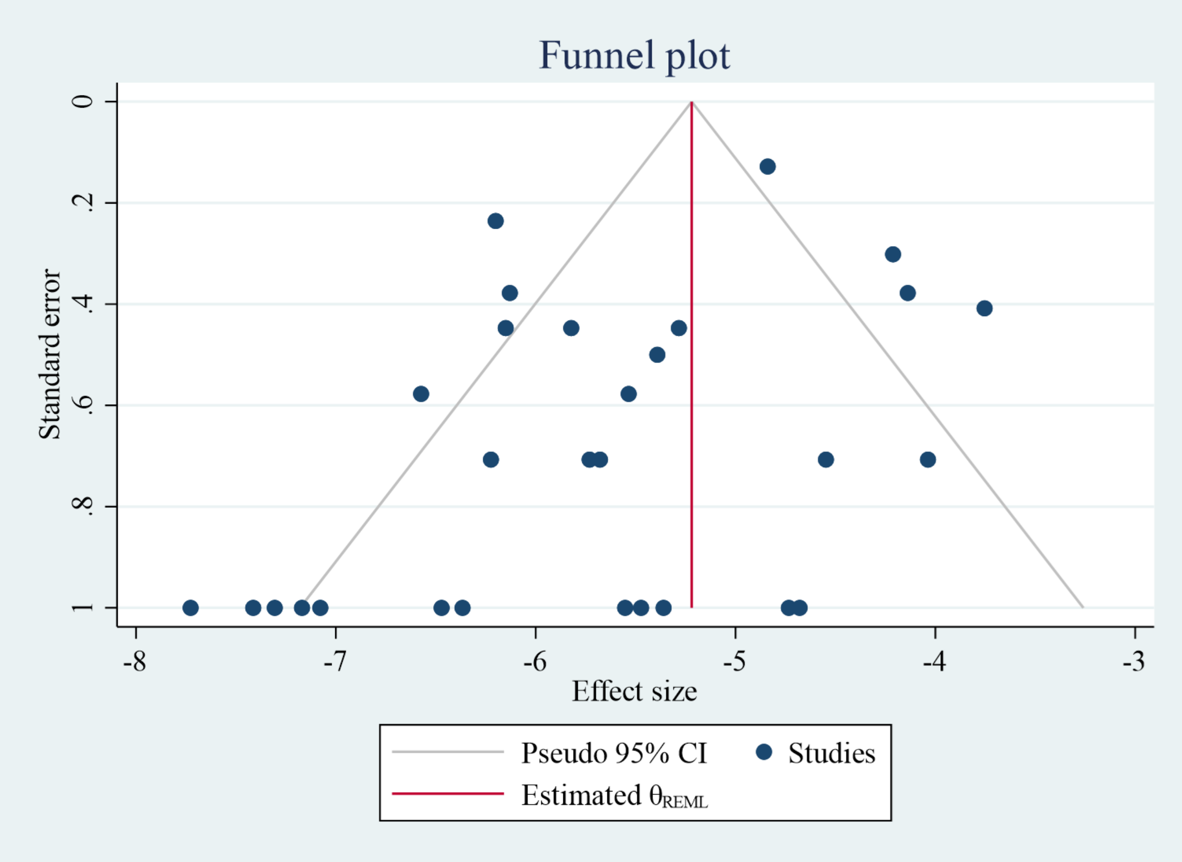

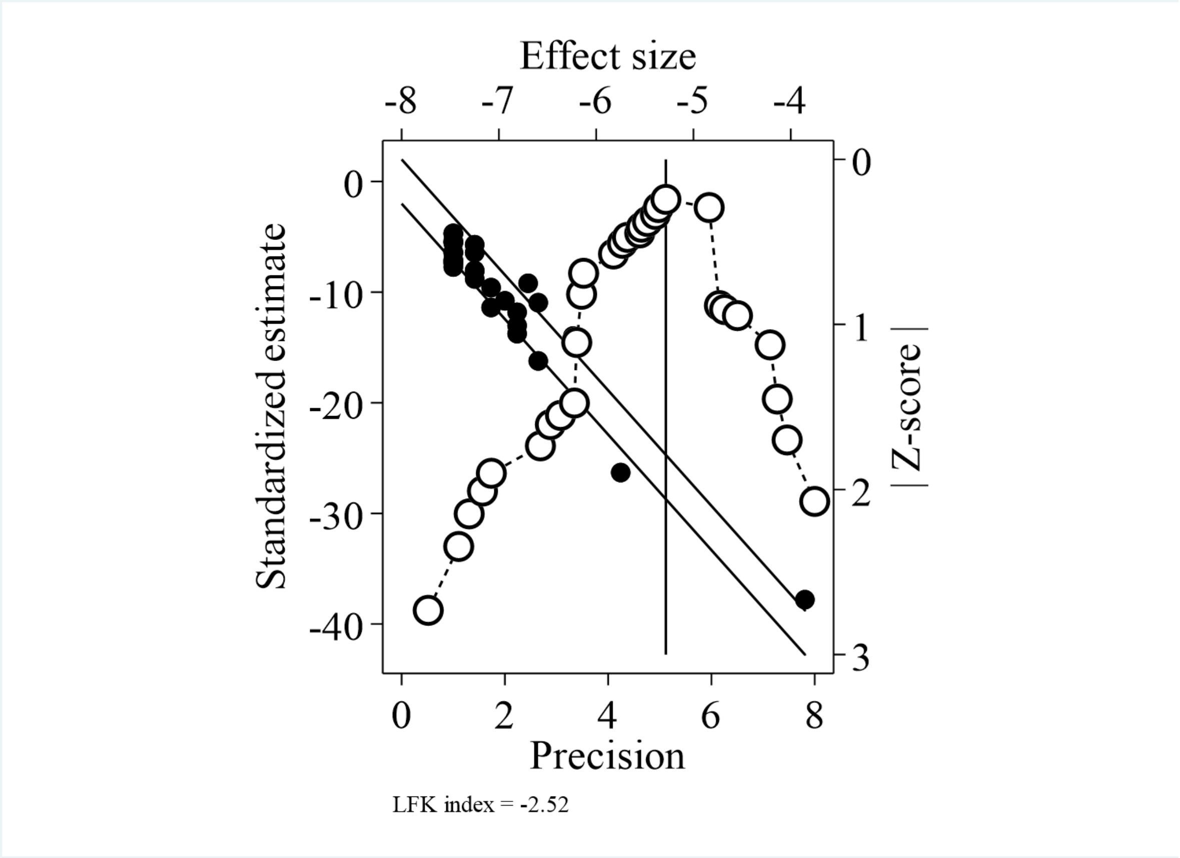

Figure S4 Funnel Plot and Doi Plot of Incidence Rate Estimation of PD-Associated Peritonitis (Continued)

Q. Polymicrobial

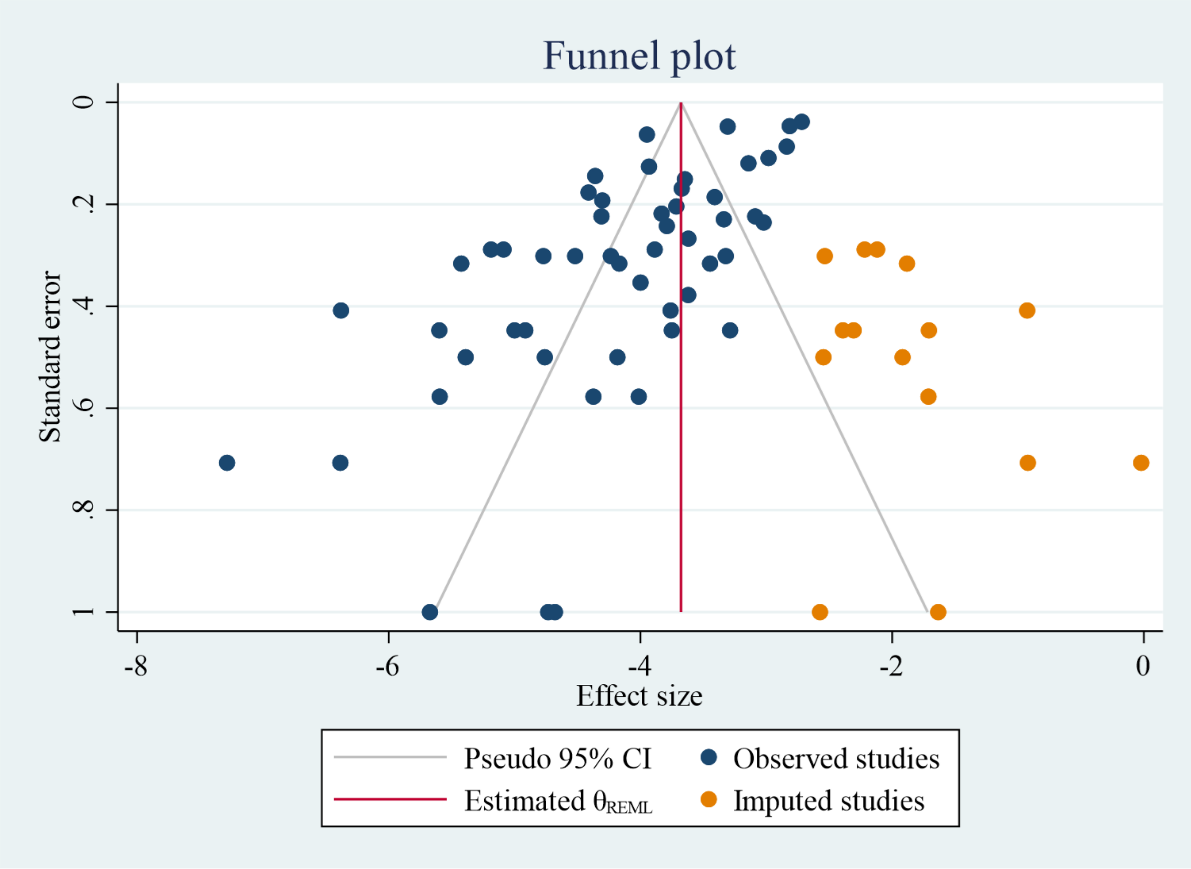

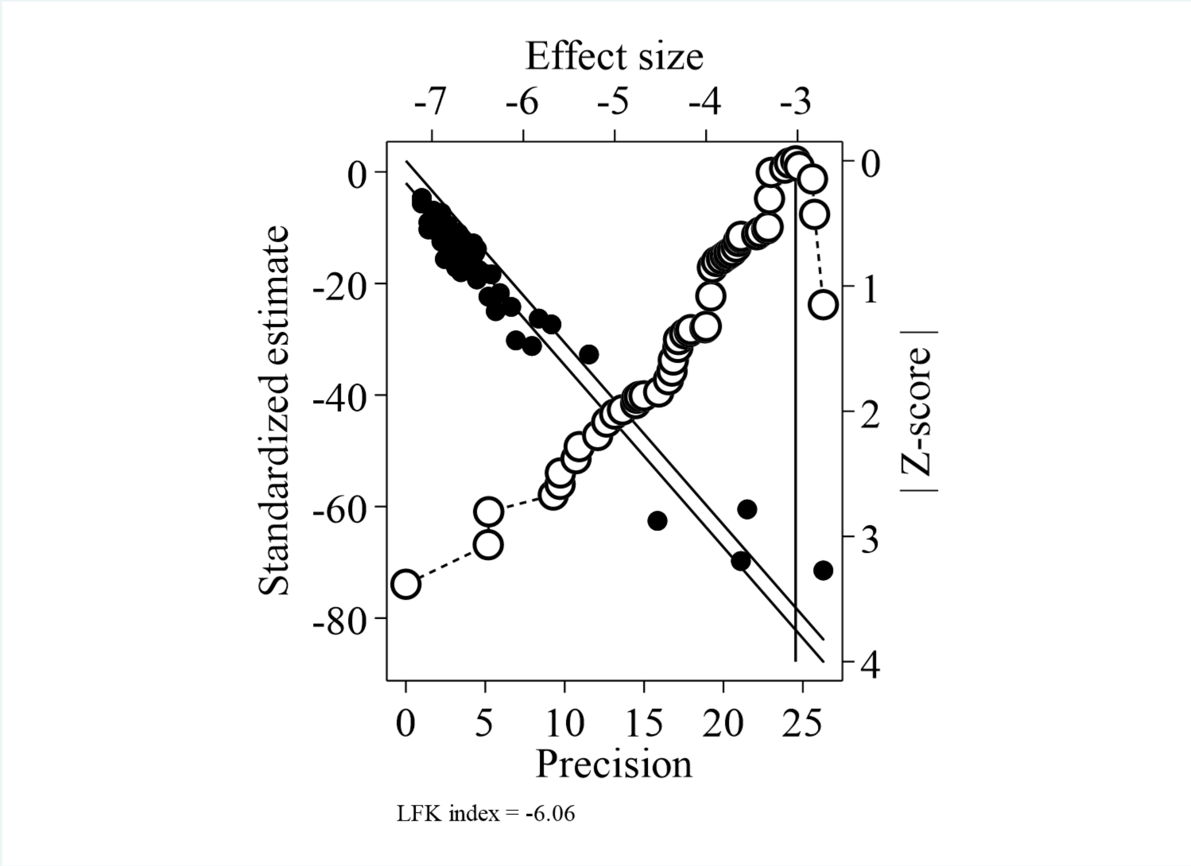

Figure S4 Funnel Plot and Doi Plot of Incidence Rate Estimation of PD-Associated Peritonitis (Continued)

R. Culture Negative

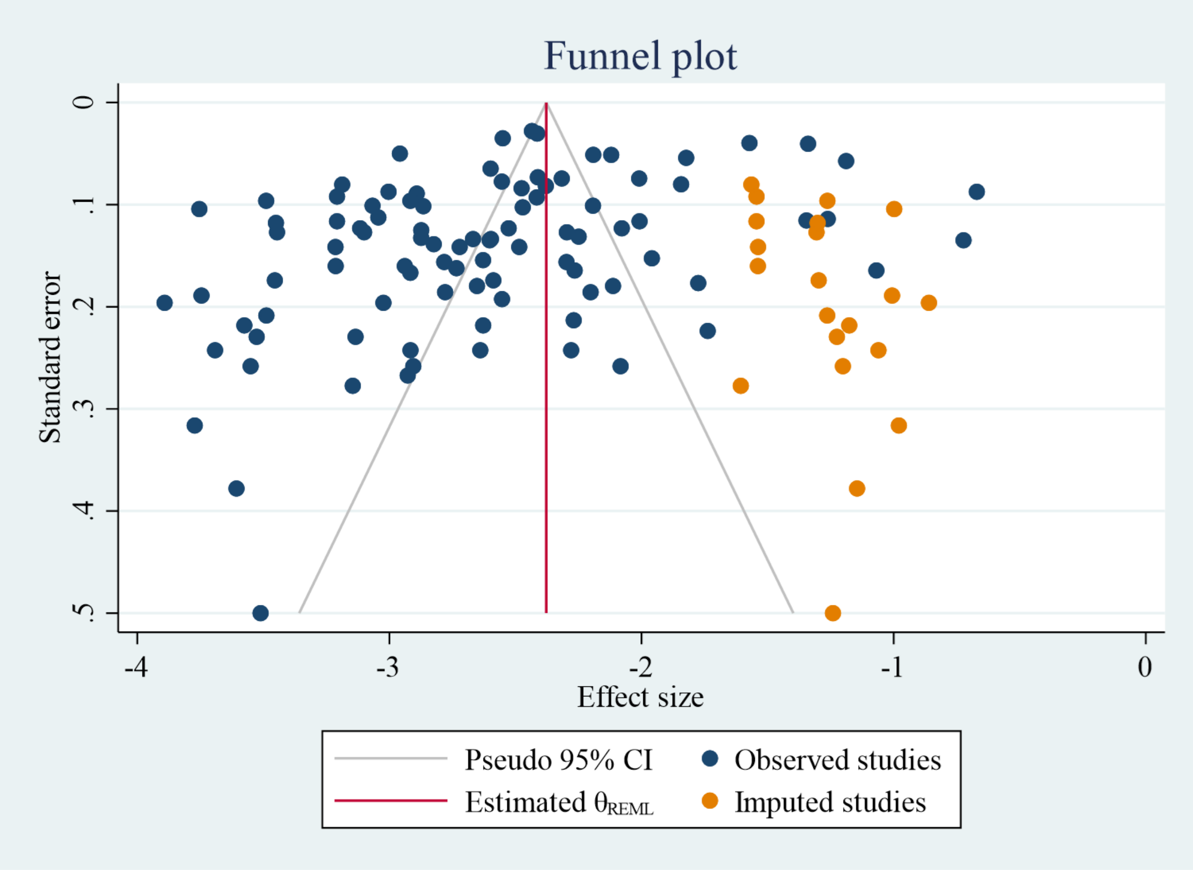

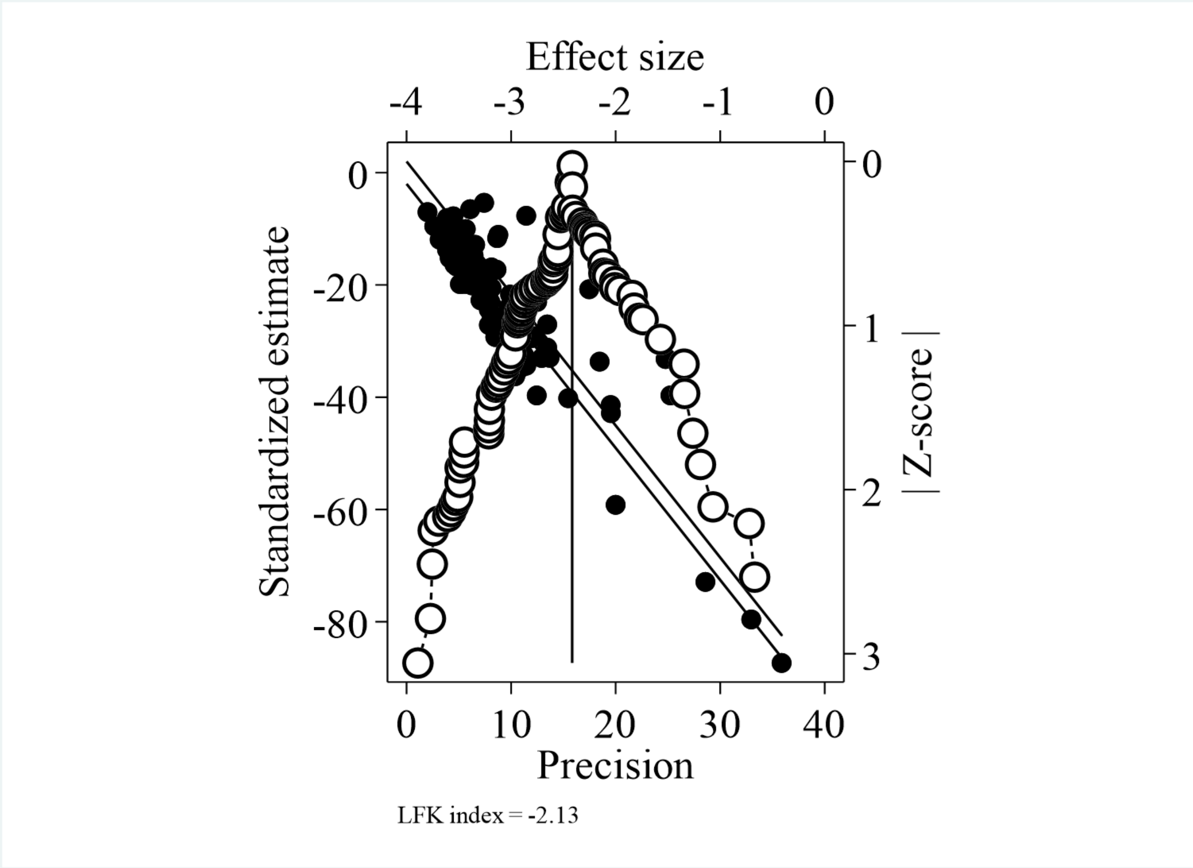

File S1 PRISMA 2020 Statement Checklist[151]

Section and Topic	Item #	Checklist item	Location where item is reported
**TITLE**			
Title	1	Identify the report as a systematic review.	Page 1
**ABSTRACT**			
Abstract	2	See the PRISMA 2020 for Abstracts checklist.	Page 1,2
**INTRODUCTION**			
Rationale	3	Describe the rationale for the review in the context of existing knowledge.	Page 2,3
Objectives	4	Provide an explicit statement of the objective(s) or question(s) the review addresses.	Page 3
**METHODS**			
Eligibility criteria	5	Specify the inclusion and exclusion criteria for the review and how studies were grouped for the syntheses.	Page 3,4 Table S2
Information sources	6	Specify all databases, registers, websites, organisations, reference lists and other sources searched or consulted to identify studies. Specify the date when each source was last searched or consulted.	Page 3<br>Table S1
Search strategy	7	Present the full search strategies for all databases, registers and websites, including any filters and limits used.	Table S1
Selection process	8	Specify the methods used to decide whether a study met the inclusion criteria of the review, including how many reviewers screened each record and each report retrieved, whether they worked independently, and if applicable, details of automation tools used in the process.	Page 3,4
Data collection process	9	Specify the methods used to collect data from reports, including how many reviewers collected data from each report, whether they worked independently, any processes for obtaining or confirming data from study investigators, and if applicable, details of automation tools used in the process.	Page 3.4
Data items	10a	List and define all outcomes for which data were sought. Specify whether all results that were compatible with each outcome domain in each study were sought (e.g. for all measures, time points, analyses), and if not, the methods used to decide which results to collect.	Page 4
	10b	List and define all other variables for which data were sought (e.g. participant and intervention characteristics, funding sources). Describe any assumptions made about any missing or unclear information.	Page 4
Study risk of bias assessment	11	Specify the methods used to assess risk of bias in the included studies, including details of the tool(s) used, how many reviewers assessed each study and whether they worked independently, and if applicable, details of automation tools used in the process.	Page 4
Effect measures	12	Specify for each outcome the effect measure(s) (e.g. risk ratio, mean difference) used in the synthesis or presentation of results.	Page 4,5
Synthesis methods	13a	Describe the processes used to decide which studies were eligible for each synthesis (e.g. tabulating the study intervention characteristics and comparing against the planned groups for each synthesis (item #5)).	Page 4,5
	13b	Describe any methods required to prepare the data for presentation or synthesis, such as handling of missing summary statistics, or data conversions.	Page 4,5
	13c	Describe any methods used to tabulate or visually display results of individual studies and syntheses.	Page 4,5
	13d	Describe any methods used to synthesize results and provide a rationale for the choice(s). If meta-analysis was performed, describe the model(s), method(s) to identify the presence and extent of statistical heterogeneity, and software package(s) used.	Page 4,5
	13e	Describe any methods used to explore possible causes of heterogeneity among study results (e.g. subgroup analysis, meta-regression).	Page 5
	13f	Describe any sensitivity analyses conducted to assess robustness of the synthesized results.	Page 5
Reporting bias assessment	14	Describe any methods used to assess risk of bias due to missing results in a synthesis (arising from reporting biases).	Page 4,5

Section and Topic	Item #	Checklist item	Location where item is reported
Certainty assessment	15	Describe any methods used to assess certainty (or confidence) in the body of evidence for an outcome.	NA
**RESULTS**			
Study selection	16a	Describe the results of the search and selection process, from the number of records identified in the search to the number of studies included in the review, ideally using a flow diagram.	Page 5<br>Figure 1
	16b	Cite studies that might appear to meet the inclusion criteria, but which were excluded, and explain why they were excluded.	Table S3
Study characteristics	17	Cite each included study and present its characteristics.	Page 6, Table 1<br>Table S4
Risk of bias in studies	18	Present assessments of risk of bias for each included study.	Table 1, Table S5
Results of individual studies	19	For all outcomes, present, for each study: (a) summary statistics for each group (where appropriate) and (b) an effect estimate and its precision (e.g. confidence/credible interval), ideally using structured tables or plots.	Table 2<br>Figure 2, 3, 4
Results of syntheses	20a	For each synthesis, briefly summarise the characteristics and risk of bias among contributing studies.	Page 8,12,13<br>Figure 2,3,4
	20b	Present results of all statistical syntheses conducted. If meta-analysis was done, present for each the summary estimate and its precision (e.g. confidence/credible interval) and measures of statistical heterogeneity. If comparing groups, describe the direction of the effect.	Page 8,12,13<br>Figure 2,3,4
	20c	Present results of all investigations of possible causes of heterogeneity among study results.	Page 12,13, Table 2
	20d	Present results of all sensitivity analyses conducted to assess the robustness of the synthesized results.	Page 12,13, Table S7
Reporting biases	21	Present assessments of risk of bias due to missing results (arising from reporting biases) for each synthesis assessed.	Page 13,14, Table S8, Figure S4
Certainty of evidence	22	Present assessments of certainty (or confidence) in the body of evidence for each outcome assessed.	NA
**DISCUSSION**			
Discussion	23a	Provide a general interpretation of the results in the context of other evidence.	Page 13
	23b	Discuss any limitations of the evidence included in the review.	Page 14,15
	23c	Discuss any limitations of the review processes used.	Page 13-15
	23d	Discuss implications of the results for practice, policy, and future research.	Page 15
**OTHER INFORMATION**			
Registration and protocol	24a	Provide registration information for the review, including register name and registration number, or state that the review was not registered.	Page 3
	24b	Indicate where the review protocol can be accessed, or state that a protocol was not prepared.	Page 3
	24c	Describe and explain any amendments to information provided at registration or in the protocol.	Page 3
Support	25	Describe sources of financial or non-financial support for the review, and the role of the funders or sponsors in the review.	Page 16
Competing interests	26	Declare any competing interests of review authors.	Page 16
Availability of data, code and other materials	27	Report which of the following are publicly available and where they can be found: template data collection forms; data extracted from included studies; data used for all analyses; analytic code; any other materials used in the review.	NA

Page MJ, McKenzie JE, Bossuyt PM, Boutron I, Hoffmann TC, Mulrow CD, et al. The PRISMA 2020 statement: an updated guideline for reporting systematic reviews. BMJ 2021;372:n71.

File S2 Study Quality Appraisal Tool

**Quality Appraisal Tool based on Modified Hoy et al (2012)[152]**	
Q1	Was the study's target population **a close representation** of the national population in relation to relevant variables, e.g. age, sex, occupation?
Q2	Was the sampling frame a **true or close representation** of the target population?
Q3	Was some form of **random selection** used to select the sample, OR was a census undertaken?
Q4	Was the likelihood of **non-response (or incomplete online survey responses) bias minimal**?
Q5	Were data collected **directly from the subjects** (as opposed to a proxy)?
Q6	Was an acceptable case definition used in the study?
Q7	Was the study instrument that measured the parameter of interest (i.e. peritoneal dialysis-associated peritonitis) shown to have **reliability and validity (if necessary)**?
Q8	Was the **same mode of data collection** used for all subjects?
Q9	Was the **length of the shortest prevalence period** for the parameter of interest appropriate?
Q10	Were the **numerator(s) and denominator(s)** for the parameter of interest appropriate?

File S3 eReferences

1. Balasubramanian G, McKitty K, Fan SL. Comparing automated peritoneal dialysis with continuous ambulatory peritoneal dialysis: survival and quality of life differences? *Nephrol Dial Transplant*. May 2011;26(5):1702-8. doi:10.1093/ndt/gfq607
2. Brown MC, Simpson K, Kerssens JJ, Mactier RA. Peritoneal dialysis-associated peritonitis rates and outcomes in a national cohort are not improving in the post-millennium (2000-2007). *Perit Dial Int*. Nov-Dec 2011;31(6):639-50. doi:10.3747/pdi.2010.00185
3. Davenport A, Wellsted D. Does antifungal prophylaxis with daily oral fluconazole reduce the risk of fungal peritonitis in peritoneal dialysis patients? The Pan Thames Renal Audit. *Blood Purif*. 2011;32(3):181-5. doi:10.1159/000328735
4. Huang ST, Chuang YW, Cheng CH, et al. Evolution of microbiological trends and treatment outcomes in peritoneal dialysis-related peritonitis. *Clin Nephrol*. May 2011;75(5):416-25. doi:10.5414/cnp75416
5. Kanjanabuch T, Chancharoenthana W, Katavetin P, et al. The incidence of peritoneal dialysis-related infection in Thailand: a nationwide survey. *J Med Assoc Thai*. Sep 2011;94 Suppl 4:S7-12.
6. Liawnoraset W. Prevalence and factors affecting peritonitis in CAPD patients in Maharat Nakhon Ratchasima Hospital under universal coverage scheme during 2008-2010: a three-year experience. *J Med Assoc Thai*. Sep 2011;94 Suppl 4:S19-24.
7. Luvira V, Satirapoj B, Supasyndh O, et al. A single-centre experience: peritoneal dialysis-related infections in patients on long-term dialysis. *J Med Assoc Thai*. Sep 2011;94 Suppl 4:S30-6.
8. Martin LC, Caramori JC, Fernandes N, Divino-Filho JC, Pecoits-Filho R, Barretti P. Geographic and educational factors and risk of the first peritonitis episode in Brazilian Peritoneal Dialysis study (BRAZPD) patients. *Clin J Am Soc Nephrol*. Aug 2011;6(8):1944-51. doi:10.2215/cjn.11431210
9. Muñoz de Bustillo E, Borrás F, Gómez-Roldán C, et al. Impact of peritonitis on long-term survival of peritoneal dialysis patients. *Nefrologia*. 2011;31(6):723-32. doi:10.3265/Nefrologia.pre2011.Oct.10987
10. Oygar DD, Yalin AS, Altiparmak MR, Ataman R, Serdengecti K. Obligatory referral among other factors associated with peritonitis in peritoneal dialysis patients. *Nefrologia*. 2011;31(4):435-40. doi:10.3265/Nefrologia.pre2011.Apr.10796
11. Quintanar Lartundo JA, Palomar R, Dominguez-Diez A, et al. Microbiological profile of peritoneal dialysis peritonitis and predictors of hospitalization. *Adv Perit Dial*. 2011;27:38-42.
12. Topanthanont S, Sakulsaengprapha A. Peritonitis in peritoneal dialysis patients: Ramathibodi Hospital experience. *J Med Assoc Thai*. Sep 2011;94 Suppl 4:S25-9.
13. Aksu N, Yavascan O, Anil M, Kara OD, Bal A, Anil AB. Chronic peritoneal dialysis in children with special needs or social disadvantage or both: contraindications are not always contraindications. *Perit Dial Int*. Jul-Aug 2012;32(4):424-30. doi:10.3747/pdi.2009.00202
14. Barone RJ, Cámpora MI, Gimenez NS, Ramirez L, Panese SA, Santopietro M. Continuous ambulatory peritoneal dialysis versus automated peritoneal dialysis and peritonitis in the short and very long term at risk. *Adv Perit Dial*. 2012;28:44-9.
15. Basturk T, Koc Y, Unsal A, et al. Fungal peritonitis in peritoneal dialysis: a 10 year retrospective analysis in a single center. *Eur Rev Med Pharmacol Sci*. Nov 2012;16(12):1696-700.
16. Chen A, Martz K, Rao PS. Does allograft failure impact infection risk on peritoneal dialysis: a North American Pediatric Renal Trials and Collaborative Studies Study. *Clin J Am Soc Nephrol*. Jan 2012;7(1):153-7. doi:10.2215/cjn.03160411
17. Levallois J, Nadeau-Fredette AC, Labbé AC, Laverdière M, Ouimet D, Vallée M. Ten-year experience with fungal peritonitis in peritoneal dialysis patients: antifungal susceptibility patterns in a North-American center. *Int J Infect Dis*. Jan 2012;16(1):e41-3. doi:10.1016/j.ijid.2011.09.016
18. Nayak A, Karopadi A, Antony S, Sreepada S, Nayak KS. Use of a peritoneal dialysis remote monitoring system in India. *Perit Dial Int*. Mar-Apr 2012;32(2):200-4. doi:10.3747/pdi.2011.00124

19. Oliveira LG, Luengo J, Caramori JC, Montelli AC, Cunha Mde L, Barretti P. Peritonitis in recent years: clinical findings and predictors of treatment response of 170 episodes at a single Brazilian center. *Int Urol Nephrol*. Oct 2012;44(5):1529-37. doi:10.1007/s11255-011-0107-7
20. Pierce DA, Williamson JC, Mauck VS, Russell GB, Palavecino E, Burkart JM. The effect on peritoneal dialysis pathogens of changing topical antibiotic prophylaxis. *Perit Dial Int*. Sep-Oct 2012;32(5):525-30. doi:10.3747/pdi.2011.00183
21. Rocha A, Rodrigues A, Teixeira L, Carvalho MJ, Mendonça D, Cabrita A. Temporal trends in peritonitis rates, microbiology and outcomes: the major clinical complication of peritoneal dialysis. *Blood Purif*. 2012;33(4):284-91. doi:10.1159/000337377
22. Su CY, Pei J, Lu XH, Tang W, Wang T. Gastrointestinal symptoms predict peritonitis rates in CAPD patients. *Clin Nephrol*. Apr 2012;77(4):267-74. doi:10.5414/cn107249
23. Xu R, Chen Y, Luo S, Li Y, Dong J. The influence of duration of peritoneal dialysis therapy on the outcomes of initial and subsequent peritonitis is different. *Perit Dial Int*. Jul-Aug 2012;32(4):473-6. doi:10.3747/pdi.2011.00193
24. Xu R, Zhuo M, Yang Z, Dong J. Experiences with assisted peritoneal dialysis in China. *Perit Dial Int*. Jan-Feb 2012;32(1):94-101. doi:10.3747/pdi.2010.00213
25. Yap DY, Chu WL, Ng F, Yip TP, Lui SL, Lo WK. Risk factors and outcome of contamination in patients on peritoneal dialysis--a single-center experience of 15 years. *Perit Dial Int*. Nov-Dec 2012;32(6):612-6. doi:10.3747/pdi.2011.00268
26. Yap DY, To KK, Yip TP, et al. Streptococcus bovis peritonitis complicating peritoneal dialysis--a review of 10 years' experience. *Perit Dial Int*. Jan-Feb 2012;32(1):55-9. doi:10.3747/pdi.2010.00304
27. Chern YB, Ho PS, Kuo LC, Chen JB. Lower education level is a major risk factor for peritonitis incidence in chronic peritoneal dialysis patients: a retrospective cohort study with 12-year follow-up. *Perit Dial Int*. Sep-Oct 2013;33(5):552-8. doi:10.3747/pdi.2012.00065
28. Huang JW, Lien YC, Yang CY, et al. High peritoneal KT/V and peritonitis rates are associated with peritoneal calcification. *PLoS One*. 2013;8(8):e71636. doi:10.1371/journal.pone.0071636
29. Kerschbaum J, Vychytil A, Lhotta K, et al. Treatment with oral active vitamin D is associated with decreased risk of peritonitis and improved survival in patients on peritoneal dialysis. *PLoS One*. 2013;8(7):e67836. doi:10.1371/journal.pone.0067836
30. Nessim SJ, Komenda P, Rigatto C, Verrelli M, Sood MM. Frequency and microbiology of peritonitis and exit-site infection among obese peritoneal dialysis patients. *Perit Dial Int*. Mar-Apr 2013;33(2):167-74. doi:10.3747/pdi.2011.00244
31. Özbalci N, Sezer MT, Koyuncuoğlu J, Ergonul E, Yayli G. Evaluation of peritonitis incidence, etiology, associated factors and prognosis of continuous ambulatory peritoneal dialysis patients. *Turkish Nephrology, Dialysis and Transplantation Journal*. 2013;22(2):158-162. doi:10.5262/tndt.2013.1002.04
32. Portolés J, Janeiro D, Lou-Arnal LM, et al. First episodes of peritoneal infection: description and prognostic factors. *Nefrologia*. 2013;33(3):316-24. doi:10.3265/Nefrologia.pre2013.Feb.11733
33. Tsai CC, Lee JJ, Liu TP, et al. Effects of age and diabetes mellitus on clinical outcomes in patients with peritoneal dialysis-related peritonitis. *Surg Infect (Larchmt)*. Dec 2013;14(6):540-6. doi:10.1089/sur.2012.195
34. Hsieh YP, Wang SC, Chang CC, Wen YK, Chiu PF, Yang Y. The negative impact of early peritonitis on continuous ambulatory peritoneal dialysis patients. *Perit Dial Int*. Sep-Oct 2014;34(6):627-35. doi:10.3747/pdi.2013.00024
35. Isla RA, Mapiye D, Swanepoel CR, Rozumyk N, Hubahib JE, Okpechi IG. Continuous ambulatory peritoneal dialysis in Limpopo province, South Africa: predictors of patient and technique survival. *Perit Dial Int*. Jul-Aug 2014;34(5):518-25. doi:10.3747/pdi.2013.00334
36. Kumar KV, Mallikarjuna HM, Gokulnath, Jayanthi S. Fungal peritonitis in continuous ambulatory peritoneal dialysis: The impact of antifungal prophylaxis on patient and technique outcomes. *Indian J Nephrol*. Sep 2014;24(5):297-301. doi:10.4103/0971-4065.133005
37. Najafi I, Alatab S, Atabak S, et al. Seventeen years' experience of peritoneal dialysis in Iran: first official report of the Iranian peritoneal dialysis registry. *Perit Dial Int*. Sep-Oct 2014;34(6):636-42. doi:10.3747/pdi.2012.00054

38. Nieto-Ríos JF, Díaz-Betancur JS, Arbeláez-Gómez M, et al. Peritoneal dialysis-related peritonitis: twenty-seven years of experience in a Colombian medical center. *Nefrologia*. 2014;34(1):88-95. doi:10.3265/Nefrologia.pre2013.Nov.12002
39. Nishina M, Yanagi H, Kakuta T, Endoh M, Fukagawa M, Takagi A. A 10-year retrospective cohort study on the risk factors for peritoneal dialysis-related peritonitis: a single-center study at Tokai University Hospital. *Clin Exp Nephrol*. Aug 2014;18(4):649-54. doi:10.1007/s10157-013-0872-y
40. Núñez-Moral M, Sánchez-Álvarez JE, González-Díaz I, Peláez-Requejo B, Quintana-Fernández A, Rodríguez-Suárez C. Seasonal variations and influence of the weather on the appearance of peritoneal infection. *Nefrologia*. Nov 17 2014;34(6):743-8. doi:10.3265/Nefrologia.pre2014.Jul.12420
41. Prasad KN, Singh K, Rizwan A, et al. Microbiology and outcomes of peritonitis in northern India. *Perit Dial Int*. Mar-Apr 2014;34(2):188-94. doi:10.3747/pdi.2012.00233
42. Chang TI, Kang EW, Kim HW, et al. Low Serum Bicarbonate Predicts Residual Renal Function Loss in Peritoneal Dialysis Patients. *Medicine (Baltimore)*. Aug 2015;94(31):e1276. doi:10.1097/md.0000000000001276
43. Courivaud C, Bardonnet K, Crepin T, Bresson-Vautrin C, Rebibou JM, Ducloux D. Serum immunoglobulin G levels and peritonitis in peritoneal dialysis patients. *J Nephrol*. Aug 2015;28(4):511-5. doi:10.1007/s40620-015-0176-2
44. Figueiredo AE, Moraes TP, Bernardini J, et al. Impact of patient training patterns on peritonitis rates in a large national cohort study. *Nephrol Dial Transplant*. Jan 2015;30(1):137-42. doi:10.1093/ndt/gfu286
45. Lei Y, Xiong Y, Zhang L, et al. Comparison of Long-Term Outcomes between Peritoneal Dialysis Patients with Diabetes as a Primary Renal Disease or as a Comorbid Condition. *PLoS One*. 2015;10(5):e0126549. doi:10.1371/journal.pone.0126549
46. Ozisik L, Ozdemir FN, Tanriover MD. The changing trends of peritoneal dialysis related peritonitis and novel risk factors. *Ren Fail*. Jul 2015;37(6):1027-32. doi:10.3109/0886022x.2015.1052952
47. Pi HC, Ren YP, Wang Q, Xu R, Dong J. Serum 25-Hydroxyvitamin D Level Could Predict the Risk for Peritoneal Dialysis-Associated Peritonitis. *Perit Dial Int*. Dec 2015;35(7):729-35. doi:10.3747/pdi.2014.00177
48. Sanabria M, Devia M, Hernández G, et al. Outcomes of a peritoneal dialysis program in remote communities within Colombia. *Perit Dial Int*. Jan-Feb 2015;35(1):52-61. doi:10.3747/pdi.2012.00301
49. Buttigieg J, Borg Cauchi A, Rogers M, Farrugia E, Fava S. Seasonal Variation in the Peritoneal Dialysis-Related Infections: A Single Center Experience in the Mediterranean. *Ther Apher Dial*. Oct 2016;20(5):501-506. doi:10.1111/1744-9987.12416
50. Duquennoy S, Béchade C, Verger C, Ficheux M, Ryckelynck JP, Lobbedez T. Is Peritonitis Risk Increased in Elderly Patients on Peritoneal Dialysis? Report from the French Language Peritoneal Dialysis Registry (RDPLF). *Perit Dial Int*. May-Jun 2016;36(3):291-6. doi:10.3747/pdi.2014.00154
51. El-Reshaid W, Al-Disawy H, Nassef H, Alhelaly U. Comparison of peritonitis rates and patient survival in automated and continuous ambulatory peritoneal dialysis: a 10-year single center experience. *Ren Fail*. Sep 2016;38(8):1187-92. doi:10.1080/0886022x.2016.1209025
52. Higuchi C, Ito M, Masakane I, Sakura H. Peritonitis in peritoneal dialysis patients in Japan: a 2013 retrospective questionnaire survey of Japanese Society for Peritoneal Dialysis member institutions. *Renal Replacement Therapy*. 2016;2(1):2. doi:10.1186/s41100-016-0014-6
53. Kotera N, Tanaka M, Aoe M, et al. Age-Related Differences of Organism-Specific Peritonitis Rates: A Single-Center Experience. *Ther Apher Dial*. Dec 2016;20(6):655-660. doi:10.1111/1744-9987.12449
54. Mizuno M, Ito Y, Suzuki Y, et al. Recent analysis of status and outcomes of peritoneal dialysis in the Tokai area of Japan: the second report of the Tokai peritoneal dialysis registry. *Clin Exp Nephrol*. Dec 2016;20(6):960-971. doi:10.1007/s10157-016-1249-9

55. Nakao M, Yamamoto I, Maruyama Y, et al. 33 Years of Peritoneal Dialysis-Associated Peritonitis: A Single-Center Study in Japan. *Ther Apher Dial*. Feb 2016;20(1):60-5. doi:10.1111/1744-9987.12372
56. Pai MF, Yang JY, Chen HY, et al. Comparing long-term outcomes between early and delayed initiation of peritoneal dialysis following catheter implantation. *Ren Fail*. Jul 2016;38(6):875-81. doi:10.3109/0886022x.2016.1165069
57. Pérez-Fontan M, Machado Lopes D, García Enríquez A, et al. Inhibition of Gastric Acid Secretion by H2 Receptor Antagonists Associates a Definite Risk of Enteric Peritonitis and Infectious Mortality in Patients Treated with Peritoneal Dialysis. *PLoS One*. 2016;11(2):e0148806. doi:10.1371/journal.pone.0148806
58. Perl J, Parpia S, Nessim SJ. Does the risk of peritoneal dialysis-associated peritonitis vary by time on treatment? *Clin Nephrol*. Apr 2016;85(4):209-14. doi:10.5414/cn108758
59. Sandrini M, Vizzardi V, Valerio F, et al. Incremental peritoneal dialysis: a 10 year single-centre experience. *J Nephrol*. Dec 2016;29(6):871-879. doi:10.1007/s40620-016-0344-z
60. Santhakumaran T, Samad N, Fan SL. Hydration status measured by BCM: A potential modifiable risk factor for peritonitis in patients on peritoneal dialysis. *Nephrology (Carlton)*. May 2016;21(5):404-9. doi:10.1111/nep.12622
61. Shea YF, Lam MF, Lee MS, et al. Prevalence of Cognitive Impairment Among Peritoneal Dialysis Patients, Impact on Peritonitis and Role of Assisted Dialysis. *Perit Dial Int*. May-Jun 2016;36(3):284-90. doi:10.3747/pdi.2014.00247
62. Tian Y, Xie X, Xiang S, et al. Risk factors and outcomes of high peritonitis rate in continuous ambulatory peritoneal dialysis patients: A retrospective study. *Medicine (Baltimore)*. Dec 2016;95(49):e5569. doi:10.1097/md.0000000000005569
63. Alatab S, Najafi I, Pourmand G, Hosseini M, Shekarchian S. Risk factors of severe peritoneal sclerosis in chronic peritoneal dialysis patients. *Ren Fail*. Nov 2017;39(1):32-39. doi:10.1080/0886022x.2016.1244075
64. Htay H, Johnson DW, Wu SY, Oei EL, Foo MWY, Choo JCJ. Comparison of Topical Chlorhexidine and Mupirocin for the Prevention of Exit-Site Infection in Incident Peritoneal Dialysis Patients. *Perit Dial Int*. May-Jun 2017;37(3):266-272. doi:10.3747/pdi.2016.00257
65. Lee SM, Son YK, Kim SE, An WS. Clinical Outcomes of Peritoneal Dialysis in End-Stage Renal Disease Patients with Liver Cirrhosis: A Propensity Score Matching Study. *Perit Dial Int*. May-Jun 2017;37(3):314-320. doi:10.3747/pdi.2016.00129
66. Ndlovu KC, Sibanda W, Assounga A. Peritonitis outcomes in patients with HIV and end-stage renal failure on peritoneal dialysis: a prospective cohort study. *BMC Nephrol*. Feb 3 2017;18(1):48. doi:10.1186/s12882-017-0466-0
67. Ong LM, Ch'ng CC, Wee HC, et al. Risk of Peritoneal Dialysis-Related Peritonitis in a Multi-Racial Asian Population. *Perit Dial Int*. 1-2 2017;37(1):35-43. doi:10.3747/pdi.2015.00141
68. Vargas E, Blake PG, Sanabria M, et al. Early Peritonitis in a Large Peritoneal Dialysis Provider System in Colombia. *Perit Dial Int*. 1-2 2017;37(1):30-34. doi:10.3747/pdi.2016.00030
69. Al Wakeel JS, Al Ghonaim MA, Aldohayan A, et al. Appraising the outcome and complications of peritoneal dialysis patients in self-care peritoneal dialysis and assisted peritoneal dialysis: A 5-year review of a single Saudi center. *Saudi J Kidney Dis Transpl*. Jan-Feb 2018;29(1):71-80. doi:10.4103/1319-2442.225197
70. Fang P, Lu J, Liu YH, Deng HM, Zhang L, Zhang HQ. Benefit of an operating vehicle preventing peritonitis in peritoneal dialysis patients: a retrospective, case-controlled study. *Int Urol Nephrol*. Jun 2018;50(6):1163-1170. doi:10.1007/s11255-018-1823-z
71. Hsu CK, Lee CC, Chen YT, et al. Multidisciplinary predialysis education reduces incidence of peritonitis and subsequent death in peritoneal dialysis patients: 5-year cohort study. *PLoS One*. 2018;13(8):e0202781. doi:10.1371/journal.pone.0202781
72. Ponce D, de Moraes TP, Pecoits-Filho R, Figueiredo AE, Barretti P. Peritonitis in Children on Chronic Peritoneal Dialysis: The Experience of a Large National Pediatric Cohort. *Blood Purif*. 2018;45(1-3):118-125. doi:10.1159/000484344
73. Rope R, Nanayakkara N, Wazil A, et al. Expanding CAPD in Low-Resource Settings: A Distance Learning Approach. *Perit Dial Int*. Sep-Oct 2018;38(5):343-348. doi:10.3747/pdi.2017.00251

74. Alfayez SM, Alsaqoub SM, Qattan AY, et al. Peritoneal dialysis related infections in a tertiary care hospital in Riyadh, Saudi Arabia. *Saudi Med J*. Feb 2019;40(2):147-151. doi:10.15537/smj.2019.2.23898
75. Balzer MS, Claus R, Haller H, Hiss M. Are ISPD Guidelines on Peritonitis Diagnosis Too Narrow? A 15-Year Retrospective Single-Center Cohort Study on PD-Associated Peritonitis Accounting for Untrained Patients. *Perit Dial Int*. May-Jun 2019;39(3):220-228. doi:10.3747/pdi.2018.00179
76. Einbinder Y, Cohen-Hagai K, Shitrit P, et al. ISPD guideline-driven retraining, exit site care and decreased peritonitis: a single-center experience in Israel. *Int Urol Nephrol*. Apr 2019;51(4):723-727. doi:10.1007/s11255-019-02100-w
77. Gadola L, Poggi C, Dominguez P, Poggio MV, Lungo E, Cardozo C. Risk Factors And Prevention of Peritoneal Dialysis-Related Peritonitis. *Perit Dial Int*. Mar-Apr 2019;39(2):119-125. doi:10.3747/pdi.2017.00287
78. Hu S, Tong R, Bo Y, Ming P, Yang H. Fungal peritonitis in peritoneal dialysis: 5-year review from a North China center. *Infection*. Feb 2019;47(1):35-43. doi:10.1007/s15010-018-1204-7
79. Jahromi SE, Ahmed F, Pourahmad S, Roozbeh J, Najafi I, Ebrahimi V. Predictors of peritoneal dialysis associated peritonitis; application of the zero-inflated negative binomial model. *Journal of Nephropathology*. 2019;8(4)doi:10.15171/jnp.2019.36
80. Karthikeyan B, Sharma RK, Kaul A, Gupta A, Prasad N, Bhadauria DS. Clinical Characteristics, Patient and Technique Survival in Elderly Patients on Peritoneal Dialysis. *Indian J Nephrol*. Sep-Oct 2019;29(5):334-339. doi:10.4103/ijn.IJN_377_18
81. Lasfar LB, Guedri Y, Zellama D, et al. Long-term clinical outcomes of peritoneal dialysis patients: 10-year experience of a single unit from Tunisia. *Saudi J Kidney Dis Transpl*. Mar-Apr 2019;30(2):451-461. doi:10.4103/1319-2442.256852
82. Liao JL, Zhang YH, Xiong ZB, et al. The Association of Cognitive Impairment with Peritoneal Dialysis-Related Peritonitis. *Perit Dial Int*. May-Jun 2019;39(3):229-235. doi:10.3747/pdi.2018.00180
83. Lichodziejewska-Niemierko M, Chmielewski M, Wojtaszek E, et al. Current epidemiology and practice patterns in prevention and treatment of PD-related infections in Poland. *Int Urol Nephrol*. Feb 2019;51(2):335-341. doi:10.1007/s11255-018-2057-9
84. Wang HH, Huang CH, Kuo MC, et al. Microbiology of peritoneal dialysis-related infection and factors of refractory peritoneal dialysis related peritonitis: A ten-year single-center study in Taiwan. *J Microbiol Immunol Infect*. Oct 2019;52(5):752-759. doi:10.1016/j.jmii.2018.10.013
85. Wojtaszek E, Grzejszczak A, Grygiel K, Małyszko J, Matuszkiewicz-Rowińska J. Urgent-Start Peritoneal Dialysis as a Bridge to Definitive Chronic Renal Replacement Therapy: Short- and Long-Term Outcomes. *Front Physiol*. 2018;9:1830. doi:10.3389/fphys.2018.01830
86. Bonnal H, Bechade C, Boyer A, et al. Effects of educational practices on the peritonitis risk in peritoneal dialysis: a retrospective cohort study with data from the French peritoneal Dialysis registry (RDPLF). *BMC Nephrol*. May 29 2020;21(1):205. doi:10.1186/s12882-020-01867-w
87. Hwang TY, Kim MG, Oh SW, Jo SK, Cho WY, Yang J. Pathogens of peritoneal dialysis peritonitis: Trends from a single-center experience over 15 years. *Kidney Res Clin Pract*. Jun 30 2020;39(2):221-227. doi:10.23876/j.krcp.19.035
88. Ma X, Shi Y, Tao M, et al. Analysis of risk factors and outcome in peritoneal dialysis patients with early-onset peritonitis: a multicentre, retrospective cohort study. *BMJ Open*. Feb 13 2020;10(2):e029949. doi:10.1136/bmjopen-2019-029949
89. Noppakun K, Kasemset T, Wongsawad U, et al. Changes in serum albumin concentrations during transition to dialysis and subsequent risk of peritonitis after peritoneal dialysis initiation: a retrospective cohort study. *J Nephrol*. Dec 2020;33(6):1275-1287. doi:10.1007/s40620-020-00716-1
90. Perl J, Fuller DS, Bieber BA, et al. Peritoneal Dialysis-Related Infection Rates and Outcomes: Results From the Peritoneal Dialysis Outcomes and Practice Patterns Study (PDOPPS). *Am J Kidney Dis*. Jul 2020;76(1):42-53. doi:10.1053/j.ajkd.2019.09.016

91. Tekkarişmaz N, Torun D. Long-term clinical outcomes of peritoneal dialysis patients: 9-year experience of a single centre in Turkey. *Turk J Med Sci*. Apr 9 2020;50(2):386-397. doi:10.3906/sag-1909-98
92. Viglino G, Neri L, Barbieri S, Tortone C. Videodialysis: a pilot experience of telecare for assisted peritoneal dialysis. *J Nephrol*. Feb 2020;33(1):177-182. doi:10.1007/s40620-019-00647-6
93. Zeng Y, Jiang X, Feng S, et al. The influence of seasonal factors on the incidence of peritoneal dialysis-associated peritonitis. *Ren Fail*. Nov 2020;42(1):807-817. doi:10.1080/0886022x.2020.1804401
94. AlZabli SM, Alsuhaibani MA, BinThunian MA, et al. Peritonitis in children on peritoneal dialysis: 12 years of tertiary center experience. *Int J Pediatr Adolesc Med*. Dec 2021;8(4):229-235. doi:10.1016/j.ijpam.2020.09.001
95. de la Espada Piña V, Ganga PLQ, Junquero JMG, et al. Two decades of analysis of peritonitis in peritoneal dialysis in Andalusia: Epidemiological, clinical, microbiological and progression aspects. *Nefrologia (Engl Ed)*. Jul-Aug 2021;41(4):417-425. doi:10.1016/j.nefroe.2021.10.004
96. Htay H, Choo JCJ, Johnson DW, et al. Chlorhexidine-impregnated sponge dressing for prevention of catheter exit-site infection in peritoneal dialysis patients: a pilot study. *Int Urol Nephrol*. Apr 2021;53(4):803-812. doi:10.1007/s11255-020-02674-w
97. Hu J, Zhang H, Yi B. Peritoneal transport status and first episode of peritonitis: a large cohort study. *Ren Fail*. Dec 2021;43(1):1094-1103. doi:10.1080/0886022x.2021.1949350
98. Kang M, Kim YL, Kang E, et al. Evolving outcomes of peritoneal dialysis: secular trends at a single large center over three decades. *Kidney Res Clin Pract*. Sep 2021;40(3):472-483. doi:10.23876/j.krcp.21.020
99. Li R, Zhang D, He J, et al. Characteristics Analysis, Clinical Outcome and Risk Factors for Fungal Peritonitis in Peritoneal Dialysis Patients: A 10-Year Case-Control Study. *Front Med (Lausanne)*. 2021;8:774946. doi:10.3389/fmed.2021.774946
100. Liu D, Lin Y, Gong N, et al. Degree and duration of hypokalemia associated with peritonitis in patients undergoing peritoneal dialysis. *Int J Clin Pract*. Aug 2021;75(8):e14188. doi:10.1111/ijcp.14188
101. Neu AM, Richardson T, De Souza HG, et al. Continued reduction in peritonitis rates in pediatric dialysis centers: results of the Standardizing Care to Improve Outcomes in Pediatric End Stage Renal Disease (SCOPE) Collaborative. *Pediatr Nephrol*. Aug 2021;36(8):2383-2391. doi:10.1007/s00467-021-04924-0
102. Oki R, Tsuji S, Hamasaki Y, et al. Time until treatment initiation is associated with catheter survival in peritoneal dialysis-related peritonitis. *Sci Rep*. Mar 22 2021;11(1):6547. doi:10.1038/s41598-021-86071-y
103. Sakurada T, Fujishima R, Yamada S, et al. Seasonality of peritoneal dialysis-related peritonitis in Japan: a single-center, 10-year study. *Clin Exp Nephrol*. Jan 2021;25(1):52-57. doi:10.1007/s10157-020-01953-1
104. Santos JE, Rodríguez Magariños C, García Gago L, et al. Long-term trends in the incidence of peritoneal dialysis-related peritonitis disclose an increasing relevance of streptococcal infections: A longitudinal study. *PLoS One*. 2020;15(12):e0244283. doi:10.1371/journal.pone.0244283
105. Song Q, Yan H, Yu Z, et al. Assisted peritoneal dialysis: a feasible KRT modality for frail older patients with end-stage kidney disease (ESKD). *Sci Rep*. Jul 22 2021;11(1):14928. doi:10.1038/s41598-021-94032-8
106. Tangjittrong K. Risk Factors for The First Episode of Peritonitis in Continuous Ambulatory Peritoneal Dialysis Patients in Pranangklao Hospital. *J Med Assoc Thai*. 2021;104(12):1966-1970. doi:10.35755/jmedassocthai.2021.12.13194
107. Witoon R, Yongsiri S, Wanitchanont A, Chaivanit P. Effect of glomerular filtration rate at peritoneal dialysis initiation on clinical outcomes: A retrospective cohort study. *J Med Assoc Thai*. 2021;104(1):10-17. doi:10.35755/jmedassocthai.2021.1.10806
108. Cheikh Hassan HI, Murali K, Lonergan M, et al. Association of Peritonitis With Cardiovascular Mortality Over Time in the Peritoneal Dialysis Population: An Australia and New Zealand Dialysis and Transplant Registry Study. *Kidney Int Rep*. Nov 2022;7(11):2388-2396. doi:10.1016/j.ekir.2022.08.008

109. Kanjanabuch T, Puapatanakul P, Halue G, et al. Implementation of PDOPPS in a middle-income country: Early lessons from Thailand. *Perit Dial Int*. Jan 2022;42(1):83-91. doi:10.1177/0896860821993950
110. Kolland M, Ribitsch W, Prenner A, et al. Effluent microscopy is a useful adjuvant diagnostic tool in peritoneal dialysis-associated peritonitis. *Clin Nephrol*. Jul 2022;98(1):33-41. doi:10.5414/cn110732
111. Lau SY, Bee BC, Wong HS, Omar MS, Bahari N. Predictive Factors, Treatment, and Outcomes of Coagulase-Negative Staphylococcal Peritonitis in Malaysian Peritoneal Dialysis Patients: A Single-Center Study. *Int J Nephrol*. 2022;2022:8985178. doi:10.1155/2022/8985178
112. Melanson J, Kachmar J, Laurin LP, Elftouh N, Nadeau-Fredette AC. Assisted Peritoneal Dialysis Implementation: A Pilot Program From a Large Dialysis Unit in Quebec. *Can J Kidney Health Dis*. 2022;9:20543581221113387. doi:10.1177/20543581221113387
113. Özbek Ö, Akdam H, Öncü S, Yeniçerioğlu Y, Öncü S. Peritoneal dialysis related peritonitis; a single center results. *Revista de Nefrologia, Dialisis y Trasplante*. 2022;42(3):206-214.
114. Szeto CC, Ng JK, Fung WW, et al. Excessive risk and poor outcome of hospital-acquired peritoneal dialysis-related peritonitis. *Clin Kidney J*. Nov 2022;15(11):2107-2115. doi:10.1093/ckj/sfac164
115. Wu H, Yi C, Zhang D, et al. Changes of antibiotic resistance over time among Escherichia coli peritonitis in Southern China. *Perit Dial Int*. Mar 2022;42(2):218-222. doi:10.1177/08968608211045272
116. Zhai Y, Zhou Q, Fang X, et al. Reduction in peritonitis rates: 18-year results from the most active pediatric peritoneal dialysis center in China. *Pediatr Nephrol*. Oct 2022;37(10):2437-2448. doi:10.1007/s00467-022-05450-3
117. Alves CA, Cálice-Silva V, Rufino EM, et al. Urgent-Start Peritoneal Dialysis: Brazilian Experience. *Blood Purif*. 2023;52(6):556-563. doi:10.1159/000529133
118. Çankaya E, Altunok M, Yağanoğlu AM. The effect of rural and urban life on peritonitis rates in chronic peritoneal patients. *Ren Fail*. Dec 2023;45(1):2163504. doi:10.1080/0886022x.2022.2163504
119. Chienwichai K, Sangaew S, Chuachanpipat L, Chang A. Comparison of clinical outcomes between culture-negative and positive peritonitis in patients undergoing maintenance peritoneal dialysis: a prospective cohort study. *BMC Nephrol*. Nov 17 2023;24(1):340. doi:10.1186/s12882-023-03389-7
120. Forté V, Novelli S, Zaidan M, Snanoudj R, Verger C, Beaudreuil S. Microbiology and outcomes of polymicrobial peritonitis associated with peritoneal dialysis: a register-based cohort study from the French Language Peritoneal Dialysis Registry. *Nephrol Dial Transplant*. May 4 2023;38(5):1271-1281. doi:10.1093/ndt/gfac267
121. Guo S, Wu H, Ji J, et al. Association between gut microbial diversity and technique failure in peritoneal dialysis patients. *Ren Fail*. Dec 2023;45(1):2195014. doi:10.1080/0886022x.2023.2195014
122. Pak WLW, Chan KL, Chan Z, et al. Device-assisted continuous ambulatory peritoneal dialysis: A single-centre experience. *Perit Dial Int*. Jan 2023;43(1):92-99. doi:10.1177/08968608221085430
123. Rivera AS, Vesga JI, Páez S, et al. Early outcomes of peritoneal dialysis in a middle-income country: A retrospective cohort study of a large dialysis network in Colombia. *Perit Dial Int*. Nov 2023;43(6):467-474. doi:10.1177/08968608231198977
124. Srivastava AK, Ghosh I, Sonawane S. Clinical profile and microbiological spectrum of patients with continuous ambulatory peritoneal dialysis-associated peritonitis at a tertiary care center. *Med J Armed Forces India*. Dec 2023;79(Suppl 1):S175-s180. doi:10.1016/j.mjafi.2022.05.001
125. Taher A, Enaya A, Sarsour A, et al. Growing a peritoneal dialysis program in Palestine from zero to 178 patients in 5 years: a single-center experience. *BMC Nephrol*. Jan 26 2023;24(1):21. doi:10.1186/s12882-023-03064-x
126. Yang TY, Tian YC, Yen TH, Chang MY, Lin CY, Liu SH. Tuberculous peritonitis in patients on peritoneal dialysis: a 35-year experience from a large medical center in Northern Taiwan. *Ren Fail*. Dec 2023;45(1):2153064. doi:10.1080/0886022x.2022.2153064

127. Ye YJ, Lou JJ, Zhang YN, Lou XY, Chen Q. Is simply washing hands before dialysis procedures sufficient for reducing peritoneal dialysis peritonitis?-A single center study from 2015 to 2020 in Yiwu, China. *Ther Apher Dial*. Apr 2023;27(2):335-342. doi:10.1111/1744-9987.13924
128. Aldriwesh MG, Alaqeel RA, Alangari RO, et al. Microbiology and Antimicrobial Resistance Profile of Infectious Peritonitis: A Cohort Study with 10-year Follow-up. *Journal of Nature and Science of Medicine*. 2024;7(1):35-41. doi:10.4103/jnsm.jnsm_4_23
129. Centellas-Pérez FJ, Ortega-Cerrato A, Vera M, et al. Impact of Remote Monitoring on Standardized Outcomes in Nephrology-Peritoneal Dialysis. *Kidney Int Rep*. Feb 2024;9(2):266-276. doi:10.1016/j.ekir.2023.10.034
130. Dandecha P, Phongphithakchai A. Effect of Exchange Systems and Procedure on Long Term Peritonitis in ESKD Patients Undergoing CAPD :A Retrospective Comparative Cohort Study. *Iran J Kidney Dis*. Aug 20 2024;18(4):212-220.
131. Dogan I, Ucar E, Odabas M, et al. The effect of hand fine motor skills on peritoneal dialysis-related peritonitis. *Ther Apher Dial*. Apr 2024;28(2):284-296. doi:10.1111/1744-9987.14079
132. Dong X, Xie C, Yi C, et al. Clinical characteristics and antibiotic treatment of peritoneal dialysis-associated peritonitis caused by Pseudomonas species: a review of 15 years' experience from southern China. *Microbiol Spectr*. Jun 4 2024;12(6):e0009624. doi:10.1128/spectrum.00096-24
133. Gül CB. Peritonitis in patients on peritoneal dialysis: a 12-year experience from a large medical center in Bursa. *European Research Journal*. 2024;10(1):92-96. doi:10.18621/eurj.1372796
134. Ito Y, Tu C, Yamaguchi M, et al. Risk factors of peritoneal dialysis-related peritonitis in the Japan Peritoneal Dialysis Outcomes and Practice Patterns Study (PDOPPS). *Clin Kidney J*. Jul 2024;17(7):sfae202. doi:10.1093/ckj/sfae202
135. Liang Q, Zhao H, Wu B, et al. Risk Factors of the Occurrence and Treatment Failure of Refractory Peritoneal Dialysis-Associated Peritonitis: A Single-Center Retrospective Study From China. *Semin Dial*. Sep-Oct 2024;37(5):386-392. doi:10.1111/sdi.13202
136. Mehrotra R, Williamson DE, Betts CR, et al. A Prospective Clinical Study to EvaluAte the AbiliTy of the CloudCath System to Detect Peritonitis During In-Home Peritoneal Dialysis (CATCH). *Kidney Int Rep*. Apr 2024;9(4):929-940. doi:10.1016/j.ekir.2024.01.033
137. Nabil H, Sara EM, Hajar F, Naima O, Loubna B. Repeat peritonitis in peritoneal dialysis: A cohort study. *Bulletin de la Dialyse a Domicile*. 2024;7(1)doi:10.25796/bdd.v7i1.83363
138. Nardelli L, Scalamogna A, Ponzano F, et al. Peritoneal dialysis related peritonitis: insights from a long-term analysis of an Italian center. *BMC Nephrol*. May 11 2024;25(1):163. doi:10.1186/s12882-024-03594-y
139. Park PG, Ahn YH, Kang HG, Ha IS. Decreased incidence of peritoneal dialysis-associated peritonitis in young children. *J Paediatr Child Health*. Oct 2024;60(10):531-537. doi:10.1111/jpc.16630
140. Tobijaszewska M, Martus G, Sunnerhagen T, Segelmark M, Ljungquist O. A population-based study on the incidence and aetiology of infectious complications in peritoneal dialysis in South Sweden. *Infect Dis (Lond)*. Mar 2024;56(3):230-243. doi:10.1080/23744235.2023.2292133
141. Alfano G, Pittiglio M, Ferri C, et al. Screening for nasal carriage of Staphylococcus (S.) aureus reduces the risk of S. aureus peritonitis in patients on peritoneal dialysis (PD). *Int Urol Nephrol*. May 2025;57(5):1639-1648. doi:10.1007/s11255-024-04309-w
142. Andrew W, Michael C, Daniel C, et al. Peritoneal dialysis catheter insertion in patients with obesity: a cohort study and single centre approach to increasing uptake. *BMC Nephrol*. Jul 1 2025;26(1):293. doi:10.1186/s12882-025-04210-3
143. Guo L, Karim SA, Qian H, et al. Retrospective Analysis of Multi-Center Peritoneal Dialysis-Associated Peritonitis in Jiangsu Province: A Retrospective Analysis from 2016 to 2024. *J Nat Sci Biol Med*. 2025;16(1):201-210. doi:10.5281/zenodo.15346972
144. Kim JH, Kim SM, Kang M, et al. Characteristics of patients and facility of peritoneal dialysis in Korea: Results from the Peritoneal Dialysis Outcomes and Practice Patterns Study (PDOPPS) Korea. *Perit Dial Int*. Sep 2025;45(5):281-290. doi:10.1177/08968608241252015
145. Soetendorp H, Grupper A, Hazan E, Wasserman A, Schwartz D, Kliuk-Ben Bassat O. Reduction of peritoneal dialysis associated infections using a novel exit-site care practice. *Perit Dial Int*. Mar 2025;45(2):113-120. doi:10.1177/08968608241270296

146. Tsuchiya Y, Matsuo N, Masuda N, Maruyama Y, Tanno Y, Yokoo T. Clinical impact of device connections on PD-related peritonitis. *Clin Exp Nephrol*. Sep 2025;29(9):1279-1285. doi:10.1007/s10157-025-02684-x
147. Yu X, Wang J, Liang C, et al. Elevated effluent lactate independently predicts the adverse outcomes of peritoneal dialysis associated peritonitis. *Sci Rep*. Jul 5 2025;15(1):24009. doi:10.1038/s41598-025-09876-1
148. Al Laham J, Alshaaili K, Elsayed HME, Al-Kharusi AW, Al Kharusi K, Al Aamri K. Fungal Peritonitis in Peritoneal Dialysis: An 18-year Experience from Nizwa Hospital, Sultanate of Oman. *Saudi J Med Med Sci*. Jan-Mar 2026;14(1):75-79. doi:10.4103/sjmms.sjmms_534_25
149. Cho HG, Lee JG, Park JW, et al. Characteristics of peritoneal dialysis-related infection according to pet ownership: An 8-year single-center experience. *PLoS One*. 2026;21(5):e0348012. doi:10.1371/journal.pone.0348012
150. Okamoto K, Matsui M, Eriguchi M, Samejima KI, Tsuruya K. Association of nephrology referral timing with long-term survival and peritoneal dialysis-associated peritonitis in patients initiating peritoneal dialysis: a single-center retrospective study. *BMC Nephrol*. Apr 6 2026;27(1)doi:10.1186/s12882-026-04956-4
151. Page MJ, McKenzie JE, Bossuyt PM, et al. The PRISMA 2020 statement: an updated guideline for reporting systematic reviews. *BMJ*. Mar 29 2021;372:n71. doi:10.1136/bmj.n71
152. Hoy D, Brooks P, Woolf A, et al. Assessing risk of bias in prevalence studies: modification of an existing tool and evidence of interrater agreement. *J Clin Epidemiol*. Sep 2012;65(9):934-9. doi:10.1016/j.jclinepi.2011.11.014
